# Associations between recent intimate partner violence and receipt and quality of perinatal health services in Uttar Pradesh

**DOI:** 10.1371/journal.pone.0232079

**Published:** 2020-05-14

**Authors:** Jay G. Silverman, Ruvani W. Fonseka, Nabamallika Dehingia, Sabrina C. Boyce, Dharmendra Chandurkar, Kultar Singh, Katherine Hay, Yamini Atmavilas, Anita Raj

**Affiliations:** 1 Center on Gender Equity and Health (GEH), University of California, San Diego, California, United States of America; 2 Sambodhi Research and Communications Pvt. Ltd., Noida, Uttar Pradesh, India; 3 Bill & Melinda Gates Foundation Seattle Office, Seattle, Washington, United States of America; 4 Bill & Melinda Gates Foundation India Country Office, Delhi, Uttar Pradesh, India; Ben-Gurion University of the Negev Faculty of Health Sciences, ISRAEL

## Abstract

**Background:**

India suffers some of the highest maternal and neonatal mortality rates in the world. Intimate partner violence (IPV) can be a barrier to utilization of perinatal care, and has been associated with poor maternal and neonatal health outcomes. However, studies that assess the relationship between IPV and perinatal health care often focus solely on receipt of services, and not the quality of the services received.

**Methods and findings:**

Data were collected in 2016–2017 from a representative sample of women (15-49yrs) in Uttar Pradesh, India who had given birth within the previous 12 months (N = 5020), including use of perinatal health services and past 12 months experiences of physical and sexual IPV. Multivariate logistic regression models assessed whether physical or sexual IPV were associated with perinatal health service utilization and quality.

Reports of IPV were not associated with odds of receiving antenatal care or a health worker home visit during the third trimester, but physical IPV was associated with fewer diagnostic tests during antenatal visits (beta = -0.30), and fewer health topics covered during home visits (beta = -0.44). Recent physical and recent sexual IPV were both associated with decreased odds of institutional delivery (physical IPV AOR 0.65; sexual IPV AOR 0.61), and recent sexual IPV was associated with leaving a delivery facility earlier than recommended (AOR = 1.87). Neither form of IPV was associated with receipt of a postnatal home visit, but recent physical IPV was associated with fewer health topics discussed during such visits (beta = -0.26).

**Conclusions:**

In this study, reduced quantity and quality of perinatal health care were associated with recent IPV experiences. In cases where IPV was not related to care receipt, IPV remained associated with diminished care quality. Additional study to understand the mechanisms underlying associations between IPV and care qualities is required to inform health services.

## Introduction

More than 7,000 newborns and 830 women die each day due to neonatal and maternal complications [1,2], prompting the UN to make lowering global maternal and neonatal mortality key targets of the Sustainable Development Goals for 2030 [3]. India accounts for over 1 in 7 global maternal deaths (45,000 women) and over 1 in 4 global neonatal deaths (630,000 infants) each year [4].

Perinatal health interventions (interventions occurring before, during, and after birth) can greatly decrease the risks of maternal and neonatal mortality [5]. Three perinatal health interventions key to reducing maternal and neonatal mortality are high quality antenatal care ANC), delivery by trained medical providers, and high-quality postnatal care (PNC) [6]. Typical indicators of adequacy of antenatal care, including those utilized in major studies of the associations of care to morbidity and mortality, focus solely on quantity of such care received, e.g., receipt of a minimum of four ANC visits [6,7]. However, quality of ANC is known to be critical to maternal and neonatal survival, independent of quantity (number of visits), and quality, rather than quantity of perinatal care has been described recently by the WHO as the greatest barrier to reaching global development goals [8,9].

One factor in women’s lives that occurs outside of the realm of health care, but that is seen to relate to both access to care and poor maternal and neonatal outcomes is intimate partner violence (IPV). Globally, IPV has been associated with insufficient ANC and not giving birth with assistance from skilled providers [10–12]. IPV during pregnancy has also been associated with lack of maternal weight gain and low birthweight [13–15]. In India, 28 percent of women in India experience intimate partner violence (IPV) during their pregnancy [16], and women experiencing this perinatal abuse are less likely to receive adequate and timely ANC [17], and more likely to have premature rupture of membranes [18]. Research among women in India also indicates that IPV relates to greater odds of major infant morbidities (e.g., respiratory distress, fever, and vomiting), and lower odds of neonates receiving critical forms of preventative care (e.g., delayed bathing and exclusive breastfeeding) [19,20].

As with most studies of receipt of health care and maternal and child morbidity, published quantitative research on perinatal care and IPV has solely focused on quantities of care, rather than qualities of care received [10–12]. The majority of such studies have included antenatal care and delivery but have not assessed IPV as a potential barrier to receipt of postnatal care [10–12]. Finally, few studies of the relationship between IPV and perinatal health have assessed the independent contributions of physical IPV and sexual IPV in association with perinatal health and health care, either focusing solely on physical IPV or combining physical and sexual IPV in their analyses [21,22].

Uttar Pradesh, India's most populous state (204 million), has a maternal mortality rate (MMR) of 258 to 345 maternal deaths per 100,000 live births, much higher than the national MMR of 174, and a neonatal mortality rate (NMR) of 50 deaths in the first month of life per 1000 live births, nearly twice the national NMR of 28 [4,23]. At these elevated rates, Uttar Pradesh, with 3 other states in India (Madhya Pradesh, Bihar, and Rajasthan) contributes 1 in 7 global neonatal deaths each year [24]. Forty percent of women in Uttar Pradesh report ever experiencing intimate partner violence (IPV) from their current husband, with 20 percent reporting this form of abuse during the last twelve months [25].

This current study assesses the independent and joint contributions of physical and sexual IPV during the perinatal period on both quantities and qualities of perinatal care received, including ANC, delivery and postnatal care, among a large population-based sample of women in Uttar Pradesh, India who had a live birth during the preceding 12 months.

## Methods

All participants agreeing to participate in the study provided formal oral consent prior to the survey. The study received its Institutional Review Board (IRB) approval from Public Health Service- Ethical Review Board (PHS-ERB), the Health Ministry Screening Committee’s (HMSC) Indian Council for Medical Research (ICMR) and the Clinical Trial Registry–India (Reference number CTRI/2015/09/006219). Data analyzed in the current study were collected as part of a midline evaluation of the Uttar Pradesh Technical Support Unit (UP-TSU), an intervention focused on support of the public health system in UP. The data were collected from 250 blocks (geographic areas including a population of approximately 100,000) of 49 districts in UP; the design is described elsewhere in Seth et. al [26]. The 250 blocks were selected from the list of all blocks within the 49 districts using simple random sampling. The geographic catchment area of a community health worker, known as ASHA in India (one ASHA catchment area covers around 1000 households), was the primary sampling unit for this survey. Six ASHA catchment areas were selected from within each of the 250 blocks, again using simple random sampling. A census of all the households within the selected ASHA areas was conducted to identify women aged 15–49 years who had a live birth in last 12 months. A total of 12,000 eligible women were approached and 9,294 consented and gave interviews. Data on IPV were collected from every second woman interviewed in the study, with only one woman per household selected for participation, resulting in a final N of 5020 women. Data were collected from June to September 2016 from these ASHA areas.

The tools were pilot tested and revised based on the findings. The pilot study was conducted in eight ASHA catchment areas. The eight areas were in the selected 250 blocks, but not a part of the ASHA catchment areas selected for the final survey. Four women from each of the eight ASHA catchment areas were randomly selected for interviews during the pilot study.

Given the sensitive nature of the information collected in this study, only female staff conducted interviews with the participating women from June to October 2016. The staff were trained in sensitive assessment by senior researchers who were experienced in conducting studies on gender-based violence. Complete privacy was ensured during each interview and if indications of immediate lethal risk was indicated, the survey was to be stopped. However, no such incidents occurred. Informed formal verbal consent was obtained from every participating woman in the study. All eligible women were informed in detail about the aims and significance of the research, and asked if they consented to being interviewed. The investigators recorded the response of all eligible women, and only those who consented were interviewed. All participants were interviewed individually in a private setting with the interviews lasting for around 60 minutes. Only one woman per household was interviewed. Data was collected on mobile handheld devices and included no identifiable information; individuals were not tracked for purposes of the evaluation. These protocols were reviewed and accepted by Public Health Service- Ethical Review Board (PHS-ERB), the Health Ministry Screening Committee’s (HMSC) Indian Council for Medical Research (ICMR) and the Clinical Trial Registry–India (Reference number CTRI/2015/09/006219). PHS-ERB is an independent ethical review board in India.

### Measures

Adequate quantity of ANC was assessed based on women’s report of receiving at least four ANC visits during their recent pregnancy, either at home or at a health facility, as per current WHO guidance [27]. A continuous variable indicating quality of ANC received was developed based on the reported number of the six WHO recommended ANC tests (weight, BP, abdomen, ultrasound, hemoglobin, and urine) reported. Receipt of any home visit by a frontline health worker (FLW) during the last trimester of the recent pregnancy was assessed via a single item. In India, every village has three community health workers, ASHA, Anganwadi Worker, and Auxiliary Nurse Midwife. These three are collectively referred to as FLWs. The FLWs are responsible for providing pregnant women and young mothers with basic care, including home-visits to provide counseling. Quality of home visits by FLWs was indicated by the number of health topics (potential topics were: taking rest, eating healthy food, diet diversity, quantity of food to consume, weight to gain, minimum 4 ANC check-ups, TT injections, need for IFA tablets during pregnancy, consumption of calcium tablets, planning for a skilled birth attendant, obtaining a new blade for delivery, obtaining a new thread for delivery, saving money for potential complications, delivering in a health facility, identifying transportation to go to the facility, keeping important phone numbers handy, staying in the health facility for 48 hours, information on danger signs) that women reported were covered by FLWs at home visits during pregnancy.

Institutional delivery was assessed based on a single item regarding whether a woman had the recent live birth either at home or at a birth facility (government health facility, privately owned hospital/clinic inclusive of skilled birth attendants, or an NGO hospital/clinic for the index childbirth). Quality of care hypothesized to relate to IPV was assessed via a single items regarding whether the woman left the birth facility earlier than recommended by an attending health provider.

Postnatal care indicators a single item asking whether a woman had received any home visit from an FLW within 24 hours of delivery or discharge from a birth facility. Quality of postnatal care was considered as a continuous variable based on the number of topics (exclusive breastfeeding till six months, positioning of baby for breastfeeding, managing breastfeeding problems, frequency of breastfeeding, skin to skin contact, danger signs for mother post childbirth, danger signs for baby, keeping the baby warm and immunization schedule for the child) discussed by an FLW across all home visits occurring within two months of childbirth.

Physical intimate partner violence was indicated by a positive response to whether any of the following acts was perpetrated in the past 12 months by the women’s current husband: slapped her, twisted her arm or pulled her hair; pushed her, shook her or threw something at her, kicked her, dragged her or beat her up; hit her with his fist or with something that could hurt her; and threatened or attacked her with a weapon. Sexual IPV was, similarly, indicated by a ‘yes’ response to any of the following forms of abused reported to have been experienced in the previous 12 months: physically forced her to have sexual intercourse with him even when she did not want to; physically forced her to perform other sexual acts that she did not want to; used threats or other actions to make her perform sexual acts that she did not want to; had sexual intercourse when she did not want to because she was afraid of what her husband might do if she refused; forced her to do something sexual that she found degrading or humiliating. Both IPV assessments were based on items in the taken from India’s National Family Health Survey-3 [28].

Socio-demographic variables included age, age at first marriage (categorized as <18 or ≥18), household wealth, literacy, spouse education, caste and religion, and parity (categorized as 1, 2, 3+ births). The Standard of Living Index (SLI) was used as a proxy indicator for characterizing household wealth; the SLI methodology is used for this purpose in the Demographic and Health Surveys across multiple national contexts, including India [29]. SLI scores were considered as quartiles (0–24, 25–49, 50–74, and 75–100; range = 0–100). A woman was considered literate if she reported being able to both read and write in at least one language. Personal and spousal education was considered dichotomously based on reports of whether or not each had completed primary education (i.e., completed school through 5th grade). Caste and religion was categorized as either Scheduled Caste/Scheduled Tribe (SC/ST), Muslim, or neither SC/ST nor Muslim.

### Statistical analysis

Descriptive analyses were used to characterize the sample, both overall, by whether or not they experienced physical IPV, sexual IPV, and each health service outcome. In all analyses, physical and sexual IPV were considered as separate independent variates, as they were not found to be collinear, and to allow estimation of the independent effects of each form of IPV, regardless of co-occurrence. Chi square analyses were used to assess associations. Multivariate regression models (logistic for binary outcomes, linear for continuous outcomes) were developed to determine whether the IPV predictors were associated with each outcome. The models were assessed for goodness-of-fit. Models were adjusted for age, age at marriage, caste/religion, wealth, literacy, personal and husband’s education, and parity based on their known associations with receipt of health care. Analyses regarding quality of health services excluded women who did not receive that service. Sample weights calculated based on the multistage sampling design were utilized in all analyses. Data were analyzed using STATA 13.0 software [30].

## Results

Approximately 1 in 10 women having recently given birth (11%) received the minimum recommended four ANC visits or more (see Table 1). The quality of ANC, as measured by the number of tests received, averaged 4.25 across the sample. Both ANC-related outcomes were associated with older age at marriage, wealth, literacy, spousal education, and having fewer children. Half of women in the current sample (51%) reported receiving a home visit by an FLW during their last trimester of pregnancy, with literacy, and spousal education, and marginally associated with older age at marriage (p < .1). Greater quality of FLW home visits based on number of topics discussed was associated with not experiencing physical IPV in the past 12 months.

**Table 1:** Demographics of women having a live birth in the past 12 months in the 25 highest-need districts of Uttar Pradesh, India by antenatal health service outcomes (unweighted ns and weighted percentages; N = 5020).

		Total		≥ 4 ANC		Number of ANC tests		Home visit during 3rd trimester		Number of topics discussed at home visits	
Characteristic		n	(%)	n	(%)	Mean	(SE)	n	(%)	Mean	(SE)
Total		**5020**	**(100)**	**600**	**(11.32)**	**4.25**	**(0.05)**	**2584**	**(50.50)**	**4.19**	**(0.09)**
*Background Characteristics*											
Age											
	15–24	1658	(32.96)	229	(13.93)	4.44	(0.07)	851	(50.35)	4.16	(0.15)
	25–29	2155	(42.67)	257	(13.94)	4.21	(0.07)	1130	(51.51)	4.27	(0.13)
	30+	1207	(24.37)	114	(13.95)	4.01	(0.09)	603	(49.02)	4.06	(0.18)
Age at marriage											
	less than 18	1300	(24.65)	123	(8.35)*	3.99	(0.11)*	607	(46.73)^	4.15	(0.19)
	18+	3720	(75.40)	476	(12.29)	4.32	(0.06)	1977	(51.76)	4.20	(0.11)
Wealth quartile											
	1 (poorest)	1426	(28.66)	116	(8.50)*	3.71	(0.09)*	691	(47.41)	4.01	(0.17)
	2	2462	(48.11)	271	(10.28)	4.27	(0.07)	1280	(50.80)	4.33	(0.14)
	3	1072	(22.05)	197	(16.89)	4.66	(0.08)	581	(53.41)	4.09	(0.18)
	4 (wealthiest)	60	(1.17)	15	(17.85)	5.29	(0.27)	32	(61.04)	4.14	(0.82)
Literacy											
	Illiterate	2442	(48.70)	195	(7.82)*	3.95	(0.09)*	1208	(48.28)*	4.03	(0.13)
	Literate	2578	(51.30)	196	(14.63)	4.47	(0.06)	1376	(52.65)	4.33	(0.12)
Spouse education											
	≤ 5th standard	1194	(23.09)	95	(7.12)*	3.85	(0.12)*	566	(45.83)*	4.07	(0.19)
	> 5th standard	3826	(76.91)	504	(12.58)	4.35	(0.06)	2018	(51.93)	4.22	(0.11)
Caste/religion											
	Neither SC/ST nor Muslim	2891	(54.94)	359	(11.48)	4.36	(0.06)	1483	(49.76)	4.15	(0.11)
	SC/ST	1372	(28.46)	142	(10.31)	3.93	(0.10)	700	(50.59)	4.15	(0.16)
	Muslim	757	(16.60)	98	(12.52)	4.38	(0.12)	401	(52.94)	4.36	(0.22)
Parity											
	1	1411	(27.43)	231	(16.20)*	4.54	(0.07)	726	(51.25)	4.29	(0.17)
	2	1391	(28.22)	154	(11.67)	4.34	(0.08)	723	(49.84)	4.13	(0.15)
	3+	2218	(44.35)	214	(8.08)	3.97	(0.08)	1135	(50.51)	4.16	(0.13)
Physical IPV in past 12 months											
	No	2740	(54.38)	314	(11.16)	4.39	(0.08)	1496	(52.83)	4.44	(0.14)*
	Yes	2280	(45.62)	286	(11.58)	4.07	(0.08)	1088	(47.78)	3.96	(0.11)
Sexual IPV in past 12 months											
	No	4537	(90.58)	537	(11.29)	4.26	(0.06)	2345	(50.60)	4.16	(0.09)
	Yes	483	(9.42)	63	(11.94)	4.13	(0.16)	239	(49.76)	4.43	(0.36)

SE denotes "Standard Error,"

*p-value <0.05

^p-value <0.1 and ≥0.05

Across the sample, almost three-quarters of women (73%) reported having given birth at a birth facility and not at home. Institutional delivery was associated with younger age, older age at marriage, wealth, literacy, spousal education, not being of SC/ST, lower parity, and not experiencing physical or sexual IPV in the past 12 months (see Table 2). Quality of delivery care was indicated by whether a woman left the facility earlier than recommended by health care providers; 1 in 5 women (20%) who delivered institutionally reported doing so. Leaving a birth facility earlier than recommended was associated with being in the poorest wealth quartile and having experienced sexual IPV in the past 12 months; being under 18 years at marriage and having a spouse educated beyond 5^th^ grade were both marginally associated with leaving a birth facility earlier than recommended (p < .1).

**Table 2:** Demographics of women having a live birth in the past 12 months in the 25 highest-need districts of Uttar Pradesh, India by delivery and postnatal health service outcomes (unweighted ns and weighted percentages; N = 5020).

		Institutional delivery		Left facility earlier than recommended		< 24 hours postnatal visit		No. topics discussed at postnatal visits	
Characteristic		n	(%)	n	(%)	n	(%)	Mean	(SE)
Total		**3679**	**(73.28)**	**800**	**(20.09)**	**1144**	**(21.6)**	**2.34**	**(0.08)**
*Background Characteristics*									
Age									
	15–24	1312	(80.34)*	303	(21.58)	376	(21.17)	2.29	(0.11)
	25–29	1569	(73.07)	335	(18.80)	491	(21.54)	2.34	(0.10)
	30+	798	(64.09)	162	(20.06)	277	(22.30)	2.42	(0.16)
Age at marriage									
	less than 18	853	(65.01)*	200	(22.71)^	271	(19.30)^	2.24	(0.12)
	18+	2826	(75.99)	600	(19.36)	873	(22.36)	2.38	(0.09)
Wealth quartile									
	1 (poorest)	919	(65.09)*	167	(15.73)*	293	(19.23)	2.37	(0.15)
	2	1822	(73.05)	404	(21.70)	589	(21.98)	2.38	(0.10)
	3	882	(83.45)	214	(21.29)	247	(23.95)	2.25	(0.16)
	4 (wealthiest)	56	(91.70)	15	(21.50)	15	(20.24)	1.81	(0.48)
Literacy									
	Illiterate	1605	(65.82)*	333	(20.19)	525	(20.24)	2.29	(0.11)
	Literate	2074	(80.36)	467	(20.01)	619	(22.90)	2.39	(0.09)
Spouse education									
	≤ 5th standard	759	(63.42)*	146	(17.10)^	265	(20.69)	2.52	(0.17)
	> 5th standard	2920	(76.24)	654	(20.84)	879	(21.88)	2.29	(0.08)
Caste/religion									
	Neither SC/ST nor Muslim	2197	(76.77)*	495	(20.88)	647	(21.14)	2.38	(0.10)
	SC/ST	934	(66.05)	188	(19.14)	315	(21.21)	2.32	(0.14)
	Muslim	548	(74.12)	117	(18.84)	182	(23.80)	2.28	(0.19)
Parity									
	1	1169	(84.22)*	273	(20.70)	321	(22.39)	2.49	(0.11)
	2	1028	(72.54)	222	(19.00)	327	(20.46)	2.26	(0.11)
	3+	1482	(66.99)	305	(19.00)	496	(21.84)	2.31	(0.11)
Physical IPV in past 12 months									
	No	2087	(77.58)*	454	(20.00)	615	(21.24)	2.5	(0.10)*
	Yes	1592	(68.16)	346	(20.21)	529	(22.04)	2.18	(0.09)
Sexual IPV in past 12 months									
	No	3365	(74.44)*	693	(19.20)*	1036	(21.41)	2.32	(0.08)
	Yes	314	(62.18)	107	(30.31)	108	(23.50)	2.60	(0.24)

SE denotes "Standard Error,"

*denotes p-value <0.05 for distribution

^denotes p-value <0.1 and ≥0.05 for distribution

Slightly more than 1 in 5 women (22%) reported receiving a post-natal home visit by an FLW within 24 hours of giving birth or returning home post-delivery. Women’s likelihood of receiving a postnatal visit was marginally associated with marriage at older age (p < .1) but no other demographics. Higher quality of postnatal care, measured by the number of topics covered during postnatal home visits (mean 2.34) was associated with not having experienced physical IPV in the past 12 months.

Approximately half of women sampled (46%) reported having experienced physical IPV in the last 12 months, while almost 1 in 10 (9%) reported having experienced sexual IPV in the same time frame (See Table 3). Recent physical IPV was more prevalent among older women; no other demographics were associated with past 12 months experiences of either physical or sexual IPV. The number of women who experienced both forms of IPV was n = 310. Among those that experienced physical IPV (N = 2280), 13.6% also experienced sexual IPV, while among those experiencing sexual IPV (N = 483), 64.2% also experienced physical IPV.

**Table 3:** Sample demographics by forms of IPV among women with a live birth in the past 12 months across the 25 highest-need districts of Uttar Pradesh, India (unweighted ns and weighted percentages; N = 5020).

		Total		Physical IPV (past 12 months)		Sexual IPV (past 12 months)	
Characteristic		n	%	n	%	n	%
Total		**5020**	**(100)**	**2280**	**(45.62)**	**483**	**(9.4)**
Age							
	15–24	1658	(32.96)	723	(41.63)*	165	(9.18)
	25–29	2155	(42.67)	990	(46.68)	203	(9.45)
	30+	1207	(24.37)	567	(49.16)	115	(9.65)
Age at marriage							
	less than 18	1300	(24.65)	611	(48.34)	129	(9.69)
	18+	3720	(75.40)	1669	(44.73)	354	(9.32)
Wealth quartile							
	1 (poorest)	1426	(28.66)	663	(48.64)	134	(9.39)
	2	2462	(48.11)	1106	(45.27)	243	(9.75)
	3	1072	(22.05)	480	(42.82)	98	(8.66)
	4 (wealthiest)	60	(1.17)	31	(38.47)	8	(10.10)
Literacy							
	Illiterate	2442	(48.70)	1055	(43.43)	239	(9.71)
	Literate	2578	(51.30)	1225	(47.69)	244	(9.13)
Spouse education							
	≤ 5th standard	1194	(23.09)	536	(47.19)	122	(10.48)
	> 5th standard	3826	(76.91)	1744	(45.15)	361	(9.09)
Caste/religion							
	Neither SC/ST nor Muslim	2891	(54.94)	1276	(44.68)	290	(9.93)
	SC/ST	1372	(28.46)	663	(48.71)	124	(9.35)
	Muslim	757	(16.60)	341	(43.44)	69	(7.81)
Parity							
	1	1411	(27.43)	615	(43.01)	134	(9.47)
	2	1391	(28.22)	641	(47.34)	141	(9.14)
	3+	2218	(44.35)	1024	(46.14)	208	(9.56)

*p-value <0.05 for distribution

In multivariate analyses, neither form of IPV was associated with receiving 4 or more ANC visits (See Table 4). Recent physical IPV was significantly associated with the quality of care outcomes of receipt of fewer recommended tests during ANC visits and fewer topics discussed during FLW home visits, and marginally associated with having an FLW home visit during the 3^rd^ trimester. Sexual IPV in the past 12 months was not associated receipt or quality of ANC or FLW home visits during pregnancy.

**Table 4:** Multivariate associations of physical and sexual IPV with antenatal care outcomes among women having a live birth in the past 12 months in the 25 highest-need districts of Uttar Pradesh, India (N = 5020).

		≥ 4 ANC		Number of ANC tests received		ASHA home visit during 3rd trimester		Number of topics covered in home visits during pregnancy	
Characteristic		AOR	95% CI	Beta	95% CI	AOR	95% CI	Beta	95% CI
Physical IPV in past 12 months									
	No	Ref	--	Ref	--	Ref	--	Ref	--
	Yes	1.05	(0.81–1.34)	-0.30	(-0.51–0.08)*	0.81	(0.65–1.01)^	-0.44	(-0.78–0.15)*
Sexual IPV in past 12 months									
	No	Ref	--	Ref	--	Ref	--	Ref	--
	Yes	1.08	(0.75–1.55)	-0.02	(-0.31–0.25)	1.03	(0.79–1.34)	0.18	(-0.52–0.88)

AOR: “Adjusted odds ratio,” 95% CI: "95% confidence interval,"

*denotes p-value <0.05

^denotes p-value <0.1 and ≥0.05. All models adjusted for age, age at marriage, wealth, literacy, spousal education, caste/religion, and parity.

Recent physical and sexual IPV were both significantly associated with greater odds of home delivery (See Table 5). In addition, having experienced sexual IPV in the past 12 months was associated with increased odds of a women leaving a birth facility earlier than recommended (OR = 1.87, CI = 1.36–2.58). Neither physical nor sexual IPV were associated with receipt of a post-natal FLW home visit within 24 hours, but having experienced physical IPV in the past 12 months was significantly associated with discussion of fewer recommended topics during post-natal home visits.

**Table 5:** Multivariate associations of physical and sexual IPV with delivery and postnatal care outcomes among women having a live birth in the past 12 months in the 25 highest-need districts of Uttar Pradesh, India (N = 5020).

		Institutional delivery		Left facility early		< 24 hours post natal ASHA home visit		Number of topics covered in post natal home visits	
Characteristic		AOR	95% CI	AOR	95% CI	AOR	95% CI	Beta	95% CI
Physical IPV in past 12 months									
	No	Ref	--	Ref	--	Ref	--	Ref	--
	Yes	0.65	(0.46–0.92)*	0.97	(0.77–1.21)	1.03	(0.85–1.26)	-0.26	(-0.52–0.01)*
Sexual IPV in past 12 months									
	No	Ref	--	Ref	--	Ref	--	Ref	--
	Yes	0.61	(0.44–0.83)*	1.87	(1.36–2.58)*	1.13	(0.83–1.52)	0.19	(-0.30–0.69)

AOR: “Adjusted odds ratio,” 95% CI: "95% confidence interval,"

*denotes p-value <0.05

^denotes p-value <0.1 and ≥0.05. All models adjusted for age, age at marriage, wealth, literacy, spousal education, caste/religion, and parity

## Discussion

The current findings suggest that women in high-need districts of Uttar Pradesh who experience IPV are both less likely to receive multiple forms of perinatal health care known to reduce maternal and neonatal mortality, and less likely to receive higher qualities of health service for those types of care that they do receive. Notably, for the two cases where IPV did not affect the likelihood of specific forms of care (4 or more ANC visits, immediate postnatal home visit), IPV was associated with significantly lower quality of both forms of care, indicating the need to consider both quantities and qualities of health services when attempting to understand how IPV might relate to poorer maternal and neonatal outcomes. Associations of IPV with decreased quality of perinatal care were found across all perinatal periods of care–antenatal, delivery and postnatal, indicating that effects of IPV on qualities of essential health interventions span the full continuum of care.

Approximately half (45.6%) of all women who had experienced a live birth in the year in these 25 high-need districts of Uttar Pradesh, India reported experiencing physical IPV in the past year; 1 in 10 (9.4%) reported experiencing sexual IPV within this period. Because the current sample was limited to women who had given birth in the past year, this likelihood of this violence having occurred during their last trimester of their recent pregnancy or the immediate postnatal period is high. Thus, a very large percentage of women who are meant to be served by the public health system via engagement in antenatal, delivery and postnatal care are likely to be currently experiencing IPV. The current findings that this large number of women are significantly less likely to receive a home visit from a FLW both during their third trimester and within 24 hours of delivery, and that the quality/completeness of care that they do receive is significantly lower than other women, should be of great concern and a priority of efforts to improve the reach and efficacy of the public health system in Uttar Pradesh. These findings also support previous assertions that the associations consistently observed between IPV and poor maternal and neonatal health may be, in part, explained by abused women’s reduced exposure to health service interventions known to increase the risk for morbidity [31–33].

The growing research literature regarding the public health impact of another gender-based social issue, child marriage, echoes the current findings. Women in India married under age 18 years are significantly less likely to receive antenatal care, and the neonates of such women are more likely to be in poor health, e.g., have low birthweight [34,35]. Forms of marginalization based on literacy, poverty and religion have also been found to affect receipt of care, with receiving a visit from a FLW found to be less likely to result in either four or more ANC visits or in facility delivery [26]. Similar to current findings, this pattern of effects suggests that the quality of FLW visits may be reduced for marginalized groups. Collectively, this body of research suggests that the quality and efficacy of perinatal health interventions is reduced based on IPV and other forms of gendered and non-gendered marginalization.

Research is required to understand the reasons for delivery and receipt of lower quality care. Possible hypotheses for future work to test might include abused women actively shortening the length of both FLW home visits and their stay at a birth facility due to fears of further violence. Such fear may be based on threats they have received from their husband regarding punishment for not completing household tasks, not feeding and caring for in-laws, for allowing others in the home, or spending an extended period away from home. Other possible explanations include shortening of home visits by FLWs due to their discomfort or concerns for their own safety in the homes of abused women, perhaps related to the presence or controlling behaviors the husband or other family members. FLWs may also look down upon women who are exhibiting behaviors associated with trauma, spending less time with such women, thus, providing fewer services. These and other hypotheses will require extensive qualitative research. Based on the resulting increased understanding of these dynamics, health systems must develop, implement and assess the efficacy of policies to reduce these disparities in care.

The current study also attempted to distinguish effects related to physical and those related to sexual IPV regarding perinatal health care receipt and quality. Physical IPV was associated with reduced quality of ANC, FLW visits during pregnancy and FLW visits in the postnatal period. Both physical and sexual IPV were associated with reduced likelihood of facility delivery, but physical IPV was not associated with leaving a birth facility earlier than recommended. In contrast, sexual IPV was associated with this outcome, but no other indicators of health care quality. One possible explanation for this difference might be that sexual IPV and related sexual entitlement by a husband may be accompanied by imposing greater limitations on her interactions with others that he sees as sexual, including giving birth. This may lead to demands that a woman remain home for her delivery and, that if she does leave home for delivery, that she minimize such interactions and return home quickly regardless of the risks this may pose for the woman or the neonate. A greater understanding of the different ways that experiences of physical and sexual IPV may compromise health service interactions is required in order for health systems to develop effective ways to reduce these impacts.

The current findings should be viewed in light of several limitations related to the design of the study. The prevalence of both physical and sexual IPV found in this study is approximately double that found by the nationally-representative NFHS-4 (National Family Health Survey-4; 22.6% physical IPV and 5.2% for sexual IPV), a survey conducted in the same year and via the same measures. Several reasons may explain this discrepancy. Foremost, the current sample was drawn from the “poorest performing” third of districts of Uttar Pradesh, a state reporting a higher prevalence for lifetime experiences of either physical or sexual IPV than that observed nationally (32.4% vs 29.2%). This presents a challenge to generalization of the current findings to either all of Uttar Pradesh or India as a whole. The inclusion criteria related to recent live birth skew the current sample towards younger ages, with 75% of participants in the current under age 30 compared to XX% of the national sample; younger age is associated with greater risk for IPV in this sample as well as that of the NFHS-4. However, this is also a strength of the current study in that it includes those women with recent experiences of antenatal, delivery and postnatal health services, reducing any potential recall bias relative to studies of these services among the general population of women. Another strength of the current analyses is consideration of recent experience of IPV, those in the past 12 months, rather than lifetime experience of IPV. The observed associations between IPV and receipt and quality of perinatal health care reflect experiences of IPV concurrent with the index pregnancy and/or postnatal period, and not separated by an unknown period from these events. Another notable limitation is the cross-sectional nature of the analyses, precluding conclusions regarding the temporal relationship or directionality of the observed associations. Longitudinal analyses are required to confirm the current interpretations of these associations.

## Conclusion

The findings of the current study indicate that women who experience IPV during pregnancy and/or the postnatal period are significantly less likely to receive multiple core perinatal health services, and receive significantly lower qualities of these services when they do receive them. If these findings are confirmed via longitudinal study and analyses of data from other geographic contexts, this disparity in receipt and quality of critical health interventions during pregnancy, at delivery and following delivery may represent a key mechanism underlying associations of IPV and maternal and neonatal morbidity and mortality. Identifying and addressing the barriers responsible for these inadequacies of care based on IPV, a form of violence affecting half of the women in the current sample, should be prioritized in order to improve maternal health and child survival in this region of India.

## Supporting information

S1 File Household questionnaire. Household survey used for data collection (In English and Hindi).
(PDF)
