# Supplementary material for: Associations between recent intimate partner violence and receipt and quality of perinatal health services in Uttar Pradesh
Source: PLoS One. 2020 May 14;15(5):e0232079. doi: 10.1371/journal.pone.0232079 (PMC7224484; doi:10.1371/journal.pone.0232079)
Supplement: S1 File — Household survey used for data collection (In English and Hindi). (PDF) [file pone.0232079.s001.pdf]

MLE for UP – TSU
Midline Study – 2016
Household Questionnaire

उत्तर प्रदेश – तकनीकी सहायता इकाई (टीएसयू)
के लिए एमएलई
मिडलाईन अध्ययन 2016
परिवार प्रश्नावली

I.	Village Information xkao dh tkudkjh						
		District<br>जिला	Block<br>ब्लोक	Village<br>गाँव	ASHA Area<br>आशा क्षेत्र	Structure No.<br>भवन संख्या	HH No.<br>घर संख्या	UID<br>यू.आई.डी.
A.	Name<br>नाम							
B.	Code<br>कोड							
	Total eligible women in the household<br>परिवार में कुल चयनित महिलाएं			Woman ID<br>महिला की संख्या				

II.	Respondent Information mÙkjnkrk dh tkudkjh	
A.	Name of the head of the household घर के मुखिया का नाम	
B.	Address पता	
C.	Important Landmark nearest to the HH<br>घर के पास का कोई महत्वपुर्ण चिन्ह	
D.	Phone Number फोन नंबर	☐☐☐☐☐☐☐☐☐☐
E.	Respondent category<br>उत्तरदाता का वर्ग	1 = MOTHER OF 0-5 MONTH CHILD 0–5 महिने की आयु वाले बच्चे की माँ<br>2 = MOTHER OF 6-11 MONTH CHILD 6–11 महिने की आयु वाले बच्चे की माँ<br>3 = MOTHER OF 12-23 MONTH CHILD 12–23 महिने की आयु वाले बच्चे की माँ<br>4 = WOMAN WHO HAD ABORTION/MISCARRIAGE महिला जिसने गर्भपात कराया है/बच्चा गिर गया है<br>5 = ADOLOSCENT GIRL 10-19 YEARS OLD 10–19 साल की किशोरी<br>7 = WOMEN WHO HAD STILL BIRTH/DEATH OF A CHILD महिला जिसने मृत बच्चे को जन्म दिया/बच्चे की मृत्यु हो गयी<br>8= CURRENTLY PREGNANT WOMEN 15-49 YEARS OLD वर्तमानं में 15-49 साल की उम्र की शादी शुदा महिला
F.	Name of the Woman महिला का नाम	
G.	Age of the woman महिला की आयु	☐☐
H.	**ASK ONLY IF CATEGORY CHOSEN FOR QUESTION E=1, 2, 3, 7**<br><br>Name of the Child बच्चे का नाम	
I.	**ASK ONLY IF CATEGORY CHOSEN FOR QUESTION E=1, 2, 3, 7**<br><br>Date of Birth of the Child बच्चे की जन्म तिथि	☐☐/☐☐/☐☐☐☐
J.	**ASK ONLY IF CATEGORY CHOSEN FOR QUESTION E=1, 2, 3, 7**<br><br>Age of the Child (in completed months) बच्चे की आयु (पूर्ण महिनो मे)	☐☐

8	**Survey Information *(To be filled by the enumerator)*** शाक्षात्कार की जानकारी (शाक्षात्कारकर्ता द्वारा भरा जायगा)	
A.	Name of the respondent उत्तरदाता का नाम<br>(Auto coded from F)	
B.	**First Visit** पहली मुलाकात	
C.	Interviewer name शाक्षात्कारकर्ता का नाम	
D.	Interviewer code शाक्षात्कारकर्ता का कोड	☐☐
E.	Date of the interview शाक्षात्कार की तिथि	☐☐/☐☐/☐☐☐☐
F.	Result of the interview शाक्षात्कार का नतीजा	1 = INTERVIEW COMPLETED पुर्ण रुप से भरा गया<br>2 = VISIT RESCHEDULED दुबारा बुलाया गया<br>3 = INTERVIEW INCOMPLETE शाक्षातकार पुर्ण रुप से नहीं भरा गया<br>4 = REFUSED मना कर दिया गया<br>5 = HOUSEHOLD NOT LOCATED घर नहीं मिला

		6 = RESPONDENT IS OUT OF STATION FOR EXTENDED PERIOD उत्तरदाता लम्बे समय के लिए बाहर गए हैं<br>7 = RESPONDENT IS TEMPORARILY AWAY उत्तरदाता कुछ समय के लिए बाहर गए हैं<br>88 = OTHER (SPECIFY) अन्य (स्पष्ट करें)	
9	**Second Visit** दूसरी मुलाकात		
A.	Interviewer name शाक्षात्कारकर्ता का नाम		
B.	Interviewer code शाक्षात्कारकर्ता का कोड		□□
C.	Date of the interview शाक्षात्कार की तिथि		□□/□□/□□□□
D.	Result of the interview शाक्षात्कार का नतीजा		□
1	**Third Visit** तीसरी मुलाकात		
A.	Interviewer name शाक्षात्कारकर्ता का नाम		
B.	Interviewer code शाक्षात्कारकर्ता का कोड		
C.	Date of the interview शाक्षात्कार की तिथि		
D.	Result of the interview शाक्षात्कार का नतीजा		
E.	**RECORD TIME WHEN YOU START THE INTERVIEW.** शाक्षात्कारकर्ता शाक्षात्कार शुरू करने का समय दर्ज करें		□□ HOUR घंटे<br>□□ MINUTES मिनट

11 INTRODUCTION

Hello, My name is ___________________. I am a representative of the Sambodhi Research and Communications Pvt. Ltd. You are being asked to take part in a big survey designed to understand and improve healthcare being delivered in Uttar Pradesh state. We represent a group of researchers studying healthcare practices in partnership with the state government of Uttar Pradesh. A number of health programs will be started over the next few years in Uttar Pradesh to improve maternal and child health. The main purpose of our survey is to document the effects of these programs in improving maternal and child health.

नमस्कार। मेरा नाम ....है। मैं संबोधि रिसर्च एन्ड कम्युनिकेशन्स की प्रतिनिधि हूँ। मैं आपसे ऊत्तर प्रदेश में प्रदान की जाने वाली स्वास्थ्य सेवाओं को समझने और बेहतर बनाने के लिए संरचित किये जा रहे एक बड़े सर्वेक्षण में भाग लेने के लिए कह रही हूँ। मैं शोधकर्ताओं के एक ऐसे समूह का प्रतिनिधित्व करती हूँ जो ऊत्तर प्रदेश सरकार के साथ मिलकर स्वास्थ्य सेवाओं का अध्यन कर रही है। ऊत्तर प्रदेश में मातृ एवं शिशु स्वास्थ्य को बेहतर बनाने के लिये अगले कुछ वर्षों में कई स्वास्थ्य कार्यक्रम प्रारम्भ किये जायेंगे। इस सर्वेक्षण का मुख्य उद्देश्य माताओं और बच्चों के स्वास्थ्य में सुधार लाने वाले इस कार्यक्रम के प्रभाव का अनुमान लगाना है।

We want to know and understand the current conditions of maternal and child health and the services available to women and children in your community. We are contacting about 5,000 households in Uttar Pradesh for this survey now. There is another round of surveys planned over the next years to try understand the impact of the programs.

हम समुदायों में माताओं और शिशुओं के स्वास्थ्य की वर्तमान स्थिति और मातृ एवं शिशु स्वास्थ्य के लिये उपलब्ध स्वास्थ्य सेवाओं को जानना और समझना चाहते हैं। हम पूरे ऊत्तर प्रदेश से करीब 5,000 परिवारों में सर्वेक्षण करेंगे। इस सर्वेक्षण के बाद कार्यक्रम से होने वाले प्रभाव को देखने के लिए अगले वर्ष सर्वेक्षण का 1 और दौर होगा।

We will ask questions to women who had pregnancy termination in the last two years. We would request them to answer a few questions about general household information, other family members in household, their experiences and level of satisfaction with health care related to pregnancy, delivery and newborn health, and some basic information about your household assets. The households are selected randomly to participate in the survey. The information collected from about 5,000 households from across the state will be combined in a report and research papers to discuss with the government of Uttar Pradesh and other health professionals.

हम उन महिलाओं से प्रश्न पूछेंगे जिनके पिछले दो वर्ष में गर्भ समापति हुई हो। हम उन महिलाओं से उनके परिवार की सामान्य जानकारी, घर के अन्य सदस्य, गर्भावस्था, प्रसव और नवजात शिशुओं के स्वास्थ्य संबंधित उपलब्ध सेवाओं के अनुभव और सन्तुष्टी के स्तर और पारिवारिक सम्पति के बारे में जानकारी देने का अनुरोध करेंगे। परिवारों का चयन रैनडम विधि से किया गया है। पूरे राज्य से करीब 5,000 परिवारों से एकत्र की गई सूचना को एकत्रित करके एक रिपोर्ट और कुछ रिसर्च पेपर बनाए जाएंगे जिनकी ऊत्तर प्रदेश सरकार और अन्य स्वास्थ्य सेवा में काम करने वाले व्यक्तियों के साथ मिलकर चर्चा की जायेगी।

All the answers that you/others provide will be kept private — only survey researchers will have access to this information. You may choose not to participate in the interview. You can stop the interview at any time or ask me to clarify any question. You may also choose to withdraw from the survey at any time.

आपके या अन्य उत्तरदाताओं के द्वारा दी गई जानकारी गोपनीय रखी जायेगी – यह जानकारी केवल शोधकर्ताओं तक ही सीमित रहेगी। अगर आप चाहें तो आप साक्षात्कार में भाग लेने से मना कर सकती हैं। आप साक्षात्कार किसी भी समय रोक सकती हैं या किसी प्रश्न को समझाने के लिये मुझे कह सकती हैं। आप किसी भी समय इस साक्षात्कार में भाग लेने से मना कर सकती हैं।

Do you have any questions for me now? क्या आप मुझे कोई प्रश्न पूछना चाहती हैं?

ANSWER QUESTIONS AS COMPLETELY AS POSSIBLE AND PROCEED. जहाँ तक सम्भव प्रश्नों का उत्तर दें।

I have discussed with ___________________ the above procedures, explicitly pointing out potential risks or discomforts. I have asked whether any questions remain and have answered these questions to the best of my ability. The subject verbally agreed to participate in this study.

मैंने उपरोक्त विवरण को .... को पूरी तरह से बताया है। मैंने उनसे पूछा कि क्या उनके और कोई प्रश्न हैं और अपनी पूरी योग्यता के साथ उन सवालों का जवाब दिया है। उत्तरदाता ने मौखिक रूप से इस अध्यन में भाग लेने के लिए सहमती दी है।

A. RESPONSE

1 = YES → **Very good →** START INTERVIEW.
हाँ cgr vPNk शाक्षात्कार शुरू करें।

0 = NO → **Thank you for your time →** DETERMINE IF ANOTHER TIME WOULD WORK. RECORD RESULT CODE AND APPOINTMENT ON COVERSHEET.
नहीं vkidk le; dk fy, /kU;okn पता करें कि क्या और समय सही रहेगा। नतीजे का कोड कवर शीट पर लिखें।

A. HOUSEHOLD ROSTER (RC1/2/3/4/5/6/7/8)

A1 to A13 of this section will be asked to all the categories: mother of 0-5 month, mother of 6-11 month, mother of 12-23 month, woman who had an abortion/miscarriage, adolescent girl of 10-19 years, currently married women of 15-49 years, women who had still birth/death of a child, currently pregnant women of 15-49 years

***AID**: A household member is "a person or group of people who usually live and eat from the same kitchen". Any visitor who has been living in that house and eating from the same kitchen since a while will also be included as a member of the household*
सहायताः घर एक या एक से ज्यादा लोगों का ऐसा समुह है जो आम तौर पर एक साथ रेहते हैं और एक चुल्हे का बना खाते हों।

A1.	A2.	A3.	A4.	A5.	**A6.**
Line Number<br>लाइन नम्बर	Please tell me the names of all the persons who usually live in your household, starting with the head of the household. Be sure to include yourself as well as visitors.<br>अपने घर मे सामान्य तौर पर रहने वाले सभी व्यक्तियों के नाम बताइये और शुरूआत परिवार के मुखिया से कीजिये। अपने आप को और मेहमानों को भी शामिल कीजिये।	What is the relationship of [NAME] to the head of household?<br>घर के मुखिया से (नाम) का क्या संबन्ध है?<br><br>**SEE CODES BELOW**<br>uhp dkM n[k	What is the sex of [NAME]?<br>(नाम) का लिंग क्या है?<br><br>1 = MALE पूरूष<br>2 = FEMALE महिला<br>3 = TRANSGENDER हिजड़ा	How old is [NAME]?<br>(नाम) कितने साल का है?<br><br>**ENTER IN COMPLETED YEARS**<br>i.k o"kk e fy[k<br><br>99 = DO NOT KNOW पता नहीं	ASK ONLY IF AGE >9 YEARS<br>यदि आयु 9 साल से अधिक है तभी पूछें<br>What is [NAME's] marital status?<br>(नाम) की वैवाहिक स्थिति क्या है?<br>1 = CURRENTLY MARRIED वर्तमान मे शादीशुदा<br>2 = MARRIED, GAUNA NOT PERFORMED शादीशुदा लेकिन गौना नहीं हुआ<br>3 = WIDOWED विधवा<br>4 = DIVORCED तलाकशुदा<br>5 = SEPARATED अलग–अलग<br>6 = DESERTED त्यागा गया<br>7 = NEVER MARRIED कभी शादी नहीं हुई
01					
02					

Codes for Relationship

1 = HEAD OF THE HOUSEHOLD परिवार का मुखिया	10 = NIECE/NEPHEW भांजा/भांजी/भतिजा/भतीजी
2 = SPOUSE पति/पत्नी	11 = GRAND PARENT/GRAND PARENT-IN-LAW दादा/दादी/नाना/नानी
3 = SON/DAUGHTER बेटा/बेटी	12 = OTHER RELATIVE अन्य रिश्तेदार
4 = SON/DAUGHTER IN LAW दामाद/बहु	13 = ADOPTED/FOSTER/STEPCHILD गोद लिया/सौतेला बच्चा
5 = GRANDCHILD पोता/पोती	14 = DOMESTIC SERVANT नौकर/नौकरानी
6 = FATHER/MOTHER माता/पिता	15 = OTHER NOT RELATED अन्य संबंधित नहीं
7 = FATHER/MOTHER IN LAW सास/ससुर	16 = VISITOR/GUEST मेहमान
8 = BROTHER/SISTER भाई/बहन	88 = OTHER (SPECIFY) अन्य (स्पष्ट करें)
9 = BROTHER/SISTER IN LAW साला/साली/भाभी/जेठ/जेठानी	99 = NOT STATED नहीं बताया

A7.	Name of the target woman चयनित महिला का नाम	
A8.	Line Number of target woman चयनित महिला का लाइन नंबर	☐☐
A9.	Do you know how to read and write?<br>क्या आप पढ लिख सकती हैं?	1 = YES, READ AND WRITE हाँ, पढ़ना और लिखना दोनो<br>2 = YES, READ ONLY हाँ, केवल पढ़ना<br>3 = CAN SIGN ONLY केवल दस्तखत करना<br>4 = CANT READ AND WRITE पढ़ और लिख नहीं सकते
A10.	Have you ever attended school?<br>क्या आप कभी स्कूल गयी हैं?	1 = YES हाँ<br>0 = NO नहीं → **GO TO A12**

A11.	What is the highest standard or class you completed?<br>आप कितनी कक्षा तक पढ़ी हैं?	☐☐ STANDARD कक्षा<br>98 = MADARSA EDUCATED मदरसे मे पढ़ाई
A12.	Apart from housework, have you worked in any job for which you were paid in cash or kind in the last 12 months?<br>घर के काम के अतिरिक्त, क्या पिछले 12 महिनों मे आपने कोई काम किया था जिससे आपको पैसा या कुछ और मिला था?	1 = YES हाँ<br>0 = NO नहीं → **GO TO A14a**

A13.	What kinds of work did/do you do?<br>आपने किस प्रकार का काम किया था/करती हैं?<br><br>**PROBE: ANY OTHERS?**<br>çkc % dN vkj\<br><br>**RECORD ALL MENTIONED**<br>lHkh mÙkj fy[kA	1 = FARMER (CROPS) किसान<br>2 = AGRICULTURAL DAY LABOR दैनिक खेती मजदूर<br>3 = HERDING ANIMALS जानवर चराना<br>4 = NON AGRICULTURAL DAY LABOR दैनिक गैर खेती मजदूर<br>5 = MAID SERVANT नौकर<br>6 = COOK खाना बनाना<br>7 = CHILD CARE PROVIDER बच्चों की देखभाल करना<br>8 = SERVICE/SALARIED WORKER सर्विस/वेतन भोगी<br>9 = SMALL/COTTAGE INDUSTRY छोटा/कुटीर उद्योग<br>10 = PETTY BUSINESS/SHOP छोटा व्यापार/दुकान<br>11 = BUSINESS/TRADER व्यवसाय/व्यापार<br>12 = OTHER SELF-EMPLOYMENT अन्य खुद का रोजगार<br>13 = SKILLED WORKER कुशल कारीगर<br>88 = OTHER (SPECIFY) अन्य (स्पष्ट करें)
Question A14-A23 will not be asked to Category 5: Adolescent girls of 10-19 years of age		
A14.	**(RC1,2,3,4,6,7,8)**How old were you when you got married for the first time? जब आपकी पहली बार शादी हुई थी उस समय आपकी आयु कितनी थी?<br>**PROBE : ASK FOR FIRST MARRIAGE IF MARRIED MORE THAN ONCE.**<br>çkc % ;fn efgyk dh 'kkfn ,d l vf/kd ckj 'kknh gbZ g] mldh igyh 'kknh d ckj e iN\	☐☐ IN COMPLETED YEARS पूर्ण वर्षो मे
A15.	**(RC1,2,3,4,5,6,7,8)**Did your husband ever attend school?<br>क्या आपके पति कभी स्कूल गये हैं?	1 = YES हाँ<br>0 = NO नहीं → **GO TO A17**<br>9 = NOT APPLICABLE लागू नहीं → **GO TO A17**
A16.	**(RC1,2,3,4,5,6,7,8)**What is the highest standard or class your husband completed?<br>आपके पति कितनी कक्षा तक पढ़े हैं?	☐☐ STANDARD कक्षा<br>98 = MADARSA EDUCATED मदरसे मे पढ़ाई<br>99 = DO NOT KNOW पता नहीं
A17.	**(RC1,2,3,4,5,6,7,8)**In the past 12 months, has your husband been employed?<br>क्या पिछले 12 महिनों मे आपके पति ने कोई काम किया है?	1 = YES हाँ<br>0 = NO नहीं → **GO TO A18a**
A18.	**(RC1,2,3,4,5,6,7,8)**What is your husband's occupation?<br>आपके पति क्या काम करते हैं?<br><br>**PROBE: IF NOT WORKING CURRENTLY, ASK WHAT DID HE DO EARLIER. ANY OTHERS?**<br>çkc % ;fn efgyk d ifr vHkh dkbZ dke ugh dj jg g] iN og igy D;k dke djr FkA dN vkj\<br><br>**RECORD ALL MENTIONED**<br>lHkh mÙkj fy[kA	1 = FARMER (CROPS) किसान<br>2 = AGRICULTURAL DAY LABOR दैनिक खेती मजदूर<br>3 = NON AGRICULTURAL DAY LABOR दैनिक गैर खेती मजदूर<br>4 = SERVICE/SALARIED WORKER सर्विस/वेतन भोगी<br>5 = SMALL/COTTAGE INDUSTRY छोटा/कुटीर उद्योग<br>6 = BUSINESS/TRADERS व्यवसाय/व्यापार<br>7 = RICKSHAW/VAN PULLING रिक्शा/गाड़ी खीचना<br>8 = SERVANT नौकर<br>9 = COOK खाना बनाना<br>10 = OTHER SELF-EMPLOYMENT अन्य खुद का रोजगार<br>11 = PETTY BUSINESS/SHOP छोटा व्यापार/दुकान

		14 = JOBLESS बेरोजगार<br>13 = SKILLED WORKER कुशल कारीगर<br>14 = TOO OLD TO WORK बहुत बूढें हैं काम नहीं कर सकते<br>15 = PENSION पेंशन भोगी<br>88 = OTHER (SPECIFY) अन्य (स्पष्ट करें)<br>99 DO NOT KNOW पता नहीं
**A18 A**	**ASK IF CODED 1 in A8A, ELSE GO TO B1**<br>**(RC1,2,3,4,6,7,8)Is your husband currently residing with you in this house or does he live elsewhere?**<br>क्या आपका पति वर्तमान में आपके साथ रह रहे है के वोह कहीं और रहते है?	1= RESIDING WITH THE WOMAN IN THE SAME HOUSE<br>पत्नी के साथ एक ही घर में रह रहे है<br>2=HUSBAND LIVES IN A SEPARATE HOUSE<br>पति अलग घर में रहते है<br>3= MIGRATED WITHIN DISTRICT<br>इसी शहर में पलायन किया हो----→**SKIP TO A20**<br>4= MIGRATED TO ANOTHER DISTRICT/STATE<br>दूसरे शहर में पलायन किया हो----→**SKIP TO A20**<br>9= NOT APPLICABLE<br>लागू नहीं
A19.	**(RC1,2,3,4,6,7,8)**Has your husband migrated for work outside the village in the past 12 months?<br>क्या पिछले 12 महिनों में आपके पति ने काम के लिए गाँव से बाहर पलायन किया है?	1 = YES हाँ<br>0 = NO नहीं → **GO TO SECTION B**
A20.	**(RC1,2,3,4,6,7,8)**How many times did your husband migrate for work in the past 12 months?<br>पिछले 12 महिनों में आपके पति ने काम के लिए गाँव से बाहर कितनी बार पलायन किया है?	NO. OF TIMES ☐☐<br>पलायन की संख्या
**A23**	**(RC1,2,3,4,6,7,8)**What is the reason for migration for your husband?<br>आपके पति ने किस कारण से पलायन किया था?	1 = BETTER AVAILABILITY OF LOCAL LABOUR WORK बेहतर स्थानिय मजदूरी (काम)<br>2 = BETTER LOCAL WAGE बेहतर स्थानिय मजदूरी (पैसा)<br>3 = TO GET TIMELY PAYMENT OF WAGES समय पर पैसा मिलता है<br>4 = TO AVOID LONG WORKING HOURS ज्यादा देर तक काम करने से बचने के लिए<br>88 = OTHERS (SPECIFY) अन्य (स्पष्ट करें)

B. BIRTH HISTORY (RC1/2/3/4/6/7/8)

This section will be asked to all the categories except adolescent girls of age 10-19 years.

Read out to the respondent: Now, I am going to ask you a few questions about your children.

mÙkjnkrk dks i<dj lquk;sa vc eSa vkils vkids cPpksa ds ckjs esa dqN ç'u iNwxhA

B1.	How many children have you given birth to till now? Please include all children that were born, whether still alive or not. Also include your children who do not currently live with you.<br>आपने अभी तक कितने बच्चों को जन्म दिया है? कृप्या उन सभी बच्चों को शामिल करें जीवित जन्मे थे – चाहे वह अभी जीवित हैं या नहीं। उन बच्चों को भी शामिल करें जो वर्तमान मे आपके साथ नहीं रहते हैं।	☐☐ TOTAL NUMBER OF CHILDREN BORN ALIVE<br>कुल जीवित जन्मे बच्चों की संख्या<br>**IF 0 BIRTHS, THEN GO TO B10**
**B1A**	**IF 00 in B1, skip to B10**<br>How old were you when you had your first child?<br>जब आपका पहला बच्चा पैदा हुआ था तब आपकी उम्र कितनी थी?	☐☐ AGE IN YEARS

Read out to the respondent: Now I would like to record the names of all the children you've given birth to, whether still alive or not, starting with the first one you had. RECORD NAMES OF ALL CHILDREN GIVEN BIRTH TO FIRST **(B2)** THEN ASK **B3 – B10** FOR EACH CHILD.
उत्तरदाता को पढ़कर सुनायें: अब मै आपके सबसे पहले बच्चे से शुरु करते हुए उन सभी बच्चों के नाम लिखुंगी जिनको आपने जन्म दिया है, चाहे वह अभी जीवित हैं या नहीं। पहले सभी जन्मे बच्चों के नाम लिखें। उसके बाद हर बच्चे के लिए **B3 – B10** पूछें।

B2.	**B2a.**	B3.	B4.	B5.	B6.	B8.	B9.
What name was given to your (first/next) baby?<br>आपने (पहले/अगले)	CHECK BOX OF FOCAL CHILD ONLY<br>केवल चयनित	Was this a single/multiple birth?<br>क्या बच्चा अकेला पैदा हुआ था या एक से अधिक बच्चों का जन्म हुआ था?	What is the sex of [NAME]?<br>(नाम) का लिंग क्या है?<br>1 = MALE<br>पूरूश	Is [NAME] still alive?<br>क्या (नाम) अभी जीवित है?<br>1 = YES<br>हाँ	**IF DEAD:** How old was (NAME) when he/she died?<br>**IF '1 YR,' PROBE:**	What is/would have been the age of [NAME] in completed years?<br>(नाम) की आयु क्या है/होती?<br>**RECORD IN COMPLETED YEARS.**<br>आयु पूर्ण वर्षों में लिखें।	**IF [NAME] IS AGED LESS THAN 2 YEAR, ASK:** What is/would have been

बच्चे को क्या नाम दिया गया?	बच्चे को चिंहित करें	1 = SINGLE एकेला<br>2 = MULTIPLE एक से अधिक	2 = FEMALE महिला<br>3 = TRANSGENDER हिजड़ा	0 = NO नहीं<br>9 = DO NOT KNOW पता नहीं	How many months old was (NAME)? ;fn e`r g% मृत्यु के समय उसकी आयु क्या थी? ;fn mÙkj ,d o"k g% rk çkc dj% (नाम) कितने महीने का था?<br>1 = DAYS दिन<br>2 = MONTHS महीना<br>3 = YEARS साल		the age of [NAME] in completed months? ;fn ¼uke½ dh vk;q 2 o"k ls de g] iN% (नाम) की आयु पूर्ण महिनों में क्या है/होती ?
		☐	☐			☐☐	☐☐

B10.	Have you ever given birth to a child who was born dead? By this we mean a child who did not show any signs of life such as crying, breathing, or movement?<br>क्या आपने कभी ऐसे किसी बच्चे को जन्म दिया है जो मृत पैदा हुआ था? यानी ऐसा बच्चा जिसने जिन्दा होने का कोई लक्षण नहीं दिखाया, जैसे रोना, साँस लेना या कोई हरकत करना?	1 = YES हाँ<br>0 = NO नहीं → **GO TO B12**
B11.	If yes, how many?<br>यदि हाँ, तो कितनी बार?	☐ NUMBER OF STILL BIRTHS<br>मृत जन्मों की संख्या
B12.	Have you ever had an abortion or miscarriage?<br>क्या कभी आपने गर्भपात कराया था या आपका गर्भ गिराये गया था?	1 = YES हाँ<br>0 = NO नहीं → **GO TO B14**
B13.	If yes, how many?<br>यदि हाँ, तो कितनी बार?	☐ NUMBER OF ABORTIONS गर्भपात कितनी बार<br>☐ NUMBER OF MISCARRIAGE गर्भ गिरना कितनी बार
B14.	Are you pregnant now?<br>क्या आप अभी गर्भवती हैं?	1 = YES हाँ<br>0 = NO नहीं → **GO TO B17**<br>99 = UNSURE अनिश्चित → **GO TO B17**
B15.	How many months pregnant are you now?<br>अभी आपको कितने महीने का गर्भ है?	☐ COMPLETED MONTHS पूर्ण महीने
B16.	After the child you are expecting now, would you or your husband like to have another child, or would you prefer not to have any more children?<br>अभी आपको जो बच्चा होने जा रहा है उसके बाद क्या आप या आपके पति एक/एक और बच्चे चाहते हैं या आप कोई और बच्चा नहीं चाहते हैं?	1 = HAVE A/ANOTHER CHILD बच्चा/एक और बच्चा चाहते हैं → **GO TO B18**<br>2 = PREFER NO MORE CHILDREN कोई और बच्चा नहीं चाहते → **GO TO B19**<br>3 = SAYS SHE CAN NOT GET PREGNANT कहती हैं कि वह गर्भवती नहीं हो सकती → **GO TO B19**<br>88 = UNDECIDED/DO NOT KNOW अनिश्चित/पता नहीं → **GO TO B19**
B17.	**IF NOT PREGNANT ASK:**<br>Would you or your husband like to have a/another child or would you prefer not to have any more children?<br>क्या आप या आपके पति, बच्चा/एक और बच्चा चाहती हैं या आप कोई और बच्चा नहीं चाहते हैं?	1 = HAVE A/ANOTHER CHILD बच्चा/एक और बच्चा चाहते हैं<br>2 = PREFER NO MORE CHILDREN कोई और बच्चा नहीं चाहते → **GO TO B19**<br>3 = SAYS SHE CAN NOT GET PREGNANT कहती हैं कि वह गर्भवती नहीं हो सकते → **GO TO B19**<br>88 = UNDECIDED/DO NOT KNOW अनिश्चित/पता नहीं → **GO TO B19**
B18.	When would you like to have your next child?<br>आप अगला बच्चा कब चाहती हैं?	☐ ☐☐<br>1 = MONTHS महीने<br>2 = YEARS वर्ष<br>76= NOT YET PLANNED अभी नहीं सोचा<br>77 = AS SOON AS POSSIBLE जितनी जल्दी संभव हो<br>79 = WHENEVER GOD DECIDES जब भी भगवान चाहे

		80 = CANNOT GET PREGNANT<br>गर्भवती नहीं हो सकती
B19.	At the time you became pregnant (current or last), did you want to become pregnant then, did you want to wait until later, or did you not want to have any/any more children at all?<br>जब आप (इस बार/अखरी बार) गर्भवती हुई थी, क्या आप उस समय गर्भवती होना चाहती थीं, कुछ समय बाद गर्भवती होना चाहती थीं या आप कोई और बच्चा/बच्चे नहीं चाहती थीं?	1 = THEN तब<br>2 = LATER बाद में<br>3 = NOT AT ALL बिल्कुल नहीं
**B19a**	SKIP IF **0 BIRTHS FOR B1**<br>HAS YOUR MENSTRUAL PERIOD RETURNED SINCE THE BIRTH OF YOUR YOUNGEST CHILD?<br>आपके सबसे छोटे बच्चे के जन्म के बाद से क्या आपको मासिक धर्म हुआ है?	YES हाँ .... 1<br>NO नहीं .... 0 →<br>**GO TO B19C**
**B19 b**	When did your last menstrual period start?<br>आपका पिछला मासिक धर्म कब शुरू हुआ था?<br>*3*<br>*33RECORD 'YEARS AGO' ONLY IF LAST MENSTRUAL PERIOD STARTED ONE OR MORE YEARS AGO. IF 12 MONTHS OR MORE THE ANSWER MUST BE RECORDED IN YEARS.*<br>*(date if given)*<br>अगर पिछला मासिक धर्म एक या उससे अधिक साल पहले हुआ था तभी "सालों" में कोड करें। अगर १२ महीने या उससे ज्यादा है तो उत्तर "सालों" में दर्ज करें।	DAYS AGO दिन पहले .... 1<br>WEEKS AGO सप्ताह पहले .... 2<br>MONTHS AGO महीने पहले .... 3<br>YEARS AGO साल पहले .... 4<br>IN MENOPAUSE/HAS HAD HYSTERECTOMY मासिक धर्म नहीं होता है गर्भाशय को सर्जरी / के द्वारा हटा दिया गया है.................... 994<br>BEFORE LAST BIRTH पिछले जन्म के पहले....995<br>NEVER MENSTRUATED<br>कभी मासिक धर्म नहीं हुआ................. 996
Now I would like to know from you if you are aware of anaemia<br>अब मैं आपसे जानना चाहूंगी के आपको अनेमिया के बारें में कितनी जानकारी है		
**B19c**	Are you aware about what anaemia is?<br>क्या आपको अनेमिया/खून की कमी के बारें में पता है, जो आयरन की कमी की वजह से होता है?	1 = YES हाँ<br>0 = NO नहीं **SKIP TO B19e**
**B19 d**	**ASK IF YES FOR B19c**<br>What according to you causes anaemia?<br>आपके हिसाब से अनेमिया किस कारन से होता है?<br>**MULTIPLE CHOICE**	1=Not consuming Iron rich diet<br>आयरन युक्त खाना न खाने की वजह से<br>2=Not taking Iron supplementation during pregnancy<br>गर्भवस्था के दौरान आयरन की टेबलेट या आयरन की सिरप न लेने की वजह से<br>3=No interval between subsequent pregnancies<br>बच्चो के बीच में अंतर न होने की वजह से<br>4=Increased blood loss during periods<br>माहवारी में ज्यादा खून बहने से<br>5=Due to hook worm infestation<br>पेट में कीदें लग जाने पर<br>88=Others<br>अन्य<br>99= DO NOT KNOW<br>नहीं पता
**B19 d1**	What is the treatment for anaemia?<br>अनेमिया/खून की कमी का इलाज क्या है?<br>**MULTIPLE CHOICE**	1= CONSUMPTION OF IRON SUPPLEMENTS/IFA SYRUP AND TABLETS<br>आयरन की गोलिया या सिरप लेना<br>2= CONSUMPTION OF IRON RICH FOOD

		आयरन युक्त आहार लेना<br>88=OTHERS<br>अन्य<br>99=DO NOT KNOW<br>पता नहीं
**B19e**	What are the some of the food items which are rich in iron?<br><br>कुछ ऐसे खाने की चीज़ें बताईये जिनमें आयरन होता है?<br><br>**MULTIPLE CHOICE**	1=Dates/dry fruits<br>मेवे, खजूर, बादाम, मुमफली इत्यादि<br>2=Chicken/Mutton<br>मॉस- चिकन, मटन<br>3=Green leafy vegetables<br>हरी पट्टी वाली सब्जियां<br>4=Eggs<br>अन्दा<br>5=Milk<br>दूध<br>6=Cereals<br>अनाज<br>88=Others<br><br>99= DO NOT KNOW

**C. ABORTION/MISCARRIAGE (RC4)**<br>This section will only be asked to Category 4:Women who had an abortion or miscarriage in the last 1 year<br>***Read out to the respondent: Now, I would like to ask you some questions about when you were pregnant with the child for which you had an abortion/miscarriage.***<br>*mÙkjnkrk dk i<dj lquk;s vc e vki l ml le; d ckj e dN ç'u iNxh tc vki xHkorh Fkh* ***उस बच्चे के साथ जो खराब हो गया था।***		
C0.	Did the woman have a miscarriage or abortion?<br>क्या महिला का कभी बच्चा खराब हो गया हो या गर्भपात कराया हो?<br>(Auto coded)	1= Miscarriage बच्चा खराब हो गया<br>2= Abortion गर्भपात कराया
C0a	How many months pregnant were you when you had the abortion/miscarriage?<br>आप कितने महीने गर्भवती थी जब आपका बच्चा खराब हो गया हो या गर्भपात कराया हो?	☐ NO. OF MONTHS
C0a1	**ASK ONLY IF CODED 2 FOR C0**<br>Was the abortion surgical or did you take any medicine?<br>क्या आपका गर्भपात ऑपरेशन से हुआ था या आपने दवाई ली थी गर्भपात के लिए?	1= surgical<br>ऑपरेशन से/ सर्जरी से<br>2= medicine<br>दवाई ली थी, ऑपरेशन नहीं किया था
C0a2	**ASK ONLY IF CODED 2 FOR C0a1**<br>What was the procedure used?<br>कौन सी प्रणाली का प्रयोग हुआ था?<br>ASK FOR THE MEDICAL RECORD IF AVAILABLE AND NOTE	1= D&C(Dilation and Curettage)<br>2=Vacuum Aspiration<br>99=Do not know
C0b	Did you register this pregnancy with ASHA or the ANM?<br>क्या यह गर्भावस्था ऐ एन एम् / आशा के साथ रजिस्टर करवाया गया था?	1 = YES हाँ<br>0= NO नहीं → **GO TO C1**
C0c	In which month of your pregnancy did you register with ASHA or the ANM?<br>गर्भवती होने के कौनसे महीने में आपने ऐ एन एम् /आशा के साथ गर्भावस्था का रजिस्टर करवाया था?	☐ PREGNANCY MONTH<br>गर्भावस्था का महिना
C1.	During your pregnancy, did you go to see anyone for antenatal checkup?<br>जब आप गर्भवती थी तब क्या प्रसव–पूर्व जाँच के लिए आप किसी के पास गयी थी?	1= YES हाँ<br>0= NO नहीं

C2.	During your pregnancy, did anyone come to you for antenatal checkup?<br>जब आप गर्भवती थी तब क्या प्रसव–पूर्व जाँच के लिए कोई आपके पास आया था?	1= YES हाँ<br>0= NO नहीं
C2a	**IF C1 = 0 & C2 = 0 SKIP TO C5A**<br>Where all did you receive antenatal checkup during this pregnancy?<br>जब आप गर्भवती थी तब कहाँ कहाँ प्रसव–पूर्व जाँच करवाया गया था?<br><br>**PROBE : ANY OTHERS?**<br>çkc ¼ dN vkj\<br><br>**RECORD ALL MENTIONED**<br>lHkh mÙkj fy[kA	1 = YOUR HOME अपना घर<br>2 = PARENT'S HOME माता–पिता का घर<br>3 = GOVERNMENT/MUNICIPAL HOSPITAL सरकारी/नगर निगम हस्पताल<br>4 = GOVERNMENT DISPENSARY सरकारी डिस्पेंसरी<br>5 = UHC/UHP/UFWC यूएचसी/यूएचपी/यूएफडब्ल्यूसी<br>6 = CHC सीएचसी<br>7 = APHC/NPHC/BPHC एपीएचसी/एनपीएचसी/बीपीएचसी<br>8 = SUB-CENTER उपकेन्द्र<br>9 = ANGANWADI CENTER आँगनवाड़ी केन्द्र<br>10 = VHND ग्राम स्वास्थ्य एवं पोष्ण दिवस<br>11 = NGO HOSPITAL/CLINIC गैर सरकारी संस्था का हस्पताल/क्लीनिक<br>12 = PRIVATE HOSPITAL/CLINIC प्राइवेट हस्पताल/क्लीनिक<br>88 = OTHERS (SPECIFY) अन्य (स्पष्ट करें)
C3.	**IF C1 = 0 & C2 = 0 SKIP TO C5A**<br><br>Who all did your antenatal checkups during your pregnancy?<br>जब आप गर्भवती थी तब आपकी प्रसव–पूर्व जाँच किस–किस ने की थी?<br><br>**PROBE : ANY OTHERS?**<br>çkc ¼ dN vkj\<br><br>**RECORD ALL MENTIONED**<br>lHkh mÙkj fy[kA	1 = GOVERNMENT DOCTOR सरकारी डाक्टर<br>2 = PRIVATE DOCTOR प्राइवेट डाक्टर<br>3 = STAFF NURSE स्टाफ नर्स<br>4 = LHV लेडी हैल्थ विजिटर (एल.एच.वी.)<br>5 = MALE HEALTH WORKER पुरुष स्वास्थ्य कर्मी<br>6 = ANM ए.एन.एम.<br>7 = OTHER HEALTH PERSONNEL अन्य स्वास्थ्य कर्मी<br>8 = ASHA आशा<br>9 = AWW आँगनवाड़ी कार्यकर्ता<br>10 = SBA/TRAINED DAI एसबीए/प्रशिक्षित दाई<br>11 = DAI दाई<br>12 = RMP आर.एम.पी. (झोला छाप डाक्टर)<br>88 OTHER (SPECIFY) अन्य (स्पष्ट करें)
C3a	In which months of your pregnancy did you receive first antenatal check-up?<br>गर्भावस्था के कौनसे महीने में पहली बार प्रसव–पूर्व जाँच हुई थी?	☐ COMPLETED PREGNANCY MONTHS<br>गर्भवस्था के महीने
C4.	When you were pregnant, how many times did you receive antenatal checkup?<br>जब आप गर्भवती थी तब आपकी कितनी बार प्रसव–पूर्व जाँच हुई थी?	☐ NO. OF TIMES इतनी बार<br>99 DO NOT KNOW पता नहीं

C5. During your pregnancy, as part of your antenatal check-ups were any of the following done at least once?
जब आप गर्भवती थी तब क्या आपकी प्रसव–पूर्व जाँच के रूप में निम्नलिखित जाँच कम से कम एक बार की गयी थी?

READ EACH ITEM BELOW (a-h) AND CODE YES OR NO FOR EACH. IF 'NO' GO TO NEXT LETTER
¼,&,p½ rd i<+ vkj iR;d d fy, gk ;k ugh dk dkM djA ;fn ugh g vxy v{kj ij tk;A

	Tests<br>जाँचें	YES हाँ	NO<br>नहीं	DO NOT KNOW पता नहीं
a.	Were you weighed? क्या आपका वजन लिया गया था?	1	0	99
b.	Was your blood pressure measured? क्या आपके रक्तचाप (ब्लड प्रैशर) की जाँच की गई थी?	1	0	99
c.	Did you give a urine sample? क्या आपने पिशाप का नमूना दिया था?	1	0	99
d.	Was your blood checked for hemoglobin level? हीमोग्लोबिन का स्तर जानने के लिये क्या आपकी खून कि जाँच की गयी थी?	1	0	99
e.	Was your abdomen checked? क्या आपके पेट की जाँच की गई थी?	1	0	99
f.	Was your ultrasound done? क्या आपका अल्ट्रासाउंड किया गया था?	1	0	99

C5A.	During your pregnancy, were you identified as anaemic?	1 = YES हाँ<br>0 = NO नहीं<br>99 = DO NOT KNOW पता नहीं

	**जब आप गर्भवती थी तब क्या आपको बताया गया था के आपको खून की कमी है?**	
C6.	During your pregnancy, were you given a TT injection?<br>जब आप गर्भवती थी तो क्या आपको टि्टनस की सुई लगी थी?	1 = YES हाँ<br>0 = NO नहीं → **GO TO C8**<br>99 = DO NOT KNOW पता नहीं → **GO TO C8**
C7.	During your pregnancy, how many times did you receive a tetanus injection?<br>जब आप गर्भवती थी तब आपको कितने टि्टनस की सुई लगी थी?	☐☐ NO. OF TIMES कितनी बार<br>99 = DO NOT KNOW/REMEMBER पता नहीं/याद नहीं
C8.	During your pregnancy with, did you receive any Iron Folic Acid (IFA) tablets?<br>जब आप गर्भवती थी तब क्या आपको आई.एफ.ए. (ताकत की लाल गोली) की गोलियाँ मिली थी?<br><br>**SHOW SAMPLES OF IFA TABLETS**<br>vkb-,Q-,- dh xkfy;k d ueu fn[kk;A	1 = YES हाँ<br>0 = NO नहीं → **GO TO C10B**<br>99 = DO NOT KNOW पता नहीं → **GO TO C10B**
C9.	In which month of pregnancy did you buy or receive IFA tablets for the first time?<br>गर्भावस्था के किस महीने में आपको पहली बार आई.एफ.ए. (ताकत की लाल गोली) की गोलियाँ मिली थी?	☐ COMPLETED MONTHS पूर्ण महीने<br>99 = DO NOT KNOW पता नहीं
C10.	How many tablets did you buy or receive in total during your whole pregnancy?<br>जब आप गर्भवती थी तब आपको आई.एफ.ए. (ताकत की लाल गोली) की कितनी गोलियाँ मिली थी?<br><br>**PROBE FOR WHETHER TABLETS ARE ADULT (LARGE) OR PEDIATRIC (SMALL) DOSE.**<br>iN fd ;g xkfy;k cMh Fkh ;k NkVh FkhA	☐☐☐ NUMBER OF LARGE TABLETS बड़ी गोलियों की संख्या<br>☐☐☐ NUMBER OF SMALL TABLETSछोटी गोलियों की संख्या<br>999 DO NOT REMEMBER याद नहीं
C10 A.	During the whole pregnancy, how many tablets did you consume?<br>जब बच्चा आपके गर्भ में था तब आपने आई.एफ.ए. (ताकत की लाल गोली) की कितनी गोलियाँ खायी थी?	☐☐☐ NUMBER OF LARGE TABLETS बड़ी गोलियों की संख्या<br>☐☐☐ NUMBER OF SMALL TABLETSछोटी गोलियों की संख्या<br>999 DO NOT REMEMBER याद नहीं
C10 B.	During your pregnancy, were you given or did you buy any iron folic syrup?<br>जब बच्चे आपके गर्भ में था, क्या आपको आईरन फोलिक एसिड (ताकत) की सिरप मिली थी या आपने खरीदी थी?	1 = YES हाँ<br>0 = NO नहीं→ **GO TO C11**
C10 C.	In which month of pregnancy did you receive IFA syrup bottles for the first time?<br>गर्भावस्था के कौनसे महीने में आपको पहली बार IFA सिरप की बोतले मिली थी?	☐ COMPLETED MONTHS पूर्ण महीने<br>99 DO NOT KNOW पता नहीं
C10 D.	How many IFA syrup bottles did you receive in total during your whole pregnancy?<br>जब बच्चे आपके गर्भ में था तब आपने आई.एफ.ए. की सिरप की कितनी बोतलें मिली थी?	☐☐.☐ NUMBER OF BOTTLES<br>99 DO NOT REMEMBER
C10E.	During the whole pregnancy with [CHILD NAME], how many bottles did you consume?<br>जब बच्चे आपके गर्भ में था तब आपने आई.एफ.ए. की सिरप की कितनी बोतलें खायी थी?	☐☐. . ☐ NUMBER OF BOTTLES<br>99= DO NOT REMEMBER
C11.	**ASK C11 IF C8=1 OR C10B=1**<br>During the whole pregnancy, for how many days did you take the tablets or syrup?<br>जब आप गर्भवती थी पूरी गर्भावस्थ के दौरान आपने कितने दिनों तक आईरन फोलिक एसिड (ताकत) की गोली या सिरप खायी थी?<br><br>**IF ANSWER IS NOT NUMERIC, PROBE FOR APPROXIMATE NUMBER OF DAYS.**<br>;fn mÙkj l[;k e ugh g] iN fd fnuk dh l[;k yxHkx D;k FkhA	☐☐☐ NO OF DAYS FOR LARGE TABLETS बड़ी गोलियाँ खाने के दिनों की संख्या<br>☐☐☐ NO OF DAYS FOR SMALL TABLETS छोटी गोलियों दिनों की संख्या<br>☐☐☐ NO OF DAYS FOR SYRUP सिरप खाने के दिनों की संख्या<br>999 DO NOT REMEMBER याद नहीं
C11 A1.	During your pregnancy with [CHILD NAME], were you given or did you buy any calcium tablets?	1= YES हाँ<br>0= NO नहीं → **GO TO C11A4**

	जब (बच्चे का नाम) आपके गर्भ में था, क्या आपको केलसियम की गोली मिली थी या आपने खरीदी थी?	
C11 A2	During your pregnancy with [CHILD NAME], how many calcium tablets were you given or did you buy?<br>जब (बच्चे का नाम) आपके गर्भ में था, आपको केलसियम की कितनी गोली मिली थी या आपने खरीदी थी?	☐☐☐ NUMBER OF TABLETS गोलियों की संख्या<br>999 =DO NOT REMEMBER याद नहीं
C11 A3	During the whole pregnancy with [CHILD NAME], how many tablets did you consume?<br>जब (बच्चे का नाम) आपके गर्भ में था तब आपने केलसियम की कितनी गोलियाँ खायी थी?	☐☐☐ NUMBER OF TABLETS गोलियों की संख्या<br>999 DO NOT REMEMBER याद नहीं
C11 A3A	**ASK IF C11A1=1**<br>During the whole pregnancy, for how many days did you take the tablets?<br>जब आप गर्भवती थी पूरी गर्भावस्थ के दौरान आपने कितने दिनों तक गोली खायी थी?<br><br>**IF ANSWER IS NOT NUMERIC, PROBE FOR APPROXIMATE NUMBER OF DAYS.**<br>;fn mÙkj l[;k e ugh g] iN fd fnuk dh l[;k yxHkx D;k FkhA	☐☐☐ NO OF DAYS FOR TABLETS गोलियाँ खाने के दिनों की संख्या<br>999=DO NOT REMEMBER याद नहीं
C11 A4	During your pregnancy with [CHILD NAME], did you receive any Take Home Ration (THR) from the anganwadi centre?<br>आप जब गर्भवती थी तब क्या आपको अनंगवादी केंद्र से पुरत आहार मिलता था?	1 = YES हाँ<br>0= NO नहीं GOTO C11A6
C11 A5	For how long did you receive THR during your pregnancy?<br>गर्भवस्था के दौरान आपको कितने समय तक पुरत आहार मिला था?	☐ ☐☐<br>1 = WEEKS घंटे<br>2 = MONTHSदिन<br>= WEEKS सप्ताह 999=DO NOT REMEMBER याद नहीं
C11A6	During your pregnancy with [CHILD NAME], did you take rest more frequently than when you were not pregnant?<br>जब (बच्चे का नाम) आपके गर्भ में था तब क्या आप गर्भवती न होने के समय की तुलना में अधिक बार आराम करती थी?	1 = YES हाँ<br>0 = NO नहीं
C11A7	During your pregnancy with [CHILD NAME] did you consume more food as compared to when you were not pregnant?<br>जब (बच्चे का नाम) आपके गर्भ में था तब क्या आप गर्भवती न होने के समय की तुलना में अधिक भोजन करती थी?	1 = YES हाँ<br>= NO नहीं
C11A8	Which of the following foods did you consume almost daily during pregnancy?<br><br>कौनसे पढके सुनाई जाने वाली चीज़ें आपने रोजाना गर्भवस्था के दौरान खाया?<br><br>MULTIPLE CHOICE	1= Dark green leafy vegetables हरि सब्जियां<br>2=Yellow or orange fruits or vegetables (e.g. Pumpkin, carrots, ripe mango, ripe papaya) पीली या नारंगी फल और सब्जी<br>3=Lentils, dal दल<br>4=Roti, bread or rice रोटी, चावल, ब्रेड<br>5=Eggs अन्दा<br>6=Meat, chicken or fish मॉस<br>7=Milk, paneer or yogurt दूध, पनीर, दही<br>88=Othersअन्य
C11A9	During your pregnancy with [CHILD NAME], did you avoid picking up heavy objects?<br>जब (बच्चे का नाम) आपके गर्भ में था तब क्या आप भारी सामान उठाने से बचती थी?	1 = YES हाँ<br>0 = NO नहीं
C11 A.	Are you aware about the different danger signs during pregnancy which require visit to a health facility or contacting a healthcare provider?<br>क्या आप गर्भावस्था के दौरान होने वाले खतरे के विभिन्न लक्षणों के बारे में जानती हैं जिनके लिए स्वास्थ्य केंद्र जाने की या स्वास्थ्य प्रदाता से संपर्क करने की जरूरत होती है?	1 = YES हाँ<br>0 = NO नहीं → **GO TO C11D**

C11 B.	What are the different danger signs during pregnancy which require visit to a health facility or contacting a healthcare provider?<br>गर्भावस्था के दौरान खतरे के वह कौन से लक्षण हैं जिनके लिए स्वास्थ्य केंद्र जाने की या स्वास्थ्य प्रदाता से संपर्क करने की जरूरत होती है?<br><br>**PROBE : ANY OTHERS?**<br>çkc % dN vkj\<br><br>**RECORD ALL MENTIONED**<br>lHkh mÙkj fy[kA	1 = EXCESSIVE VAGINAL BLEEDING योनि से अत्यधिक खून निकलना<br>2 = DIFFICULTY IN BREATHING सांस लेन में कठिनाई<br>3 = SEVERE HEADACHE तेज सिर दर्द<br>4 = BLURRED VISION धुंधला दिखना<br>5 = SWELLING OF THE HANDS, FEET, BODY OR FACE हाथों, पैरों, शरीर और चेहरे का सूजन<br>6 = HIGH FEVER तेज बुखार<br>7 = LOSS OF CONSCIOUSNESS होश खोना<br>8 = SEVERE ABDOMINAL PAIN पेट में तेज दर्द<br>9 = CONVULSIONS दौरे पड़ना<br>10 = HIGH BLOOD PRESSURE उच्च रक्तचाप<br>88 OTHERS (SPECIFY) अन्य (स्पष्ट करें)
C11 C.	From where did you get the information about these danger signs during pregnancy?<br>गर्भावस्था के दौरान खतरे के लक्षणों के बारे में आपको कहाँ से जानकारी मिली थी?<br><br>**PROBE : ANY OTHERS?**<br>çkc % dN vkj\<br><br>**RECORD ALL MENTIONED**<br>lHkh mÙkj fy[kA	1 = GOVERNMENT DOCTOR सरकारी डाक्टर<br>2 = PRIVATE DOCTOR प्राइवेट डाक्टर<br>3 = STAFF NURSE स्टाफ नर्स<br>4 = LHV लेडी हैल्थ विजिटर (एल.एच.वी.)<br>5 = MALE HEALTH WORKER पुरुष स्वास्थ्य कर्मी<br>6 = ANM ए.एन.एम.<br>7 = OTHER HEALTH PERSONNEL अन्य स्वास्थ्य कर्मी<br>8 = ASHA आशा<br>9 = AWW आँगनवाड़ी कार्यकर्ता<br>10 = SBA/TRAINED DAI एसबीए/प्रशिक्षित दाई<br>11 = DAI दाई<br>12 = RMP आर.एम.पी. (झोला छाप डाक्टर)<br>13 = FAMILY MEMBER परिवार के सदस्य<br>14 = FRIENDS/NEIGHBOUR दोस्त/पड़ौसी<br>15 = RADIO/TV/NEWS PAPER रेडियो/टेलिविजन/अखबार<br>16 = MOBILE SMS मोबाइल एसएमएस<br>88 = OTHERS (SPECIFY) अन्य (स्पष्ट करें)<br>98 = NO ONE कोई नहीं<br>99 DO NOT KNOW मालूम नहीं
C11 D.	During your pregnancy with [CHILD NAME] were you told to visit a doctor/facility in case of any pregnancy complications?<br>जब (बच्चे का नाम) आपके गर्भ मे था तब क्या आपको यह बताया गया था कि गर्भावस्था से संबंधित किसी भी परेशानी के मामले में डाक्टर के पास/स्वास्थ्य केंद्र पर जायें?	1 = YES हाँ<br>0 = NO नहीं

C12.	During your pregnancy what kind of problems did you experience?<br>जब आप गर्भवती थी तब आपको किस प्रकार की परेशानी हुई थी?<br><br>**DO NOT READ LIST. CODE ALL MENTIONS IN FIRST COLUMN. THEN READ EACH ITEM BELOW (a-j) THAT WASN'T MENTIONED AND CODE RESPONSE IN SECOND COLUMN. IF NOT MENTIONED, SKIP TO NEXT LETTER.**<br>lwph dks u i<sA igys dkye esa lHkh mYys[k fd;s x;s mÙkjksa dks dksM djsaA blds ckn mu fcUnqvksa ¼,&t½ dks i<s+ ftudk mYys[k ugha fd;k x;k Fkk vkSj nwljs dkye esa mÙkj dks dksM djsaA ;fn ugha crk;k x;k vxys fcUnq ij tk;sA	1 = YES, SPONTANEOUS<br>हाँ, स्वयं बताया	2 = YES, AFTER READIN G<br>हाँ, पढ़ने के बाद	0 = NO<br>नहीं
a.	Excessive vaginal bleeding योनी से अत्यधिक रक्तस्राव	1	2	0
b.	Difficulty in breathing साँस लेन में कठिनाई	1	2	0
c.	Severe headache तेज सिर दर्द	1	2	0
d.	Blurred vision धुंधला दिखना	1	2	0
e.	Swelling of the hands, feet, body or face हाथों, पैरों, शरीर और चेहरे पर सूजन	1	2	0
f.	High fever तेज बुखार	1	2	0

g.	Loss of consciousness चेतना (होश) खोना	1	2	0
h.	Severe abdominal pain पेट में तेज दर्द	1	2	0
i.	Convulsions दौरे पड़ना	1	2	0
j.	High Blood Pressure उच्च रक्तचाप	1	2	0

C13.	**ASK ONLY IF ANY OF THE RESPONSES IN C12 (a-j) IS CODED 1 OR 2, ELSE GO TO C15**<br>rHkh iNs tc lh12 ¼,&t½ esa fn;s x;s fdlh mÙkj dk dksM 1 gks(ugha rks lh36 ij tk;sA<br>Did you seek treatment for this problem?<br>क्या आपने इस समस्या का इलाज कराया था?	1 = YES हाँ<br>0 = NO नहीं → **GO TO C15**
C14.	From where did you seek treatment?<br>आपने इलाज कहाँ से कराया था?<br><br>**PROBE : ANY OTHERS?**<br>çkc % dqN vkSj\<br><br>**RECORD ALL MENTIONED**<br>lHkh mÙkj fy[ksA	1 = GOVERNMENT/MUNICIPAL HOSPITAL सरकारी/नगरपालिका का अस्पताल<br>2 = GOVERNMENT DISPENSARY सरकारी दवाखाना<br>3 = UHC/UHP/UFWC यूएचसी/यूएचपी/यूएफडब्ल्यूसी<br>4 = CHC सीएचसी<br>5 = APHC/NPHC/BPHC एपीएचसी/एनपीएचसी/बीपीएचसी<br>6 = SUB-CENTER उपकेन्द्र<br>7 = ANGANWADI CENTER आँगनवाड़ी केन्द्र<br>8 = VHND ग्राम स्वास्थ्य एवं पोषण दिवस<br>9 = NGO HOSPITAL/CLINIC गैर सरकारी संस्था का अस्पताल/क्लीनिक<br>10 = PRIVATE HOSPITAL/CLINIC प्राइवेट अस्पताल/क्लीनिक<br>11 = MEDICINE SHOP दवा की दुकान<br>12 = FOLK HEALER ओझा/वैद्य/झाड़फूँक<br>13 = HOME REMEDIES घरेलू उपचार<br>14 = RMP आर.एम.पी. (झोला छाप डाक्टर)<br>88 OTHERS (SPECIFY) अन्य (स्पष्ट करें)
C15.	Did the ASHA come to meet you at your home during your pregnancy?<br>जब आप गर्भवती थी तब क्या आशा आपके घर आपसे मिलने आई थी?	1 = YES हाँ<br>0 = NO नहीं → **GO TO C17**
C16.	How many times did the ASHA come to meet you at your home during your pregnancy?<br>जब आप गर्भवती थी तब आशा आपके घर आपसे मिलने कितनी बार आई थी?	☐☐ NO. OF TIMES इतनी बार<br>99 DO NOT REMEMBER याद नहीं
C17.	Did the AWW come to meet you at your home during your pregnancy?<br>जब आप गर्भवती थी तब क्या आँगनवाड़ी कार्यकर्ता आपके घर आपसे मिलने आई थी?	1 = YES हाँ<br>1 = NO नहीं → **GO TO C19**
C18.	How many times did the AWW come to meet you at your home during your pregnancy ?<br>जब आप गर्भवती थी तब आँगनवाड़ी कार्यकर्ता आपके घर आपसे मिलने कितनी बार आई थी?	☐☐ NO. OF TIMES इतनी बार<br>99 DO NOT REMEMBER याद नहीं
C19.	Did the ANM come to meet you at your home during your pregnancy?<br>जब आप गर्भवती थी तब क्या ए.एन.एम. आपके घर आपसे मिलने आई थी?	1 = YES हाँ<br>0 = NO नहीं → **GO TO C21**
C20.	How many times did the ANM come to meet you at your home?<br>जब आप गर्भवती थी तब ए.एन.एम. आपके घर आपसे मिलने कितनी बार आई थी?	☐☐ NO. OF TIMES इतनी बार<br>99 DO NOT REMEMBER याद नहीं

C20A	**THINKING ABOUT THE LAST VISIT**<br>Which frontline worker had visited you the last time at home?<br><br>पिछली बार आपसे मिलने आपके घर आशा, अन्गंवादी कार्यकर्ता, ऐ एन एम् में से कौन आई थी?	1=ASHA<br>2=AWW<br>3=ANM<br>9=NONE
C20B	How much time the frontline worker who visited you last spend during the session (in minutes)?	☐☐<br>MINUTES मिनट

	पिछली बार आपके घर जो कार्यकर्ता आई थी उसने कितना समय बिताया था ? (मिनट में)	
C20A	On what topics did she advise/counsel you?<br><br>आपको उसने चीज़ों के बारें में सलाह दी ?<br><br>**PROBE : ANY OTHERS?**<br>çkc ¶ dN vkj\<br><br>**RECORD ALL MENTIONED**<br>lHkh mÙkj fy[kA	1= MATERNAL NUTRITION मात्री पोषण<br>2= IFA CONSUMPTION IFA की सिरप या गोलियां लेना<br>3= DIETARY DIVERSITY FOR NUTRITION आहार बिबीधता ताकि महिला को ज़रुरत का पोषण मिले<br>4= TAKING CARE OF YOUR HEALTH DURING PREGNANCY गर्भावस्था के दौरान अपने स्वास्थ्य की देखभाल करना<br>5= GOING TO THE HOSPITAL FOR A CHECK-UP DURING PREGNANCY गर्भावस्था के दौरान जांच के लिए अस्पताल जाना<br>6= GOING TO A HEALTH FACILITY FOR DELIVERY प्रसव कराने के लिए स्वास्थ्य केंद्र या अस्पताल जाना<br>7= PLANNING IN ADVANCEFOR DELIVERY प्रसव के लिए पहले से तैयारी किस प्रकार करनी है<br>8= HOW TO TAKE CARE OF THE NEWBORN नवजाति शिशु की देखरेख कैसे करनी है<br>9=EXCLUSIVE BREASTFEEDING केवल स्तनपान<br>10= COMPLEMENTARY FEEDING पूरक आहार/ उपरी आहार<br>11= IMMUNIZATION टीकाकरण<br>12= FAMILY PLANNING परिवार नियोजन<br>13= HANDWASHING AND SANITATION हाथ धोना और साफसफाई<br>88= OTHERS (SPECIFY) अन्य (स्पष्ट करें)
C20C	Was she clear in explaining the topics?<br>क्या वह विषयों को स्पष्ट रूप से समझा पायी थी?	1 = YES हाँ<br>0 = NO नहीं
C20D	Were you able to understand everything?<br>क्या आप सब कुछ समझ पायी थी?	1 = YES हाँ<br>0 = NO नहीं
C20 E	Was she able to answer all your queries clearly?<br>क्या वह आपके सभी सवालों का स्पष्ट रूप से उत्तर दे पायी थी?	1 = YES हाँ<br>0 = NO नहीं
**C20F**	What job aid tools did she use during this most recent visit?<br>आशा/आंगनवाडी कार्यकर्ता ने सबसे हाल के इस दौरे के समय किस साधन या उपकरण का उपयोग किया?<br>**CODE ALL THAT APPLY**<br>lHkh ykxw gksus okys dksM djsa	1= PLASTIC PICTURE CARDS (describe mobile kunji cards) प्लास्टिक के पिक्चर कार्ड (मोबाइल कुंजी)<br>2= PAMPHLET पम्फलेट<br>3=KATORA/SPOON सी कटोरा/चम्मच<br>4= COPPER-T ई कॉपर–टी<br>5= MALA-D एफ माला–डी<br>88=OTHERS (SPECIFY) अन्य (बताएं)
**C20H.**	**<u>If C20F=1 OR C20G=1, ASK C20H. ELSE GO TO C21.</u>**<br><br>Did you find the usage of these tools useful?<br>क्या आपने इन उपकरणों को उपयोगी पाया?	YES हॉ...1<br>NO नहीं...............0 **GO TO C21→**
**C20I.**	Which of these tools helped you to understand the issues explained by FLW?<br>इनमें से कौन कौन से उपकरणों से आपको अग्रणी कतार की कार्यकर्ता (एफएलडब्ल्यू) द्वारा स्पष्ट किये गये मुद्दों को समझने में मदद मिली?	PLASTIC PICTURE CARDS प्लास्टिक के पिक्चर कार्ड......1<br>PAMPHLET पम्फलेट ....2<br>KATORA कटोरा ....3<br>COPPER-T कॉपर–टी...4

	**CODE ALL THAT APPLY**<br>lHkh ykxw gksus okys dksM djs	MALA-D माला डी .... 5<br>OTHER अन्य............................ 88

C21.	Did the LHV/Government/ Doctor come to meet you at your home during your pregnancy?<br>जब आप गर्भवती थी तब क्या एल.एच.वी./सरकारी डाक्टर आपके घर आपसे मिलने आये थे?	1 = YES हाँ<br>0 = NO नहीं → **GO TO** C23
C22.	How many times did the LHV/Government Doctor come to meet you at your home?<br>जब आप गर्भवती थी तब ` एल.एच.वी./सरकारी डाक्टर आपके घर आपसे मिलने कितनी बार आये थे?	☐☐ NO. OF TIMES इतनी बार<br>99 DO NOT REMEMBER याद नहीं
C23.	Where did the termination of pregnancy take place?<br>गर्भसमापन कहाँ हुआ था?<br><br>**IF AT A FACILITY, PROBE: WHAT TYPE OF FACILITY WAS THIS? IF UNABLE TO DETERMINE IF A HOSPITAL, HEALTH CENTRE, OR CLINIC IS PUBLIC OR PRIVATE MEDICAL SECTOR, WRITE THE NAME OF THE PLACE.**<br>;fn LokLF; lfo/kk esa rc çkc djsa LokLF; lfo/kk fdl çdkj dh Fkh\ ;fn ;g fu/kkZfjr djus esa leL;k gks jgh gS fd LokLF; lfo/kk] vLirky Fkk LokLF; dsUæ lkoZtfud Fkk ;k futh {ks= dk] LFkku dk uke fy[ksA<br>______________________________<br>NAME OF PLACE स्थान का नाम<br><br>**CODE ONLY ONE**<br>dksy ,d mÙkj	1 = HOME घर पर<br>2 = GOVERNMENT/MUNICIPAL HOSPITAL सरकारी/नगरपालिका का अस्पताल<br>3 = GOVERNMENT DISPENSARY सरकारी दवाखाना<br>4 = UHC/UHP/UFWC यूएचसी/यूएचपी/यूएफडब्ल्यूसी<br>5 = CHC सीएचसी<br>6 = APHC/NPHC/BPHC एपीएचसी/एनपीएचसी/बीपीएचसी<br>7 = SUB-CENTER उपकेन्द्र<br>8 = NGO HOSPITAL/CLINIC गैर सरकारी संस्था का अस्पताल/क्लीनिक<br>9 = PRIVATE HOSPITAL/CLINIC प्राइवेट अस्पताल/क्लीनिक<br>88 OTHERS (SPECIFY) अन्य (स्पष्ट करें)
C24.	Who conducted the pregnancy termination?<br>गर्भसमापन किसने किया था?<br><br>**CODE ONLY ONE**<br>dksy ,d mÙkj	1 = GOVERNMENT DOCTOR सरकारी डाक्टर<br>2 = PRIVATE DOCTOR प्राइवेट डाक्टर<br>3 = STAFF NURSE स्टाफ नर्स<br>4 = LHV लेडी हैल्थ विजिटर (एल.एच.वी.)<br>5 = MALE HEALTH WORKER पुरुष स्वास्थ्य कर्मी<br>6 = ANM ए.एन.एम.<br>7 = OTHER HEALTH PERSONNEL अन्य स्वास्थ्य कर्मी<br>8 = ASHA आशा<br>9 = AWW आँगनवाड़ी कार्यकर्ता<br>10 = SBA/TRAINED DAI एसबीए/प्रशिक्षित दाई<br>11 = DAI दाई<br>12 = RMP आर.एम.पी. (झोला छाप डाक्टर)<br>13 = FAMILY MEMBER परिवार के सदस्य<br>14 = FRIENDS/NEIGHBOUR मित्र/पड़ौसी<br>88 = OTHERS (SPECIFY) अन्य (स्पष्ट करें)<br>98 = NO ONE कोई नहीं<br>99 DO NOT KNOW पता नहीं
C25.	Who assisted the pregnancy termination?<br>गर्भपात करने मे किस किस ने सहायता की थी?<br><br>**PROBE FOR THE TYPE OF PERSON AND RECORD ALL PERSONS ASSISTING. ANY OTHERS? RECORD ALL MENTIONED**<br>çkc % ml 0;fDr dk izdkj D;k Fkk ftlus vkidh lgk;rk dh Fkh vkj lHkh 0;fDr;ksa dk ntZ djsaA D;k dksbZ vkj Fkk\ lHkh mÙkj fy[ksA	1 = GOVERNMENT DOCTOR सरकारी डाक्टर<br>2 = PRIVATE DOCTOR प्राइवेट डाक्टर<br>3 = STAFF NURSE स्टाफ नर्स<br>4 = LHV लेडी हैल्थ विजिटर (एल.एच.वी.)<br>5 = MALE HEALTH WORKER पुरुष स्वास्थ्य कर्मी<br>6 = ANM ए.एन.एम.<br>7 = OTHER HEALTH PERSONNEL अन्य स्वास्थ्य कर्मी<br>8 = ASHA आशा<br>9 = AWW आँगनवाड़ी कार्यकर्ता<br>10 = SBA/TRAINED DAI एसबीए/प्रशिक्षित दाई<br>11 = DAI दाई<br>12 = RMP आर.एम.पी. (झोला छाप डाक्टर)

	**IF RESPONDENT SAYS NO ONE ASSISTED, PROBE TO DETERMINE WHETHER ANY ADULTS WERE PRESENT AT THE DELIVERY.**<br>यदि उत्तरदाता यह कहती है कि किसी ने सहायता नहीं की, यह निर्धारित करने के लिए पूछें कि क्या कोई वयस्क व्यक्ति गर्भपात के समय उपस्थित था।	13 = FAMILY MEMBER परिवार के सदस्य<br>14 = FRIENDS/NEIGHBOUR मित्र/पड़ौसी<br>88 = OTHERS (SPECIFY) अन्य (स्पष्ट करें)<br>98 = NO ONE कोई नहीं<br>99 DO NOT KNOW पता नहीं
C25A.	Did any health service provider give you this tablet **[SHOW MISOPROSTOL TABLETS]** to consume immediately after abortion?<br>क्या किसी स्वास्थ्य दाता ने आपके गर्भपात के तुरन्त बाद खाने के लिए यह गोली ¼feb l kçkLVky dh xksfy;k fn[kk;h½ दी थी?	1 = YES हाँ<br>0 = NO नहीं → **GO TO C26A**
C25B.	How many tablets did the health service provider give you?<br>स्वास्थ्य सेवा प्रदाता ने आपको कितनी गोलियाँ दी थी?	☐ NUMBER OF TABLETS गोलियों की संख्या
C25C.	How many tablets did you consume?<br>आपने कितनी गोलियाँ खाई थी?	☐ NUMBER OF TABLETS गोलियों की संख्या<br>0 = NO TABLETS CONSUMED कोई गोली नहीं खायी → **GO TO** C26A
C26A.	Did you go to a health care facility after the pregnancy termination?<br>क्या आप गर्भपात के बाद किसी स्वास्थ्य सुविधा केंद्र में गए थें?	1 = YES हाँ<br>0 = NO नहीं
C26B.	**ASK ONLY IF C26A=1, ELSE GO TO C33**<br>Who all went with you to the health facility for your abortion/miscarriage?<br>जब गर्भपात/ बच्चा खराब हो गया था तब आप स्वास्थ्य सुविधा गयी थी, आपके साथ कौन गया था?<br><br>**PROBE : ANY OTHERS?**<br>çkc % dN vkj\<br><br>**RECORD ALL MENTIONED**<br>lHkh mÙkj fy[ksA	1 = HUSBAND पति<br>2 = MOTHER-IN-LAW सास<br>3 = MOTHER माँ<br>4 = OTHER RELATIVES अन्य रिश्तेदार<br>5 = FRIENDS/NEIGHBORS मित्र/पड़ौसी<br>6 = ANM ए.एन.एम.<br>7 = DAI दाई<br>8 = SBA/TRAINED DAI एसबीए/प्रशिक्षित दाई<br>9 = ASHA आशा<br>10 = AWW आँगनवाड़ी कार्यकर्ता<br>88 = OTHERS (SPECIFY) अन्य (स्पष्ट करें)<br>F98 NO ONE कोई नहीं
C33A	**<u>ASK ONLY IF C0=1 (MISCARRIAGE)</u>**<br>What was the reason that caused the pregnancy termination?<br>ग्रभावस्था के समापन के क्या कारण थे?<br><br>**MULTIPLE RESPONSE POSSIBLE**	1= EXCESSIVE VAGINAL BLEEDING योनी से अत्यधिक रक्तस्राव<br>2= SEVERE ABDOMINAL PAIN पेट में तेज दर्द<br>3= CONVULSIONS दौरे पड़ना<br>4 = FOUL SMELLING VAGINAL DISCHARGE योनि से बदबूदार पानी निकलना<br>88 =OTHERS (SPECIFY) अन्य (स्पष्ट करें)<br>99= DO NOT KNOW पता नहीं
C34.	**<u>ASK ONLY IF C0=2 (ABORTION)</u>**<br>What was the main reason for terminating the pregnancy?<br>गर्भपात करने का मुख्य कारण क्या था?	1 = UNWANTED PREGNANCY AT THAT TIME<br>उस समय बच्चा नही चाहिए था<br>2 = HAD DESIRED NUMBER OF CHILDREN<br>जितने बच्चे चाहिए थे उतने हैं।<br>3 = CANNOT AFFORD ANOTHER CHILD<br>एक और बच्चा संभालने में समर्थ नही है।<br>4 = OTHER CHILD ARE VERY YOUNG SO KEEPING A GAP IN THEM<br>अभी बच्चा छोटा हैं इसलिए अगले बच्चे में अंतर रखने के लिए<br>5 = CONCERNED ABOUT OWN HEALTH<br>अपने स्वास्थ्य की चिंता हैं।<br>6= HAD PREGNANCY COMPLICATIONS<br>गर्भावस्था के दौरान गतिलता थी<br>88 = OTHERS (SPECIFY)<br>अन्य (स्पष्ट करें)
C34A	Do you know of any danger signs or symptoms that a woman might have during delivery or within the first month after	1 = YES हाँ<br>0 = NO नहीं → **GO TO C34D**

	delivery that would require seeking medical care?<br>क्या आप खतरे के ऐसे लक्षणों या संकेतों के बारे मे जानती हैं जिनका अनुभव महिला प्रसव के दौरान या प्रसव के बाद एक महीने के अन्दर कर सकती है और जिसके लिए चिकित्सा देखरेख की जरूरत होती है?	
C34B	Could you name some of the danger signs or symptoms that a woman might have during delivery or within the first month after delivery that would require seeking medical care?<br>क्या आप खतरे के ऐसे लक्षणों या संकेतों के बारे मे बता सकती हैं जिनका अनुभव महिला प्रसव के दौरान या प्रसव के बाद एक महीने के अन्दर कर सकती है और जिसके लिए चिकित्सा देखरेख की जरूरत होती है?<br><br>**PROBE : ANY OTHERS?**<br>çkc ¼ dN vkj\<br><br>**RECORD ALL MENTIONED**<br>lHkh mÙkj fy[kA	1 = PROLONGED LABOUR FOR MORE THAN 12 HOURS लंबे समय तक, 12 घंटे से अधिक समय तक प्रसव पीड़ा<br>2 = RETAINED PLACENTA गर्भनाल का अंदर रहना<br>3 = OBSTRUCTED LABOUR (MALPOSITION/PRESENTATION) रुकावट के साथ प्रसव (शिशु का गलत स्थिति में होना)<br>4 = SEVERE BLEEDING अत्यधिक खून निकलना<br>5 = DIFFICULTY BREATHING सांस लेने में कठिनाई<br>6 = SEVERE PAIN IN ABDOMEN पेट में तेज दर्द<br>7 = SEVERE WEAKNESS अत्यधिक कमजोरी<br>8 = CONVULSIONS दौरे पड़ना<br>9 = FOUL SMELLING VAGINAL DISCHARGE योनि से बदबूदार पानी निकलना<br>10 = FEVER बुखार<br>11 = SEVERE HEADACHE तेज सिर दर्द<br>12 = LOSS OF CONSCIOUSNESS होश खोना<br>13 = DOUBLE VISION एक के दो दिखना<br>88 OTHERS (SPECIFY) अन्य (स्पष्ट करें)
C34C	From where did you get the information about these danger signs that a woman can have during delivery or post-delivery?<br>प्रसव के दौरान या प्रसव के बाद महिला को खतरे के इन संकेतों के बारे में आपको कहाँ से जानकारी मिली थी?<br><br>**PROBE : ANY OTHERS?**<br>çkc ¼ dN vkj\<br><br>**RECORD ALL MENTIONED**<br>lHkh mÙkj fy[kA	1 = GOVERNMENT DOCTOR सरकारी डाक्टर<br>2 = PRIVATE DOCTOR प्राइवेट डाक्टर<br>3 = STAFF NURSE स्टाफ नर्स<br>4 = LHV लेडी हैल्थ विजिटर (एल.एच.वी.)<br>5 = MALE HEALTH WORKER पुरुष स्वास्थ्य कर्मी<br>6 = ANM ए.एन.एम.<br>7 = OTHER HEALTH PERSONNEL अन्य स्वास्थ्य कर्मी<br>8 = ASHA आशा<br>9 = AWW आँगनवाड़ी कार्यकर्ता<br>10 = SBA/TRAINED DAI एसबीए/प्रशिक्षित दाई<br>11 = DAI दाई<br>12 = RMP आर.एम.पी. (झोला छाप डाक्टर)<br>13 = FAMILY MEMBER परिवार के सदस्य<br>14 = FRIENDS/NEIGHBOUR दोस्त/पड़ौसी<br>15 = RADIO/TV/NEWS PAPER रेडियो/टेलिविजन/समाचार पत्र<br>16 = MOBILE SMS मोबाइल एसएमएस<br>88 = OTHERS (SPECIFY) अन्य (स्पष्ट करें)<br>98 = NO ONE कोई नहीं<br>F99 DO NOT KNOW पता नहीं
C34D	Were you told to visit a doctor/facility in case of any complications during delivery or in the first month after delivery?<br>क्या आपको यह बताया गया था कि प्रसव के समय या प्रसव के बाद एक महीने के अन्दर किसी भी जटिलता या समस्या होने पर डाक्टर/स्वास्थ्य केंद्र जायें?	1 = YES हाँ<br>0 = NO नहीं

C35	Did you experience any danger signs during the pregnancy termination or during the first month after pregnancy termination?<br>गर्भसमापन के समय या उसके बाद एक महीने के अन्दर क्या आपने किसी समस्या या परेशानी का अनुभव किया था?<br><br>**DO NOT READ LIST. CODE ALL MENTIONS IN FIRST COLUMN. THEN READ EACH ITEM BELOW (a-r) THAT WASN'T MENTIONED AND CODE RESPONSE IN SECOND COLUMN. IF NOT MENTIONED, SKIP TO NEXT LETTER.**<br>lph dk u i<A igy dkye e lHkh mYy[k fd; x; mÙkjk dk dkM djA bl d ckn mu fcUnvk ¼,&vkj½	1 = YES, SPONTANEOUS<br>हाँ, स्वयं बताया	2 = YES, AFTER READING<br>हाँ, पढ़ने के बाद	0 = NO<br>नहीं

	dk i<s ftudk mYys[k ugh fd;k x;k Fkk vkj nl js dkye es mÙkj dk dkM djsA ;fn ugh crk;k x;k vxys fcUnq ij tk;sA			
g.	Excessive bleeding before delivery प्रसव के पहले अत्यधिक खून निकलना	1	2	0
h.	Excessive bleeding immediately after delivery प्रसव के तुरन्त बाद अत्यधिक खून निकलना	1	2	0
i.	High Blood Pressure उच्च रक्तचाप	1	2	0
j.	Difficulty breathing साँस लेने में कठीनाई	1	2	0
k.	Severe pain in abdomen पेट में तेज दर्द	1	2	0
l.	Severe weakness अत्यधिक कमजोरी	1	2	0
m.	Convulsions दौरे पड़ना	1	2	0
n.	Foul smelling vaginal discharge योनी से बदबूदार स्राव	1	2	0
o.	Fever बुखार	1	2	0
p.	Severe headache तेज सिर दर्द	1	2	0
q.	Loss of consciousness बेहोश होना	1	2	0
r.	Blurred vision धुंधला दिखना	1	2	0
C36	**ASK ONLY IF IN ANY OF THE RESPONSES 0 (g-r) IS CODED 1 OR 2, ELSE GO TO C37A**<br>;g i'u rHkh iNs tc lh35 ¼,&vkj½ es fdlh mÙkj dk dkM 1 g(ugh rk lh38 ij tk;s<br><br>Did you seek treatment for this problem?<br>क्या आपने इस समस्या का इलाज कराया था?	1 = YES हाँ<br>0 = NO नहीं → **GO TO C37A**		
C37.	From where did you seek treatment?<br>आपने इलाज कहाँ कराया था?<br><br>**PROBE : ANY OTHERS?**<br>çkc ¼ dN vkj\<br><br>**RECORD ALL MENTIONED**<br>lHkh mÙkj fy[kA	1 = GOVERNMENT/MUNICIPAL HOSPITAL सरकारी /नगरपालिका का अस्पताल<br>2 = GOVERNMENT DISPENSARY सरकारी दवाखाना<br>3 = UHC/UHP/UFWC यूएचसी/यूएचपी/यूएफडब्ल्यूसी<br>4 = CHC सीएचसी<br>5 = APHC/NPHC/BPHC एपीएचसी/एनपीएचसी/बीपीएचसी<br>6 = SUB-CENTER उपकेन्द्र<br>7 = ANGANWADI CENTER आँगनवाड़ी केन्द्र<br>8 = VHND ग्राम स्वास्थ्य एवं पोष्ण दिवस<br>9 = NGO HOSPITAL/CLINIC गैर सरकारी संस्था का अस्पताल/क्लीनिक<br>10 = PRIVATE HOSPITAL/CLINIC प्राइवेट अस्पताल/क्लीनिक<br>11 = MEDICINE SHOP दवा की दुकान<br>12 = FOLK HEALER ओझा/वैद्‌य/झाड़फूँक<br>13 = HOME REMEDIES घरेलू उपचार<br>14 = RMP आर.एम.पी. (झोला छाप डाक्टर)<br>88 OTHERS (SPECIFY) अन्य (स्पष्ट करें)		
C37 A.	**ASK ONLY IF C23>1 OR C26A=1**<br><br>How long did you stay at the healthcare facility after abortion/MISCARRIAGE?<br>गर्भपात के बाद स्वास्थ्य सुविधा में कब तक रूकी थी?<br>**CODE HOURS, DAYS, OR WEEKS.**<br>घंटे, दिन और सप्ताह में कोड करें।	☐ ☐☐<br>1 = HOURS घंटे<br>2 = DAYS दिन<br>0 = WEEKS सप्ताह		
C40.	Did the ASHA come to your home to check on you within 24 hours after the pregnancy termination?<br>गर्भसमापन के बाद 24 घंटे के अन्दर क्या आशा आपकी जाँच करने आपके घर आई थी?	1 = YES हाँ<br>0 = NO नहीं<br>DO NOT KNOW irk ugha		
C41.	Did the AWW come to your home to check on you within 24 hours after the termination?<br>गर्भसमापन के बाद 24 घंटे के अन्दर क्या आँगनवाड़ी कार्यकर्ता आपकी जाँच करने आपके घर आई थी?	1 = YES हाँ<br>0 = NO नहीं<br>99 = DO NOT KNOW irk ugha		
C41a	After the abortion/miscarriage, how much iron folic acid tablets and syrups bottles did you receive or purchase?	Large Tablets ☐☐<br>Small Tablets☐☐		

	गर्भसमापन के बाद IFA की कितनी गोली और सिरप की बोतलें आपको मिली थी?	Bottles □□ . □
C41 b	How many IFA tablets/syrup bottles did you consume after the abortion/miscarriage?<br><br>गर्भसमापन के बाद IFA की कितनी गोली और सिरप की बोतलें आपने खायी थी?	Large Tablets □□<br>Small Tablets□□<br><br>Bottles □□ . □
C41e	**ASK IF C41B>0 OR C41D>0**<br>After the abortion/miscarriage, for how many days did you take the tablets or syrup?<br>गर्भपात या बचा गिर जाने के बाद आपने कितने दिनों तक आईरन फोलिक एसिड (ताकत) की गोली या सिरप खायी थी?<br><br>**IF ANSWER IS NOT NUMERIC, PROBE FOR APPROXIMATE NUMBER OF DAYS.**<br>;fn mÙkj l[;k e ugh g] iN fd fnuk dh l[;k yxHkx D;k FkhA	□□□ NO OF DAYS FOR LARGE TABLETS बड़ी गोलियाँ खाने के दिनों की संख्या<br>□□□ NO OF DAYS FOR SMALL TABLETS छोटी गोलियों दिनों की संख्या<br>□□□ NO OF DAYS FOR SYRUP सिरप खाने के दिनों की संख्या<br><br>DO NOT REMEMBER याद नहीं
C41j	Were you aware about family planning methods before your abortion?<br>abortion के पहले क्या आपको परिवार नियोजन की बिधि की जानकारी थी?	1 = YES हाँ<br>0 = NO नहीं
C41k	Which family planning methods did you know at that time?<br>कौनसे पता है?	1 = FEMALE STERILIZATION महिला नसबन्दी<br>2 = MALE STERILIZATION पुरूश नसबन्दी<br>3 = PILL गर्भ निरोधक गोलियाँ<br>4 = IUCD/COPPER-T आई.यू.सी.डी./लूप<br>5 = INJECTABLES इन्जेक्शन<br>6 = CONDOM/NIRODH कान्डोम या निरोध<br>7 = IMPLANTS गर्भनिरोधक प्रत्यारोपण<br>8 = FEMALE CONDOM महिला कान्डोम<br>9 = DIAPHRAGM गर्भनिरोधक टोपी<br>10 = FOAM/JELLY फोम/जैली<br>11 = RHYTHM METHOD सुरक्षित कालअवधि<br>12 = WITHDRAWAL बाहर निकालना<br>13 = LAM 6 माह तक मा द्वारा स्तनपान जो परिवार नियोजन का एक साधन भी हैं<br>14 = EMERGENCY CONTRACEPTION आपातकाल गर्भनिरोधक<br>15त्र ाज।छक।त्क व।ै डम्ज्भ्क मानक तिथि बिधि<br>88 = OTHER (SPECIFY) अन्य (स्पष्ट करें)
C41l	Why did you not opt for family planning methods then?<br>परिवार नियोजन के तरीके क्यूँ नहीं अपनाये?	1=Wanted a child at that timeउस वक़्त बच्चा चाहिए था<br>2= Husband/In-laws did not allow to use family planning methods पति/सां ससुर परिवार नियोजन के बिधि इस्तेमाल करने नहीं देते<br>3=Did not have any method available with self at that time उस वक़्त अपने पास कोई बिधि नहीं थी<br>4= Did not have money to buy the contraceptives पैसे नहीं थे खरीदने के लिए<br>88=Others अन्य

D. ANTENATAL CARE (RC1/2)

This section will be asked to category1 and category2, i.e. recently delivered mothers of 0-11 months child. Few of the questions will also be asked to category 3,mothers of 11-23 months child(indicated in the questions)

Read out to the respondent: Now, I would like to ask you some questions about when you were pregnant with [CHILD NAME].

mÙkjnkrk dk i<dj lquk;s vc e vkil ml le; d ckj e dN ç'u iNxh tc ¼cPp dk uke½ vkid iV e FkkA

D1.	During your pregnancy with [CHILD NAME], did you register the pregnancy?<br>जब (बच्चे का नाम) आपके गर्भ में था, तब क्या आपने गर्भावस्था का पंजीकरण कराया था?	1 = YES हाँ<br>0 = NO नहीं → **GO TO D5**
D2.	Whom did you register your pregnancy with for the first time?<br>आपने गर्भावस्था का पंजीकरण पहली बार किसके पास कराया था?	1 = AWW आँगनवाड़ी कार्यकर्ता<br>2 = ASHA आशा<br>3 = ANM ए.एन.एम.<br>88 = OTHERS (SPECIFY) अन्य (स्पष्ट करें)

D3.	Did you get any card (Mother and Child Protection Card) after registering the pregnancy?<br>क्या गर्भावस्था का पंजीकरण कराने के बाद आपको कोई कोई कार्ड (माँ–बच्चा संरक्षण कार्ड) मिला था?	1 = YES हाँ<br>0 = NO नहीं
D4.	In which month of pregnancy was your pregnancy registered?<br>गर्भावस्था के किस महीने में आपने गर्भावस्था का पंजीकरण कराया था?	☐ COMPLETED MONTHS पूर्ण महीने<br>99 DO NOT KNOW पता नहीं
D5.	During your pregnancy with [CHILD NAME], did you go to see anyone for antenatal checkup?<br>जब (बच्चे का नाम) आपके गर्भ में था तब क्या प्रसव–पूर्व जाँच के लिए आप किसी के पास गयी थी?	1 = YES हाँ<br>0 = NO नहीं
D6.	During your pregnancy with [CHILD NAME], did anyone come to you for antenatal checkup?<br>जब (बच्चे का नाम) आपके गर्भ में था तब क्या प्रसव–पूर्व जाँच के लिए कोई आपके पास आया था?	1 = YES हाँ<br>0 = NO नहीं
D7.	**IF D5 = 0 & D6= 0 SKIP TO D15A**<br>;fn lh5 vkj lh6 e 0 g lh16 ij tk;<br>Who all did your antenatal checkups during your pregnancy with [CHILD NAME]?<br>जब (बच्चे का नाम) आपके गर्भ में था तब आपकी प्रसव–पूर्व जाँच किस–किस ने की थी?<br><br>**PROBE : ANY OTHERS?**<br>çkc dN vkj\<br><br>**RECORD ALL MENTIONED**<br>lHkh mÙkj fy[kA	1 = GOVERNMENT DOCTOR सरकारी डाक्टर<br>2 = PRIVATE DOCTOR प्राइवेट डाक्टर<br>3 = STAFF NURSE स्टाफ नर्स<br>4 = LHV लेडी हैल्थ विजिटर (एल.एच.वी.)<br>5 = MALE HEALTH WORKER पुरुष स्वास्थ्य कर्मी<br>6 = ANM ए.एन.एम.<br>7 = OTHER HEALTH PERSONNEL अन्य स्वास्थ्य कर्मी<br>8 = ASHA आशा<br>9 = AWW आँगनवाड़ी कार्यकर्ता<br>10 = SBA/TRAINED DAI एसबीए/प्रशिक्षित दाई<br>11 = DAI दाई<br>12 = RMP आर.एम.पी. (झोला छाप डाक्टर)<br>88 = OTHER (SPECIFY) अन्य (स्पष्ट करें)
D8.	Where all did you receive antenatal checkup when you were pregnant with [CHILD NAME]?<br>जब (बच्चे का नाम) आपके गर्भ में था तब आपकी प्रसव–पूर्व जाँच कहाँ हुई थी?<br><br>**PROBE : ANY OTHERS?**<br>çkc dN vkj\<br><br>**RECORD ALL MENTIONED**<br>lHkh mÙkj fy[kA	1 = YOUR HOME अपना घर<br>2 = PARENT'S HOME माता–पिता का घर<br>3 = GOVERNMENT/MUNICIPAL HOSPITAL सरकारी/नगर निगम हस्पताल<br>4 = GOVERNMENT DISPENSARY सरकारी डिस्पेंसरी<br>5 = UHC/UHP/UFWC यूएचसी/यूएचपी/यूएफडब्ल्यूसी<br>6 = CHC सीएचसी<br>7 = APHC/NPHC/BPHC एपीएचसी/एनपीएचसी/बीपीएचसी<br>8 = SUB-CENTER उपकेन्द्र<br>9 = ANGANWADI CENTER आँगनवाड़ी केन्द्र<br>10 = VHND ग्राम स्वास्थ्य एवं पोष्ण दिवस<br>11 = NGO HOSPITAL/CLINIC गैर सरकारी संस्था का हस्पताल/क्लीनिक<br>12 = PRIVATE HOSPITAL/CLINIC प्राइवेट हस्पताल/क्लीनिक<br>88 = OTHERS (SPECIFY) अन्य (स्पष्ट करें)
D9.	When you were pregnant with [CHILD NAME], how many months pregnant were you when you received antenatal checkup for the first time?<br>जब (बच्चे का नाम) आपके गर्भ में था और आपकी पहली बार प्रसव–पूर्व जाँच हुई तब आप कितने महीने के गर्भ से थी?<br><br>**CODE IN COMPLETED MONTHS**<br>i.k eghuk e fy[kA	☐ COMPLETED MONTHS पूर्ण महीने<br>99 = DO NOT KNOW पता नहीं
D10.	**(RC3)** When you were pregnant with [CHILD NAME], how many times did you receive antenatal checkup?<br>जब (बच्चे का नाम) आपके गर्भ में था तब आपकी कितनी बार प्रसव–पूर्व जाँच हुई थी?	☐ NO. OF TIMES इतनी बार<br>99 = DO NOT KNOW पता नहीं
D11.	How many times did you receive antenatal check-up during the last three months of your pregnancy?<br>गर्भावस्था के अखरी तीन महिनों मे आपकी कितनी बार प्रसव–पूर्व जाँच हुई थी?	☐ NO. OF TIMES इतनी बार<br>0 → **GO TO D13**<br>99 = DO NOT KNOW पता नहीं
D12.	Where did you receive antenatal care during the last three months of pregnancy?<br>गर्भावस्था के अखरी तीन महिनों मे आपकी प्रसव–पूर्व जाँचें कहाँ हुई थी?	1 = YOUR HOME अपना घर<br>2 = PARENT'S HOME माता–पिता का घर

	**PROBE : ANY OTHERS?**<br>çkc % dN vkj\<br><br>**RECORD ALL MENTIONED**<br>lHkh mÙkj fy[kA	3 = GOVERNMENT/MUNICIPAL HOSPITAL सरकारी/नगर निगम हस्पताल<br>4 = GOVERNMENT DISPENSARY सरकारी डिस्पेंसरी<br>5 = UHC/UHP/UFWC यूएचसी/यूएचपी/यूएफडब्ल्यूसी<br>6 = CHC सीएचसी<br>7 = APHC/NPHC/BPHC एपीएचसी/एनपीएचसी/बीपीएचसी<br>8 = SUB-CENTER उपकेन्द्र<br>9 = ANGANWADI CENTER आँगनवाड़ी केन्द्र<br>10 = VHND ग्राम स्वास्थ्य एवं पोष्ण दिवस<br>11 = NGO HOSPITAL/CLINIC गैर सरकारी संस्था का हस्पताल/क्लीनिक<br>12 = PRIVATE HOSPITAL/CLINIC प्राइवेट हस्पताल/क्लीनिक<br>88 = OTHERS (SPECIFY) अन्य (स्पष्ट करें)

D13. **(RC3)** During your pregnancy with [CHILD NAME], as part of your antenatal check-ups were any of the following done at least once?
जब (बच्चे का नाम) आपके गर्भ में था तब क्या आपकी प्रसव–पूर्व जाँच के रूप में निम्नलिखित जाँच कम से कम एक बार की गयी थी?

READ EACH ITEM BELOW (a-h) AND CODE YES OR NO FOR EACH. IF 'NO' GO TO NEXT LETTER
¼,&,p½ rd iz'u vkj ÁR;sd ds fy, gka ;k ugha dk dksM djsA ;fn ugha gS vxys v{kj ij tk;sA

	Tests जाँचें	YES हाँ	NO नहीं	DO NOT KNOW पता नहीं
a.	Were you weighed? क्या आपका वजन लिया गया था?	1	0	99
b.	Was your blood pressure measured? क्या आपके रक्तचाप (ब्लड प्रेशर) की जाँच की गई थी?	1	0	99
c.	Did you give a urine sample? क्या आपने पिशाप का नमूना दिया था?	1	0	99
d.	Was your blood checked for hemoglobin level? हीमोग्लोबिन का स्तर जानने के लिये क्या आपके खून कि जाँच की गयी थी?	1	0	99
e.	Was your abdomen checked? क्या आपके पेट की जाँच की गई थी?	1	0	99
f.	Was your ultrasound done? क्या आपका अल्ट्रासाउंड किया गया था?	1	0	99

D14.	**ASK ONLY IF YES IN D13b**<br>rHkh iwNs ;fn **D13b** dk mÙkj gka gS(<br>During the last 3 months of your pregnancy with [CHILD NAME], was your blood pressure checked?<br>जब (बच्चे का नाम) आपके गर्भ में था, तब गर्भावस्था के अखरी तीन महिनों मे क्या आपके रक्तचाप (ब्लड प्रेशर) की जाँच की गई थी?	1 = YES हाँ<br>0 = NO नहीं<br>99 = DO NOT KNOW पता नहीं
D15.	**ASK ONLY IF YES IN D13d**<br>rHkh iwNs ;fn **D13d** dk mÙkj gka gS(<br>During the last 3 months of your pregnancy with [CHILD NAME], was your blood checked for haemoglobin level?<br>जब (बच्चे का नाम) आपके गर्भ में था, तब गर्भावस्था के अखरी तीन महिनों मे क्या आपके हीमोग्लोबिन की स्तर जानने के लिये आपके खून की जाँच की गयी थी?	1 = YES हाँ<br>0 = NO नहीं<br>99 = DO NOT KNOW पता नहीं
**D15a**	**(RC3)** During your pregnancy, were you identified as anaemic?<br>**जब आप गर्भवती थी तब क्या आपको बताया गया था के आपको खून की कमी है?**	1 = YES हाँ<br>0 = NO नहीं<br>99 = DO NOT KNOW पता नहीं
D16.	**(RC3)** During your pregnancy with [CHILD NAME], were you given a TT injection?<br>जब (बच्चे का नाम) आपके गर्भ में था तो क्या आपको टि्टनस की सुई लगी थी?	1 = YES हाँ<br>0 = NO नहीं → **GO TO D18**<br>99 = DO NOT KNOW पता नहीं → **GO TO D18**
D17.	**(RC3)** During your pregnancy with [CHILD NAME], how many times did you receive a tetanus injection?<br>जब (बच्चे का नाम) आपके गर्भ में था तब आपको कितने टि्टनस की सुई लगी थी?	☐☐ NO. OF TIMES कितनी बार<br>99 = DO NOT KNOW/REMEMBER पता नहीं/याद नहीं
D18.	**(RC3)** During your pregnancy with [CHILD NAME], did you receive any Iron Folic Acid (IFA) tablets?<br>जब (बच्चे का नाम) आपके गर्भ में था तब क्या आपको आई.एफ.ए. (ताकत की लाल गोली) की गोलियाँ मिली थी?<br><br>**SHOW SAMPLES OF IFA TABLETS**<br>vkbZ-,Q-,- dh xksfy;ksa ds ueus fn[kk;sA	1 = YES हाँ<br>0 = NO नहीं → **GO TO D22**<br>99 = DO NOT KNOW पता नहीं → **GO TO D22**
D19.	In which month of pregnancy did you receive IFA tablets for the first time?	☐ COMPLETED MONTHS पूर्ण महीने<br>99 = DO NOT KNOW पता नहीं

	गर्भावस्था के किस महीने में आपको पहली बार आई.एफ.ए. (ताकत की लाल गोली) की गोलियाँ मिली थी?	
D20.	**(RC3)** How many tablets did you receive in total during your whole pregnancy with [CHILD NAME]?<br>जब (बच्चे का नाम) आपके गर्भ में था तब आपको आई.एफ.ए. (ताकत की लाल गोली) की कितनी गोलियाँ मिली थी?<br><br>**PROBE FOR WHETHER TABLETS ARE ADULT (LARGE) OR PEDIATRIC (SMALL) DOSE.**<br>iN: fd ;g xkfy;k cMh Fkh ;k NkVh FkhA	☐☐☐ NUMBER OF LARGE TABLETS बड़ी गोलियों की संख्या<br>☐☐☐ NUMBER OF SMALL TABLETSछोटी गोलियों की संख्या<br>999 = DO NOT REMEMBER याद नहीं
D21.	**(RC3)** During the whole pregnancy with [CHILD NAME], how many tablets did you consume?<br>जब (बच्चे का नाम) आपके गर्भ में था तब आपने आई.एफ.ए. (ताकत की लाल गोली) की कितनी गोलियाँ खायी थी?	☐☐☐ NUMBER OF LARGE TABLETS बड़ी गोलियों की संख्या<br>☐☐☐ NUMBER OF SMALL TABLETSछोटी गोलियों की संख्या<br>999 = DO NOT REMEMBER याद नहीं
D22.	**(RC3)** During your pregnancy with [CHILD NAME], were you given or did you buy any iron folic syrup?<br>जब (बच्चे का नाम) आपके गर्भ में था, क्या आपको आईरन फोलिक एसिड (ताकत) की सिरप मिली थी या आपने खरीदी थी?	1 = YES हाँ<br>0 = NO नहीं SKIP to D23
**D22a**	**(RC3)** In which month of pregnancy did you receive IFA syrup for the first time?<br>गर्भावस्था के किस महीने में आपको पहली बार आई.एफ.ए. सिरप मिली थी?	☐ COMPLETED MONTHS पूर्ण महीने<br>99 = DO NOT KNOW पता नहीं
**D22 b**	**(RC3)** How many did you receive in total during your whole pregnancy with [CHILD NAME]?<br>जब (बच्चे का नाम) आपके गर्भ में था तब आपको आई.एफ.ए. की कितनी बोतलें मिली थी?	☐☐☐ NUMBER<br>999 = DO NOT REMEMBER याद नहीं
**D22c**	**(RC3)** During the whole pregnancy with [CHILD NAME], how many did you consume?<br>जब (बच्चे का नाम) आपके गर्भ में था तब आपने आई.एफ.ए. की कितनी बोतलें खायी थी?	☐☐☐ NUMBER<br>999 = DO NOT REMEMBER याद नहीं
D23.	**ASK IF D21>0 OR D22=1, ELSE GO TO D23A**<br>**(RC3)** During the whole pregnancy with [CHILD NAME], for how many days did you take the tablets or syrup?<br>जब (बच्चे का नाम) आपके गर्भ में था, पूरी गर्भावस्थ के दौरान आपने कितने दिनों तक आईरन फोलिक एसिड (ताकत) की गोली या सिरप खायी थी?<br><br>**IF ANSWER IS NOT NUMERIC, PROBE FOR APPROXIMATE NUMBER OF DAYS.**<br>;fn mÙkj l[;k e ugh g] iN fd fnuk dh l[;k yxHkx D;k FkhA	☐☐☐ NO OF DAYS FOR LARGE TABLETS बड़ी गोलियाँ खाने के दिनों की संख्या<br>☐☐☐ NO OF DAYS FOR SMALL TABLETS छोटी गोलियां दिनों की संख्या<br>☐☐☐ NO OF DAYS FOR SYRUP सिरप खाने के दिनों की संख्या<br>999 DO NOT REMEMBER याद नहीं
**D24 C**	**(RC3)** During your pregnancy with [CHILD NAME], were you given or did you buy calcium tablets?<br>जब (बच्चे का नाम) आपके गर्भ में था, क्या आपको केलसियम की गोली मिली थी या आपने खरीदी थी?	1 = YES हाँ<br>0 = NO नहीं → **GO TO D24F**
**D24 D**	**(RC3)** During your pregnancy with [CHILD NAME], how many calcium tablets were you given or did you buy?<br>जब (बच्चे का नाम) आपके गर्भ में था, आपको केलसियम की कितनी गोली मिली थी या आपने खरीदी थी?	☐☐☐ NUMBER OF TABLETS गोलियों की संख्या<br>999 DO NOT REMEMBER याद नहीं
**D24 E**	**(RC3)** During the whole pregnancy with [CHILD NAME], how many tablets did you consume?<br>जब (बच्चे का नाम) आपके गर्भ में था तब आपने केलसियम की कितनी गोलियाँ खायी थी?	☐☐☐ NUMBER OF TABLETS गोलियों की संख्या<br>999 DO NOT REMEMBER याद नहीं
**D24 F**	**ASK IF D24E>0 ELSE GO TO D24G**<br>**(RC3)** During the whole pregnancy with [CHILD NAME], for how many days did you take the tablets?<br>जब (बच्चे का नाम) आपके गर्भ में था, पूरी गर्भावस्थ के दौरान आपने कितने दिनों तक गोली खायी थी?<br><br>**IF ANSWER IS NOT NUMERIC, PROBE FOR APPROXIMATE NUMBER OF DAYS.**<br>;fn mÙkj l[;k e ugh g] iN fd fnuk dh l[;k yxHkx D;k FkhA	☐☐☐ NO OF DAYS FOR TABLETS गोलियाँ खाने के दिनों की संख्या<br>999 = DO NOT REMEMBER याद नहीं

D25.	During your pregnancy with [CHILD NAME], did you ever receive any Take Home Ration from the Anganwadi Center?	1 = YES हाँ<br>0 = NO नहीं GOTO D26

	जब (बच्चे का नाम) आपके गर्भ में था तब क्या आपको कभी भी आँगनवाड़ी केन्द्र से पंजीरी मिली थी?	
**D25A**	For how long did you receive THR during your pregnancy?<br>गर्भवस्था के दौरान आपको कितने समय तक पुरत आहार मिला था?	☐ ☐☐<br>1 = WEEKS सप्ताह<br>2 = MONTHS महीने<br>999=DO NOT REMEMBER याद नहीं
D26.	During your pregnancy with [CHILD NAME], did you take rest more frequently than when you were not pregnant?<br>जब (बच्चे का नाम) आपके गर्भ में था तब क्या आप गर्भवती न होने के समय की तुलना में अधिक बार आराम करती थी?	1 = YES हाँ<br>0 = NO नहीं
D27.	During your pregnancy with [CHILD NAME] did you consume more food as compared to when you were not pregnant?<br>जब (बच्चे का नाम) आपके गर्भ में था तब क्या आप गर्भवती न होने के समय की तुलना में अधिक भोजन करती थी?	1 = YES हाँ<br>0 = NO नहीं
**D27a**	Which of the following foods did you consume almost daily during pregnancy?<br><br>कौनसे पढके सुनाई जाने वाली चीज़ें आपने रोजाना गर्भवस्था के दौरान खाया?<br><br>MULTIPLE CHOICE	1= Dark green leafy vegetables हरि सब्जियां<br>2=Yellow or orange fruits or vegetables (e.g. Pumpkin, carrots, ripe mango, ripe papaya) पीली या नारंगी फल और सब्जी<br>3=Lentils, dal दल<br>4=Roti, bread or rice रोटी, चावल, ब्रेड<br>5=Eggs अन्दा<br>6=Meat, chicken or fish मॉस<br>7=Milk, paneer or yogurt दूध, पनीर, दही<br>88=Othersअन्य
**D27c**	How many kgs. did you gain during pregnancy?<br>गर्भवस्था के दौरान कितना वजन बढ़ा?	__ KGS<br>99 = DO NOT KNOW पता नहीं
D28.	During your pregnancy with [CHILD NAME], did you avoid picking up heavy objects?<br>जब (बच्चे का नाम) आपके गर्भ में था तब क्या आप भारी सामान उठाने से बचती थी?	1 = YES हाँ<br>0 = NO नहीं
Read out to the respondent: *Now, I would ask you about awareness of different danger signs during pregnancy and complications that a woman may experience during her pregnancy.*<br>mÙkjnkrk dk i<dj lquk;h vc eSa vki ls xHkkZoLFkk ds nkSjku [krjs ds fofHkUu y{k.kksa vkSj tfVyrkvksa dh tkx:drk ds ckjs esa iwNuk pkgwxh] tks ,d efgyk xHkkZoLFkk ds nkSjku vuqHko dj ldrh g\		
D29.	Are you aware about the different danger signs during pregnancy which require visit to a health facility or contacting a healthcare provider?<br>क्या आप गर्भावस्था के दौरान होने वाले खतरे के विभिन्न लक्षणों के बारे में जानती हैं जिनके लिए स्वास्थ्य केंद्र जाने की या स्वास्थ्य प्रदाता से संपर्क करने की जरूरत होती है?	1 = YES हाँ<br>0 = NO नहीं → **GO TO D32**
D30.	What are the different danger signs during pregnancy which require visit to a health facility or contacting a healthcare provider?<br>गर्भावस्था के दौरान खतरे के वह कौन से लक्षण हैं जिनके लिए स्वास्थ्य केंद्र जाने की या स्वास्थ्य प्रदाता से संपर्क करने की जरूरत होती है?<br><br>**PROBE : ANY OTHERS?**<br>çkcs dqN vkSj\<br><br>**RECORD ALL MENTIONED**<br>lHkh mÙkj fy[kA	1 = EXCESSIVE VAGINAL BLEEDING योनि से अत्यधिक खून निकलना<br>2 = DIFFICULTY IN BREATHING सांस लेन में कठिनाई<br>3 = SEVERE HEADACHE तेज सिर दर्द<br>4 = BLURRED VISION धुंधला दिखना<br>5 = SWELLING OF THE HANDS, FEET, BODY OR FACE हाथों, पैरों, शरीर और चेहरे का सूजन<br>6 = HIGH FEVER तेज बुखार<br>7 = LOSS OF CONSCIOUSNESS होश खोना<br>8 = SEVERE ABDOMINAL PAIN पेट में तेज दर्द<br>9 = CONVULSIONS दौरे पड़ना<br>10 = HIGH BLOOD PRESSURE उच्च रक्तचाप<br>88 = OTHERS (SPECIFY) अन्य (स्पष्ट करें)
D31.	From where did you get the information about these danger signs during pregnancy?<br>गर्भावस्था के दौरान खतरे के लक्षणों के बारे में आपको कहाँ से जानकारी मिली थी?<br><br>**PROBE : ANY OTHERS?**<br>çkcs dqN vkSj\	1 = GOVERNMENT DOCTOR सरकारी डाक्टर<br>2 = PRIVATE DOCTOR प्राइवेट डाक्टर<br>3 = STAFF NURSE स्टाफ नर्स<br>4 = LHV लेडी हैल्थ विजिटर (एल.एच.वी.)<br>5 = MALE HEALTH WORKER पुरुष स्वास्थ्य कर्मी<br>6 = ANM ए.एन.एम.

<table>
<tr><td></td><td>RECORD ALL MENTIONED
lHkh mÙkj fy[kA</td><td colspan="3">7 = OTHER HEALTH PERSONNEL अन्य स्वास्थ्य कर्मी
8 = ASHA आशा
9 = AWW आँगनवाड़ी कार्यकर्ता
10 = SBA/TRAINED DAI एसबीए/प्रशिक्षित दाई
11 = DAI दाई
12 = RMP आर.एम.पी. (झोला छाप डाक्टर)
13 = FAMILY MEMBER परिवार के सदस्य
14 = FRIENDS/NEIGHBOUR दोस्त/पड़ौसी
15 = RADIO/TV/NEWS PAPER रेडियो/टेलिविजन/अखबार
16 = MOBILE SMS मोबाइल एसएमएस
88 = OTHERS (SPECIFY) अन्य (स्पष्ट करें)
98 = NO ONE कोई नहीं
99 = DO NOT KNOW पता नहीं</td></tr>
<tr><td>D32.</td><td>During your pregnancy were you told to visit a doctor/facility in case of any pregnancy complications?
जब (बच्चे का नाम) आपके गर्भ मे था तब क्या आपको यह बताया गया था कि गर्भावस्था से संबंधित किसी भी परेशानी के मामले में डाक्टर के पास/स्वास्थ्य केंद्र पर जायें?</td><td colspan="3">1 = YES हाँ
0 = NO नहीं</td></tr>
<tr><td>D33.</td><td>During your pregnancy what kind of problems did you experience?
इस गर्भवस्था के दौरान आपको किस प्रकार की परेशानी हुई है?

DO NOT READ LIST. CODE ALL MENTIONS IN FIRST COLUMN. THEN READ EACH ITEM BELOW (a-j) THAT WASN'T MENTIONED AND CODE RESPONSE IN SECOND COLUMN. IF NOT MENTIONED, SKIP TO NEXT LETTER.
lph dk u i<A igy dkye e lHkh mYy[k fd; x; mÙkjk dk dkM djA bld ckn mu fcUnvk ¼,&t½ dk i< ftudk mYy[k ugh fd;k x;k Fkk vkj nlj dkye e mÙkj dk dkM djA ;fn ugh crk;k x;k vxy fcn ij tk;A</td><td>1 = YES, SPONTANEOUS
हाँ, स्वयं बताया</td><td>2 = YES, AFTER READING
हाँ, पढ़ने के बाद</td><td>0 = NO
नहीं</td></tr>
<tr><td>a.</td><td>Excessive vaginal bleeding योनी से अत्यधिक रक्तस्राव</td><td>1</td><td>2</td><td>0</td></tr>
<tr><td>b.</td><td>Smelly vaginal discharge योनि से बदबूदार पानी निकलना</td><td>1</td><td>2</td><td>0</td></tr>
<tr><td>c.</td><td>Swelling of the hands, feet, body or face हाथों, पैरों, शरीर और चेहरे पर सूजन</td><td>1</td><td>2</td><td>0</td></tr>
<tr><td>d.</td><td>Headache सिर दर्द</td><td>1</td><td>2</td><td>0</td></tr>
<tr><td>e.</td><td>Blurred vision धुंधला दिखना</td><td>1</td><td>2</td><td>0</td></tr>
<tr><td>f.</td><td>Convulsions दौरे पड़ना</td><td>1</td><td>2</td><td>0</td></tr>
<tr><td>g.</td><td>Febrile illness बुखार जिसका कारण पता नहीं</td><td>1</td><td>2</td><td>0</td></tr>
<tr><td>h.</td><td>Severe abdominal pain that is not labor pain पेट में तेज दर्द जो प्रसव पीड़ा नहीं थी</td><td>1</td><td>2</td><td>0</td></tr>
<tr><td>i.</td><td>Pallor चेहरे का फीकापन</td><td>1</td><td>2</td><td>0</td></tr>
<tr><td>j.</td><td>Difficulty in breathing साँस लेन में कठिनाई</td><td>1</td><td>2</td><td>0</td></tr>
<tr><td>k.</td><td>Swelling of the hands, feet, body or face हाथों, पैरों, शरीर और चेहरे पर सूजन</td><td>1</td><td>2</td><td>0</td></tr>
<tr><td>l.</td><td>High fever तेज बुखार</td><td>1</td><td>2</td><td>0</td></tr>
<tr><td>m.</td><td>Loss of consciousness चेतना (होश) खोना</td><td>1</td><td>2</td><td>0</td></tr>
<tr><td>n.</td><td>High Blood Pressure उच्च रक्तचाप</td><td>1</td><td>2</td><td>0</td></tr>
<tr><td>o.</td><td>Heart disease दिल की बीमारी</td><td>1</td><td>2</td><td>0</td></tr>
<tr><td>p.</td><td>Diabetes मधुमेय</td><td>1</td><td>2</td><td>0</td></tr>
<tr><td>q.</td><td>Other medically diagnosed disease कोई बीमारी जो चिकित्सक द्वारा बतायी गयी है</td><td>1</td><td>2</td><td>0</td></tr>
<tr><td>D34.</td><td>ASK ONLY IF ANY OF THE RESPONSES IN D33(a-q) IS CODED 1 OR 2q
rHkh iN tc D33(a-q) e fn; x; fdlh mÙkj dk dkM 1 g

Did you seek treatment for this problem?
क्या आपने इस समस्या का इलाज कराया था?</td><td colspan="3">1 = YES हाँ
0 = NO नहीं → GO TO D36</td></tr>
<tr><td>D35.</td><td>From where did you seek treatment?
आपने इलाज कहाँ से कराया था?</td><td colspan="3">1 = GOVERNMENT/MUNICIPAL HOSPITAL सरकारी/नगरपालिका का अस्पताल</td></tr>
</table>

	**PROBE : ANY OTHERS?**<br>çkc % dqN vkj\<br><br>**RECORD ALL MENTIONED**<br>lHkh mÙkj fy[ksA	2 = GOVERNMENT DISPENSARY सरकारी दवाखाना<br>3 = UHC/UHP/UFWC यूएचसी / यूएचपी / यूएफडब्ल्यूसी<br>4 = CHC सीएचसी<br>5 = APHC/NPHC/BPHC एपीएचसी / एनपीएचसी / बीपीएचसी<br>6 = SUB-CENTER उपकेन्द्र<br>7 = ANGANWADI CENTER आँगनवाड़ी केन्द्र<br>8 = VHND ग्राम स्वास्थ्य एवं पोषण दिवस<br>9 = NGO HOSPITAL/CLINIC गैर सरकारी संस्था का अस्पताल / क्लीनिक<br>10 = PRIVATE HOSPITAL/CLINIC प्राइवेट अस्पताल / क्लीनिक<br>11 = MEDICINE SHOP दवा की दुकान<br>12 = FOLK HEALER ओझा / वैद्य / झाड़फूँक<br>13 = HOME REMEDIES घरेलू उपचार<br>14 = RMP आर.एम.पी. (झोला छाप डाक्टर)<br>88 = OTHERS (SPECIFY) अन्य (स्पष्ट करें)		
Read out to the respondent: Now, I would like to ask you about preparations or decisions you made for the delivery of [CHILD NAME].<br>mÙkjnkrk dks i<+dj lqukus;s% vc eSa vkils ¼cPps dk uke½ ds tUe ds ckjs esa dh xbZ rS;kfj;ksa vkSj fy;s x;s QSlyksa ds ckjs esa iNuk pkgwaxhA				
D36.	Prior to the delivery, did you plan or intend to deliver [CHILD NAME] at home or in a health facility?<br>प्रसव से पूर्व आपने (बच्चे का नाम) को कहाँ जन्म देने का सोचा था – घर पर या स्वास्थ्य केन्द्र मे?	1 = AT HOME घर पर<br>2 = IN A HEALTH FACILITY स्वास्थ्य केंद्र में<br>3 = DID NOT PLAN योजना नहीं बनाई		
D37.	Did you discuss plans for your delivery with [CHILD NAME's] father?<br>क्या आपने प्रसव के संबंध में अपनी योजनाओं के बारे में (बच्चे का नाम) के पिता से विचार–विमर्श किया था?	1 = YES हाँ<br>0 = NO नहीं<br>9 = NOT APPLICABLE लागू नहीं		
D38.	Did you discuss your plans for your delivery with your mother-in-law?<br>क्या आपने प्रसव के संबंध में अपनी योजनाओं के बारे में अपनी सास से विचार–विमर्श किया था?	1 = YES हाँ<br>0 = NO नहीं<br>9 = NOT APPLICABLE लागू नहीं		
D39.	Now I would like to ask you about anything specific that you did to prepare for a delivery. Did you do anything specific to prepare for delivery?<br>अब मैं आपसे प्रसव के लिए आपके द्वारा की गई विशेष तैयारी के संबंध में बारे में पूछना चाहूँगी। क्या अपने प्रसव के लिए तैयारी के संबंध में कुछ विशेष किया था?<br><br>**DO NOT READ LIST. CODE ALL MENTIONS IN FIRST COLUMN. THEN READ EACH ITEM BELOW (a-l) THAT WASN'T MENTIONED AND CODE RESPONSE IN SECOND COLUMN. IF NOT MENTIONED, SKIP TO NEXT LETTER.**<br>lwph dks u i<+sA igys dkye esa lHkh mYysf[k fd;s x;s mÙkjksa dks dksM djsA blds ckn uhps fcUnqvksa ¼,&,y½ dks i<+s ftudk mYys[k ugha fd;k x;k Fkk vkSj nqljs dkye esa mÙkj dks dksM djsA ;fn ugha crk;k x;k vxys fcanq ij tk;sA	1 = YES, SPONTANEOUS<br>हाँ, स्वयं बताया	2 = YES, AFTER READING<br>हाँ, पढ़ने के बाद	0 = DID NOT RECEIVE ANY ADVICE<br>कोई सलाह नहीं मिली
a.	Identify and arrange for a skilled birth attendant, such as an ANM, DAI to be present during childbirth<br>प्रसव कराने वाले व्यक्ति की पहचान करना या इंतजाम करना, जैसे कि ए.एन.एम., दाई जो बच्चे के जन्म के समय उपस्थित रहे	1	2	0
b.	Obtain a new blade to cut the cord<br>नाल को काटने के लिए नया ब्लेड लाना	1	2	0
c.	Obtain a new/clean thread to tie the cord<br>नाल को बांधने के लिए नया / साफ धागा लाना	1	2	0
d.	Obtain clean cloth for drying the baby<br>शिशु को पोंछने के लिए साफ कपड़ा लाना	1	2	0
e.	Obtain clean cloth for wrapping the baby<br>शिशु को लपेटने के लिए साफ कपड़ा लाना	1	2	0
f.	Save money for the delivery<br>प्रसव के लिए पैसा बचाना	1	2	0
g.	Identify a health facility to go to in case of an emergency<br>ऐसी स्वास्थ्य सुविधा की पहचान करना जहाँ आपात्कालीन स्थिति में जाया जा सके	1	2	0

h.	Identify in advance a vehicle you would use to reach health facility for delivery or in case of emergency<br>प्रसव के समय या आपात्कालीन स्थिति मे स्वास्थ्य सुविधा जाने के लिए वाहन की पहचान करना	1	2	0
i.	Kept important phone numbers handy, like the phone numbers of the ASHA, hospital, and ambulance<br>आशा, अस्पताल या एंबुलेंस जैसे महत्वपूर्ण फोन नम्बर अपने पास रखना	1	2	0
j.	Identify a person to accompany you to the healthcare facility<br>ऐसे व्यक्ति की पहचान करना जो आपके साथ स्वास्थ्य केंद्र जा सके	1	2	0
k.	Identifying a person with the similar blood group as the woman<br>ऐसे व्यक्ति की पहचान करना जिसका ब्लड ग्रुप और महिला का ब्लड ग्रुप समान हो	1	2	0
l.	Ensure soap is available for the person conducting the delivery<br>सुनिश्चित करना कि प्रसव कराने वाले के लिए साबुन उपलब्ध है	1	2	0
Read out to the respondent Now I'd like to talk to you in more detail about contacts you had with health service providers such as ASHAs, AWWs, ANMs, LHVs and Government doctors either at home<br>mÙkjnkrk dk i<dj lquk;s vc eSa vkils vkids }kjk ?kj ij ;k vU; dgh vk'kk] vkxuokMh dk;Zdrk] ,-,u-,e-] ,y,poh ;k ljdkjh MkDVj tSls LokLF; lsok çnkrkvk dk lkFk dh xbZ HkkVka dk foojk.k tkuuk pkgxhA				
D40.	**(RC3)** Did the ASHA come to meet you at your home during your pregnancy with [CHILD NAME]?<br>जब (बच्चे का नाम) आपके गर्भ में था, तब क्या आशा आपके घर आपसे मिलने आई थी?	1 = YES हाँ<br>0 = NO नहीं → **GO TO D43**		
**D40A**	How many times did the ASHA come to meet you at your home during the pregnancy with [CHILD NAME]?<br>जब (बच्चे का नाम) आपके गर्भ में था, तब आशा आपके घर आपसे मिलने कितनी बार आई थी?	☐☐ NO. OF TIMES इतनी बार<br>99 = DO NOT REMEMBER याद नहीं		
D41.	Did the ASHA come to meet you at your home during the last three months of your pregnancy with [CHILD NAME]?<br>जब (बच्चे का नाम) आपके गर्भ में था, तब क्या गर्भावस्था के आखरी तीन महिनों में आशा आपके घर आपसे मिलने आई थी?	1 = YES हाँ<br>0 = NO नहीं → **GO TO D42A**		
D42.	How many times did the ASHA come to meet you at your home during the last three months of your pregnancy with [CHILD NAME]?<br>जब (बच्चे का नाम) आपके गर्भ में था, तब गर्भावस्था के आखरी तीन महिनों में आशा आपके घर आपसे मिलने कितनी बार आई थी?	☐☐ NO. OF TIMES इतनी बार<br>99 DO NOT REMEMBER याद नहीं		
D43.	**(RC3)** Did the AWW come to meet you at your home during your pregnancy with [CHILD NAME]?<br>जब (बच्चे का नाम) आपके गर्भ में था तब क्या आँगनवाड़ी कार्यकर्ता आपके घर आपसे मिलने आई थी?	1 = YES हाँ<br>0 = NO नहीं → **GO TO** D46		
**D43A**	How many times did the AWW come to meet you at your home during the pregnancy with [CHILD NAME]?<br>जब (बच्चे का नाम) आपके गर्भ में था, तब आँगनवाड़ी कार्यकर्ता आपके घर आपसे मिलने कितनी बार आई थी?	☐☐ NO. OF TIMES इतनी बार<br>99 = DO NOT REMEMBER याद नहीं		
D44.	Did the AWW come to meet you at your home during the last three months of your pregnancy with [CHILD NAME]?<br>जब (बच्चे का नाम) आपके गर्भ में था, तब क्या गर्भावस्था के आखरी तीन महिनों में आँगनवाड़ी कार्यकर्ता आपके घर आपसे मिलने आई थी?	1 = YES हाँ<br>0 = NO नहीं → **GO TO** D45A		
D45.	How many times did the AWW come to meet you at your home during the last three months of your pregnancy with [CHILD NAME]?<br>जब (बच्चे का नाम) आपके गर्भ में था, तब गर्भावस्था के आखरी तीन महिनों में आँगनवाड़ी कार्यकर्ता आपके घर आपसे मिलने कितनी बार आई थी?	☐☐ NO. OF TIMES इतनी बार<br>99 DO NOT REMEMBER याद नहीं		
D46.	**(RC3)** Did the ANM come to meet you at your home during your pregnancy with [CHILD NAME]?<br>जब (बच्चे का नाम) आपके गर्भ में था तब क्या ए.एन.एम. आपके घर आपसे मिलने आई थी?	1 = YES हाँ<br>0 = NO नहीं → **GO TO D49**		
**D46A**	How many times did the ANM come to meet you at your home during the pregnancy with [CHILD NAME]?<br>जब (बच्चे का नाम) आपके गर्भ में था, तब ए.एन.एम. आपके घर आपसे मिलने कितनी बार आई थी?	☐☐ NO. OF TIMES इतनी बार<br>99 = DO NOT REMEMBER याद नहीं		
D47.	Did the ANM come to meet you at your home during the last three months of your pregnancy with [CHILD NAME]?<br>जब (बच्चे का नाम) आपके गर्भ में था, तब क्या गर्भावस्था के आखरी तीन महिनों में ए.एन.एम. आपके घर आपसे मिलने आई थी?	1 = YES हाँ<br>0 = NO नहीं → **GO TO D48a**		

D48.	How many times did the ANM come to meet you at your home during the last three months of your pregnancy with [CHILD NAME]?<br>जब (बच्चे का नाम) आपके गर्भ में था, तब गर्भावस्था के आखरी तीन महिनों में ए.एन.एम. आपके घर आपसे मिलने कितनी बार आई थी?	☐☐ NO. OF TIMES इतनी बार<br>99 DO NOT REMEMBER याद नहीं

D49.	**(RC3)** Did the LHV/Government Doctor come to meet you at your home during your pregnancy with [CHILD NAME]?<br>जब (बच्चे का नाम) आपके गर्भ में था तब क्या एल.एच.वी./सरकारी डाक्टर आपके घर आपसे मिलने आये थे?	1 = YES हाँ<br>0 = NO नहीं → **GO TO D52**
D50.	Did the LHV/Government Doctor come to meet you at your home during the last three months of your pregnancy with [CHILD NAME]?<br>जब (बच्चे का नाम) आपके गर्भ में था, तब क्या गर्भावस्था के आखरी तीन महिनों में एल.एच.वी./सरकारी डाक्टर आपके घर आपसे मिलने आये थे?	1 = YES हाँ<br>0 = NO नहीं → **GO TO D52**
D51.	How many times did the LHV/Government Doctor come to meet you at your home during the last three months of your pregnancy with [CHILD NAME]?<br>जब (बच्चे का नाम) आपके गर्भ में था, तब गर्भावस्था के आखरी तीन महिनों में एल.एच.वी./सरकारी डाक्टर आपके घर आपसे मिलने कितनी बार आये थे?	1 = YES हाँ<br>0 = NO नहीं
D52.	During your pregnancy with [CHILD NAME], did anyone tell you about your expected delivery date?<br>जब (बच्चे का नाम) आपके गर्भ में था, तब क्या किसी ने आपको आपकी प्रसव की संभावित तिथि बतायी थी?	1 = YES हाँ<br>0 = NO नहीं

D53.	During your pregnancy with [CHILD NAME], what advice did you receive from ASHA/AWW/ANM related to your pregnancy/delivery during home visits?<br>जब (बच्चे का नाम) आपके गर्भ में था तब आपको आपकी गर्भावस्था/प्रसव के बारे में आशा/आँगनवाड़ी कार्यकर्ता/ए.एन.एम. ने से क्या सलाह मिली थी?<br><br>**DO NOT READ LIST. CODE ALL MENTIONS IN FIRST COLUMN. THEN READ EACH ITEM BELOW (a-p) THAT WASN'T MENTIONED AND CODE RESPONSE IN SECOND COLUMN. IF NOT MENTIONED, SKIP TO NEXT LETTER.**<br>lwph dks u i<sA igys dkye esa lHkh mYys[k fd;s x;s mÙkjksa dks dkM djsA blds ckn mu fcUnqvksa ¼,&ih dks i<sa ftudk mYys[k ugha fd;k x;k Fkk vkSj nwljs dkye esa mÙkj dk dksM djsA ;fn ugha crk;k x;k rc vxys fcUnq ij tk;sA<br><br>**Note to investigator: Please note that these are focused on advice on what the woman needs to do to take care of her health during pregnancy and prepare for delivery. Tell the woman you will ask about advice on caring for her child next.**<br>bofLVxsVj dks fy, fVIi.kh% d`i;k /;ku nsa fd budk eq[; lac/k xHkkZ/kkj.k ds nkSjku vkSj Álo dh rS;kjh ds le; viuh LokLF; dh ns[kjs[k gsrq efgyk ds fy, tks vko';d gS mlls lacf/kr lykg ls gSA mÙkjnkrk dks crk;sa fd blds ckn vki mlls cPps dh ns[kjs[k lac/kh lykg ds ckjs esa iwNsxsA	1 = YES, SPONTANEOUS<br>हाँ, स्वयं बताया	2 = YES, AFTER READING<br>हाँ, पढ़ने के बाद	0 = DID NOT RECEIVE ANY ADVICE<br>कोई सलाह नहीं मिली
a.	Taking rest आराम करना	1	2	0
b.	Eating more/healthy food अधिक/स्वास्थ्यकारी भोजन करना	1	2	0
b1	Dietary diversity आहार बिबीधता	1	2	0
B1 a	Quantity of food to consume खाने की मात्रअ	1	2	0
B1 b	Weight to gain कितना वजन बढ़ना	1	2	0
c.	Must undergo at least 4 ANC check-ups कम से कम 4 प्रसव–पूर्व जांच कराना	1	2	0
d.	Must take TT injections ट्टिनस के टीके अवश्य लगाना	1	2	0
e.				
E1	Must consume 180 IFA tablets during pregnancy १८० ifa की गोली लेना	1	2	0

f.	Need of IFA (Iron) tablets during pregnancy गर्भावस्था के दौरान आई.एफ.ए. की गोलियों की जरूरत	1	2	0
F1	Need of IFA (Iron) tablets after delivery प्रसव के बाद आई.एफ.ए. की गोलियों की जरूरत	1	2	0
F2.	Problems that you may face due to consuming the IFA tablets and syrup आई.एफ.ए. (ताकत की लाल गोली) की गोलियाँ खाने से होने वाली समस्याओं के बारे में	1	2	0
g.	Must consume calcium tablets केलसियम की गोलियाँ अवश्य लेना	1	2	0
h.	Need of calcium tablets during pregnancy गर्भावस्था के दौरान केलसियम की गोलियों की जरूरत	1	2	0
i.	Need of calcium tablets after delivery प्रसव के बाद केलसियम की गोलियों की जरूरत	1	2	0
I1	Must consume 180 calcium tablets during pregnancy गर्भवस्था के दौरान १८० कैल्शियम की गोलिया अवश्य लेना	1	2	0
j.	Planning for a skilled birth attendant to be present if planning a home delivery यदि घर पर प्रसव कराने की योजना है तब कुशल जन्म सहायक की उपस्थिति की योजना बनाना	1	2	0
k.	Obtaining a new blade for delivery प्रसव के लिए नया ब्लेड लाना	1	2	0
l.	Obtaining a new thread for delivery प्रसव के लिए नया धागा लाना	1	2	0
m.	Saving money for potential complications संभावित जटिलताओं या समस्याओं के लिए पैसे की बचत	1	2	0
n.	Delivering in a health facility स्वास्थ्य सुविधा में प्रसव कराना	1	2	0
o.	Identifying transportation to go to health facility स्वास्थ्य सुविधा जाने के लिए वाहन की पहचान करना	1	2	0
p.	Kept important phone numbers handy, like the phone numbers of the ASHA, hospital, and ambulance आशा, अस्पताल या एंबुलेंस जैसे महत्वपूर्ण फोन नम्बर अपने पास रखना	1	2	0
q.	Staying 48 hours in the health facility after delivery प्रसव के बाद 48 घंटे तक स्वास्थ्य सुविधा में रहना	1	2	0
r.	Information on the danger of excessive vaginal bleeding, foul vaginal discharge, abdominal pain, or fever after delivery योनि से अत्यधिक खून निकलने, योनि से बदबूदार पानी निकलने, पेट में दर्द या प्रसव के बाद बुखार आदि के बारे में जानकारी	1	2	0
s.	Identifying a facility to go to in case of an emergency आपात्कालीन स्थिति होने पर जिस स्वास्थ्य सुविधा में जाना है उसकी पहचान करना	1	2	0
D54.	During your pregnancy with [CHILD NAME], what advice did you receive on caring for your child from ASHA/AWW/ANM ?<br>जब (बच्चे का नाम) आपके गर्भ में था तब आशा /अनंवादी कार्यकर्ता/ ऐ एन एम् से आपको बच्चे की देखरेख के बारे में क्या सलाह मिली थी?<br><br>**DO NOT READ LIST. CODE ALL MENTIONS IN FIRST COLUMN. THEN READ EACH ITEM BELOW (a-i) THAT WASN'T MENTIONED AND CODE RESPONSE IN SECOND COLUMN. IF NOT MENTIONED, SKIP TO NEXT LETTER.**<br>lwph dk u i<A igys dkye es lHkh mYys[k fd;s x;s mÙkjks dk dkM djsA blds ckn mu fcUnqvks ¼,&vkb½ dks i<s ftudk mYys[k ugha fd;k x;k Fkk vkSj nwljs dkye es mÙkj dk dkM djsA ;fn ugha crk;k x;k rc vxys fcUnq ij tk;sA	1 = YES, SPONTANEOUS<br>हाँ, स्वयं बताया	2 = YES, AFTER READING<br>हाँ, पढ़ने के बाद	0 = DID NOT RECEIVE ANY ADVICE<br>कोई सलाह नहीं मिली
a.	Not applying anything on the cord नाल काटने के बाद उसपर कुछ नहीं लगाना	1	2	0
b.	How to keep the baby warm बच्चे को गर्म रखना	1	2	0
c.	Skin to skin contact शरीर से सटा कर रखना	1	2	0
d.	Not bathing the baby within 3 days after birth in case of normal baby सामान्य प्रसव के बाद तीन दिनों तक बच्चे को न नहलाना	1	2	0
e.	Not bathing the baby within 7 days after birth in case of low birth weight baby यदि बच्चा आपरेशन से हुआ है सात दिनों तक बच्चे को न नहलाना	1	2	0
f.	Putting baby to breast within one hour after delivery प्रसव के बाद 1 घंटे के अन्दर बच्चे को स्तनपान कराना	1	2	0

g.	Not feeding the baby honey, janam gutti, etc., before breastfeeding for the first time पहली बार स्तनपान कराने से पहले बच्चे को शहद, जन्म घुट्टी आदि न देना	1	2	0
G1	Not feeding the baby honey, janam gutti etc during the first 3 days after delivery<br>बच्चे को घुट्टी, शहद वगेरा जन्म के पहले 3 दिन में नहीं पिलाना	1	2	0
h.	Exclusive breastfeeding till 6 months 6 महीनों तक केवल स्तनपान कराना	1	2	0
i.	Information on the danger signs of the baby like - having trouble breathing, being difficult to wake up, losing interest in breastfeeding, or being cold to the touch<br>बच्चे के खतरे के संकेतों के बारे में जानकारी, जैसे कि सांस लेने में दिक्कत, बच्चे को उठाने मे कठिनाई, स्तनपान में बच्चे की दिलचस्पी न हो, छूने पर उसका शरीर ठंडा लगना	1	2	0
j.	Immunisation of the child बच्चे का टीकाकरण	1	2	0
D54A	**(RC3)** After the delivery, how much iron folic acid tablets and syrups bottles did you receive or purchase?<br><br>प्रसव के बाद आपको कितने IFA की गोलीया और IFA की सिरप की बोतलें मिली थी?	Large Tablets ☐☐<br>बड़ी गोली<br>Small Tablets ☐☐<br>छोटी गोली<br><br>Bottles ☐☐ . ☐☐<br>बोतल		
D54B	**(RC3)** How many IFA tablets/syrup bottles did you consume after the delivery?<br><br>प्रसव के बाद आपको कितने IFA की गोली और सिरप की बोतलें खायी थी?	Large Tablets ☐☐<br>बड़ी गोली<br>Small Tablets ☐☐<br>छोटी गोली<br><br>Bottles ☐☐ . ☐☐<br>बोतल		
D54C	**(RC3)** After the delivery, how many calcium tablets did you receive or purchase?<br><br>प्रसव के बाद आपको कितने कैल्शियम की गोलीया मिली थी?	टेबलेट'<br><br>Tablets ☐☐		
D54D	**(RC3)** How many calcium tablet did you consume after the delivery?<br><br>सव के बाद आपको कितने कैल्शियम की गोली खायी थी?	टेबलेट'<br><br>Tablets ☐☐		
D54E	**ASK IFD54C >0 OR D54B>0**<br>After the delivery, for how many days did you take the tablets or syrup?<br>प्रसव के बाद आपने कितने दिनों तक आईरन फोलिक एसिड (ताकत) की गोली या सिरप खायी थी?<br><br>**IF ANSWER IS NOT NUMERIC, PROBE FOR APPROXIMATE NUMBER OF DAYS.**<br>;fn mÙkj l[;k e ugh g] iN fd fnuk dh l[;k yxHkx D;k FkhA	☐☐☐ NO OF DAYS FOR LARGE TABLETS बड़ी गोलियाँ खाने के दिनों की संख्या<br>☐☐☐ NO OF DAYS FOR SMALL TABLETS छोटी गोलियों दिनों की संख्या<br>☐☐☐ NO OF DAYS FOR SYRUP सिरप खाने के दिनों की संख्या<br>☐☐☐ NO OF DAYS FOR CALCIUM TABLETS कैल्शियम की गोली खाने के दिनों की संख्या<br><br>DO NOT REMEMBER याद नहीं		

**E. LABOR AND DELIVERY (RC1/2)**<br>This section will be asked to category1 and category2, i.e. recently delivered mothers of 0-11 months child. Few of the questions will also be asked to category 3,mothers of 11-23 months child(indicated in the questions)		
Read out to the respondent The next questions ask about labor and delivery and your experience during the birth of [CHILD NAME].<br>mÙkjnkrk dk i<dj luk;s vxys dN ç'u vkid çlo vkj ¼cPps dk uke½ d tUe d le; d vuHkok d ckj e gA		
E1.	**(RC3)** Where did you deliver [CHILD NAME]?<br>आपने (बच्चे का नाम) को जन्म कहाँ दिया था?<br>**IF AT A FACILITY, PROBE: WHAT TYPE OF FACILITY WAS THIS? IF UNABLE TO DETERMINE IF A HOSPITAL,**	1 = HOME घर पर<br>2 = GOVERNMENT/MUNICIPAL HOSPITAL सरकारी/नगरपालिका का अस्पताल<br>3 = GOVERNMENT DISPENSARY सरकारी दवाखाना

	**HEALTH CENTRE, OR CLINIC IS PUBLIC OR PRIVATE MEDICAL SECTOR, WRITE THE NAME OF THE PLACE.**<br>;fn LokLF; lfo/kk e rc çkc djh LokLF; lfo/kk fdl çdkj dh Fkh\ ;fn ;g fu/kkfjr djuk e leL;k gks jgh gS fd LokLF; lfo/kk] vLirky Fkk LokLF; dUæ lkoZtfud Fkk ;k futh {k= dk] LFkku dk uke fy[kA<br><br>____________________<br>NAME OF PLACE स्थान का नाम<br><br>**CODE ONLY ONE**<br>dsoy ,d mÙkj	4 = UHC/UHP/UFWC<br>यूएचसी / यूएचपी / यूएफडब्ल्यूसी<br>5 = CHC सीएचसी<br>6 = APHC/NPHC/BPHC<br>एपीएचसी / एनपीएचसी / बीपीएचसी<br>7 = SUB-CENTER उपकेन्द्र<br>8 = NGO HOSPITAL/CLINIC गैर सरकारी संस्था का अस्पताल / क्लीनिक<br>9 = PRIVATE HOSPITAL/CLINIC प्राइवेट अस्पताल / क्लीनिक<br>88 OTHERS (SPECIFY) अन्य (स्पष्ट करें)
E1 A	**SKIP IF E1=1**<br><br>Did you go to any other facility before coming to this facility?<br>इस स्वास्थ्य सुविधा में जाने से पहले क्या आप किसी और स्वास्थ्य सुविधा गई थी?	1 = YES हाँ<br>0 = NO नहीं → **GO TO** E3
E1 B	**SKIP IF E1=1**<br><br>Where did you go?<br>आप किस स्वास्थ्य सुविधा में गई थी?<br><br>____________________<br>NAME OF PLACE स्थान का नाम	1 = GOVERNMENT/MUNICIPAL HOSPITAL सरकारी / नगरपालिका का अस्पताल<br>2 = GOVERNMENT DISPENSARY सरकारी दवाखाना<br>3 = UHC/UHP/UFWC<br>यूएचसी / यूएचपी / यूएफडब्ल्यूसी<br>4 = CHC सीएचसी<br>5 = APHC/NPHC/BPHC<br>एपीएचसी / एनपीएचसी / बीपीएचसी<br>6 = SUB-CENTER उपकेन्द्र<br>7 = NGO HOSPITAL/CLINIC गैर सरकारी संस्था का अस्पताल / क्लीनिक<br>8 = PRIVATE HOSPITAL/CLINIC प्राइवेट अस्पताल / क्लीनिक<br>88 = OTHERS (SPECIFY) अन्य (स्पष्ट करें)
E1 C	Why did you move out of this facility and go to the facility where the child was delivered?	1=GOT REFERRED TO THE NEXT FACILITY<br>रेफेर किया गया दूसरे फैसिलिटी में<br>2=THERE WERE NO AVAILABLE LABOR ROOMS FOR THE DELIVERY<br>डिलीवरी के लिए लबोर रूम्स उपलब्ध नहीं थे<br>3=DOCTOR/NURSE DID NOT ATTEND TO US<br>डॉक्टर/नर्स आये ही नहीं हमें देखने<br>4=DID NOT LIKE THE SERVICES AT THAT FACILITY<br>उस फैसिलिटी की सुविधाएं नहीं पसंद आई<br>88=OTHERS<br>अन्य
E2.	**ASK ONLY IF D36=2 AND E1=1:**<br><br>Earlier, you said you planned to deliver at a facility, but you delivered at home. Why did you change your mind?<br>पहले आपने बताया था कि आप स्वास्थ्य सुविधा में प्रसव कराना चाहती थीं, पर आपने घर पर प्रसव कराया। आपने अपना मन क्यों बदला था?<br><br>**PROBE : ANY OTHERS?**<br>çkc % dqN vkj\<br><br>**RECORD ALL MENTIONED**<br>lHkh mÙkj fy[kA	1 = DELIVERING AT HOME SEEMED LIKE IT WOULD BE MORE COMFORTABLE घर पर प्रसव कराना अधिक सुविधाजनक लगा<br>2 = IT WAS TOO INCONVENIENT TO GO TO THE FACILITY WHEN THE TIME ARRIVED जब समय आया तो स्वास्थ्य सुविधा में पहुंचना काफी असुविधाजनक था<br>3 = FAMILY CONVINCED ME NOT TO GO TO THE FACILITY परिवार ने मुझे स्वास्थ्य सुविधा न जाने के लिए मनाया<br>4 = I COULD NOT AFFORD THE COSTS OF DELIVERING AT A FACILITY, SUCH AS TRANSPORT COSTS मैं स्वास्थ्य सुविधा में प्रसव का खर्च जैसे कि परिवहन का खर्च, नहीं उठा सकती थी<br>5 = THERE WAS NO TRANSPORTATION TO THE FACILITY स्वास्थ्य सुविधा जाने के लिए वाहन नहीं था

		6 = THE BABY CAME BEFORE WE COULD GET TO THE FACILITY स्वास्थ्य सुविधा जाने से पहले ही बच्चा गर्भ से बाहर आ गया<br>88 = OTHER (SPECIFY) अन्य (स्पष्ट करें)
E3.	**(RC3)** Who conducted the delivery of [CHILD NAME]?<br>(बच्चे का नाम) का जन्म किसने कराया?<br><br>**CODE ONLY ONE**<br>dsoy ,d mÙkj	1 = GOVERNMENT DOCTOR सरकारी डाक्टर<br>2 = PRIVATE DOCTOR प्राइवेट डाक्टर<br>3 = STAFF NURSE स्टाफ नर्स<br>4 = LHV लेडी हैल्थ विजिटर (एल.एच.वी.)<br>5 = MALE HEALTH WORKER पुरुष स्वास्थ्य कर्मी<br>6 = ANM ए.एन.एम.<br>7 = OTHER HEALTH PERSONNEL अन्य स्वास्थ्य कर्मी<br>8 = ASHA आशा<br>9 = AWW आँगनवाड़ी कार्यकर्ता<br>10 = SBA/TRAINED DAI एसबीए/प्रशिक्षित दाई<br>11 = DAI दाई<br>12 = RMP आर.एम.पी. (झोला छाप डाक्टर)<br>13 = FAMILY MEMBER परिवार के सदस्य<br>14 = FRIENDS/NEIGHBOUR मित्र/पड़ौसी<br>88 = OTHERS (SPECIFY) अन्य (स्पष्ट करें)<br>98 = NO ONE कोई नहीं<br>99 = DO NOT KNOW पता नहीं
E4.	Who assisted the delivery of [CHILD NAME]?<br>(बच्चे का नाम) के जन्म में किस किस ने सहायता की थी?<br><br>**PROBE FOR THE TYPE OF PERSON AND RECORD ALL PERSONS ASSISTING. ANY OTHERS? RECORD ALL MENTIONED**<br>çkc % ml 0;fDr dk izdkj D;k Fkk ftlus vkidh lgk;rk dh Fkh vkj lHkh 0;fDr;ksa dk ntZ djsa D;k dksbZ vkj Fkk\ lHkh mÙkj fy[ksa<br><br>**IF RESPONDENT SAYS NO ONE ASSISTED, PROBE TO DETERMINE WHETHER ANY ADULTS WERE PRESENT AT THE DELIVERY.**<br>यदि उत्तरदाता यह कहती है कि किसी ने सहायता नहीं की, यह निर्धारित करने के लिए पूछें कि क्या कोई वयस्क व्यक्ति प्रसव के समय उपस्थित था।	1 = GOVERNMENT DOCTOR सरकारी डाक्टर<br>2 = PRIVATE DOCTOR प्राइवेट डाक्टर<br>3 = STAFF NURSE स्टाफ नर्स<br>4 = LHV लेडी हैल्थ विजिटर (एल.एच.वी.)<br>5 = MALE HEALTH WORKER पुरुष स्वास्थ्य कर्मी<br>6 = ANM ए.एन.एम.<br>7 = OTHER HEALTH PERSONNEL अन्य स्वास्थ्य कर्मी<br>8 = ASHA आशा<br>9 = AWW आँगनवाड़ी कार्यकर्ता<br>10 = SBA/TRAINED DAI एसबीए/प्रशिक्षित दाई<br>11 = DAI दाई<br>12 = RMP आर.एम.पी. (झोला छाप डाक्टर)<br>13 = FAMILY MEMBER परिवार के सदस्य<br>14 = FRIENDS/NEIGHBOUR मित्र/पड़ौसी<br>88 = OTHERS (SPECIFY) अन्य (स्पष्ट करें)<br>98 = NO ONE कोई नहीं<br>99 = DO NOT KNOW पता नहीं
ASK E5–E17QUESTIONS ONLY TO WOMEN WHO DELIVERED AT A HEALTH FACILITY<br>çu **E5–E17**mu dsoy efgykvksa ls iwNs ftUgksaus LokLF; lqfo/kk esa çlo djk;k gksA		
E5.	Who all went with you to the health facility for your delivery?<br>जब प्रसव के लिए आप स्वास्थ्य सुविधा गयी थी, आपके साथ कौन गया था?<br><br>**PROBE : ANY OTHERS?**<br>çkc % dksbZ vkSj\<br><br>**RECORD ALL MENTIONED**<br>lHkh mÙkj fy[ksa	1 = HUSBAND पति<br>2 = MOTHER-IN-LAW सास<br>3 = MOTHER माँ<br>4 = OTHER RELATIVES अन्य रिश्तेदार<br>5 = FRIENDS/NEIGHBORS मित्र/पड़ोसी<br>6 = ANM ए.एन.एम.<br>7 = DAI दाई<br>8 = SBA/TRAINED DAI एसबीए/प्रशिक्षित दाई<br>9 = ASHA आशा<br>10 = AWW आँगनवाड़ी कार्यकर्ता<br>88 OTHERS (SPECIFY) अन्य (स्पष्ट करें)<br>98= NO ONE कोई नहीं
**E5 A**	During the delivery, apart from the health service provider was there anybody else present in the labour room?<br>प्रसव के समय क्या स्वास्थ्य सेवा प्रदाता के अतिरिक्त लेबर रूम मे कोई और उपस्थित था?	1 = YES हाँ<br>0 = NO नहीं.<br>99 = DO NOT KNOW पता नहीं
**E5 B**	Was the woman denied companionship of the husband/relatives/or any other attendant during delivery?	1 = YES हाँ<br>0 = NO नहीं<br>99 = DO NOT KNOW पता नहीं

	क्या महिला के पति, रिश्तेदार, घर के अन्य लोगों को प्रसव के समय लेबर रूम में उपस्थित रहने से रोका गया था?	
E6.	**(RC3)** Was the delivery caesarean?<br>क्या प्रसव आपरेशन द्वारा किया गया था?	1 = YES हाँ<br>0 = NO नहीं
E7.	Were you given any injection to increase the labour pain at the time of delivery of [CHILD NAME]?<br>क्या (बच्चे का नाम) के जन्म के समय प्रसव पीड़ा बढ़ाने के लिए आपको कोई सुई लगायी गयी थी?	1 = YES हाँ<br>0 = NO नहीं
E8.	**(RC3)** Were you given an injection in your thigh immediately after delivery of [CHILD NAME]?<br>क्या (बच्चे का नाम) के तुरन्त बाद आपकी जाँग पर कोई सुई लगायी गयी थी?	1 = YES हाँ<br>0 = NO नहीं<br>99 = DO NOT KNOW पता नहीं
E9.	**(RC3)** How long did you stay at the healthcare facility after [CHILD NAME] was delivered?<br>आप (बच्चे का नाम) के जन्म के बाद स्वास्थ्य सुविधा में कब तक रूकी थी?<br>**CODE HOURS, DAYS, OR WEEKS.**<br>घंटे, दिन और सप्ताह में कोड करें।	☐ ☐☐<br>1 = HOURS घंटे<br>2 = DAYS दिन<br>3 = WEEKS सप्ताह
E9a	Was this earlier than the nurse/doctor recommended?<br>नर्स/डॉक्टर ने आपको जितने समय स्वस्थ्य केंद्र में रुकने की सलाह दी थी क्या आप उस समय से पहले स्वस्थ्य केंद्र से लौट आये ?	1 = YES<br>0 = NO<br>99 = DID NOT RECOMMEND
E9b	What mode of transportation did you take to get to travel from healthcare facility to home?<br>आप स्वस्थ्य केंद्र से घर कैसे गए?<br>**CODE ALL THAT APPLY**	1= AMBULANCE<br>2 TAXI/JEEP/TRACTOR<br>3=MOTOR CYCLE<br>4=CAR/TRUCK<br>5= BUS<br>6= BULLOCK/ANIMAL-DRAWN CART<br>7= ON FOOT<br>8=BICYCLE<br>9= AUTORICKSHAW/TEMPO<br>88= OTHER (SPECIFY)
E9c	How long did it take you to reach the home from healthcare facility?<br>आपको स्वस्थ्य केंद्र से घर पहुचने में कितना समय लगा?	☐ ☐☐<br>1 = HOURS घंटे<br>2 = DAYS दिन
E10.	How much did it cost you out of your pocket during delivery on:<br>प्रसव के लिए आपका कितना पैसा खर्च हुआ था?	0000 = NO MONEY PAID कोई पैसा नहीं दिया<br>9999 = DO NOT KNOW/REMEMBER पता नहीं/याद नहीं
a.	Total कुल खर्चा	☐☐☐☐☐ AMOUNT IN INR राशि भारतीय रुपये में
b.	Hospital stay अस्पताल में ठहरने	☐☐☐☐☐ AMOUNT IN INR राशि भारतीय रुपये में
c.	Tests done जाँचें	☐☐☐☐☐ AMOUNT IN INR राशि भारतीय रुपये में
d.	Medicines दवायें	☐☐☐☐☐ AMOUNT IN INR राशि भारतीय रुपये में
e.	Transportation यातायात	☐☐☐☐☐ AMOUNT IN INR राशि भारतीय रुपये में
f.	Any other costs अन्य कोई खर्चा	☐☐☐☐☐ AMOUNT IN INR राशि भारतीय रुपये में
E11.	How the out of pocket cost was met?<br>आपने जो पैसा खर्च किया उसे कहाँ से प्राप्त किया था?<br><br>**PROBE : ANY OTHERS?**<br>çkc % dìN vkj\<br><br>**RECORD ALL MENTIONED**<br>lHkh mÙkj fy[kA	1 = BANK ACCOUNT/SAVINGS बैंक खाता/बचत<br>2 = BORROWED FROM NEIGHBOURS/ RELATIVES/ FRIENDS रिश्तेदार/ पड़ौसियों/दोस्तों से पैसे उधार लिय<br>3 = BORROWED FROM MONEY LENDER साहूकार से पैसे उधार लिये<br>4 = SELLING LAND जमीन बेची<br>5 = SELLING JEWELLERY गहने बेचे<br>6 = SELLING OTHER ASSETS दूसरी संपत्तियां बेची<br>7 = MORTGAGING LAND जमीन गिरवी<br>8 = MORTGAGING JEWELLERY गहने गिरवी<br>9 = MORTGAGING OTHER ASSETS अन्य संपत्ति गिरवी<br>10 = INSURANCE बीमा<br>11= GIFTS FROM NEIGHBOURS/ FRIENDS/RELATIVES पड़ौसियों/मित्रों/रिश्तेदारों से उपहार

		88 = OTHERS (SPECIFY) अन्य (स्पष्ट करें)<br>99 = DO NOT KNOW पता नहीं
**SKIP E12-E17 IF E1CODED 8 OR 9**		
E12.	Did you receive any financial assistance for delivery care?<br>क्या आपको प्रसव देखरेख के लिए कोई वित्तीय सहायता/पैसा मिला था?	1 = YES हाँ<br>0 = NO नहीं → **GO TO** E15<br>99 = DO NOT KNOW पता नहीं → **GO TO** E15
E13.	How much was the payment received?<br>आपको कितनी वित्तीय सहायता/पैसा मिला था?	□□□□ TOTAL AMOUNT PAID कुल दी गई राशि<br>9999 = DO NOT KNOW पता नहीं
E14.	How long after the delivery did you receive the financial payment/assistance?<br>प्रसव के कितने समय बाद आपको वित्तीय सहायता/पैसा मिला था?	□ □□<br>1 = DAYS दिन<br>2 = WEEKS सप्ताह<br>3 = MONTHS माह<br>77 = IMMEDIATELY AT THE HOSPITAL अस्पताल में तुरन्त
E15.	Did you have to pay a bribe for getting the payment/assistance?<br>क्या आपको वित्तीय सहायता/पैसा मिलने के लिए किसी को रिश्वत देना पड़ा था?	1 = YES हाँ<br>0 = NO नहीं → **GO TO** E18
E16.	Who did you have to pay a bribe for getting the payment/assistance?<br>आपने वित्तीय सहायता/पैसा मिलने के लिए किसको रिश्वत दी थी?<br><br>**PROBE : ANY OTHERS?**<br>çkc s dN vkj\<br><br>**RECORD ALL MENTIONED**<br>lHkh mÙkj fy[kA	1 = GOVERNMENT DOCTOR सरकारी डाक्टर<br>2 = STAFF NURSE स्टाफ नर्स<br>3 = LHV लेडी हैल्थ विजिटर (एल.एच.वी.)<br>4 = MALE HEALTH WORKER पुरुष स्वास्थ्य कर्मी<br>5 = ANM ए.एन.एम.<br>6 = OTHER HEALTH PERSONNEL अन्य स्वास्थ्य कर्मी<br>7 = ASHA आशा<br>8 = AWW आँगनवाड़ी कार्यकर्ता<br>9 = SBA/TRAINED DAI एसबीए/प्रशिक्षित दाई<br>10 = DAI दाई<br>88 = OTHERS (SPECIFY) अन्य (स्पष्ट करें)<br>99 = DO NOT KNOW पता नहीं
E17.	How much money had to be paid in order to get the payment/assistance?<br>आपने वित्तीय सहायता/पैसा मिलने के लिए कितनी रिश्वत दी थी?	□□□□ TOTAL AMOUNT PAID कुल दी गई राशि<br>9999 = DO NOT KNOW पता नहीं
Read out to the respondent Now, I'd like to ask you some questions about things that can happen during delivery and your experiences.<br>mÙkjnkrk dk i<dj luk;a vc e vki l çlo d nkjku ?kV I du okyh ?kVuk;a vkj vkid vuHkok d ckj e dN ç'u iNuk pkgxhA		
E18.	Do you know of any danger signs or symptoms that a woman might have during delivery or within the first month after delivery that would require seeking medical care?<br>क्या आप खतरे के ऐसे लक्षणों या संकेतों के बारे मे जानती हैं जिनका अनुभव महिला प्रसव के दौरान या प्रसव के बाद एक महीने के अन्दर कर सकती है और जिसके लिए चिकित्सा देखरेख की जरूरत होती है?	1 = YES हाँ<br>0 = NO नहीं → **GO TO** E21
E19.	Could you name some of the danger signs or symptoms that a woman might have during delivery or within the first month after delivery that would require seeking medical care?<br>क्या आप खतरे के ऐसे लक्षणों या संकेतों के बारे मे बता सकती हैं जिनका अनुभव महिला प्रसव के दौरान या प्रसव के बाद एक महीने के अन्दर कर सकती है और जिसके लिए चिकित्सा देखरेख की जरूरत होती है?<br><br>**PROBE : ANY OTHERS?**<br>çkc s dN vkj\<br><br>**RECORD ALL MENTIONED**<br>lHkh mÙkj fy[kA	1 = PROLONGED LABOUR FOR MORE THAN 12 HOURS लंबे समय तक, 12 घंटे से अधिक समय तक प्रसव पीड़ा<br>2 = RETAINED PLACENTA गर्भनाल का अंदर रहना<br>3 = OBSTRUCTED LABOUR (MALPOSITION/PRESENTATION) रुकावट के साथ प्रसव (शिशु का गलत स्थिति में होना)<br>4 = SEVERE BLEEDING अत्यधिक खून निकलना<br>5 = DIFFICULTY BREATHING सांस लेने में कठिनाई<br>6 = SEVERE PAIN IN ABDOMEN पेट में तेज दर्द<br>7 = SEVERE WEAKNESS अत्यधिक कमजोरी<br>8 = CONVULSIONS दौरे पड़ना<br>9 = FOUL SMELLING VAGINAL DISCHARGE योनि से बदबूदार पानी निकलना<br>10 = FEVER बुखार<br>11 = SEVERE HEADACHE तेज सिर दर्द<br>12 = LOSS OF CONSCIOUSNESS होश खोना

		13 = DOUBLE VISION एक के दो दिखना<br>88 = OTHERS (SPECIFY) अन्य (स्पष्ट करें)
E20.	From where did you get the information about these danger signs that a woman can have during delivery or post-delivery?<br>प्रसव के दौरान या प्रसव के बाद महिला को खतरे के इन संकेतों के बारे में आपको कहाँ से जानकारी मिली थी?<br><br>**PROBE : ANY OTHERS?**<br>çkc ¼ dN vkj\<br><br>**RECORD ALL MENTIONED**<br>IHkh mÙkj fy[kA	1 = GOVERNMENT DOCTOR सरकारी डाक्टर<br>2 = PRIVATE DOCTOR प्राइवेट डाक्टर<br>3 = STAFF NURSE स्टाफ नर्स<br>4 = LHV लेडी हैल्थ विजिटर (एल.एच.वी.)<br>5 = MALE HEALTH WORKER पुरुष स्वास्थ्य कर्मी<br>6 = ANM ए.एन.एम.<br>7 = OTHER HEALTH PERSONNEL अन्य स्वास्थ्य कर्मी<br>8 = ASHA आशा<br>9 = AWW आँगनवाड़ी कार्यकर्ता<br>10 = SBA/TRAINED DAI एसबीए/प्रशिक्षित दाई<br>11 = DAI दाई<br>12 = RMP आर.एम.पी. (झोला छाप डाक्टर)<br>13 = FAMILY MEMBER परिवार के सदस्य<br>14 = FRIENDS/NEIGHBOUR दोस्त/पड़ौसी<br>15 = RADIO/TV/NEWS PAPER रेडियो/टेलिविजन/समाचार पत्र<br>16 = MOBILE SMS मोबाइल एसएमएस<br>88 = OTHERS (SPECIFY) अन्य (स्पष्ट करें)<br>98 = NO ONE कोई नहीं<br>99 = DO NOT KNOW पता नहीं
E21.	Were you told to visit a doctor/facility in case of any complications during delivery or in the first month after delivery?<br>क्या आपको यह बताया गया था कि प्रसव के समय या प्रसव के बाद एक महीने के अन्दर किसी भी जटिलता या समस्या होने पर डाक्टर/स्वास्थ्य केंद्र जायें?	1 = YES हाँ<br>0 = NO नहीं
**E21A**	Did your pregnancy end before it was expected?<br>या गर्भावस्था समय से पहले समाप्त हुआ था?	1 = YES हाँ<br>0= NO नहीं
**E21B**	How much time before the expected date of delivery did the pregnancy end?<br>प्रसव की सम्भावित तिथि से कितने **समय** पहले गर्भावस्था का समापन हुआ था?	☐ ☐☐<br>1 = DAYS दिन<br>2 = WEEKS सप्ताह<br>3 = MONTHS माह

E22.	(RC3) Did you experience any danger signs during the delivery or during the first month after delivery?<br>प्रसव के समय या उसके बाद एक महीने के अन्दर क्या आपने किसी समस्या या परेशानी का अनुभव किया था?<br><br>**DO NOT READ LIST. CODE ALL MENTIONS IN FIRST COLUMN. THEN READ EACH ITEM BELOW (a-r) THAT WASN'T MENTIONED AND CODE RESPONSE IN SECOND COLUMN. IF NOT MENTIONED, SKIP TO NEXT LETTER.**<br>lph dks u i<sA igys dkye esa lHkh mYys[k fd;s x;s mÙkjksa dks dksM djsA blds ckn mu fcUnqvksa ¼,&vkj½ dks i<sa ftudk mYys[k ugha fd;k x;k Fkk vkj nwljs dkye esa mÙkj dks dksM djsA ;fn ugha crk;k x;k vxys fcanq ij tk;sA	1 = YES, SPONTANEOUS<br>हाँ, स्वयं बताया	2 = YES, AFTER READING<br>हाँ, पढ़ने के बाद	0 = NO<br>नहीं
a.	Premature labor (less than 37 weeks) समय से पहले (37 सप्ताह से पहले) प्रसव पीड़ा	1	2	0
b.	Preterm/premature rupture of membrane समय से पहले झिल्ली का फटना	1	2	0
c.	Prolonged labor for more than 12 hours प्रसव पीड़ा लंबे समय तक (12 घंटे से अधिक) रहना	1	2	0
d.	Retained placenta खेड़ी का बाहर नहीं निकलना	1	2	0
e.	Obstructed labor रुकावट के साथ प्रसव पीड़ा होना	1	2	0
f.	Breech/mal presentation भ्रूण का गलत स्थिति में होना	1	2	0
g.	Excessive bleeding before delivery प्रसव के पहले अत्यधिक खून निकलना	1	2	0
h.	Excessive bleeding immediately after delivery प्रसव के तुरन्त बाद अत्यधिक खून निकलना	1	2	0
i.	High Blood Pressure उच्च रक्तचाप	1	2	0
j.	Difficulty breathing साँस लेने में कठीनाई	1	2	0
k.	Severe pain in abdomen पेट में तेज दर्द	1	2	0
l.	Severe weakness अत्यधिक कमजोरी	1	2	0
m.	Convulsions/fits दौरे पड़ना	1	2	0

n.	Foul smelling vaginal discharge योनी से बदबूदार स्राव	1	2	0
o.	Fever बुखार	1	2	0
p.	Severe headache तेज सिर दर्द	1	2	0
q.	Loss of consciousness बेहोश होना	1	2	0
r.	Blurred vision धुंधला दिखना	1	2	0

E23. ASK ONLY IF RESPONSE TO E22h=1 OR 2, ELSE GO TO E23A1
rHkh iNs tc ***E22h*** dk mÙkj 1 ;k 2 gk(ugha rks ***E24*** ij tk;s

**A.**	Did you have any bleeding along with experiencing weakness/dizziness?<br>क्या खून निकलने के साथ आपने कमजोरी/सिर चकराने का अनुभव किया था?	1 = YES हाँ<br>0 = NO नहीं
**B.**	Did you notice fist-sized blood clots from your vagina?<br>क्या आपने योनि से खून के मुट्ठी आकार के थक्के निकलते देखा था?	1 = YES हाँ<br>0 = NO नहीं
**C.**	Did the bleeding wet your clothes, the bed, or the floor?<br>क्या खून आने से आपके कपड़े, बिस्तर या फर्श गीला हो गया था?	1 = YES हाँ<br>0 = NO नहीं
**D.**	Did you experience high grade fever/chills?<br>क्या आपको तेज बुखार/कंपकंपी का अनुभव हुआ था?	1 = YES हाँ<br>0 = NO नहीं
**E23A1**	Did you have sepsis after the delivery (HIGH FEVER WITH CHILLS, FOUL SMELLING VAGINAL DISCHARGE, PAIN IN THE LOWER ABDOMEN)?<br>*क्या आपको प्रसव के बाद संक्रमण हुआ था (तेज़ बुखार, कपकपी, योनी से बदबूदार पानी निकलना, पेट के निच्च्ले हिस्से में तेज़ दर्द) ?*	1 = YES हाँ<br>0= NO नहीं
E24.	**ASK ONLY IF ANY OF THE RESPONSES *E22*(a-r) OR E23A1 IS CODED 1 OR 2 ELSE GOTO E26**<br>;g Á'u rHkh iNs tc ***E22*(a-r)** esa fdlh mÙkj dk dkM 1 ;k 2 gS<br><br>Did you seek treatment for this problem?<br>क्या आपने इस समस्या का इलाज कराया था?	1 = YES हाँ<br>0 = NO नहीं → **GO TO E26**
E25.	From where did you seek treatment?<br>आपने इलाज कहाँ कराया था?<br><br>**PROBE : ANY OTHERS?**<br>çkcs % dqN vkSj\<br><br>**RECORD ALL MENTIONED**<br>lHkh mÙkj fy[ksA\\\	1 = GOVERNMENT/MUNICIPAL HOSPITAL सरकारी/नगरपालिका का अस्पताल<br>2 = GOVERNMENT DISPENSARY सरकारी दवाखाना<br>3 = UHC/UHP/UFWC यूएचसी/यूएचपी/यूएफडब्ल्यूसी<br>4 = CHC सीएचसी<br>5 = APHC/NPHC/BPHC एपीएचसी/एनपीएचसी/बीपीएचसी<br>6 = SUB-CENTER उपकेन्द्र<br>7 = ANGANWADI CENTER आँगनवाड़ी केन्द्र<br>8 = VHND ग्राम स्वास्थ्य एवं पोषण दिवस<br>9 = NGO HOSPITAL/CLINIC गैर सरकारी संस्था का अस्पताल/क्लीनिक<br>10 = PRIVATE HOSPITAL/CLINIC प्राइवेट अस्पताल/क्लीनिक<br>11 = MEDICINE SHOP दवा की दुकान<br>12 = FOLK HEALER ओझा/वैद्य/झाड़फूँक<br>13 = HOME REMEDIES घरेलू उपचार<br>14 = RMP आर.एम.पी. (झोला छाप डाक्टर)<br>88 = OTHERS (SPECIFY) अन्य (स्पष्ट करें)
E26.	Did any health service provider give you this tablet **[SHOW MISOPROSTOL TABLETS]** to consume immediately after delivery?<br>क्या किसी स्वास्थ्य दाता ने आपके प्रसव के तुरन्त बाद खाने के लिए यह गोली ¼feb lkçkLVky dh xkfy;k fn[kk;s½ दी थी?	1 = YES हाँ<br>0 = NO नहीं → **GO TO** E31
E27.	How many tablets did the health service provider give you?<br>स्वास्थ्य सेवा प्रदाता ने आपको कितनी गोलियाँ दी थी?	☐ NUMBER OF TABLETS गोलियों की संख्या
**E29**	Did you consume the misoprostol tablet after the delivery of [CHILD NAME]?<br>क्या आपने (बच्चे का नाम) के जन्म के बाद मिइसोप्रोस्टोल की गोलियाँ खाई थी?	1 = YES हाँ<br>0 = NO नहीं → **GO TO** E31
E28.	How many tablets did you consume?<br>आपने कितनी गोलियाँ खाई थी?	☐ NUMBER OF TABLETS गोलियों की संख्या<br>0 = NO TABLETS CONSUMED कोई गोली नहीं खायी → **GO TO** E31
E30.	When did you consume the misoprostol tablet?<br>आपने मिइसोप्रोस्टोल की गोलियाँ कब खाई थी?	1 = BEFORE DELIVERY OF PLACENTA खेड़ी (आँवल) के निकलने से पहले

	**CODE ONLY ONE**<br>dsoy ,d mÙkj	2 = AFTER DELIVERY OF PLACENTA खेड़ी (आँवल) के निकलने के बाद<br>3 = AFTER INITIATION OF BREASTFEEDING स्तनपान शुरू करने के बाद<br>88 = OTHERS (SPECIFY) अन्य (स्पष्ट करें)
Read out to the respondent The next questions are about newborn care practices after the delivery of [CHILD NAME].<br>mÙkjnkrk dks i<dj lqukb;s vxys dN ç'u ¼cPps dk uke½ ds tUe ckn uotkr f'k'kq dh n[kHkky dh çFkkvksa ds ckjs esa gSA		
E31.	Who cut the umbilical cord?<br>नाल किसने काटी थी?<br><br>**CODE ONLY ONE**<br>dsoy ,d mÙkj	1 = GOVERNMENT DOCTOR सरकारी डाक्टर<br>2 = PRIVATE DOCTOR प्राइवेट डाक्टर<br>3 = STAFF NURSE स्टाफ नर्स<br>4 = LHV लेडी हैल्थ विजिटर (एल.एच.वी.)<br>5 = MALE HEALTH WORKER पुरुष स्वास्थ्य कर्मी<br>6 = ANM ए.एन.एम.<br>7 = OTHER HEALTH PERSONNEL अन्य स्वास्थ्य कर्मी<br>8 = ASHA आशा<br>9 = AWW आँगनवाड़ी कार्यकर्ता<br>10 = SBA/TRAINED DAI एसबीए/प्रशिक्षित दाई<br>11 = DAI दाई<br>12 = RMP आर.एम.पी. (झोला छाप डाक्टर)<br>13 = CORD CUTTER (USE LOCAL TERM) नाल काटने वाला/वाली (स्थानीय नाम का उपयोग करें)<br>14 = MOTHER-IN-LAW सास<br>15 = RESPONDENT'S MOTHER उत्तरदाता की माँ<br>16 = OTHER FAMILY MEMBER परिवार का अन्य सदस्य<br>17 = SELF स्वयं<br>88 = OTHERS (SPECIFY) अन्य (स्पष्ट करें)<br>99 = DO NOT KNOW पता नहीं
E32.	What instrument was used to cut the umbilical cord?<br>नाल को काटने के लिए क्या प्रयोग किया गया था?<br><br>**PROBE : Was it new or used and from where did you get it?**<br>çkcs% D;k ;g u;k Fkk ;k iqjkuk vkSj vkius mls dgka ls çkIr fd;k\<br><br>**CODE ONLY ONE**<br>dsoy ,d mÙkj	1 = NEW BLADE FROM HOME/ DDK KIT घर/डीडीके किट से नया ब्लेड<br>2 = USED BLADE पहले इस्तेमाल किया गया ब्लेड<br>3 = BLADE FROM SERVICE PROVIDER सेवा प्रदाता का ब्लेड<br>4 = SCISSOR कैंची<br>88 = OTHERS (SPECIFY) अन्य (स्पष्ट करें)<br>99 = DO NOT KNOW पता नहीं
E33.	What was used to tie the cord?<br>नाल को बाँधने के लिए क्या प्रयोग किया गया था?<br><br>**PROBE : Was it new or used and from where did you get it?**<br>çkcs% D;k ;g u;k Fkk ;k iqjkuk vkSj vkius mls dgka ls çkIr fd;k\<br><br>**CODE ONLY ONE**<br>dsoy ,d mÙkj	1 = NEW THREAD FROM HOME/DDK KIT घर/डीडीके किट से नया धागा<br>2 = USED THREAD पहले इस्तेमाल किया गया धागा<br>3 = THREAD FROM SERVICE PROVIDER सेवा प्रदाता का धागा<br>4 = CORD CLIP गर्भनाल क्लिप<br>88 = OTHERS (SPECIFY) अन्य (स्पष्ट करें)<br>99 = DO NOT KNOW पता नहीं
E34.	Was anything applied to the cord of [CHILD NAME] after it was cut?<br>क्या (बच्चे का नाम) की नाल काटने के बाद उस पर कुछ लगाया था?	1 = YES हाँ<br>0 = NO नहीं<br>99 = DO NOT KNOW पता नहीं
E35.	Was anything applied to the umbilicus after the cord dropped off?<br>क्या नाल गिरने के बाद नाभि पर कुछ लगाया गया था?	1 = YES हाँ<br>0 = NO नहीं<br>99 = DO NOT KNOW पता नहीं
E36.	**IF E34 AND E35 BOTH ARE 0 OR 99 SKIP TO E37**<br><br>What was applied to the cord after cutting and tying?<br>नाल को बाँधने और काटने के बाद क्या लगया गया था?<br><br>**PROBE : ANY OTHERS?**<br>çkcs % dN vkSj\<br><br>**RECORD ALL MENTIONED**	1 = DETTOL/SAVLON डेटाल/सैव्लोन<br>2 = ALCOHOL/SPIRIT शराब/स्प्रिट<br>3 = MUSTARD OIL सरसों का तेल<br>4 = SINDOOR सिंदूर<br>5 = GHEE घी<br>6 = BORIC POWDER बोरिक पाउडर<br>7 = GENTIAN VIOLET (NEELI DAWAI) जेनसिन व्वाइलेट (नीली दवा)

	lHkh mÙkj fy[kA	8 = TALCUM POWDER टेलकम पाउडर<br>9 = COW DUNG गाय का गोबर<br>10 = ASH राख<br>11 = CHLOROHEXIDINE क्लोरोहेक्सिडाइन<br>88 = OTHERS (SPECIFY) अन्य (स्पष्ट करें)<br>99 = DO NOT KNOW पता नहीं
**E36A**	How long after the delivery was anything applied to the cord?<br>बच्चे के जन्म के कितने समय बाद नाल पे कुछ लगाया गया था?	☐☐☐<br>0= IMMEDIATELY /MINUTES(less than 1 hours) तुरंत/मिनट में (1 घण्टे के अन्दर)<br>1 = HOURS घंटे<br>2 = DAYS दिन<br>99 = DO NOT KNOW पता नहीं
E37.	After delivery, was the baby wiped dry, wiped with a wet cloth, or bathed?<br>प्रसव के बाद क्या बच्चे को सुखे कपडे से पोछा गया, गीले कपड़े से पोंछा गया या नहलाया गया?	1 = WIPED WITH A DRY CLOTH सुखे कपड़े से पोंछा गया<br>2 = WIPED WITH A WET CLOTH गीले कपड़े से पोंछा गया<br>3 = BATHED नहलाया गया<br>99 = DO NOT KNOW पता नहीं
**E37A**	ASK IF E37=1 ,ELSE SKIP TO E38<br><br>How soon after the delivery was the baby wiped dry?<br>प्रसव के कितने समय बाद क्या बच्चे को सुखे कपडे से पोछा गया?	☐☐☐<br>0=MINUTES<br>1 = HOURS घंटे<br>2 = DAYS दिन<br>99 = DO NOT KNOW पता नहीं
E38.	Was [CHILD NAME] wrapped in a cloth after delivery?<br>प्रसव के बाद क्या (बच्चे का नाम) को कपड़े में लपेटा गया था?	1 = YES हाँ<br>0 = NO नहीं<br>99 DO NOT KNOW पता नहीं
**E38A**	**ASK IF E38=1, ELSE SKIP TO E39**<br><br>Was the child wrapped in the same cloth used for wiping?<br>क्या बच्चे को जिस कपडे से पोचा था, उसी कपडे से लपेटा था?	1 = YES हाँ<br>0 = NO नहीं<br>99=DO NOT KNOW पता नहीं
E39.	How soon after the delivery was [CHILD NAME] given (his/her) first bath?<br>प्रसव के कितने समय बाद (बच्चे का नाम) को पहली बार नहलाया गया था?<br><br>**CODE ONLY ONE**<br>doy ,d mÙkj	☐☐☐<br>0 = IMMEDIATELY (less than 1 hours) तुरंत (1 घण्टे के अन्दर)<br>1 = HOURS घंटे<br>2 = DAYS दिन<br>98 = NOT BATHED अभी तक नहलाया नहीं गया<br>99 = DO NOT KNOW पता नहीं
E40.	When was [CHILD NAME] weighed for the first time?<br>(बच्चे का नाम) का वजन पहली बार कब लिया गया था?	☐☐☐ NO. OF DAYS दिनों की संख्या<br>000 = SAME DAY OF BIRTH जन्म के ही दिन<br>998 = NEVER WEIGHED कभी वजन नहीं लिया गया<br>→ **GO TO** E42<br>999 = DO NOT KNOW/REMEMBER पता नहीं/याद नहीं → **GO TO E42**
E41.	What was the weight of [CHILD NAME]?<br>(बच्चे का नाम) का वजन क्या था?<br><br>**RECORD IN GRAMS**<br>xzke esa ntZ djsa	☐. ☐☐☐ FROM HEALTH CARD स्वास्थ्य कार्ड से<br>☐. ☐☐☐ FROM RECALLयाद से<br>0= IF CARD IS NOT AVAILABLE<br>9999 = DO NOT KNOW पता नहीं
E42.	Did any health worker identify your [CHILD NAME] as weak?<br>क्या किसी स्वास्थ्य कार्यकर्ता ने आपको यह बताया था कि (बच्चे का नाम) कमजोर है?	1 = YES हाँ<br>0 = NO नहीं → **GO TO E45a**
E43.	Who identified [CHILD NAME] as weak?<br>किसने बताया था कि (बच्चे का नाम) कमजोर है?<br><br>**PROBE : ANY OTHERS?**<br>çkc djsa dksbZ vkSj\<br><br>**RECORD ALL MENTIONED**<br>lHkh mÙkj fy[kA	1 = GOVERNMENT DOCTOR सरकारी डाक्टर<br>2 = PRIVATE DOCTOR प्राइवेट डाक्टर<br>3 = STAFF NURSE स्टाफ नर्स<br>4 = LHV लेडी हैल्थ विजिटर (एल.एच.वी.)<br>5 = MALE HEALTH WORKER पुरुष स्वास्थ्य कर्मी<br>6 = ANM ए.एन.एम.<br>7 = OTHER HEALTH PERSONNEL अन्य स्वास्थ्य कर्मी<br>8 = ASHA आशा<br>9 = AWW आँगनवाड़ी कार्यकर्ता

		10 = SBA/TRAINED DAI एसबीए/प्रशिक्षित दाई<br>11 = DAI दाई<br>12 = RMP आर.एम.पी. (झोला छाप डाक्टर)<br>88 = OTHERS (SPECIFY) अन्य (स्पष्ट करें)<br>99 = DO NOT KNOW पता नहीं
E44.	What did they tell you about [CHILD NAME]?<br>(बच्चे का नाम) के बारे में उन्होंने आपको क्या बताया था?<br><br>**PROBE : ANY OTHERS?**<br>çkc % dN vkj\<br><br>**RECORD ALL MENTIONED**<br>lHkh mÙkj fy[kA	1 = PREMATURE समय से पहले जन्म<br>2 = LOW BIRTH WEIGHT जन्म के समय कम वजन का है<br>3 = SMALL छोटा है<br>4 = WEAK कमजोर है<br>88 = OTHERS (SPECIFY) अन्य (स्पष्ट करें)<br>99 = DO NOT REMEMBER याद नहीं
E45.	Was [CHILD NAME] treated?<br>क्या (बच्चे का नाम) का इलाज किया गया था?	1 = YES हाँ<br>0 = NO नहीं
**E45a**	Did the child experience asphyxia(not breathing or Gasping) during birth, not being able to cry right after birth?<br>**क्या जन्म के बाद बच्चे का दम घुटा था, सास नहीं ले पा रहा था बच्चा?(चेक कीजिये बच्चा जन्म के तुरंत बाद यदि रोया नहीं था)**	1 = YES हाँ<br>0 = NO नहीं
**E45b**	SKIP IF NO FOR E45a<br><br>Was the child resuscitated with bag and mask and established spontaneous breathing?<br>**क्या बच्चे को बैग एंड मास्क के मदत से सांस वापिस लायी गयी थी?**	1 = YES हाँ<br>0 = NO नहीं
**ASK E46 AND E47 ONLY IF E01=2-9, ELSE GO TO E47A**		
E46.	At any time when you were in the labour room or during your stay in the hospital, were you advised by the nurse or anyone else to keep [CHILD NAME] naked on your chest, next to your skin?<br>जब आप प्रसव कक्ष में थीं या अस्पताल मे थीं तब क्या नर्स या किसी और ने आपको यह बताया था कि (बच्चे का नाम) को त्वचा से त्वचा से संपर्क बना कर बिना कपड़ों के अपनी छाती पर रखना है।<br><br>**SHOW PICTURES OF SKIN TO SKIN CONTACT FROM WHO GUIDELINES.**<br>fo'o LokLF; laxBu ds fn'kkfunsZ'kksa ls Ropk ls Ropk laidZ ds fp= fn[kk;sA	1 = YES हाँ<br>0 = NO नहीं GOTO E47A
E47.	Did you practice keeping [CHILD NAME] in that position during your stay in the hospital?<br>क्या अस्पताल में ठहरने के दौरान आपने (बच्चे का नाम) को इस स्थिति में रखा था?	1 = YES हाँ<br>0 = NO नहीं
**E47A**	Did the ASHA/AWW/ANM advice you to keep [CHILD NAME] naked on your chest, next to your skin?<br>क्या आपको ए.एन.एम./आशा/आँगनवाड़ी कार्यकर्ता ने (बच्चे का नाम) को त्वचा से त्वचा के बीच संपर्क बना कर बिना कपड़ों के अपनी छाती पर रखने का सुझाव दिया था?<br>**SHOW PICTURES OF SKIN TO SKIN CONTACT FROM WHO GUIDELINES.**<br>fo'o LokLF; laxBu ds fn'kkfunsZ'kksa ls Ropk ls Ropk laidZ ds fp= fn[kk;sA	1 = YES हाँ<br>0 = NO नहीं
E48.	Did you practice keeping [CHILD NAME] in this position while at home?<br>क्या घर पर आपने (बच्चे का नाम) को इस स्थिति में रखा था?	1 = YES हाँ<br>0 = NO नहीं
**E47A1**	**Ask E47A1 and E47A2 if E47=1 or E48=1.**<br><br>How soon after the delivery did you first keep [CHILD NAME] naked on your chest, next to your skin ?<br>प्रसव के कितनी देर बाद आपने (बच्चे का नाम) को पहली बार त्वचा से त्वचा से संपर्क बना कर बिना कपड़ों के अपनी छाती पर रखना है।	☐ ☐☐<br>1 = HOURS घंटे<br>2 = DAYS दिन<br>99 = DO NOT KNOW/REMEMBER पता नही/याद नहीं
**E47A2**	How much time did you keep [CHILD NAME] in that position, the first time?<br>पहली बार आपने (बच्चे का नाम) को इस अवस्था मे कितनी देर तक रक्खा था?	☐ ☐☐<br>1 = HOURS घंटे<br>2 = DAYS दिन

		99 = DO NOT KNOW/REMEMBER पता नही/याद नहीं
E49.	**IF E47 AND E48 BOTH ARE 0 SKIP TO E51**<br><br>For how many days after birth did you practice this?<br>जन्म के बाद कितने दिनों तक आपने यह किया था?	☐☐ NUMBER OF DAYS दिनों की संख्या<br>99 = DO NOT KNOW/REMEMBER पता नहीं/याद है
E50.	For how many hours each day?<br>हर दिन कितने घंटे?	☐☐.☐☐ HOURS घंटे
**E50a1**	Was the child given Hepatitis B0 dose within 24 hours of birth?<br><br>**क्या बच्चे को जन्म के 24 घंटे के अन्दर हेपेटाइटिस B0 का दोसे दिया गया था के नहीं?**	1 = YES हाँ<br>0 = NO नहीं
**E50a2**	Was the child given Polio 0 dose within 14 days of birth?<br><br>**क्या बच्चे को जन्म के 14 दिन के अन्दर पोलियो की पहली खुराग दी गयी थी?**	1 = YES हाँ<br>0 = NO नहीं<br>99 = not applicable

ASK E51-E66 ONLY IF E1=2-9
यदि संस्थागत प्रसव है तो अगले प्रश्न पूछें; यदि घर पर प्रसव हुआ है तो भाग ई पर जायें
Read out to the respondent: The next questions are about any post-natal checks that you received at the facility for your health or child's health.
उत्तरदाता को पढ़कर सुनायें: अगले प्रश्न आपके और आपके बच्चे के स्वास्थ्य के लिए स्वास्थ्य सुविधा में की गई प्रसवोत्तर जाँचों के बारे में हैं।

E51.	Before you were discharged from the health facility after [CHILD NAME] was born, did a doctor or nurse check on your health?<br>(बच्चे का नाम) के जन्म के बाद स्वास्थ्य सुविधा से छुट्टी के पहले क्या डाक्टर या नर्स ने आपके स्वास्थ्य की जाँच की थी?	1 = YES हाँ<br>0 = NO नहीं → **GO TO E53**	
E52.	Did they do the following? क्या उन्होंने निम्नलिखित किया था?	YES हाँ	NO नहीं
R1.	Check your blood pressure रक्त–चाप (ब्लड प्रेशर) की जाँच	1	0
R2.	Take your temperature तापमान (टेम्परेचर) की जाँच	1	0
E52a.	Before you were discharged from the health facility after [CHILD NAME] was born, did you receive the following advice from a doctor, nurse, ANM or someone at the facility on caring for yourself?<br>(बच्चे का नाम) के जन्म के बाद और स्वास्थ्य सुविधा से छुट्टी के पहले क्या आपको अपनी देखरेख के बारे में डाक्टर, नर्स, ए.एन.एम. या अन्य किसी से निम्न सलाह मिली थी?	YES हाँ	NO नहीं
a.	Quantity of food to eat	1	0
b.	Types of food to eat	1	0
c.	Must consume IFA after delivery	1	0
d.	Must consume 100 IFA	1	0
e.	Must consume calcium after delivery	1	0
f.	Must consume 100 calcium after delivery	1	0

E53.	Before you were discharged from the health facility after [CHILD NAME] was born, did a doctor or nurse check on [CHILD NAME] health?<br>(बच्चे के जन्म) के बाद और स्वास्थ्य सुविधा से छुट्टी के पहले क्या डाक्टर या नर्स ने (बच्चे के जन्म) के स्वास्थ्य की जाँच की थी?	1 = YES हाँ<br>0 = NO नहीं → **GO TO E56**	
E54.	Did they do the following? क्या उन्होंने निम्नलिखित किया था?	YES हाँ	NO नहीं
a.	Examine the baby शिशु की जाँच	1	0
b.	Check the umbilical cord नाल की जाँच	1	0
c.	Observe you breastfeeding the baby आपको शिशु को स्तनपान कराते देखा था	1	0
d.	Take the baby's temperature शिशु के तापमान की जाँच	1	0
E56.	Before you were discharged from the health facility after [CHILD NAME] was born, did you receive the following advice from a doctor, nurse, ANM or someone at the facility on caring for your new born?<br>(बच्चे का नाम) के जन्म के बाद और स्वास्थ्य सुविधा से छुट्टी के पहले क्या आपको अपने नवजात शिशु की देखरेख के बारे में डाक्टर, नर्स, ए.एन.एम. या अन्य किसी से निम्न सलाह मिली थी?	YES हाँ	NO नहीं
a.	Breast feeding within one hour of delivery प्रसव के एक घंटे के अन्दर स्तनपान कराना	1	0

A1	Where to go for advice on breastfeeding in case of any difficulties<br>स्तनपान में कठिनाई होने पर कहाँ जान चाहिए सलाह के लिए	1	0
A2	How to position and attach the baby for optimal breastfeeding<br>बच्चे को सही से स्तनपान कराने के लिए किस तरह से छाती पे लगाके रखना है	1	0
A3	How to express breastmilk by hand<br>हाथ से कैसे स्तन से दूध निकलना है	1	0
b.	Techniques of breast feeding स्तनपान कराने की तकनीकें या तरीके	1	0
c.	Not to bath baby for at least for one week in case of low birth weight baby<br>अल्प वजन बच्चे के मामले में कम से कम एक सप्ताह तक बच्चे को नहलाना नहीं है	1	0
d.	Not to discard colostrum/yellow milk कोलोस्ट्रम/पीले दूध को बच्चे को अवश्य पिलाना	1	0
e.	Keep naked baby on bare chest of mother and wrap with a cloth or blanket<br>कपड़ों के बिना शिशु को मां की खुली छाती पर रखना और कपड़े या कंबल से ढक कर रखना	1	0
f.	To keep the child warm बच्चे को गर रखना	1	0
g.	Not to apply anything on the cord नाल पर कुछ भी नहीं लगाना	1	0
h.	Exclusive breast feeding for 6 months जन्म के बाद छह महीने तक केवल स्तनपान कराना	1	0
H1	When to start giving water and other fluids and foods to the child in addition to breastmilk<br>बच्चे को पानी और अन्य तरल पदार्थ कबसे दिया जाना चाहिए	1	0
H2	Danger of not exclusively breastfeeding for 6 months<br>६ महीने तक केवल स्तनपान न करवाने का खतरा	1	0
H3.	How to assess that the child is consuming an adequate supply of breastmilk<br>बच्चा सही मात्र में माँ का दूध पि रहा है उसको कैसे आंकना	1	0
H4.	How to increase supply of breastmilk in the first 6 months<br>जन्म के बाद पहले ६ महीने में स्तनों में दूध की मात्रा को कैसे बढाया जाए	1	0
i.	Return for routine postpartum check-up within 7 days even if you are feeling fine सात दिन के बाद ठीक महसूस करने पर भी दुबारा जांच के लिए वापस अस्पताल जाना	1	0
j.	Return if any complications experienced by mother or new-born<br>जच्चा–बच्चा को कोई समस्या हो तो वापस अस्पताल जाना	1	0
k.	Immunization schedule for the child बच्चे की टीकाकरण अनुसूची	1	0
l.	Not to bath baby for at least 3 days in case of normal delivery सामान्य प्रसव के मामले में कम से कम तीन दिन तक बच्चे को नहलाना नहीं है	1	0
E57.	In your opinion, how were the services in this facility? Would you say they were excellent, good, average or poor?<br>आपके विचार से इस स्वास्थ्य सुविधा में सेवाएं किस प्रकार की थीं? क्या वे बहुत अच्छी थीं, अच्छी थीं, औसत प्रकार की थीं, या ठीक नहीं थीं?	1 = EXCELLENT बहुत अच्छी<br>2 = GOOD अच्छी<br>3 = AVERAGE लगभग<br>4 = POOR ठीक नहीं थी<br>99 = CAN NOT SAY कह नहीं सकती → **GO TO E59**	
E58.	Can you tell me why you have ranked the services as ___________?<br>क्या आप बता सकती हैं कि आपने सेवाओं को इस प्रकार आँकलन क्यों किया ?<br><br>**PROBE: ANY OTHERS?**<br>çkc % dN vkj\<br><br>**RECORD ALL MENTIONED**<br>lHkh mÙkj fy[kA	1 = DOCTOR ALWAYS THERE डॉक्टर हमेशा मौजूद था<br>2 =FACILITY ALWAYS OPEN स्वास्थ्य सुविधा/केंद्र हमेशा खुली थी<br>3 = FACILITY ALWAYS HAS NECESSARY MEDICINES स्वास्थ्य सुविधा में हमेशा जरूरी दवायें उपलब्ध थीं<br>4 = NOT A LONG WAIT लंबे समय तक इंतजार नहीं करना पड़ा<br>5 = STAFF TREAT WOMEN WITH RESPECT कर्मचारी महिलाओं के साथ सम्मान के साथ पेश आते थे<br>6 = OFTEN DOCTOR NOT THERE अक्सर डॉक्टर नहीं होता था<br>7 = OFTEN FACILITY IS CLOSED अक्सर स्वास्थ्य केंद्र बंद होता था<br>8 = FACILITY DOES NOT HAVE NECESSARY MEDICINES स्वास्थ्य सुविधा में जरूरी दवायें नहीं मिलती थीं<br>9 = LONG WAIT TO BE SEEN लंबे समय तक इंतजार करना पड़ता था<br>10 = STAFF TREAT WOMEN POORLY कर्मचारी महिलाओं के साथ बुरा व्यवहार करते थे	

		11 = NO ELECTRICITY IN FACILITY स्वास्थ्य केन्द्र मे बिजली की सुविधा नहीं थी<br>12 = NO SPACE TO STAY FOR ACCOMPANYING PERSON साथ आने वाले व्यक्ति के लिए ठहरने की कोई जगह नहीं थी<br>13 = NO STAFF STAYS AT NIGHT IN THE FACILITY<br>स्वास्थ्य केंद्र में कोई कर्मचारी रात को नहीं रहता था<br>14 = NO LATRINE OR WATER FACILITY शौचालय या पानी की सुविधा नहीं थी<br>15 = PRIVACY NOT MAINTAINED कोई गोपनीयता नहीं थी<br>16 = QUALITY OF FOOD/FOOD ARRANGEMENT WAS POOR भोजन की गुणवत्ता/भोजन का इंतजाम खराब था<br>17= FACILITY IS DIRTY स्वास्थ्य सुविधा में गंदगी है<br>88 = OTHERS (SPECIFY) अन्य (स्पष्ट करें)<br>99 = DO NOT KNOW पता नहीं
E59.	After you returned home from the facility post delivery, did you visit anyone for your check-up?<br>प्रसव के बाद स्वास्थ्य सुविधा से घर लौटने के बाद, क्या आप अपनी जाँच कराने किसी के पास गयी थी?	1 = YES हाँ<br>0 = NO नहीं → **GO TO** E62
E60.	How long after you returned home from the facility, did you go for your checkup for the first time?<br>प्रसव के बाद स्वास्थ्य सुविधा से घर लौटने के बाद, अपनी जाँच कराने किसी के पास आप पहली बार कब गयी थी?	☐ ☐☐<br>1 = DAYS दिन<br>2 = WEEKS सप्ताह<br>3 = MONTHS महीने
E61.	Where did you go for the first checkup?<br>प्रसव के बाद आप पहली बार अपनी जाँच कराने कहाँ गयी थी?	1 = GOVERNMENT/MUNICIPAL HOSPITAL सरकारी /नगरपालिका का अस्पताल<br>2 = GOVERNMENT DISPENSARY सरकारी दवाखाना<br>3 = UHC/UHP/UFWC<br>यूएचसी/यूएचपी/यूएफडब्ल्यूसी<br>4 = CHC सीएचसी<br>5 = APHC/NPHC/BPHC<br>एपीएचसी/एनपीएचसी/बीपीएचसी<br>6 = SUB-CENTER उपकेन्द्र<br>7 = ANGANWADI CENTER आँगनवाड़ी केन्द्र<br>8 = VHND ग्राम स्वास्थ्य एवं पोष्ण दिवस<br>9 = NGO HOSPITAL/CLINIC गैर सरकारी संस्था का अस्पताल/क्लीनिक<br>10 = PRIVATE HOSPITAL/CLINIC प्राइवेट अस्पताल/क्लीनिक<br>11 = MEDICINE SHOP दवा की दुकान<br>88 = OTHERS (SPECIFY) अन्य (स्पष्ट करें)
E62.	After you returned home from the facility post delivery, did you visit anyone for check-up of the [CHILD NAME]?<br>प्रसव के बाद स्वास्थ्य सुविधा से घर लौटने के बाद, क्या आप (बच्चे का नाम) की जाँच कराने किसी के पास गयी थी?	1 = YES हाँ<br>0 = NO नहीं → **GO TO E65**
E63.	How long after you returned home from the facility, did you go for the checkup of [CHILD NAME] for the first time?<br>प्रसव के बाद स्वास्थ्य सुविधा से घर लौटने के बाद, (बच्चे का नाम) की जाँच कराने किसी के पास आप पहली बार कब गयी थी?	☐ ☐☐<br>1 = DAYS दिन<br>2 = WEEKS सप्ताह<br>3 = MONTHS महीने
E64.	Where did you go for the first checkup of [CHILD NAME]?<br>प्रसव के बाद आप पहली बार (बच्चे का नाम) की जाँच कराने कहाँ गयी थी?	1 = GOVERNMENT/MUNICIPAL HOSPITAL सरकारी /नगरपालिका का अस्पताल<br>2 = GOVERNMENT DISPENSARY सरकारी दवाखाना<br>3 = UHC/UHP/UFWC<br>यूएचसी/यूएचपी/यूएफडब्ल्यूसी<br>4 = CHC सीएचसी<br>5 = APHC/NPHC/BPHC<br>एपीएचसी/एनपीएचसी/बीपीएचसी

		6 = SUB-CENTER उपकेन्द्र<br>7 = ANGANWADI CENTER आँगनवाड़ी केन्द्र<br>8 = VHND ग्राम स्वास्थ्य एवं पोषण दिवस<br>9 = NGO HOSPITAL/CLINIC गैर सरकारी संस्था का अस्पताल/क्लीनिक<br>10 = PRIVATE HOSPITAL/CLINIC प्राइवेट अस्पताल/क्लीनिक<br>11 = MEDICINE SHOP दवा की दुकान<br>88 = OTHERS (SPECIFY) अन्य (स्पष्ट करें)
E65.	Did the ASHA come to your home to check on you or [CHILD NAME] within 24 hours after you returned home from the facility?<br>प्रसव के बाद स्वास्थ्य सुविधा से घर लौटने के बाद 24 घंटे के अन्दर क्या आशा आपकी या आपके (बच्चे का नाम) की जाँच करने आपके घर आई थी?	1 = YES हाँ<br>0 = NO नहीं<br>99 = DO NOT KNOW पता नहीं
E66.	Did the AWW come to your home to check on you or [CHILD NAME] within 24 hours after you returned home from the facility?<br>प्रसव के बाद स्वास्थ्य सुविधा से घर लौटने के बाद 24 घंटे के अन्दर क्या आँगनवाड़ी कार्यकर्ता आपकी या आपके (बच्चे का नाम) की जाँच करने आपके घर आई थी?	1 = YES हाँ<br>0 = NO नहीं<br>99 DO NOT KNOW पता नहीं
**ASK E67-E74 ONLY TO THOSE WHO HAD A HOME DELIVERY [E1=1]**		
E67.	After the delivery, did you visit anyone for your check-up?<br>प्रसव के बाद क्या आप अपनी जाँच कराने किसी के पास गयी थी?	1 = YES हाँ<br>0 = NO नहीं → **GO TO E70**
E68.	How long after delivery did you go for the first check up?<br>प्रसव के कितने समय बाद आप पहली बार अपनी जाँच कराने किसी के पास गयी थी?	☐ ☐☐<br>1 = HOURS घंटे<br>2 = DAYS दिन<br>3 = WEEKS सप्ताह<br>4 = MONTHS महीने<br>99 = DO NOT KNOW पता नहीं
E69.	Where did you go for the first checkup?<br>आप पहली बार अपनी जाँच कराने कहाँ गयी थी?	1 = GOVERNMENT/MUNICIPAL HOSPITAL सरकारी /नगरपालिका का अस्पताल<br>2 = GOVERNMENT DISPENSARY सरकारी दवाखाना<br>3 = UHC/UHP/UFWC यूएचसी/यूएचपी/यूएफडब्ल्यूसी<br>4 = CHC सीएचसी<br>5 = APHC/NPHC/BPHC एपीएचसी/एनपीएचसी/बीपीएचसी<br>6 = SUB-CENTER उपकेन्द्र<br>7 = ANGANWADI CENTER आँगनवाड़ी केन्द्र<br>8 = VHND ग्राम स्वास्थ्य एवं पोषण दिवस<br>9 = NGO HOSPITAL/CLINIC गैर सरकारी संस्था का अस्पताल/क्लीनिक<br>10 = PRIVATE HOSPITAL/CLINIC प्राइवेट अस्पताल/क्लीनिक<br>11 = MEDICINE SHOP दवा की दुकान<br>88 = OTHERS (SPECIFY) अन्य (स्पष्ट करें)
E70.	After the delivery, did you visit anyone for checkup of the [CHILD NAME]?<br>प्रसव के बाद क्या आप (बच्चे का नाम) की जाँच करने किसी के पास गयी थी?	1 = YES हाँ<br>0 = NO नहीं → **GO TO E73**
E71.	How long after delivery, did the first checkup of [CHILD NAME] take place?<br>प्रसव के कितनी समय बाद पहली बार (बच्चे का नाम) की जाँच हुई थी?	☐ ☐☐<br>1 = HOURS घंटे<br>2 = DAYS दिन<br>3 = WEEKS सप्ताह<br>4 = MONTHS महीने<br>99 = DO NOT KNOW पता नहीं
E72.	Where did you go for the first checkup of [CHILD NAME]?<br>प्रसव के बाद आप पहली बार (बच्चे का नाम) की जाँच कराने कहाँ गयी थी?	1 = GOVERNMENT/MUNICIPAL HOSPITAL सरकारी /नगरपालिका का अस्पताल<br>2 = GOVERNMENT DISPENSARY सरकारी दवाखाना<br>3 = UHC/UHP/UFWC यूएचसी/यूएचपी/यूएफडब्ल्यूसी<br>4 = CHC सीएचसी

		5 = APHC/NPHC/BPHC एपीएचसी / एनपीएचसी / बीपीएचसी<br>6 = SUB-CENTER उपकेन्द्र<br>7 = ANGANWADI CENTER आँगनवाड़ी केन्द्र<br>8 = VHND ग्राम स्वास्थ्य एवं पोषण दिवस<br>9 = NGO HOSPITAL/CLINIC गैर सरकारी संस्था का अस्पताल / क्लीनिक<br>10 = PRIVATE HOSPITAL/CLINIC प्राइवेट अस्पताल / क्लीनिक<br>11 = MEDICINE SHOP दवा की दुकान<br>88 = OTHERS (SPECIFY) अन्य (स्पष्ट करें)
E73.	Did the ASHA come to your home to check on you or [CHILD NAME] within 24 hours after your delivery?<br>प्रसव के बाद 24 घंटे के अन्दर क्या आशा आपकी या आपके (बच्चे का नाम) की जाँच करने आपके घर आई थी?	1 = YES हाँ<br>0 = NO नहीं<br>99 = DO NOT KNOW पता नहीं
E74.	Did the AWW come to your home to check on you or [CHILD NAME] within 24 hours after your delivery?<br><br>प्रसव के बाद 24 घंटे के अन्दर क्या AWW आपकी या आपके (बच्चे का नाम) की जाँच करने आपके घर आई थी?	1 = YES हाँ<br>0 = NO नहीं<br>99 = DO NOT KNOW पता नहीं
**ASK E75-E88 TO ALL WOMEN**		
E75.	How many times did the ASHA visit you at home during the <u>first week</u> after you gave birth to [CHILD NAME]?<br>(बच्चे का नाम) के जन्म के बाद पहले सप्ताह मे आशा आपके घर कितनी बार आयी थी?	☐☐ NO. OF TIMES कितनी बार<br>99 = DO NOT KNOW/DO NOT REMEMBER पता नहीं / याद नहीं
E76.	How many times did the ASHA visit you at home during the <u>first month</u> after you gave birth to [CHILD NAME]?<br>(बच्चे का नाम) के जन्म के बाद पहले महीने मे आशा आपके घर कितनी बार आयी थी?<br>**INCLUDE THE VISITS MADE IN THE FIRST WEEK.**<br>igy: llrkg dh eykdkrk dk Hkh 'kkfey djA	☐☐ NO. OF TIMES कितनी बार<br>98 = NOT APPLICABLE लागू नहीं<br>99 = DO NOT KNOW/DO NOT REMEMBER पता नहीं / याद नहीं
E77.	How many times did an ASHA visit you at home during the <u>first two months</u> after you gave birth to [CHILD NAME]?<br>(बच्चे का नाम) के जन्म के बाद पहले दो महीनें मे आशा आपके घर कितनी बार आयी थी?<br>**INCLUDE THE VISITS MADE IN THE FIRST MONTH.**<br>igy: eghu: dh eykdkrk dk Hkh 'kkfey djA	☐☐ NO. OF TIMES कितनी बार<br>98 = NOT APPLICABLE लागू नहीं<br>99 = DO NOT KNOW/DO NOT REMEMBER पता नहीं / याद नहीं
E78.	How many times did an ASHA visit you at home during the <u>first three months</u> after you gave birth to [CHILD NAME]?<br>(बच्चे का नाम) के जन्म के बाद पहले तीन महीनें मे आशा आपके घर कितनी बार आयी थी?<br>**INCLUDE THE VISITS MADE IN THE FIRST AND SECOND MONTH.**<br>igy: vkj nlj: eghu: dh eykdkrk dk Hkh 'kkfey djA	☐☐ NO. OF TIMES कितनी बार<br>98 = NOT APPLICABLE लागू नहीं<br>99 = DO NOT KNOW/DO NOT REMEMBER पता नहीं / याद नहीं
E79.	How many times did the AWW visit you at home during the <u>first week</u> after you gave birth to [CHILD NAME]?<br>(बच्चे का नाम) के जन्म के बाद पहले सप्ताह मे आँगनवाड़ी कार्यकर्ता आपके घर कितनी बार आयी थी?	☐☐ NO. OF TIMES कितनी बार<br>99 = DO NOT KNOW/DO NOT REMEMBER पता नहीं / याद नहीं
E80.	How many times did the AWW visit you at home during the <u>first month</u> after you gave birth to [CHILD NAME]?<br>(बच्चे का नाम) के जन्म के बाद पहले महीने मे आँगनवाड़ी कार्यकर्ता आपके घर कितनी बार आयी थी?<br>**INCLUDE THE VISITS MADE IN THE FIRST WEEK.**<br>igy: llrkg dh eykdkrk dk Hkh 'kkfey djA	☐☐ NO. OF TIMES कितनी बार<br>98 = NOT APPLICABLE लागू नहीं<br>99 = DO NOT KNOW/DO NOT REMEMBER पता नहीं / याद नहीं
E81.	How many times did an AWW visit you at home during the <u>first two months</u> after you gave birth to [CHILD NAME]?<br>(बच्चे का नाम) के जन्म के बाद पहले दो महीनें मे आँगनवाड़ी कार्यकर्ता आपके घर कितनी बार आयी थी?<br>**INCLUDE THE VISITS MADE IN THE FIRST MONTH.**<br>igy: eghu: dh eykdkrk dk Hkh 'kkfey djA	☐☐ NO. OF TIMES कितनी बार<br>98 = NOT APPLICABLE लागू नहीं<br>99 = DO NOT KNOW/DO NOT REMEMBER पता नहीं / याद नहीं
E82.	How many times did an AWW visit you at home during the <u>first three months</u> after you gave birth to [CHILD NAME]?<br>(बच्चे का नाम) के जन्म के बाद पहले तीन महीनें मे आँगनवाड़ी कार्यकर्ता आपके घर कितनी बार आयी थी?	☐☐ NO. OF TIMES कितनी बार<br>98 = NOT APPLICABLE लागू नहीं<br>99 = DO NOT KNOW/DO NOT REMEMBER पता नहीं / याद नहीं

	**INCLUDE THE VISITS MADE IN THE FIRST AND SECOND MONTH.**<br>igy vkj nlj eghu dh eykdkrk dk Hkh 'kkfey djA			
E83.	**DO NOT ASK IF '0' IN BOTH E78 AND E82**<br><br>What were the topics discussed during any of these visits by an ASHA/AWW during the visit after [CHILD NAME] was delivered?<br>(बच्चे का नाम) के जन्म के बाद आशा/आँगनवाड़ी कार्यकर्ता/ए.एन.एम. द्वारा की गयी भेंटों के दौरान किन विषयों पर चर्चा की गयी?<br>**DO NOT READ LIST. CODE ALL MENTIONS IN FIRST COLUMN. THEN READ EACH ITEM BELOW (a-i) THAT WASN'T MENTIONED AND CODE RESPONSE IN SECOND COLUMN. IF NOT MENTIONED, SKIP TO NEXT LETTER.**<br>lph dk u i<A igy dkye e lHkh mYys[k fd;s x;s mÙkjk dk dkM djA blds ckn mu fcUnvk ¼,&vkb½ dk i<s ftudk mYys[k ugh fd;k x;k Fkk vkj nlj dkye e mÙkj dk dkM djA ;fn ugh crk;k x;k rc vxys fcUn ij tk;A	1 = YES, SPONTANEOUS<br>हाँ, स्वयं बताया	2 = YES, AFTER READING<br>हाँ, पढ़ने के बाद	0 = DID NOT RECEIVE ANY ADVICE<br>कोई सलाह नहीं मिली
a.	Exclusive breastfeeding till 6 months 6 महीने तक केवल स्तनपान	1	2	0
A1	Danger of giving water, animal milk or anything other than breastmilk for the first 6 months<br>पानी, जानवर का दूध या माँ का दूध के अलावा कुछ और देने का खतरा	1	2	0
A2.	When to start giving water and other fluids and foods to the child in addition to breastmilk<br>बच्चे को पानी और अन्य तरल पदार्थ कबसे दिया जाना चाहिए	1	2	0
b.	How to position and attach the baby for optimal breastfeeding स्तनपान कराने के लिये षिषु क छाती से लगाके रखना चाहिए	1	2	0
B1.	How to express breastmilk by hand<br>हाथ से कैसे स्तन से दूध निकलना है	1	2	0
B2.	How to assess that the child is consuming an adequate supply of breastmilk<br>बच्चा सही मात्र में माँ का दूध पि रहा है उसको कैसे आंकना	1	2	0
B3.	How to increase supply of breastmilk in the first 6 months<br>जन्म के बाद पहले ६ महीने में स्तनों में दूध की मात्रा को कैसे बढाया जाए	1	2	0
c.	Where to go for advice on breastfeeding in case of any difficulties<br>स्तनपान में कठिनाई होने पर कहाँ जान चाहिए सलाह के लिए	1	2	0
d.	How often to breastfeed स्तनपान कब–कब कराना है	1	2	0
e.	Keep naked baby on bare chest of mother and wrap with a cloth or blanket बिना कपड़ों के शिशु को माँ की खुली छाती पर रखना और कपड़े या कम्बल से ढकना	1	2	0
f.	Information on the danger signs of the mother – excessive vaginal bleeding, foul vaginal discharge, abdominal pain, or fever after birth<br>माँ के खतरे के संकेतों के बारे में जानकारी–योनि से अत्यधिक खून निकलना, योनि से बदबूदार पानी निकलना, पेट में दर्द, जन्म देने के बाद बुखार	1	2	0
g.	Information on the danger signs of the baby - having trouble breathing, being difficult to wake up, losing interest in breastfeeding, or being cold to the touch शिशु के खतरे के संकेतों के बारे में जानकारी – साँस लेने में परेशनी, उठाने या जगाने में कठिनाई, स्तनपान में रूचि न होना या छूने पर शरीर ठंडा लगना	1	2	0
h.	Not to bath baby for at least 3 days in case of normal delivery<br>सामान्य प्रसव के मामले में कम से कम तीन दिन तक बच्चे को नहलाना नहीं है	1	2	0
i.	Immunization schedule for the child बच्चे की टीकाकरण सूची	1	2	0

E84.	**(RC3)** Do you know of any danger signs or symptoms than an infant might have within the first month after delivery that would require seeking medical care?	1 = YES हाँ<br>0 = NO नहीं → **GO TO E87**

	क्या आप खतरे के कुछ ऐसे संकेतों/लक्षणों के बारे मे जानती हैं जो प्रसव के बाद पहले महीने में शिशु को हो सकते हैं और जिनके लिये चिकित्सकीय देखरेख की जरूरी होती है?	
E85.	**(RC3)** Could you name some of the danger signs or symptoms that an infant might have within the first month after delivery that would require seeking medical care?<br>क्या आप खतरे के ऐसे संकेत/लक्षण बता सकती हैं जो प्रसव के बाद पहले महीने में शिशु को हो सकते हैं और जिनके लिये चिकित्सकीय देखरेख की जरूरी होती है?<br><br>**PROBE : ANY OTHERS?**<br>çkc ½ dN vkj\<br><br>**RECORD ALL MENTIONED**<br>lHkh mÙkj fy[kA	1 = DIARRHEA दस्त<br>2 = FEVER बुखार<br>3 = COUGH खाँसी<br>4 = BREATHING FASTER THAN USUAL WITH SHORT RAPID BREATHS हल्की तीव्र साँसों के साथ सामान्य से अधिक तेजी से साँस लेना<br>5 = CHEST INDRAWING पसली चलना<br>6 = BLOCKED OR RUNNY NOSE नाक बन्द या बहना<br>7 = POOR SUCKING सही प्रकार से चूस न पाना<br>8 = BABY NOT GAINING WEIGHT बच्चे का वजन न बढ़ना<br>9 = DROWSY/LETHARGIC/DIFFICULT TO AWAKEN सुस्त उनींदा/जगाने में कठिनाई<br>10 = COLD TO TOUCH छूने पर ठंडा लगाना<br>11 = WEAK CRY OF THE BABY शिशु का धीमे से रोना<br>12 = YELLOWING OF THE SKIN त्वचा का पीला पढ़ना<br>13 = LOSS OF INTEREST IN BREASTFEEDING स्तनपान में रूचि न होना<br>14 = TETANUS ट्टिनस<br>15 = PERTUSIS काली खाँसी<br>16 = HIV/AIDS एछ.आई.वी./एडस<br>17 = MEASLES खसरा<br>18 = MALARIA मलेरिया<br>19 = INJURIES चोट<br>20 = MENINGITIS दिमागी बुखार<br>21 = PREMATURITY समयपूर्व जन्म<br>22 = CONGENITAL DISEASES जन्मजात बिमारीयाँ<br>88 = OTHERS (SPECIFY) अन्य (स्पष्ट करें)
E86.	From where did you get the information about these danger signs that an infant can have?<br>शिशु को होने वाले खतरे के इन संकेतों के बारे में आपको कहां से जानकारी मिली?<br><br>**PROBE : ANY OTHERS?**<br>çkc ½ dN vkj\<br><br>**RECORD ALL MENTIONED**<br>lHkh mÙkj fy[kA	1 = GOVERNMENT DOCTOR सरकारी डाक्टर<br>2 = PRIVATE DOCTOR प्राइवेट डाक्टर<br>3 = STAFF NURSE स्टाफ नर्स<br>4 = LHV लेडी हैल्थ विजिटर (एल.एच.वी.)<br>5 = MALE HEALTH WORKER पुरुष स्वास्थ्य कर्मी<br>6 = ANM ए.एन.एम.<br>7 = OTHER HEALTH PERSONNEL अन्य स्वास्थ्य कर्मी<br>8 = ASHA आशा<br>9 = AWW आँगनवाड़ी कार्यकर्ता<br>10 = SBA/TRAINED DAI एसबीए/प्रशिक्षित दाई<br>11 = DAI दाई<br>12 = RMP आर.एम.पी. (झोला छाप डाक्टर)<br>13 = FAMILY MEMBER परिवार का सदस्य<br>14 = FRIENDS/NEIGHBOUR मित्र/पड़ौसी<br>15 = RADIO/TV/NEWS PAPER रेडियो/टीवी/अखबार<br>16 = MOBILE SMS मोबाइल एसएमएस<br>88 = OTHERS (SPECIFY) अन्य (स्पष्ट करें)<br>98 = NO ONE कोई नहीं<br>99 = DO NOT KNOW पता नहीं
E87.	Were you told about any doctor/facility to visit in case your child experiences any danger signs?<br>बच्चे में ये संकेत दिखने पर क्या आपसे किसी डाक्टर/स्वास्थ्य सुविधा में जाने के लिये कहा गया था?	1 = YES हाँ<br>0 = NO नहीं
**E87A**	**ASK IF >3 FOR E21B**	1 = YES हाँ

	Did the baby which was born premature receive any treatment?<br>**क्या बच्चा जो समय से पहले जन्मा था, उसको कोई इलाज मिला था स्वस्थ्य केंद्र से?**	0= NO नहीं SKIP TO E87C		
**E87B**	What treatment did the child receive at the health centre?<br>**बच्चे को स्वस्थ्य केंद्र में क्या इलाज मिला?**<br><br>MULTIPLE CHOICE	1= OXYGEN SUPPORT<br>ऑक्सीजन दिया गया<br>2=IV FLUIDS<br>बोतल चढ़ाया गया<br>3=IV ANTIBIOTICS<br>एंटीबायोटिक दावा चढ़ाया गया<br>4=BLOOD TRANSFUSION<br>खून चढ़ाया गया<br>88=OTHERS<br>99=DO NOT KNOW		
E88.	**(RC3)** Did [CHILD NAME] ever suffer from any of these problems?<br>क्या (बच्चे का नाम) को इनमे से कूछ कभी भी हुआ था?	YES		NO
a.	Diarrhea दस्त	1		0
b.	Fever बुखार	1		0
c.	Cough खाँसी	1		0
d.	Breathing faster than usual with short rapid breaths हल्की तीव्र सासों के साथ सामान्य से अधिक तेजी से सांस लेना	1		0
e.	Chest indrawing पसली चलना	1		0
f.	Blocked or runny nose नाक बन्द या बहना	1		0
g.	Poor sucking सही प्रकार से चूस न पाना	1		0
h.	Baby not gaining weight बच्चे का वजन न बढ़ना	1		0
i.	Drowsy/lethargic/difficult to awaken सुस्त उनींदा/जगाने में कठिनाई	1		0
j.	Cold to touch छूने पर ठंडा लगाना	1		0
k.	Weak cry of the baby शिशु का धीमे से रोना	1		0
l.	Yellowing of the skin त्वचा का पीला पढ़ना	1		0
m.	Loss of interest in breastfeeding स्तनपान में रूचि न होना	1		0
n.	Tetanus टीटेनस	1		0
o.	Pertusis काली खांसी	1		0
q.	Measles खसरा	1		0
r.	Malaria मलेरिया	1		0
t.	Meningitis दिमागी बुखार	1		0
v.	Congenital diseases जन्मजात बिमारीयाँ	1		0
E89	**ASK ONLY IF YES IN E88a**<br>**(RC3)** What was given to [NAME] when s/he had diarrhoea the last time?<br>जब ;नामद्ध को आखिरी बार दस्त की बिमारी हुई थी तो उसे इलाज के लिए क्या दिया गया ?	Zincः ज़िंक	1	0
		ORT/ओ आर टी	1	0
E90	**ASK ONLY IF YES IN E88b OR E88c OR E88d OR E88e**<br>**(RC3)** Was [NAME] treated with an antibiotic when s/he had these symptoms the last time?<br>जब (नाम) को आखिरी बार निमोनिया की बिमारी हुईए तो क्या उसे इलाज के लिये एंटीबायोटिक दिया गया?	Yes/ हां .... 1<br>No/ नहीं .... 2<br>Don't know/ नहीं जानते........98		
E91	**ASK ONLY IF YES IN E88b OR E88c OR E88d OR E88e**<br>**(RC3)** Did the child receive injection (gentamycin) for the last episode of fever?<br>बच्चे को जब आखिरी बार बुखार हुआ क्या तब उसको जेन्टामाईसीन का इंजेक्शन दिया गया?	Yes/हां .... 1<br>No/ नहीं........2		
E91A	**ASK ONLY IF YES IN E88Q**<br>**(RC3)**Did the child receive Vitamin A for measles?<br>क्या बच्चे को खसरे के लिए विटामिन A की खुराग मिली थी?	Yes/ हां .... 1<br>No/ नहीं ....2<br>**GOTO E87c**<br>Don't know/ नहीं जानते........99 **GOTO E87c**		
E91b	**ASK ONLY IF YES IN E88Q**<br>**(RC3)** How many times did the child receive Vitamin A doses for treatment of measles?	ज़िं TIMES		

	**विटामिन A की कितनी खुराग खसरे की इलाज़ के लिए बच्चे को मिली?**	
**E87C**	During the first month after birth, did the child have sepsis/pneumonia?<br>**जन्म के पहले महीने में क्या बच्चे को सेप्सिस/निमोनिया हुआ था?**	1 = YES हाँ<br>0= NO नहीं GOTO E91F
**E87D**	Did you go for treatment to any health facility?<br>**क्या आप किसी स्वस्थ्य केंद्र में इलाज के लिए गए थे?**	1 = YES हाँ<br>0= NO नहीं GOTO E88
**E87E**	What treatment did the child receive at the health centre?<br>**बच्चे को स्वस्थ्य केंद्र में क्या इलाज मिला?**<br><br>MULTIPLE CHOICE	1= OXYGEN SUPPORT<br>ऑक्सीजन दिया गया<br>2=IV FLUIDS<br>बोतल चढ़ाया गया<br>3=IV ANTIBIOTICS<br>एंटीबायोटिक दावा चढ़ाया गया<br>4=BLOOD TRANSFUSION<br>खून चढ़ाया गया<br>88=OTHERS<br>99=DO NOT KNOW
E91F	**(RC3)ASK IF YES FOR E88-r**<br>Was the child treated with medicine for malaria?<br>**क्या बच्चे को मलेरिया की दवाई दी गयी थी?**	Yes/ हां .... 1<br>No/ नहीं .... 2<br>Don't know/ नहीं जानते........................99
E91G	**(RC3)ASK IF YES FOR E88-r**<br>How long after he first got the fever was he given the medicine?<br>**मलेरिया के बुखार के शुरू होने के कितने समय बाद उनको दवाई दी गयी थी?**	☐☐<br><br>1=HOURS<br>2=DAYS<br>99=DO NOT KNOW

F. INFANT FEEDING (RC1/2/3)

This section will be asked to the three categories, category 1, category 2, category 3, i.e. mothers of 0-23 months child.

Read out to the respondent: Now I would like to ask you some questions on the breastfeeding and complementary feeding practices regarding [CHILD NAME]

mÙkjnkrk dk i<dj lukyें: अब मैं आपसे (बच्चे का नाम) को स्तनपान कराने और ऊपर का खाना देने के सम्बन्ध में कुछ प्रश्न पूछना pkgxhA

F1.	Did you ever breastfeed [CHILD NAME]?<br>क्या (बच्चे का नाम) को आपने कभी स्तनपान कराया?	1 = YES हाँ → **GO TO F3**<br>0 = NO नहीं
F2.	Why did you never breastfeed [CHILD NAME]?<br>आपने (बच्चे का नाम) को कभी भी स्तनपान क्यों नहीं कराया?<br><br>**PROBE : ANY OTHERS?**<br>çkc % dN vkj\<br><br>**RECORD ALL MENTIONED**<br>lHkh mÙkj fy[kA<br>**AFTER RESPONDING TO THIS QUESTION, GO TO F18**<br>इस प्रश्न का उत्तर पाने के बाद एफ18 पर जायें।	1 = PROBLEM WITH BREAST (PAIN, CRACKED NIPPLES) स्तनों में समस्या उनमें दर्द उनका फटा हुआ होना<br>2 = CHILD DID NOT SUCK WELL बच्चे ने अच्छी तरह नहीं चूसा<br>3 = NOT ENOUGH TIME TO FEED स्तनपान कराने के लिये समय नहीं<br>4 = MOTHER FELT NOT ENOUGH BREAST MILK माँ को लगा कि पर्याप्त दूध नहीं आ रहा<br>5 = MOTHER WENT BACK TO WORK माँ काम पर वापस चली गयी<br>6 = INFANT RESISTED BREASTFEEDING शिशु ने स्तनपान का विरोध किया<br>7 = NO NEED TO BREASTFEED स्तनपान कराने की जरूरत नहीं<br>88 = OTHER (SPECIFY) अन्य (स्पष्ट करें)
F3.	How long after birth did you first put [CHILD NAME] to the breast?<br>जन्म के कितने समय बाद आपने (बच्चे का नाम) को स्तनों से लगाया?<br><br>**IF LESS THAN 1 HOUR, CODE '00.' IF LESS THAN 24 HOURS, RECORD HOURS. OTHERWISE, RECORD DAYS.**<br>यदि एक घंटे से कम तो '00' का कोड करें। यदि 24 घंटे से कम तो घंटे मे लिखें। नहीं तो दिनों मे लिखें।	☐ ☐☐<br>00 = IMMEDIATELY/WITHIN 1 HOUR<br>तुरन्त/1 घंटे के अन्दर<br>1 = HOURS घंटे<br>2 = DAYS दिन<br>99 = DO NOT KNOW पता नहीं
**F3A.**	**ASK IF F3<>00, ELSE GO TO F4**	1 = YES हाँ

	Did you discuss plans to place the newborn child on your breast immediately after delivery?<br>क्या आपने नवजात शिशु को जन्म के तुरंत बाद अपने स्तन पे लगाने के बारें में के बारे में अपनी सास या पति से विचार–विमर्श किया था?	0 = NO नहीं<br>= NOT APPLICABLE लागू नहीं
F4.	Did you feed [CHILD NAME] your first yellow thick milk (local term)?<br>क्या आपने (बच्चे का नाम) को अपना पहला गाढ़ा पीला दूध पिलाया था (स्थानीय शब्दों में)?	1 = YES हाँ<br>0 = NO नहीं
F5.	Did you receive any support to breastfeed [CHILD NAME] immediately after delivery?<br>क्या (बच्चे का नाम) के जन्म के तुरन्त बाद स्तनपान कराने में आपको कोई सहायता मिली थी?	1 = YES हाँ<br>0 = NO नहीं → **GO TO F7**
F6.	Who supported you to breastfeed [CHILD NAME] immediately after delivery?<br>(बच्चे का नाम) के जन्म के तुरन्त बाद स्तनपान कराने के लिए आपको किससे सहायता मिली थी?<br><br>**PROBE : ANY OTHERS?**<br>çkc \ dN vkj\<br><br>**RECORD ALL MENTIONED**<br>lHkh mÙkj fy[kA	1 = GOVERNMENT DOCTOR सरकारी डाक्टर<br>2 = PRIVATE DOCTOR प्राइवेट डाक्टर<br>3 = STAFF NURSE स्टाफ नर्स<br>4 = LHV एल.एच.वी.<br>5 = MALE HEALTH WORKER पुरुष स्वास्थ्य कर्मी<br>6 = ANM ए.एन.एम.<br>7 = OTHER HEALTH PERSONNEL अन्य स्वास्थ्य कर्मी<br>8 = ASHA आशा<br>9 = AWW आँगनवाड़ी कार्यकर्ता<br>10 = SBA/TRAINED DAI एसबीए/प्रशिक्षित दाई<br>11 = DAI दाई<br>12 = RMP आर.एम.पी. (झोला छाप डाक्टर)<br>13 = RELATIVE/FRIENDS रिश्तेदार या मित्र<br>88 = OTHER (SPECIFY) अन्य (स्पष्ट करें)
F9.	Did you or anyone else give [CHILD NAME] anything such as honey, water, tea, jaggary, ghutti before giving breast milk for the first time?<br>क्या (बच्चे का नाम) को पहली बार स्तनपान कराने से पहले आपने या किसी और ने शहद, पानी, चाय, गुड़, घुट्टी जैसा कुछ दिया था?	1 = YES हाँ<br>0 = NO नहीं<br>99 DO NOT KNOW पता नहीं
F10.	Did you or anyone else give [CHILD NAME] anything such as honey, water, tea, jaggary, ghutti other than breast milk within the first three days after birth?<br>क्या (बच्चे का नाम) को जन्म के पहले तीन दिन में आपने या किसी और ने शहद, पानी, चाय, गुड़, घुट्टी जैसा कुछ दिया था?	1 = YES हाँ<br>0 = NO नहीं<br>99 = DO NOT KNOW पता नहीं
F11.	**ASK IF '1' IN F9 OR F10**<br>;fn ,Q9 ;k ,Q10 e mÙkj 1 gk rk ;g ç'u iNA<br><br>What was [CHILD NAME] given?<br>(बच्चे का नाम) को और क्या दिया गया था?<br><br>**PROBE : ANY OTHERS?**<br>çkc \ dN vkj\<br><br>**RECORD ALL MENTIONED**<br>lHkh mÙkj fy[kA	1 = MILK OTHER THAN BREAST MILK (ANIMAL MILK) स्तनपान के अलावा (पशुओं का दूध)<br>2 = PLAIN WATER सादा पानी<br>3 = SUGAR OR GLUCOSE WATER चीनी या ग्लूकोज का पानी<br>4 = GRIPE WATER ग्राइप वाटर<br>5 = SUGAR-SALT-WATER SOLUTION चीनी–नमक का घोल<br>6 = FRUIT JUICE फलों का रस<br>7 = INFANT FORMULA/LACTOGEN शिशु फार्मूला/लैक्टोजिन<br>8 = TEA/COFEE चाय/काफी<br>9 = HONEY शहद<br>10 = JANAM GHUTTI जन्म घुट्टी<br>88 = OTHER (SPECIFY) अन्य (स्पष्ट करें)
**F11a.**	**ASK IF '1' IN F9 OR F10, ELSE GO TO F12**<br>Did you discuss plans to avoid giving any honey, janam gutti, water, other milk or anything except breastmilk to the newborn child after delivery in the first 3 days?<br>क्या आपने बच्चे को जन्म के 3 दिन तक कोई भी तरल पदार्थ न देने के बारे में अपनी सास या पति से विचार–विमर्श किया था?	1 = YES हाँ<br>0 = NO नहीं<br>= NOT APPLICABLE लागू नहीं
F12.	Are you still breastfeeding [CHILD NAME]?<br>क्या आप (बच्चे का नाम) को अभी भी स्तनपान करा रहीं हैं?	1= YES हाँ → **GO TO F15**<br>0 = NO नहीं<br>9 = NEVER BREASTFED → **GO TO F18**

F13.	ASK IF F12=0<br><br>For how many months did you breastfeed [CHILD NAME]?<br>आपने (बच्चे का नाम) को कितने महीने तक स्तनपान कराया?	☐☐ MONTHS महीने<br>99 = DO NOT KNOW पता नहीं
F14.	ASK IF F12=0<br><br>Why did you stop breastfeeding [CHILD NAME]?<br>आपने (बच्चे का नाम) से स्तपनान कराना बन्द क्यों किया?<br><br>**PROBE : ANY OTHERS?**<br>çkc % dqN vkj\<br><br>**RECORD ALL MENTIONED**<br>lHkh mÙkj fy[kA	1 = PROBLEM WITH BREAST (PAIN, CRACKED NIPPLES) स्तनों में समस्या उनमें दर्द उनका फटा हुआ होना<br>2 = CHILD DID NOT SUCK WELL बच्चे ने अच्छी तरह नहीं चूसा<br>3 = NOT ENOUGH TIME TO FEED स्तनपान कराने के लिये समय नहीं<br>4 = MOTHER FELT NOT ENOUGH BREAST MILK माँ को लगा कि पर्याप्त दूध नहीं आ रहा<br>5 = NEW INFANT BORN नये शिशु का जन्म<br>6 = MOTHER WENT BACK TO WORK माँ काम पर वापस चली गयी<br>7 = INFANT RESISTED BREASTFEEDING शिशु ने स्तनपान का विरोध किया<br>8 = NO NEED TO BREASTFEED स्तनपान कराने की जरूरत नहीं<br>9 = INFANT ALREADY GROWN UP शिशु बड़ा हो चुका है<br>10 = GOT PREGNANT गर्भवती हो गयी<br>88 = OTHER (SPECIFY) अन्य (स्पष्ट करें)
F15.	How long did you exclusively breastfeed [CHILD NAME]?<br>आपने (बच्चे का नाम) को कितने समय तक केवल अपना दूध पिलाया था यानि उसके अलावा कोई और खाने व पीने की चीज नहीं दी ?	☐☐ COMPLETED MONTHS पूर्ण महीने<br>77 = STILL EXCLUSIVELY BREASTFEEDING अभी भी केवल स्तनपान
F16.	During the time you exclusively breastfeed [CHILD NAME] did you occasionally give him/her a little water from time to time?<br>जब आप (बच्चे का नाम) को केवल स्तनपान करा रही थीं, क्या उस समय आप उसे कभी–कभी पानी भी देती थी?	1 = YES हाँ<br>0 = NO नहीं
F17.	Have you breastfed [CHILD NAME] in the past 24 hours – either day time or night time?<br>क्या आपने (बच्चे का नाम) को पिछले 24 घंटों में दिन के समय या रात को स्तनपान कराया था?	1 = YES हाँ<br>0 = NO नहीं
**F17a.**	**ASK IF YES IN F12, ELSE GO TO F18**<br>How long do you intend to breastfeed the child?<br>आप कब तक स्तनपान करेंगे?	☐☐ MONTHS महीने<br>99 = DO NOT KNOW पता नहीं
**F17b.**	**ASK IF 77 IN F15, ELSE GO TO F18**<br>How long do you intend to breastfeed the child exclusively (with no water, other milk, fluids or foods)?<br>आप कब तक स्तन पान कराने का सोच रहे है?	☐☐ MONTHS महीने<br>99 = DO NOT KNOW पता नहीं
Read out to the respondent *Now I'd like to ask you your opinion about feeding infants.*<br>mÙkjnkrk dk i<dj lquk;¡ अब मैं आपसे शिशु को खान पान के सम्बन्ध में आपके विचार जानना चाहूँगी।		
F18.	**ASK F18-F20 FOR RC1**<br>When should a mother's first milk (colostrum) be given to the new-born?<br>नवजात शिशु को माँ का पहला दूध (कोलस्ट्रम) कब दिया जाना चाहिये?	1 = IMMEDIATELY AFTER BIRTH (WITHIN ONE HOUR)<br>जन्म के तुरन्त बाद (एक घंटे के अन्दर)<br>2 = WITHIN ONE DAY एक दिन के अन्दर<br>3 = NEVER कभी नहीं<br>88 OTHER (SPECIFY) अन्य (स्पष्ट करें)<br>99= DO NOT KNOW पता नहीं
**F18a**	When should the baby be placed on the mother's breast after delivery?<br>बच्चे को माँ के छाती में कब लगाना चाहिए, प्रसव के बाद?	1 = IMMEDIATELY AFTER BIRTH (WITHIN ONE HOUR)<br>जन्म के तुरन्त बाद (एक घंटे के अन्दर)<br>2 = WITHIN ONE DAY एक दिन के अन्दर<br>3 = NEVER कभी नहीं<br>88 = OTHER (SPECIFY) अन्य (स्पष्ट करें)<br>DO NOT KNOW पता नहीं
**F19**	How long should a baby be fed only mother's milk?<br>शिशु को केवल माँ का दूध कब तक देना चाहिये?	☐☐ MONTHS महीने<br>99 = DO NOT KNOW पता नहीं

**F20**	How long do you think a mother should breastfeed her child?<br>आपके विचार से माँ को अपने बच्चे को कितने समय तक स्तनपान कराना चाहिये?	☐☐ MONTHS महीने<br>98 = AS LONG AS SHE IS ABLE TO जब तक माँ स्तनपान करा सके<br>99 = DO NOT KNOW पता नहीं	
**F22**	**ASK ONLY IF CHILD IS LESS THAN 7 COMPLETED MONTHS**<br>In past 30 days, were you ever advised by the ASHA, ANM or AWW that you should not give anything other than breast milk (not even water) to [CHILD NAME] for 6 months?<br>क्या पिछले 30 दिनों में आशा, ए.एन.एम. या आँगनवाड़ी कार्यकर्ता ने आपको यह सलाह दी कि 6 महीने तक (बच्चे का नाम) को अपने दूध के अलावा और कुछ नहीं यानी पानी भी नहीं देना है?	1 = YES हाँ<br>0 = NO नहीं	
**F23**	Did the ASHA/AWW/ANM tell you to breast feed [CHILD NAME] till 2 years?<br>क्या आशा/आँगनवाड़ी कार्यकर्ता/ए.एन.एम. ने आपको यह बताया कि (बच्चे का नाम) को 2 वर्ष तक स्तनपान कराते रहें?	1 = YES हाँ<br>0 = NO नहीं	
Read out to the respondent Now I'd like to ask you some questions on the complimentary feeding practices of [CHILD NAME].<br>mÙkjnkrk dk i<dj lquk;sa vc esa vki ls dN ¼cPps dk uke½ के खान पान के सम्बन्ध में आपसे कुछ प्रश्न पुछना चाहूँगी।			
**F24**	Now I would like to ask you about the liquids [CHILD NAME] was given in the last 24 hours.<br>अब मैं आपसे (बच्चे का नाम) को पिछले 24 घंटों में दिये गये तरल पदार्थो के बारे मे पूछना चाहती हूँ।<br><br>In the last 24 hours did [CHILD NAME] drink:<br>क्या पिछले 24 घंटों में (बच्चे का नाम) ने निम्नलिखित तरल पदार्थ पिया था?<br>**READ (a-t) BELOW AND CODE YES OR NO FOR EACH.**<br>**(a-t)** dks i<sa vkSj çR;sd ds fy;s gka ;k ugha dk dksM djsA	YESTERDAY (Both day and night)<br>कल (दिन और रात दोनों समय)<br><br>1 = YES हाँ<br>0 = NO नहीं	
a.	Breast milk माँ का दूध		
b.	Plain water/Boiled water सादा/उबला पानी		
c.	Honey शहद		
d.	Sugar/Glucose water चीनी/ग्लूकोज का पानी		
e.	Ghutti घुट्टी		
f.	Oil to clean tongue जीभ साफ करने के लिए तेल		
g.	Rice/Dal water चवल/दालों का पानी		
h.	Commercially produced infant formula milk व्यवसायिक रूप से तैयार शिशु फार्मूला दुध		
i.	Powdered/Tinned milk डब्बे का दूध		
j.	Cow/buffalo/goat milk गाय/भैस/बकरी का दूध		
k.	Fruit juice फलों का रस		
l.	Tea or coffee चाय या काफी		
m.	Sodas like Pepsi, Coke, Orange drink सोडा जैसे पेप्सी, कोक, नारंगी पेय		
n.	Clear broth/ soup सूप		
o.	Roti mixed in milk रोटी दूध मे मिलाकर		
p.	Sattu mixed in water शत्तू पानी मे मिलाकर		
q.	Think suji/halwa पतली शुजी/हलवा		
r.	Thin khichdi पतली खीचड़ी		
s.	Medicines दवाईयाँ		
t.	Other liquids/solids (SPECIFY) अन्य तरल/ठोस पदार्थ (स्पष्ट करें)		
F25A1	Did [CHILD NAME] drink anything from a bottle with a nipple in the last 24 hours?<br><br>क्या (बच्चे का नाम) ने पिछले २४ घंटे में निप्पल वाली बोतल से कुछ पिया था ?	1 = YES<br>0 = NO<br>99 = DO NOT KNOW	
**ASK F26 TO F34 IF CHILD IS MORE THAN 4 COMPLETED MONTHS, ELSE SKIP TO F35**			
**F26**	Do you give [CHILD NAME] any solid, semi-solid, mashed or soft foods to eat?<br>क्या आप (बच्चे का नाम) को खाने के लिये कोई ठोस, अर्ध ठोस, मसला हुआ या मुलायम खाना देती हैं?	1 = YES हाँ<br>0 = NO नहीं	
**F27**	Now I would like to ask you about the food [CHILD NAME] ate during the last 24 hours, either separately or combined with other foods. In the last 24 hours did [CHILD NAME] eat:	YES हाँ	NO नहीं

	अब मैं आपसे (बच्चे का नाम) ने पिछले 24 घंटों में अलग से या किसा और भोजन के साथ मिलाकर जो खाना खाया है, उसके बारे मे पूछना चाहूँगी। क्या पिछले 24 घंटों में (बच्चे का नाम) ने निम्नलिखित भोजन खाया था?<br>**READ (a-o) BELOW AND CODE YES OR NO FOR EACH.**<br>**(a-o)** dks i<s vkj ÁR;sd ds fy;s gk ;k ugh dk dksM djsA		
a.	Porridge or gruel (Rice/Khichdi) दलिया/चावल/खिचड़ी	1	0
b.	Biscuit बिस्कुट	1	0
c.	Commercially fortified baby food such as Cerelac or Farex व्यवसायिक रूप से तैयार बच्चे का फार्मूला खाना जैसे सेरेलिक्स या फारेक्स	1	0
d.	Bread, roti, chapatti ब्रेड, रोटी, चपाती	1	0
e.	Daal (Foods made with lentils or beans) दाल (खाना जो दालों या फलियों से बनी है)	1	0
f.	Pumpkin, carrots, or sweet potatoes that are yellow or orange inside कद्दु, गाजर, या शकरकंदी जो अन्दर से पीला या नारंगी हो	1	0
g.	Dark Green leafy vegetables हरी पत्तेदार सब्जियाँ	1	0
h.	Ripe mangoes or ripe papayas पके आम, पपीता	1	0
i.	Other fruits or vegetables (bananas,potatoes) अन्य फल और सब्जियों (केला, आलू)	1	0
j.	Meat/chicken/fish माँस चिकन, मछली	1	0
k.	Egg अण्डा	1	0
l.	Nuts अखरोट, बादाम, आदि	1	0
m.	Salty purchased snacks foods (chips, kurkure, namkeen,) बाजार से खरीदा गया नमकीन स्नैक्स जैसे चिप्स, कुरकुरे, नमकीन	1	0
n.	Sweet purchased snacks (chocolate, candies) बाजार से खरीदा गया मीठे स्नैक्स जैसे चाकलेट, टाफी	1	0
o.	Other solids (SPECIFY) अन्य ठोस आहार (स्पष्ट करें)	1	0

**F28**	From what age did you start feeding [CHILD NAME] solid, semi-solid, mashed or soft foods?<br>आपने (बच्चे का नाम) को ठोस, अर्ध ठोस, मसला हुआ या मुलायम खाना देना कब से शुरू किया था?	☐ COMPLETED MONTHS पूर्ण महीनों मे
**F29**	Excluding oil for cooking, did you add oil/ghee/butter to the food you gave [CHILD NAME] in the last 24 hours?<br>खाने पकाने के तेल के अतिरिक्त, क्या पिछले 24 घंटों में आपने (बच्चे का नाम) के भोजन में तेल/घी/मक्खन ऊपर से मिलाया है?<br>**EXCLUDE OIL USED FOR COOKING**<br>[kkus idkus ds rsy dks 'kkfey u djsA	1 = YES हाँ<br>0 = NO नहीं
**F30**	How many times did [CHILD NAME] eat solid, semi-solid, mashed or soft foods other than liquids yesterday during the last 24 hours?<br>(बच्चे का नाम) ने पिछले 24 घंटों के दौरान कितनी बार ठोस, अर्ध ठोस, मसला हुआ या मुलायम भोजन खाया जिसमें तरल पदार्थ शामिल नहीं हैं?	☐☐ TIMES कितनी बार<br>**0 → GO TO F35**

**F32**	Now think only about those meals where you/another adult fed [CHILD NAME] from a separate bowl and which included roti, rice or porridge during the last 24 hours.<br>अब उन भोजनों के बारे में सोचें जो आपने या किसी अन्य वयस्क ने (बच्चे का नाम) को पिछले 24 घंटों में खिलाया जिनमें रोटी, चावल दलिया आदि।<br>How much did you feed [CHILD NAME] in each of the meals in the last 24 hours?<br>पिछले 24 घंटों में आपने (बच्चे का नाम) को हर बार कितनी मात्रा में भोजन खिलाया?<br>**INSTRUCTION:** Request the mother to get the katori or plate that she normally uses to feed [CHILD NAME], along with some water in a separate glass/jar. Explain to the mother that we will be asking her about the different meals consumed by her child in the last 24 hours separately, starting with the last meal consumed by [CHILD NAME] in the 24 hour period.<br>Request the mother to estimate the quantity eaten by [CHILD NAME] in last meal by pouring the corresponding amount of water into katori. Confirm that [CHILD NAME] consumed the entire quantity of food offered to [CHILD NAME]. In case [CHILD NAME] did not consume the entire quantity offered, ask the mother to throw out the approximate amount of water which corresponds to the amount of food not	Food Groups<br>भोजन समूह<br>1 = FATS AND OILS वसा और तेल<br>2 = PULSES, LEGUMES AND NUTS दालें, फलिया और बादाम<br>3 = GREEN LEAFY/OTHER VEGETABLES<br>हरी पत्तेदार/अन्य सब्जियाँ<br>4 = FRUITS फल<br>5 = CEREALS, GRAINS, ROOTS AND TUBERS<br>अनाज और कन्द मूल<br>6 = MILK AND MILK PRODUCTS दूध और दूध से बने उत्पाद<br>7 = EGG अन्डा<br>8 = ANIMAL PRODUCTS (MEAT, FISH, POULTRY AND LIVER/ORGAN MEAT) और पशु उत्पाद माँस (माँस – भेंड़/बकरी/गाय/बकरी/भैंस/सूअर, मछली – बड़ी/छोटी/कवचधारी/झींगा)	F33.<br>Was [NAME] given food in a separate bowl/plate ?<br>क्या (नाम) को भोजन एक अलग कटोरी/थाली में हदया गया था?<br>1 = YES हाँ<br>0 = NO नहीं	F34. How much did [NAME] consume in this meal?<br>नाम ने भोजन की कितनी मात्रा खायी?<br>1=FULL पूरा<br>2=HALF आधा<br>3=LESS THAN HALF आधे से कम

	consumed by [CHILD NAME]. Pour this water which corresponds to the quantity of food consumed by [CHILD NAME] in the katori that you have and code F34.<br>Repeat this process for the previous meal consumed by [CHILD NAME] till all the meals consumed by [CHILD NAME] as mentioned in Error! Reference source not found. are accounted for.<br>**निर्देश : अब माँ को एक अलग ग्लास में पानी के साथ वह कटोरी** yku dk dgs ftlls og ¼cPps dk uke½ dks Hkkstu djkrh gSA eka **को स्पश्ट करें कि अब हम उससे पिछले 24 घंटों में (बच्चे का नाम)** }kjk [kk;s x;s fiNys vkgkj ls 'k# djs vkj vyx&vyx vkgkjks ds ckjs esa iNsxsA<br>eka ls dgs fd ftruk vkgkj og ¼cPps dk uke½ dks nsrh gS mruk gh ikuh dVksjh esa Mky dj cPps }kjk [kk;s x;s Hkkstu dh ek=k dk vuqeku yxk;sA ;g iqf"V Hkh djs fd D;k cPps us fn;s x;s Hkkstu dk lkjh ek=k esa Hkkstu ugha fd;k rks eka ls dgs fd og dVksjh ls yxHkx mruk gh ikuh Qsd ns ftruk Hkkstu cPps us ugha [kk;kA vc ¼cPps dk uke½ }kjk [kk;s x;s Hkkstu dh ek=k ds cjkcj ikuh viuk ikl j[kh dVksjh esa Mkys vkj ,Q33 esa dksM nsA	9 = VITAMIN A RICH FRUITS/VEGETABLES<br>विटामिन ए युक्त फल/सबजियाँ<br>10 = JAGGERY/SUGAR<br>गुड/चीनी		
		☐☐, ☐☐, ☐☐, ☐☐, ☐☐		
		☐☐, ☐☐, ☐☐, ☐☐, ☐☐		
		☐☐, ☐☐, ☐☐, ☐☐, ☐☐		
		☐☐, ☐☐, ☐☐, ☐☐, ☐☐		
		☐☐, ☐☐, ☐☐, ☐☐, ☐☐		

**F35**	During the period 5-8 months after delivery, did any ASHA visit you at home to talk to you about [CHILD NAME]?<br>प्रसव के बाद 5–8 महीनों में क्या आशा (बच्चे का नाम) के बारे में बात करने आपके घर आयी थी?	1 = YES हाँ<br>0 = NO नहीं
**F36**	Did you ever receive any Take Home Ration for [CHILD NAME] from the Anganwadi center?<br>क्या आपको कभी भी (बच्चे का नाम) के लिये आँगनवाड़ी केंद्र से पंजीरी मिली है?	1 = YES हाँ<br>0 = NO नहीं
**F37**	Did you ever receive any Take Home Ration for yourself from the Anganwadi center after delivery of [CHILD NAME]? क्या आपको (बच्चे का नाम) के जन्म के बाद अपने लिये आँगनवाड़ी केंद्र से पंजीरी मिली है?	1 = YES हाँ<br>0 = NO नहीं
**F38**	**DO NOT ASK IF NO IN F36 AND F37**<br>In the past 3 months, how many times did you receive any food/ration for yourself or [CHILD NAME] from the Anganwadi center? पिछले 3 महीनों में आपको अपने लिये या (बच्चे का नाम) के लिये आँगनवाड़ी केन्द्र से कितनी बार पंजीरी मिली भोजन प्राप्त हुआ है?	☐☐ NO. OF TIMES कितनी बार<br>78 DID NOT RECEIVE नहीं मिला
**F38A**	Did you receive Take Home Ration (THR) from the AWC/VHND in the last one month?<br>आपको पिछले एक महीने में आँगनवाड़ी केंद्र से पंजीरी मिली है?	1 = YES हाँ<br>0 = NO नहीं
Read out to the respondent *Now I'd like to ask you your opinion about feeding infants.*<br>mÙkjnkrk dk i<dj lquk;s अब मैं आपसे शिशुओं के खान पान के बोरे में आपके विचार जानना चाहूँगी।		
**F39**	At what age should a child be started on complementary feeding?<br>बच्चे को ऊपर का खाना किस आयु से दिया जाना चाहिये?	☐ COMPLETED MONTHS पूर्ण महीनों में<br>99 DO NOT KNOW पता नहीं
**F39a**	How many times a day should a child of 6 to 23 months be fed?<br>दिन में ६-23 महीने के बच्चे को कितनी बार खाना खिलाना चाहिए?	☐ ☐ Number of Times<br>99=DO NOT KNOW पता नहीं
**F39b**	How many katories per day (of 250 ml size) should a child of 6-8 months, 9-11 months and 12-23 months be fed?<br>६-८, 9-११, 12-23 महीने के बच्चे को कितने कटोरी खाना खिलाना चाहिए?	☐ ☐ 6-8 months<br>☐ ☐ 9-11 months<br>☐ ☐ 12-23 months<br>99=DO NOT KNOW पता नहीं
**F40**	**IF [CHILD NAME] LESS THAN 6 COMPLETED MONTHS, GO TO F52**<br>;fn ¼cPps dk uke½ dh vk;q 6 eghuk ls de gS **F51** ij tk;s	1 = YES हाँ<br>0 = NO नहीं → **GO TO F51**

	Did the ASHA/AWW/ANM ever talk/discuss topics related to complementary feeding with you?<br>क्या आशा/आँगनवाड़ी कार्यकर्ता/ए.एन.एम. ने ऊपर के खाना के सम्बन्ध में आपसे कभी बातचीत/चर्चा की है?	
F41.	During the last month, did the ASHA/AWW/ANM ever talk/discuss topics related to complementary feeding with you? क्या पिछले महिने में आशा/आँगनवाड़ी कार्यकर्ता/ए.एन.एम. ने ऊपर के खाना के सम्बन्ध में आपसे बातचीत/चर्चा की है?	1 = YES हाँ<br>0 = NO नहीं
F42.	During any of the visits, did the ASHA/AWW/ANM tell you when to start feeding [CHILD NAME] solid, semi-solid, mashed or soft foods?<br>क्या आशा/आँगनवाड़ी कार्यकर्ता/ए.एन.एम. ने उन मुलाकातों के दौरान आपको यह बताया कि (बच्चे का नाम) को ठोस, अर्ध ठोस, मसला हुआ या मुलायम खाना कबसे देना शुरु करना है?	1 = YES हाँ<br>0 = NO नहीं → **GO TO F44**
F43.	From which month did the ASHA/AWW/ANM tell you to start feeding [CHILD NAME] solid, semi-solid, mashed or soft foods?<br>आशा/आँगनवाड़ी कार्यकर्ता/ए.एन.एम. ने आपको (बच्चे का नाम) को कब से ठोस, अर्ध ठोस, मसला हुआ या मुलायम खाना देना शुरु करने को कहा था?	☐☐ COMPLETED MONTHS पूर्ण महीनों में<br>78 = WHEN THE CHILD STARTS TEETHING जब बच्चे के दाँत निकलने लगे<br>99 = DO NOT KNOW/REMEMBER पता नहीं/याद नहीं
F44.	Did the ASHA/AWW/ANM tell you what types of food you should feed [CHILD NAME]?<br>क्या आशा/आँगनवाड़ी कार्यकर्ता/ए.एन.एम. ने आपको यह बताया कि (बच्चे का नाम) को किस प्रकार का खाना देना हैं?	1 = YES हाँ<br>0 = NO नहीं → **GO TO F46**
F45.	What foods did the ASHA/AWW/ANM tell you to feed [CHILD NAME]?<br>आशा/आँगनवाड़ी कार्यकर्ता/ए.एन.एम. ने (बच्चे का नाम) को किस प्रकार का खाना देने को कहा था?<br><br>**PROBE : ANY OTHERS?**<br>çkc % dN vkj\<br><br>**RECORD ALL MENTIONED**<br>lHkh mÙkj fy[kA	1 = RICE/DAL/KHICHIDI चवल/दाल/खिचड़ी<br>2 = ROTI/CHAPATI रोटी/चपाती<br>3 = ANY FOOD COOKED AT HOME घर पर बनाया गया कोई भी भोजन<br>4 = VEGETABLES सब्जियाँ<br>5 = FRUITS फल<br>6 = MILK दुध<br>7 = EGGS अंडे<br>8 = MEAT/FISH/CHICKEN माँस/मछली/चिकन<br>88 = OTHERS (SPECIFY) अन्य (स्पष्ट करें)
F46.	Did the ASHA/AWW/ANM tell you how many times to feed [CHILD NAME] each day?<br>क्या आशा/आँगनवाड़ी कार्यकर्ता/ए.एन.एम. ने आपको यह बताया कि (बच्चे का नाम) को कितनी बार खाना खिलाना है?	1 = YES हाँ<br>0 = NO नहीं → **GO TO f48**
F47.	How many times did the ASHA/AWW/ANM tell you to feed [CHILD NAME] per day?<br>आशा/आँगनवाड़ी कार्यकर्ता/ए.एन.एम. ने (बच्चे का नाम) को दिन में कितनी बार खाना खिलाना को कहा था?	☐ NO. OF TIMES कितनी बार<br>99 = DO NOT KNOW/REMEMBER पता नहीं/याद नहीं
F48.	Did the ASHA/AWW/ANM advise you to feed [CHILD NAME] from your plate or to feed [CHILD NAME] out of a separate plate or bowl?<br>क्या आशा/आँगनवाड़ी कार्यकर्ता/ए.एन.एम. ने आपको (बच्चे का नाम) को अपनी प्लेट से या बच्चे का नाम) को अलग प्लेट/कटोरी से खाना खिलाना को कहा था?<br>**CODE ONE ONLY**<br>doy ,d dkM	1 = FEED CHILD FROM MY PLATE अपनी प्लेट से<br>2 = FEED CHILD FROM SEPARATE PLATE OR BOWL अलग प्लेट या कटोरी से<br>78 = ADVICE NOT GIVEN सलाह नहीं दी<br>99 = DO NOT KNOW पता नहीं
F49.	Did the ASHA/AWW/ANM tell you how many katories of food to be fed [CHILD NAME] each day?<br>क्या आशा/आँगनवाड़ी कार्यकर्ता/ए.एन.एम. ने आपको यह बताया कि (बच्चे का नाम) को प्रति दिन कितनी कटोरी खाना खिलाना है?	1 = YES हाँ<br>0 = NO नहीं → **GO TO f51**
F50.	How many katories of semi-solid/soft foods did the ASHA/AWW/ANM tell you to feed [CHILD NAME] each day?<br>आशा/आँगनवाड़ी कार्यकर्ता/ए.एन.एम. ने आपको (बच्चे का नाम) को प्रति दिन कितनी कटोरी खाना खिलाना को कहा था?	☐.☐☐ NO. OF KATORIES IN A DAY प्रति दिन कटोरियों की संख्या<br>999 = DO NOT KNOW/DO NOT REMEMBER पता नहीं/याद नहीं
F51.	During the last 1 month, did you receive any counselling/advice on age appropriate complementary feeding?<br>पिछले 1 महिने में क्या आपको आयु के अनुसार उपयुक्त ऊपरी आहार देने के बारे में कोई परामर्श/सलाह मिली है?	1 = YES हाँ<br>0 = NO नहीं
F50A	Did the ASHA/AWW/ANM tell you about installation of a handwashing facility?	1 = YES हाँ<br>0 = NO नहीं

	क्या आशा/आँगनवगड़ी कार्यकर्ता/ए.एन.एम. ने आपको यह बताया कि घर पे हाथ धोने की सुविधा होनी चाहिए?	
F50B	Did the ASHA/AWW/ANM tell you about correct disposal of child's feces?<br>क्या आशा/आँगनवाड़ी कार्यकर्ता/ए.एन.एम. ने आपको यह बताया कि के बच्चे के मॉल को सही तरीके से फेंक देना?	1 = YES हाँ<br>0 = NO नही
**F52**	Did the ASHA/AWW/ANM tell you about how to feed [CHILD NAME] during illness?<br>क्या आशा/आँगनवाड़ी कार्यकर्ता/ए.एन.एम. ने आपको यह बताया कि (बच्चे का नाम) को बीमारी के समय किस प्रकार खाना खिलाना है?<br>**INCLUDE BREASTFEDING**<br>Lrkuiku dk Hkh 'kkfey djs	1 = YES हाँ<br>0 = NO नहीं → **GO TO F53B1**
**F53**	What did the ASHA/AWW/ANM tell you?<br>आशा/आँगनवाड़ी कार्यकर्ता/ए.एन.एम. ने आपको क्या बताया था?	1 = CONTINUE FEEDING AS BEFORE पहले की तरह खाना खिलाना जारी रखें<br>2 = DECREASE FEEDING खाना खिलाना कम करें<br>3 = INCREASE FEEDING खाना खिलाना बढ़ायें<br>4 = FEED ON DEMAND माँगने पर ही खाना खिलायें<br>5 = STOP FEEDING खाना खिलाना देना बंद कर दें<br>8 = OTHERS (SPECIFY) अन्य (स्पष्ट करें)<br>9 = NOT APPLICABLE लागू नहीं
F53B1	Has [CHILD NAME] ever been weighed at the AWC/VHND?<br><br>क्या (बच्चे का नाम) का कभी भी awc/vhnd में वजन नापा गया है?	1 = YES हाँ<br>0 = NO नहीं
F53B2	Has [CHILD NAME] been weighed at the AWC/VHND IN THE LAST MONTH?<br><br>क्या (बच्चे का नाम) का पिछले एक महीने में awc/vhnd में वजन नापा गया है?	1 = YES हाँ<br>0 = NO नहीं
**F53C**	Did AWW identify [CHILD NAME] in the severe underweight (RED) category?<br>क्या आँगनवाड़ी कार्यकर्ता ने (बच्चे का नाम) की पहचान गंभीर रुप से अल्पवजन (लाल) श्रेणी में की थी?	1 = YES हाँ<br>0 = NO नहीं<br>99= DO NOT KNOW पता नहीं
**F53D**	Did the AWW give [CHILD NAME] any advice/services?<br>क्या आँगनवाड़ी कार्यकर्ता ने (बच्चे का नाम) को कोई सलाह/सेवायें दी गया थी?	1 = YES हाँ<br>0 = NO नहीं → **GO TO F53F**
**F53E**	What advice/services were given to [CHILD NAME]?<br>(बच्चे का नाम) को क्या सलाह/सेवायें दी गया थी?<br><br>**PROBE : ANY OTHERS?**<br>çkc % dN vkj\<br><br>**RECORD ALL MENTIONED**<br>IHkh mÙkj fy[kA	1 = COUNSELLING ON BREASTFEEDING स्तनपान पर सलाह<br>2 = COUNSELLING ON COMPLIMENTARY FEEDING पूरक आहार पर सलाह<br>3 = DOUBLE TAKE HOME RATION दोगुना पोषाहार<br>4 = SUPPLEMENTATION OF MICRONUTRIENTS सुक्षम पोषक तत्वों की पूर्ति<br>5 = GROWTH MONITORING AT INTERVAL OF 15 DAYS हर 15 दिन पर वृद्दी निगरानी<br>88 OTHERS (SPECIFY) अन्य (स्पष्ट करें)
**F53F**	Did any health worker/ICDS worker identify [CHILD NAME] as severely acutely malnourished (SAM) child?<br>क्या स्वास्थ्य कर्मी/आईसीडीएस कर्मी ने (बच्चे का नाम) की पहचान गंभीर रुप से कुपोषित (सूखा राग) बच्चे के रुप में की थी?	1 = YES हाँ<br>0 = NO नहीं → **GO TO F53K1**
**F53G**	Was [CHILD NAME] referred to the health centre/nutrition rehabilitation centre?<br>क्या (बच्चे का नाम) को स्वास्थ्य केन्द्र/पोषण पुनावास केन्द्र जाने के लिए कहा गया था?	1 = YES हाँ<br>0 = NO नहीं → **GO TO F53K1**
**F53H**	Was [CHILD NAME] taken to the place referred?<br>क्या (बच्चे का नाम) को रेफेर किये गए स्वस्थ्य केंद्र पर ले गए थे?	1 = YES हाँ<br>0 = NO नहीं → **GO TO F53K1**
**F53I**	Did [CHILD NAME] receive any treatment?<br>क्या (बच्चे का नाम) को कोई उपचार मिला था?	1 = YES हाँ<br>0 = NO नहीं → **GO TO F53K1**

**F53J**	What treatment did [CHILD NAME] receive?<br>(बच्चे का नाम) को क्या उपचार मिली थी?<br><br>**PROBE : ANY OTHERS?**<br>çkc ¼ dN vkj\<br><br>**RECORD ALL MENTIONED**<br>lHkh mÙkj fy[kA	1 = STAY AT NRC एन.आर.सी. पर रुके<br>2 = CHECKUP BY DOCTOR डाक्टर द्वारा जाँच<br>3 = SUPPLEMENTARY FOOD AS ADVISED BY DOCTOR/FLW पुरत आहार जो डॉक्टर/ ऐ एन एम्/आशा/aww ने बताया हो<br>88 OTHERS (SPECIFY) अन्य (स्पष्ट करें)
**F53K**	Were you counselled by health service providers on correct care and feeding practices for the malnourished child?<br>क्या बच्चे के इलाज के लिए उसे किस प्रकार का आहार देना चाहिए इस मामले में आपको सलाह मिली थी?	1 = YES हाँ<br>0= NO नहीं

F53k1	**THINKING ABOUT THE LAST VISIT**<br>Which frontline worker had visited you the last time at home?<br><br>पिछली बार आपको आशा/अनंवादी कार्यकर्ता/ऐ एन एम् में से कौन आपको मिलने आपके घर आयें?	1=ASHA<br>2=AWW<br>3=ANM<br>9=NONE
F53k2	How much time the frontline worker who visited you last spend during the session (in minutes)?<br>पिछली बार आपके घर जो कार्यकर्ता आई थी उसने कितना समय बिताया था ? (मिनट में)	[ ][ ]<br>MINUTES मिनट
F53k3	On what topics did she advise/counsel you?<br><br>आपको उसने चीज़ों के बारें में सलाह दी ?<br><br>**PROBE : ANY OTHERS?**<br>çkc ¼ dN vkj\<br><br>**RECORD ALL MENTIONED**<br>lHkh mÙkj fy[kA	1= MATERNAL NUTRITION मात्री पोषण<br>2= IFA CONSUMPTION IFA की सिरप या गोलियां लेना<br>3= DIETARY DIVERSITY FOR NUTRITION आहार बिबीधता ताकि महिला को ज़रुरत का पोषण मिले<br>4= TAKING CARE OF YOUR HEALTH DURING PREGNANCY गर्भावस्था के दौरान अपने स्वास्थ्य की देखभाल करना<br>5= GOING TO THE HOSPITAL FOR A CHECK-UP DURING PREGNANCY गर्भावस्था के दौरान जांच के लिए अस्पताल जाना<br>6= GOING TO A HEALTH FACILITY FOR DELIVERY प्रसव कराने के लिए स्वास्थ्य केंद्र या अस्पताल जाना<br>7= PLANNING IN ADVANCEFOR DELIVERY प्रसव के लिए पहले से तैयारी किस प्रकार करनी है<br>8= HOW TO TAKE CARE OF THE NEWBORN नवजाति शिशु की देखरेख कैसे करनी है<br>9=EXCLUSIVE BREASTFEEDING केवल स्तनपान<br>10= COMPLEMENTARY FEEDING पूरक आहार / उपरी आहार<br>11= IMMUNIZATION टीकाकरण<br>12= FAMILY PLANNING परिवार नियोजन<br>13= HANDWASHING AND SANITATION हाथ धोना और साफसफाई<br>88= OTHERS (SPECIFY) अन्य (स्पष्ट करें)
F53k4	Was she clear in explaining the topics?<br>क्या वह विषयों को स्पष्ट रूप से समझा पायी थी?	1 = YES हाँ<br>0 = NO नहीं
F53k5	Were you able to understand everything?<br>क्या आप सब कुछ समझ पायी थी?	1 = YES हाँ<br>0 = NO नहीं

F53k6	Was she able to answer all your queries clearly?<br>क्या वह आपके सभी सवालों का स्पष्ट रूप से उत्तर दे पायी थी?	1 = YES हाँ<br>0 = NO नहीं
F53k7	What job aid tools did she use during this most recent visit?<br>आशा/आंगनवाडी कार्यकर्ता ने सबसे हाल के इस दौरे के समय किस साधन या उपकरण का उपयोग किया?<br>**CODE ALL THAT APPLY**<br>lHkh ykxw gksus okys dksM djsa	1= PLASTIC PICTURE CARDS (describe mobile kunji cards) प्लास्टिक के पिक्चर कार्ड (मोबाइल कुंजी)<br>2= PAMPHLET पम्फलेट<br>3=KATORA/SPOON सी कटोरा/चम्मच<br>4= COPPER-T ई कॉपर–टी<br>5= MALA-D एफ माला–डी<br>88=OTHERS (SPECIFY) अन्य (बताएं)
**F53k9**	**<u>If F53k7=1 OR F53k8=1, ASK F53k9. ELSE GO TO F55.</u>**<br><br>Did you find the usage of these tools useful?<br>क्या आपने इन उपकरणों को उपयोगी पाया?	YES हाँ...1<br>NO नहीं..............0
**F53k10**	Which of these tools helped you to understand the issues explained by FLW?<br>इनमें से कौन कौन से उपकरणों से आपको अग्रणी कतार की कार्यकर्ता (एफएलडब्ल्यू) द्वारा स्पष्ट किये गये मुद्दों को समझने में मदद मिली?<br><br>**CODE ALL THAT APPLY**<br>lHkh ykxw gksus okys dksM djsa	1= PLASTIC PICTURE CARDS (describe mobile kunji cards) प्लास्टिक के पिक्चर कार्ड (मोबाइल कुंजी)<br>2= PAMPHLET पम्फलेट<br>3=KATORA/SPOON सी कटोरा/चम्मच<br>4= COPPER-T ई कॉपर–टी<br>5= MALA-D एफ माला–डी<br>88=OTHERS (SPECIFY) अन्य (बताएं)
**F54a**	Are you aware about anaemia in children?<br><br>क्या आपको बच्चों में अनेमिया/खून की कमी के बारें में पता है?	1 = YES हाँ<br>0 = NO नहीं **SKIP T0 F55**
**F54b**	**ASK IF YES FOR F54a**<br><br>What according to you causes anaemia in children?<br><br>आपके हिसाब से बच्चो में अनेमिया किस कारन से होता है?<br><br>**MULTIPLE CHOICE**	1=Not consuming Iron rich diet<br>आयरन युक्त खाना न खाने की वजह से<br>2=Anaemic mother<br>माता को खून की कमी थी<br>3=Diseases such as malaria<br>मलेरिया या अन्य कोई बिमारी की वजन से<br>4=Due to hook worm infestation<br>पेट में कीदें लग जाने पर<br>88=Others<br>अन्य<br>99= DO NOT KNOW<br>नहीं पता
**F54c**	What is the treatment for anaemia?<br>अनेमिया/खून की कमी का इलाज क्या है?<br><br>**CODE ALL THAT APPLY**<br>lHkh ykxw gksus okys dksM djsa	1= CONSUMPTION OF IRON SUPPLEMENTS/IFA SYRUP AND TABLETS<br>IFA की गोलिया या सिरप लेना<br>2= CONSUMPTION OF IRON RICH FOOD<br>आयरन युक्त आहार लेना<br>3= more breasrtfeeding<br>जयादा स्तनपान करवाना<br>88=OTHERS<br>अन्य<br>99=DO NOT KNOW<br>पता नहीं

Read out to the respondent: Now, I would like to ask you about the diseases that [CHILD NAME] had and the treatment for those diseases.

mUkjnkrk dk i<dj lquk;s vc e vki l ¼cPp dk uke½ dk gku oky jkxk vkj mu jkxk d mipkj d ckj e iNuk pkgxhA		
F55.	In the last two weeks, did [CHILD NAME] have diarrhoea (loose watery stools)?<br>क्या (बच्चे का नाम) को पिछले 2 सप्ताह में दस्त हुआ है?	1 = YES हाँ<br>0 = NO नहीं → **GO TO F6**3a
F56.	Was there any blood in [CHILD NAME's] stools?<br>क्या (बच्चे का नाम) को दस्त के साथ खुन आया था?	1 = YES हाँ<br>0 = NO नहीं
F57.	Did you seek any advice/treatment for diarrhoea when [CHILD NAME] had diarrhoea the last time?<br>जब (बच्चे का नाम) को अखरी बार दस्त हुआ था, क्या आप किसी से सलाह/उपचार लिया था?	1 = YES हाँ<br>0 = NO नहीं → **GO TO F60**
F58.	Where did you seek advice/treatment from first, when [CHILD NAME] had diarrhoea the last time?<br>जब (बच्चे का नाम) को अखरी बार दस्त हुआ था, आपने पहली बार किससे सलाह/उपचार लिया था?<br><br>**CODE ALL THAT APPLY**<br>lHkh ykxw gksu okys dkM djs	1 = GOVERNMENT/MUNICIPAL HOSPITAL सरकारी/नगरपालिका का अस्पताल<br>2 = GOVERNMENT DISPENSARY सरकारी दवाखाना<br>3 = UHC/UHP/UFWC यूएचसी/यूएचपी/यूएफडब्ल्यूसी<br>4 = CHC सीएचसी<br>5 = APHC/NPHC/BPHC एपीएचसी/एनपीएचसी/बीपीएचसी<br>6 = SUB-CENTER उपकेन्द्र<br>7 = ANGANWADI CENTER आँगनवाड़ी केन्द्र<br>8 = VHND ग्राम स्वास्थ्य एवं पोषण दिवस<br>9 = NGO HOSPITAL/CLINIC गैर सरकारी संस्था का अस्पताल/क्लीनिक<br>10 = PRIVATE HOSPITAL/CLINIC प्राइवेट अस्पताल/क्लीनिक<br>11 = MEDICINE SHOP दवा की दुकान<br>12 = FOLK HEALER ओझा/वैद्य/झाड़फूँक<br>13 = HOME REMEDIES घरेलू उपचार<br>14 = RMP आर.एम.पी. (झोला छाप डाक्टर)<br>15 = ASHA आशा<br>16 = AWC आँगनवाड़ी केन्द्र<br>88 OTHERS (SPECIFY) अन्य (स्पष्ट करें)
**F58B**	What was the reason for seeking advice or treatment from that particular source?<br><br>उस केंद्र से इलाज करवाने का क्या कारन था?<br><br>**CODE ALL THAT APPLY**<br>lHkh ykxw gksu okys dkM djs	1 = DOCTOR ALWAYS THERE डॉक्टर हमेशा मौजूद था<br>2 =FACILITY ALWAYS OPEN स्वास्थ्य सुविधा/केंद्र हमेशा खुली थी<br>3 = FACILITY ALWAYS HAS NECESSARY MEDICINES स्वास्थ्य सुविधा में हमेशा जरूरी दवायें उपलब्ध थीं<br>4 = NOT A LONG WAIT लंबे समय तक इंतजार नहीं करना पड़ा<br>5 = STAFF TREAT WOMEN WITH RESPECT कर्मचारी महिलाओं के साथ सम्मान के साथ पेश आते थे<br>6 = OFTEN DOCTOR NOT THERE अक्सर डॉक्टर नहीं होता था<br>7 = OFTEN FACILITY IS CLOSED अक्सर स्वास्थ्य केंद्र बंद होता था<br>8 = FACILITY DOES NOT HAVE NECESSARY MEDICINES स्वास्थ्य सुविधा में जरूरी दवायें नहीं मिलती थीं<br>9 = LONG WAIT TO BE SEEN लंबे समय तक इंतजार करना पड़ता था<br>10 = STAFF TREAT WOMEN POORLY कर्मचारी महिलाओं के साथ बुरा व्यवहार करते थे<br>11 = NO ELECTRICITY IN FACILITY स्वास्थ्य केन्द्र मे बिजली की सुविधा नहीं थी<br>12 = NO SPACE TO STAY FOR ACCOMPANYING PERSON साथ आने वाले व्यक्ति के लिए ठहरने की कोई जगह नहीं थी<br>13 = NO STAFF STAYS AT NIGHT IN THE FACILITY स्वास्थ्य केंद्र में कोई कर्मचारी रात को नहीं रहता था

		14 = NO LATRINE OR WATER FACILITY शौचालय या पानी की सुविधा नहीं थी<br>15 = PRIVACY NOT MAINTAINED कोई गोपनीयता नहीं थी<br>16 = QUALITY OF FOOD/FOOD ARRANGEMENT WAS POOR भोजन की गुणवत्ता/भोजन का इंतजाम खराब था<br>17= FACILITY IS DIRTY स्वास्थ्य सुविधा में गंदगी है<br>88 = OTHERS (SPECIFY) अन्य (स्पष्ट करें)<br>99 = DO NOT KNOW पता नहीं	
**F58A**	Did you seek advice/treatment from other sources when [CHILD NAME] had diarrhoea the last time?<br>जब (बच्चे का नाम) को अखरी बार दस्त हुआ था, क्या आपने किसी और से सलाह/उपचार लिया था?	1 = YES हाँ<br>0 = NO नहीं	
F59.	How many days after the diarrhoea began did you first seek advice or treatment for [CHILD NAME]?<br>(बच्चे का नाम) के दस्त शुरु होने के कितने दिन बाद आपने पहली बार सलाह/उपचार लिया था?	☐☐ NO. OF DAYS दिनों की संख्या<br>99 = DO NOT KNOW/DO NOT REMEMBER पता नहीं/याद नहीं	
F60.	What all was given to [CHILD NAME] when s/he had diarrhoea the last time?<br>पिछली बार जब (बच्चे का नाम) को दस्त हुआ था तब उसे क्या दिया गया था?<br>**READ (a-m) BELOW AND CODE YES OR NO FOR EACH.**<br>¼,&,e½ rd i<s vkj ÁR;sd d fy, gk¡ ;k ugh dk dkM djs	YES हाँ	NO नहीं
a.	Zinc tablet जस्ता (जिंक) की गोली	1	0
b.	Zinc syrup जस्ता (जिंक) की घोल	1	0
c.	IV line आई वी लाइन	1	0
d.	Injections सुई	1	0
e.	Pill or syrup antibiotic एंटीबायोटिक गोली या घोल	1	0
f.	Antimotility अन्तिमोतिलिटी	1	0
g.	Home remedy/herbal medicine घरेलू उपचार/जड़ीबुटी	1	0
h.	Non-antibiotic syrup गैर–एंटीबायोटिक सिरप	1	0
i.	Pills crushed to powder and wrapped in paper कागज मे लिपटा गोलियों का चुरा	1	0
j.	ORT ओ आर टी	1	0
k.	Other (specify) अन्य (स्पष्ट करें)	1	0
**F61a.**	What was the child fed when he/she had diarrhea?<br>बच्चे को क्या खिलाया गया था जब उसको दस्त हुआ था?<br><br>**CODE ALL THAT APPLY**<br>lHkh ykxw gksus okys dksM djs	1=Breastmilk स्तनपान<br>2=Yellow or orange fruits or vegetables (e.g. Pumpkin, carrots, ripe mango, ripe papaya) पीली या नारंगी फल और सब्जी<br>3=Lentils, dal दल<br>4=Roti, bread or rice रोटी, चावल, ब्रेड<br>5=Eggs अन्दा<br>6=Meat, chicken or fish मॉस<br>7=Milk, paneer or yogurt दूध, पनीर, दही<br>88=Othersअन्य	
F61.	**ASK IF F61A CODED 1**<br>During the time [CHILD NAME] had diarrhoea, was he/she offered the same amount of breastfeeding as earlier?<br>जब (बच्चे का नाम) को दस्त हुआ था, क्या उस समय उसे उतना ही स्तनपान कराया गया था जितना उसे पहले कराया जाता था?	1 = MORE THAN USUAL सामान्य से अधिक<br>2 = SAME AS USUAL सामान्य जितना<br>3 = LESS THAN USUAL सामान्य रूप से<br>4 = DID NOT BREASTFEED स्तनपान नहीं कराया<br>9 = NOT APPLICABLE लागू नहीं	
F62.	**ASK IF F61A CODED >1**<br>During the time [CHILD NAME] had diarrhoea, was he/she offered the same amount of food as earlier?<br>जब (बच्चे का नाम) को दस्त हुआ था, क्या उस समय उसे उतना ही खाना दिया गया था जितना उसे पहले दिया जाता था?	1 = MORE THAN USUAL सामान्य से अधिक<br>2 = SAME AS USUAL सामान्य जितना<br>3 = LESS THAN USUAL सामान्य रूप से<br>4 = DID NOT GIVE FOOD खाना नहीं दिया<br>9 = NOT APPLICABLE लागू नहीं	
F63.	Does [CHILD NAME] still have diarrhoea?<br>क्या (बच्चे का नाम) को अभी भी दस्त है?	1 = YES हाँ<br>0 = NO नहीं	
**F63A**	How many times did [CHILD NAME] have diarrhoea in the last 3 months?<br>पिछले 3 महीनों में (बच्चे का नाम) को कितनी बार दस्त हुए थे ?	☐ NO. OF TIMES कितनी बार	
Read out to the respondent: Now, I would like to ask you some questions about Pneumonia.			

mUkjnkrk dk i<dj lquk;k vc eSa vkils dqN ç'u fuEufyf[kr ds ckjs esa iNwaxh		
F67.	Has [CHILD NAME] been ill with a fever at any time in the last 2 weeks?<br>क्या पिछले 2 सप्ताह में (बच्चे का नाम) बुखार आने से बीमार पड़ा है?	1 = YES हाँ<br>0 = NO नहीं
F68.	Has [CHILD NAME] been ill with a cough or other breathing difficulty at any time in the last 2 weeks?<br>क्या पिछले 2 सप्ताह में (बच्चे का नाम) खाँसी या साँस लेने मे समस्या के साथ बीमार पड़ा है?	1 = YES हाँ<br>0 = NO नहीं
F69.	When [CHILD NAME] had an illness, did he/she breathe faster than usual with short, rapid breaths, have difficulty breathing or chest in drawings?<br>क्या (बच्चे का नाम) सामान्य से अधिक तेजी के साथ साँस लेता था, जोर जोर से साँस लेता था, साँस लेने में परेशानी थी या उसकी पसली चल रही थी?	1 = YES हाँ<br>0 = NO नहीं<br>**IF NO TO F67 & F68 & F69 GO TOF79**
F70.	When [CHILD NAME] had this illness, did he/she have a problem in the chest or a blocked or running nose?<br>क्या जब (बच्चे का नाम) बीमार था तब उसे छाती में कोई समस्या थी या उसकी नाक बंद थी या नाक बह रही थी?	1 = YES हाँ<br>0 = NO नहीं
F71.	Did you seek any advice/treatment for this illness when [CHILD NAME] had the illness the last time?<br>जब (बच्चे का नाम) को अखरी बार दस्त बीमारी हुई थी, क्या आप किसी से सलाह/उपचार लिया था?	1 = YES हाँ<br>0 = NO नहीं → **GO TO F74**
F72.	Where did you seek advice/treatment from first, when [CHILD NAME] had this illness the last time?<br>जब (बच्चे का नाम) को अखरी बार बीमारी हुई थी, आपने पहली बार किससे सलाह/उपचार लिया था?<br><br>**CODE ALL THAT APPLY**<br>lHkh ykxw gksus okys dksM djs	1 = GOVERNMENT/MUNICIPAL HOSPITAL सरकारी/नगरपालिका का अस्पताल<br>2 = GOVERNMENT DISPENSARY सरकारी दवाखाना<br>3 = UHC/UHP/UFWC यूएचसी/यूएचपी/यूएफडब्ल्यूसी<br>4 = CHC सीएचसी<br>5 = APHC/NPHC/BPHC एपीएचसी/एनपीएचसी/बीपीएचसी<br>6 = SUB-CENTER उपकेन्द्र<br>7 = ANGANWADI CENTER आँगनवाड़ी केन्द्र<br>8 = VHND ग्राम स्वास्थ्य एवं पोष्ण दिवस<br>9 = NGO HOSPITAL/CLINIC गैर सरकारी संस्था का अस्पताल/क्लीनिक<br>10 = PRIVATE HOSPITAL/CLINIC प्राइवेट अस्पताल/क्लीनिक<br>11 = MEDICINE SHOP दवा की दुकान<br>12 = FOLK HEALER ओझा/वैद्य/झाड़फूँक<br>13 = HOME REMEDIES घरेलू उपचार<br>14 = RMP आर.एम.पी. (झोला छाप डाक्टर)<br>15 = ASHA आशा<br>16 = AWC आँगनवाड़ी केन्द्र<br>88 OTHERS (SPECIFY) अन्य (स्पष्ट करें)
**F72A**	What was the reason for seeking advice or treatment from that particular source?<br><br>उस केंद्र से इलाज करवाने का क्या कारन था?	1 = DOCTOR ALWAYS THERE डॉक्टर हमेशा मौजूद था<br>2 =FACILITY ALWAYS OPEN स्वास्थ्य सुविधा/केंद्र हमेशा खुली थी<br>3 = FACILITY ALWAYS HAS NECESSARY MEDICINES स्वास्थ्य सुविधा में हमेशा जरूरी दवायें उपलब्ध थीं<br>4 = NOT A LONG WAIT लंबे समय तक इंतजार नहीं करना पड़ा<br>5 = STAFF TREAT WOMEN WITH RESPECT कर्मचारी महिलाओं के साथ सम्मान के साथ पेश आते थे<br>6 = OFTEN DOCTOR NOT THERE अक्सर डॉक्टर नहीं होता था<br>7 = OFTEN FACILITY IS CLOSED अक्सर स्वास्थ्य केंद्र बंद होता था<br>8 = FACILITY DOES NOT HAVE NECESSARY MEDICINES स्वास्थ्य सुविधा में जरूरी दवायें नहीं मिलती थीं<br>9 = LONG WAIT TO BE SEEN लंबे समय तक इंतजार करना पड़ता था

		10 = STAFF TREAT WOMEN POORLY कर्मचारी महिलाओं के साथ बुरा व्यवहार करते थे<br>11 = NO ELECTRICITY IN FACILITY स्वास्थ्य केन्द्र मे बिजली की सुविधा नहीं थी<br>12 = NO SPACE TO STAY FOR ACCOMPANYING PERSON साथ आने वाले व्यक्ति के लिए ठहरने की कोई जगह नहीं थी<br>13 = NO STAFF STAYS AT NIGHT IN THE FACILITY<br>स्वास्थ्य केंद्र में कोई कर्मचारी रात को नहीं रहता था<br>14 = NO LATRINE OR WATER FACILITY शौचालय या पानी की सुविधा नहीं थी<br>15 = PRIVACY NOT MAINTAINED कोई गोपनीयता नहीं थी<br>16 = QUALITY OF FOOD/FOOD ARRANGEMENT WAS POOR भोजन की गुणवत्ता/भोजन का इंतजाम खराब था<br>17= FACILITY IS DIRTY स्वास्थ्य सुविधा में गंदगी है<br>88 = OTHERS (SPECIFY) अन्य (स्पष्ट करें)<br>99 = DO NOT KNOW पता नहीं	
**F72B**	Did you seek advice/treatment from other sources when [CHILD NAME] had this illness the last time?<br>जब (बच्चे का नाम) को अखरी बार बीमारी हुई थी, क्या आपने किसी और से सलाह/उपचार लिया था?	1 = YES हाँ<br>0 = NO नहीं → **GO TO F73**	
**F72C**	Where did you seek advice or treatment for this illness for [CHILD NAME]?<br>आपने (बच्चे का नाम) की बीमारी के समय किन स्रोतों से सलाह/उपचार लिया था?<br><br>**PROBE : ANY OTHERS?**<br>çkc % dN vkj\<br><br>**RECORD ALL MENTIONED**<br>lHkh mÙkj fy[kA	1 = GOVERNMENT/MUNICIPAL HOSPITAL सरकारी/नगरपालिका का अस्पताल<br>2 = GOVERNMENT DISPENSARY सरकारी दवाखाना<br>3 = UHC/UHP/UFWC<br>यूएचसी/यूएचपी/यूएफडब्ल्यूसी<br>4 = CHC सीएचसी<br>5 = APHC/NPHC/BPHC<br>एपीएचसी/एनपीएचसी/बीपीएचसी<br>6 = SUB-CENTER उपकेन्द्र<br>7 = ANGANWADI CENTER आँगनवाड़ी केन्द्र<br>8 = VHND ग्राम स्वास्थ्य एवं पोषण दिवस<br>9 = NGO HOSPITAL/CLINIC गैर सरकारी संस्था का अस्पताल/क्लीनिक<br>10 = PRIVATE HOSPITAL/CLINIC प्राइवेट अस्पताल/क्लीनिक<br>11 = MEDICINE SHOP दवा की दुकान<br>12 = FOLK HEALER ओझा/वैद्य/झाड़फूँक<br>13 = HOME REMEDIES घरेलू उपचार<br>14 = RMP आर.एम.पी. (झोला छाप डाक्टर)<br>15 = ASHA आशा<br>16 = AWC आँगनवाड़ी केन्द्र<br>88 = OTHERS (SPECIFY) अन्य (स्पष्ट करें)	
F73.	How many days after the illness began did you first seek advice or treatment for [CHILD NAME]?<br>(बच्चे का नाम) की बीमारी शुरु होने के कितने दिन बाद आपने पहली बार सलाह/उपचार लिया था?	☐☐ NO. OF DAYS दिनों की संख्या	
F74.	At any time during the illness, did [CHILD NAME] take any drugs for the illness?<br>बीमारी के दौरान क्या (बच्चे का नाम) को कोई दवाई दी गई थी?	1 = YES हाँ<br>0 = NO नहीं → **GO TO F76**	
F75.	What drugs did [CHILD NAME] take?<br>(बच्चे का नाम) को क्या दवाईयें दी गई?	YES हाँ	NO नहीं
a.	Antibiotic Pill एंटीबायोटिक गोली	1	0
b.	Antibiotic Syrup एंटीबायोटिक घोल	1	0
c.	Injection सुई	1	0
d.	Dispersible Tablets घुलने वाली गोलियाँ	1	0
e.	IV Fluid आई वी तरल	1	0
f.	Nebuliser नेबुलाइजर	1	0
g.	Oxygen आक्सीजन	1	0

**F75A**	**IF F75(a-d) = 1, ASK**<br><br>Was it Amoxicillin antibiotic?<br>क्या वह अमोक्सिसिल्लिन एंटीबायोटिक था?	1 = YES (INJ./SYRUP/TAB SEEN) हाँ, (सुई, घोल, गोली देखा)<br>2 = YES (PRESCRIPTION SEEN) हाँ, (पर्चा देखा)<br>0 = NO INFORMATION कोई जानकारी नहीं
F76.	During the time [CHILD NAME] was ill, was he/she offered the same amount of breastfeeding as earlier?<br>जब (बच्चे का नाम) बीमार था, क्या उस समय उसे उतना ही स्तनपान कराया गया था जितना उसे पहले कराया जाता था?	1 = MORE THAN USUAL सामान्य से अधिक<br>2 = SAME AS USUAL सामान्य जितना<br>3 = LESS THAN USUAL समान्य से कम<br>4 = DID NOT BREASTFEED स्तनपान नहीं कराया<br>9 = NOT APPLICABLE लागू नहीं
F77.	During the time [CHILD NAME] was ill, was he/she offered the same amount of food as earlier?<br>जब (बच्चे का नाम) बीमार था, क्या उस समय उसे उतना ही खाना दिया गया था जितना उसे पहले दिया जाता था?	1 = MORE THAN USUAL सामान्य से अधिक<br>2 = SAME AS USUAL सामान्य जितना<br>3 = LESS THAN USUAL समान्य से कम<br>4 = DID NOT GIVE FOOD खाना नहीं दिया<br>9 = NOT APPLICABLE लागू नहीं
F78.	Does [CHILD NAME] still have fever or cough or difficulty in breathing or chest in drawing?<br>क्या (बच्चे का नाम) को अभी भी बुखार, खाँसी या साँस लेने मे समस्या या उसकी पसली चल रही है?	1 = YES हाँ<br>0 = NO नहीं
**F78A**	How many times did [CHILD NAME] have this illness in the last 3 months?<br>पिछले 3 महीनों में (बच्चे का नाम) को कितनी बार बीमार हुई थी?	☐ NO. OF TIMES कितनी बार

F80.	Was [CHILD NAME] screened by a medical doctor for any of the following :<br>क्या निम्नलिखित में से किसी के लिए चिकित्सक ने (बच्चे का नाम) की जाँच की थी?	YES हाँ	NO नहीं
a.	Birth defects जन्म संबंधी दोश	1	0
b.	Development delays विकास में देरी	1	0
c.	Deficiency कोई कमी या नुक्स	1	0
d.	Disease रोग	1	0
F80e	Has the child been identified as anaemic by any ASHA/AWW/NURSE/DOCTOR?<br>क्या बच्चे को खून की कमी है/अनेमिया है, ऐसा आपको बताया गया है?	1 = YES हाँ<br>0 = NO नहीं	

Now we would like to talk about services received by you over the past couple of months from the health service providers

F80A6A	How many times did an ASHA/AWW visit you in the last three months?<br>पिछले 3 महीने में कितनी बार आशा या अनगंवादी कार्यकर्ता आपसे मिलने आई?	☐☐ASHA<br>☐☐AWW
F80A7	How many times did an ASHA/AWW visit you in the last one month?<br>पिछले एक महीने में कितनी बार आशा या अनगंवादी कार्यकर्ता आपसे मिलने आई?	☐☐ASHA<br>☐☐AWW
F80A8	In the last one month, what counselling/advice did you receive from ASHA or AWW?<br><br>**PROBE : ANY OTHERS?**<br>çkc ¶ dN vkj\<br><br>**RECORD ALL MENTIONED**<br>I Hkh mÙkj fy[kA	1= MATERNAL NUTRITION मात्री पोषण<br>2=VARIETY OF FOOD TO EAT खाने के लिए अलग अलग प्रकार के आहार<br>3=AMOUNT OF FOOD TO EAT खाने की मात्रा<br>4=HOW MANY IFA TABLETS TO CONSUME IFA की कितनी गोली लेना<br>5=BENEFITS OF CONSUMING IFA TABLETS IFA की गोलियों की जरूरत<br>6=HOW MANY CALCIUM TABLETS TO CONSUME कैल्शियम की कितनी गोलिया लेना<br>7=BENEFITS OF CONSUMING CALCIUM TABLETS केलसियम की गोलियों की जरूरत<br>8= TO ASSESS IF CHILD IS GETTING ENOUGH BREASTMILK बच्चा सही मात्र में माँ का दूध पि रहा है उसको कैसे आंकना

		9= HOW TO INCREASE BREASTMILK SUPPLY दूध की मात्र किस प्रकार से बढाया जाए<br>10= MANAGING BREAST PROBLEMS LIKE ENGORGEMENT OR CRACKED AND SORE NIPPLES स्तन के तकलीफों जैसे के क्रैक पड़ना, सूझ जाना , उनका इलाज<br>88=OTHERS (SPECIFY)
F80A11	Were you ever weighed at the AWC or during VHND or at a facility?<br>क्या कभी आपका वजन नापा गया था?(अन्नांवादी केंद्र में, VHND में या किसी स्वस्थ्य सुविधा में)	1 = YES हाँ<br>0= NO नहीं
F80A12	If yes, then how long before was the weight taken the last time?<br>यदि हाँ तोह पिछली बार कब वजन लिया गया था?	☐☐ MONTHS AGO
F79	Has [CHILD NAME] been registered with civil registry system?<br>क्या (बच्चे का नाम) सिविल रजिस्ट्री प्रणाली के अंतर्गत पंजीकृत है?	1 = YES हाँ<br>0 = NO नहीं

G. IMMUNISATION (RC2/3)

Read out to the respondent: Now I'm going to ask you about vaccinations that [CHILD NAME] may have received.
mÙkjnkrk dk i<dj lquk;sa vkc e vki ls mu Vhdk d ckjs e iNxh tk ¼cPps dk uke½ dk yxs gksA

G1.	Did the ASHA, AWW or ANM ever advice you on comprehensive immunÁations for [CHILD NAME]?<br>क्या आशा, आँगनवाड़ी कार्यकर्ता या ए.एन.एम. ने आपको (बच्चे का नाम) के पूर्ण टीकाकरण के बारे मे सलाह दी?	1 = YES हाँ<br>0 = NO नहीं
G2.	Did [CHILD NAME] ever receive any vaccinations to prevent (him/her) from getting diseases?<br>क्या (बच्चे का नाम) को कभी बिमारियों से बचाव के लिये कोई टीका लगा है?<br><br>**INCLUDE VACCINATIONS RECEIVED IN A PULSE POLIO PROGRAM**<br>iYl i®yh;® dk;Øe e fn, x, Vhdk dk **भी भामिल करें।**	1 = YES हाँ → **GO TO G4**<br>0 = NO नहीं
G3.	What is the <u>main reason</u> that [CHILD NAME] has not received any vaccinations?<br>(बच्चे का नाम) को कोई भी टीका न मिलने का <u>मुख्य कारण</u> क्या है?<br><br>**CODE ONLY ONE**<br>doy ,d mÙkj<br><br>**AFTER ANSWERING THIS, GO TO G9**<br>bl ç'u dk mÙkj nus d ckn **G9** ij tk,A	1 = TOO EXPENSIVE बहुत महंगा<br>2 = NO TIME TO TAKE CHILD TO FACILITY बच्चे को वहाँ तक पहूँचाने का समय नहीं<br>3 = NO TRANSPORTATION यातायात नहीं है<br>4 = NO IMMUNISATION SESSION WAS HELD<br>कोई टीकाकरण सत्र आयोजित नहीं किया गया<br>5 = IMMUNISATION SESSION WAS HELD AT AN INCONVENIENT TIME टीकाकरण सत्र असुविधाजनक समय पर आयोजित किया गया<br>6 = IMMUNISATION SITE IS TOO FAR<br>टीकाकरण की जगह बहुत दूर है<br>7= IMMUNISATIONS HAVE SIDE EFFECTS टीकाकरण के विपरीत प्रभाव हैं<br>8 = CHILD WAS NOT PRESENT IN THE HOUSEHOLD बच्चा घर पर ही नहीं था<br>9= IMMUNISATION IS DANGEROUS<br>टीकाकरण खतरनाक है<br>10 = IMMUNISATION IS UNNECESSARY<br>टीकाकरण अनावश्यक है<br>99 = DO NOT KNOW पता नहीं<br>88 = OTHER (SPECIFY) अन्य (स्पष्ट करें)
G4.	Do you have an immunisation card or MCP card where [CHILD NAME]'s vaccinations are written down?<br>क्या आपके पास (बच्चे का नाम) का कोई एसा कार्ड है जिस पर (बच्चे का नाम) के सभी टीके लिखे हैं?<br>**IF YES, ASK:** May I see the card?<br>;fn gk] iNs % क्या मैं कार्ड देख सकती हूँ	1 = YES, SAW CARD हाँ, मैने कार्ड देखा<br>2 = YES, DID NOT SEE CARD हाँ, मैंने कार्ड नहीं देखा → **GO TO G6**<br>0 = NO नहीं → **GO TO G6**

G5.	**COPY VACCINATION DATE FOR EACH VACCINE FROM THE CARD. CODE '77' IF CARD IS BLANK AND THEN SKIP TO G6. WRITE '44' IN 'DAY' COLUMN IF CARD SHOWS THAT A VACCINATION WAS GIVEN, BUT NO DATE IS RECORDED. CODE 0 IF DATE IS NOT MENTIONED & THE CARD DOES NOT SHOW THAT A VACCINE WAS GIVEN OR NOT. IF ONLY PART OF DATE IS SHOWN ON CARD, RECORD '99' OR '9999' FOR 'DO NOT KNOW' IN THE COLUMN FOR WHICH INFORMATION IS NOT GIVEN.**<br><br>टीकाकरण की तारीख को कार्ड से लिखें। यदि कार्ड खाली हैं तो 77 कोड करके जी6 पर जाए। यदि तारीख नहीं लिखी है लेकिन टीका दिया गया है तब दिन के कालम में 44 लिखें। यदि किसी टीकाकरण की तारीख नहीं लिखी है और यह भी नहीं मालूम है कि वह टीका लगा था या नहीं तो 0 कोड करें। यदि कार्ड में केवल तारीख का एक हिस्सा लिखा है तो जो जानकारी उपलब्ध नही हैं तो उस कॉलम में 99 या 9999 लिखें।	DAY<br>थ्दन	MONTH<br>महिना	YEAR<br>साल
a.	BCG बीसीजी			
b.	POLIO 0 (POLIO GIVEN AT BIRTH) पोलीयो 0 (पोलीयो जो जन्म के समय दिया गया)			
c.	HEPATITIS B 0 (HEPATITIS B GIVEN AT BIRTH) हेपाटाईटीस बी 0 (हेपाटाईटीस बी जो जन्म के समय दिया गया)			
d.	POLIO 1 पोलीयो 1			
e.	DPT 1 डीपीटी 1			
f.	PENTAVALENT 1 पेन्टावलेन्ट 1			
g.	ROTAVIRUS 1 रोटावाईरस 1			
G 1	HIB 1 हीब 1			
h.	HEPATITIS B 1 हेपाटाईटीस बी 1			
i.	POLIO 2 पोलीयो 2			
j.	DPT 2 डीपीटी 2			
k.	PENTAVALENT 2 पेन्टावलेन्ट 2			
l.	ROTAVIRUS 2 रोटावाईरस 2			
L 1	HIB 2 हीब 2			
m.	HEPATITIS B 2 हेपाटाईटीस बी 2			
n.	POLIO3 पोलीयो 3			
o.	DPT 3 डीपीटी 3			
p.	PENTAVALENT 3 पेन्टावलेन्ट 3			
q.	ROTAVIRUS 3 रोटावाईरस 3			
Q 1	HIB 3 हीब 3			
r.	HEPATITIS B 3 हेपाटाईटीस बी 3			
s.	IPV आईपीवी			
t.	MEASLES 1 खसरा 1			
u.	VITAMIN A (FIRST DOSE) विटामिन ए (पहली खुराक)			
v.	JE 1 जेई 1			
w.	DPT (16-24 MONTHS) डीपीटी (16–24 महीने)			
x.	POLIO (16-24 MONTHS) पोलियो (16–24 महीने)			
y.	VITAMIN A (SECOND DOSE) विटामिन ए (दूसरी खुराक)			
z.	JE 2 जेई 2			
aa	MEASLES 2 खसरा 2			
bb	VITAMIN A (THIRD DOSE) विटामिन ए (तीसरी खुराक)			
G6.	Please tell me if [CHILD NAME] received any of the following vaccinations:<br>कृप्या मुझे बताइये कि क्या (बच्चे का नाम) ने इनमे से कोई टीका लिया है	YES हाँ	NO नहीं	
a.	A BCG vaccination against tuberculosis that is, an injection in the arm or shoulder that usually causes a scar?<br>बीसीजी का टीका जो टीबी से लड़ने के लिये दिया जाता है और हाथ या कंधे पर लगाया जाता और उसके लगने से निशान रह जाता है?	1	0	
b.	A Polio vaccine that is drop given in the mouth?<br>पोलीयो टीका यानी मुँह में दिया जाने वाला बुंद?	1	0 → **GO TO G6f**	
c.	How many times? कितनी बार?	☐ NUMBER OF TIMES बार की संख्या		

d.	Was the first polio vaccine received in the first two weeks after birth?<br>क्या जन्म के दो सप्ताह के अन्दर पोलियो का बुंदें मिली थी?	1	0
e.	Drops in the mouth as part of the Pulse Polio campaign?<br>पल्स पोलीयो कार्यक्रम मे मुँह में पोलियो की बुँदें दी गयी थी?	1	0
f.	A DPT vaccination, that is, an injection given in the thigh, sometimes at the same time as polio drops?<br>डीपीटी का टीका जो की जाँघ पर दिया जाता है और कई बार यह पोलियो की दवाई के साथ भी दिया जाता है?	1	0 → **GO TO G6h**
g.	How many times? कितनी बार?	☐ NUMBER OF TIMES बार की संख्या	
h.	A Hepatitis B vaccination, that is, an injection given sometimes at the same time as DPT?<br>हेपाटाईटीस बी का टीका जो अक्सर डीपीटी के टीके के साथ दिया जाता है?	1	0 → **GO TO G6j**
i.	How many times? कितनी बार?	☐ NUMBER OF TIMES बार की संख्या	
j.	A drop against diarrhoea, that is, given sometimes at the same time as DPT?<br>दस्त से बचाव का टीका जो अक्सर डीपीटी के टीके के साथ दिया जाता है?	1	0 → **GO TO G6l**
k.	How many times? कितनी बार?	☐ NUMBER OF TIMES बार की संख्या	
l.	An injection against measles at right arm/shoulder?<br>खसरे से बचाव का टीका दाँए हाथ या कंधे पर?	1	0
m.	Vitamin A that is given by a spoon?<br>विटामिन ए की खुराक जो चम्मच से पिलायी जाती है?	1	0 → **GO TO G7**
n.	How many times? कितनी बार?	☐ NUMBER OF TIMES बार की संख्या	
G7.	Before [CHILD NAME] received vaccination the last time, did an ASHA/AWW/ANM visit your home to remind you that [CHILD NAME] was due for a vaccination?<br>जब (बच्चे का नाम) को आखरी बार टीका लगा था तब क्या किसी आशा/आँगनवाड़ी कार्यकर्ता/ए.एन.एम. ने आपको घर आकर याद दिलाया कि (बच्चे का नाम) को टीका लगना है?	1 = YES हाँ<br>0 = NO नहीं	
G8.	Has [CHILD NAME] received a vitamin A dose in the last 6 months?<br>क्या (बच्चे का नाम) को पिछले 6 महीने में विटामिन ए मिली थी?<br>**SHOW SYRUP**<br>flji fn[kk,A	1 = YES हाँ<br>0 = NO नहीं	
G9.	Has [CHILD NAME] received iron pills or iron syrup (like this/ any of these) in the last 6 months?<br>क्या (बच्चे का नाम) को पिछले 6 महीने में आयरन कि ऐसी गोलियाँ या सिरप मिली थी?<br>**SHOW SYRUP/TABLETS**<br>flji@xksfy;k fn[kk,A	1 = YES हाँ<br>0 = NO नहीं → **GO TO G11**	
**G9A**	Has [CHILD NAME] received any iron pills or iron syrup in the last 7 days?<br>क्या (बच्चे का नाम) ने पिछले 7 दिनों में आयरन की गोलियाँ या सिरप मिली हैं?	1 = YES हाँ<br>0 = NO नहीं	
G10.	Has [CHILD NAME] consumed any iron pills or iron syrup in the last 6 months?<br>क्या (बच्चे का नाम) ने पिछले 6 महीने में आयरन की गोलियाँ या सिरप ली हैं?	1 = YES हाँ<br>0 = NO नहीं	
G11.	Has [CHILD NAME] taken any drug to get rid of intestinal worms in the past 6 months?<br>क्या (बच्चे का नाम) ने पिछले 6 महिनों में पेट के कीड़े मारने के लिये कोई गोली ली है?	1 = YES हाँ<br>0 = NO नहीं	
**G11 A.**	**SKIP IF G2=0**<br>Apart from these vaccines, has (CHILD NAME) ever received any other vaccine?<br>इन टीकों के अलावा , कभी बच्चे को कोई और टीका लगा ?	1 = YES हाँ<br>0 = NO नहीं → **GO TO G12**	
G11 B.	Which other vaccines has (CHILD NAME) received?<br>बच्चे को कौन से टीके लगे थे ?<br>**PROBE : ANY OTHERS?**<br>çkcs dN vkj\<br><br>**RECORD ALL MENTIONED**<br>lHkh mÙkj fy[kA	1= VACCINE D वैक्सीन डी<br>2= MALARIA मलेरिया<br>3= PNEUMOCOCCAL न्यूमोकोक्कल<br>88= OTHER (SPECIFY) अन्य (स्पष्ट करें)	
G12.	**(RC 1/4/6/7/8)**Are you aware about VHND?<br>क्या आप वी.एच.एन.डी. (ग्राम स्वास्थ्य एवं पोष्ण दिवस) के बारे में जानती हैं?<br>**EXPLAIN ABOUT VHND**<br>xke LokLF; ,o ik".k fnol d ckj e crk,A	1 = YES हाँ<br>0 = NO नहीं → **GO TO H1**<br>99 = DO NOT KNOW पता नहीं → **GO TO H1**	

G13.	**(RC 1/4/6/7/8)**What services are available at the VHND?<br>वी.एच.एन.डी. (ग्राम स्वास्थ्य एवं पोष्ण दिवस) मे कौन सी सेवायें उपलब्ध होती हैं?<br><br>**PROBE : ANY OTHERS?**<br>çkc ¶ dN vkj\<br><br>**RECORD ALL MENTIONED**<br>lHkh mÙkj fy[kA	1 = IMMUNISATION FOR PREGNANT WOMEN गर्भवती महिलाओं के लिये टीकाकरण<br>2 = WEIGHT MEASUREMENT FOR PREGNANT WOMEN गर्भवती महिलाओं के वजन की जाँच<br>3 = BP MEASUREMENT FOR PREGNANT WOMEN<br>3 ¾ गर्भवती महिलाओं के रक्त चाप की जाँच<br>4 = ABDOMINAL EXAMINATION FOR PREGNANT WOMEN गर्भवती महिलाओं के पेट की जाँच<br>5 = BLOOD TESTS गर्भवती महिलाओं के खून की जाँच<br>6 = IFA TABLET DISTRIBUTION आयरन की गोलियों का वितरण<br>7 = IMMUNISATION FOR CHILDREN बच्चों का टीकाकरण<br>8 = GROWTH MONITORING FOR CHILDREN बच्चों का वजन<br>9 = THR DISTRIUTION टी.एच.आर. (पंजीरी) का वितरण<br>10 = GROUP MEETING BY ANM ए.एन.एम. द्वारा आयोजित समूह बैठक<br>88 = OTHER (SPECIFY) अन्य (स्पष्ट करें)
G14.	**(RC 1/4/6/7/8)**Was VHND organized in your village in the last month?<br>क्या पिछले महिने आपके गाँव मे वी.एच.एन.डी. (ग्राम स्वास्थ्य एवं पोष्ण दिवस) अयोजित हुई थी?	1 = YES हाँ<br>0 = NO नहीं<br>99 = DO NOT KNOW मालूम नहीं
G15.	**(RC 1/4/6/7/8)**Have you attended any VHND since you got pregnant with [NAME OF CHILD] or since you had [NAME OF CHILD]?<br>जब (बच्चे का नाम) आपके गर्भ में था या (बच्चे का नाम) के बाद क्या आपने कभी वी.एच.एन.डी. (ग्राम स्वास्थ्य एवं पोष्ण दिवस) मे भाग लिया है?	1 = YES हाँ<br>0 = NO नही
**G15 A**	**(RC 1/4/6/7/8)**Have you attended any VHND in last one month?<br>क्या पिछले 1 महिने में आपने वी.एच.एन.डी. (ग्राम स्वास्थ्य एवं पोष्ण दिवस) मे भाग लिया है?	1 = YES हाँ<br>= NO नहीं
G16.	**(RC 1/4/6/7/8)**In the last VHND that you attended, did an ASHA, AWW, or ANM talk to you about your or your children's health?<br>जब आपने आखिरी बार वी.एच.एन.डी. (ग्राम स्वास्थ्य एवं पोष्ण दिवस) मे भाग लिया था तो क्या आशा, आँगनवाड़ी कार्यकर्ताया या ए.एन.एम. ने आपसे आपके या आपके बच्चों के स्वास्थ के बारे मे चर्चा की थी ?	1 = YES हाँ<br>0 = NO नहीं
G17.	**(RC 1/4/6/7/8)**What information related to pregnancy, delivery and child care was discussed at the VHNDs?<br>वी.एच.एन.डी. (ग्राम स्वास्थ्य एवं पोष्ण दिवस) मे गर्भवास्था, प्रसव एवं बच्चें की देख रेख के बारे मे किन जानकारीयों पर चर्चा हुई थी?<br><br>**PROBE : ANY OTHERS?**<br>çkc ¶ dN vkj\<br><br>**RECORD ALL MENTIONED**<br>lHkh mÙkj fy[kA	1 = ANTENATAL CARE/PREPARING FOR DELIVERY प्रसव पूर्व देखभाल या प्रसव की तैयरी<br>2 = HOW TO TAKE CARE OF THE NEWBORN नवजात शिशु की देख रेख<br>3 = FEEDING THE BABY बच्चे का खान पान<br>4 = IMMUNIZATION टीकाकरण<br>5 = FAMILY PLANNING/USE OF CONTRACEPTIVES परिवार नियोजन या परिवार नियोजन के तरीकों का इस्तेमाल<br>6 = HYGIENE AND SANITATION स्वछता और साफ सफाई<br>7 = TALKING TO YOUR HUSBAND ABOUT FAMILY PLANNING/CONTRACEPTION परिवार नियोजन या परिवार नियोजन के तरीकों के बारे मे अपने पति से बातचीत<br>8 = TALKING TO HOUSEHOLD MEMBERS ABOUT YOUR HEALTH आपके स्वास्थ्य के बारे मे आपके परिवार के सदस्यों के साथ बातचीत<br>9 = TALKING TO HOUSEHOLD MEMBERS ABOUT YOUR CHILD'S

		HEALTH आपके बच्चे के स्वास्थ्य के बारे मे आपके परिवार के सदस्यों के साथ बातचीत<br>11=VARIETIES AND AMOUNT OF FOOD TO BE CONSUMED<br>किस प्रकार का खाना और कितने मात्र में खाना चाहिए<br>12=WEIGHT TO BE GAINED DURING AND AFTER PREGNANCY<br>वजन कितना बढ़ना चाहिए गर्भवस्था के दौरान और बच्चे के जन्म के बाद<br>13=HOW MANY IFA TABLETS TO CONSUME AND WHY<br>कितनी ifa की गोलियां और क्यूँ खानी चाहिए<br>14=HOW MANY CALCIUM TABLETS TO CONSUME AND WHY<br>कितनी कैल्शियम की गोलियां और क्यूँ खानी चाहिए<br>88 = OTHER (SPECIFY) अन्य (स्पष्ट करें)<br>F41 DO NOT KNOW पता नहीं

H. REPRODUCTIVE HEALTH (RC1/2/3/4/6/7)			
Read out to the respondent: The next questions are about family planning, and the various ways or methods that a couple can use to delay or avoid a pregnancy.<br>mÙkjnkrk dk i<dj lquk;s vxys dN ç'u ifjokj fu;kstu ds ckjs es vkSj mu rjhdksa ds ckjs es gS ftlls ,d nEifr xHkZ/kkj.k dks jksdus ;k nsj djus ds fy;s iz;ksx dj ldrs gSA			
H1.	Now I would like to talk about family planning - the various ways or methods that a couple can use to delay or avoid a pregnancy. Which ways or methods have you heard about?<br>अब मैं आपसे उन अलग अलग प्रकार के परिवार नियोजन के उपायों और तरीकों के बारे में पूछूँगी जो एक दम्पति गर्भधारण को रोकने या देर करने के लिये प्रयोग कर सकते है। आपने किन उपायों और तरीकों के बारे में सुना है?<br><br>**FOR METHODS NOT MENTIONED SPONTANEOUSLY, ASK: Have you ever heard of (METHOD)?**<br>ftu rjhdksa ds ckjs es Lo;a ugha crk;] iNs % D;k vkius dHkh ¼rjhdk½ ds ckjs es lquk gS\<br><br>**CODE '1' FOR EACH METHOD MENTIONED SPONTANEOUSLY. THEN PROCEED READING THE NAME AND DESCRIPTION OF EACH METHOD NOT MENTIONED SPONTANEOUSLY. CIRCLE CODE '2' IF METHOD IS RECOGNIZED AND CODE '0' IF NOT RECOGNIZED.**<br>Lo;a crk;s gj rjhds dk ß1Þ dkM djsA mlds ckn ftu rjhdks dk ugha crk;k mlds uke vkSj fooj.k dks i<sA ;fn rjhdk irk gks ß2Þ dkM djs vkSj rjhdk ugha irk gks ß0Þ dkM djsA		H2.<br>**ASK ONLY IF H1= 1 OR 2**<br>;fn ,N1¾1 ;k 2] rHkh iNsA<br>Have you ever used [METHOD]? क्या आपने कभी तरीके का प्रयोग किया है?
a.	**FEMALE STERILIZATION :** Women can have an operation to avoid having any more children.<br>efgyk ulcUnh % जिसमें एक महिला और बच्चे नहीं हो उसका रोकने के लिये आपरेशन करा सकती है।	1 = YES, SPONTANEOUS हाँ, स्वयं बताया<br>2 = YES, AFTER READING हाँ, पढ़ने के बाद<br>0 = NO नहीं	Have you ever had an operation to avoid having any more children?<br>और बच्चे को होने से रोकने के लिये क्या आपने कभी आपरेशन कराया है?<br>1 = YES हाँ<br>0 = NO नहीं
b.	MALE STERILIZATION : Men can have an operation to avoid having any more children.<br>पुरूाा ulcUnh % एक पुरूश और बच्चे नहीं हो उसको रोकने के लिये आपरेशन करा सकता है।	1 = YES, SPONTANEOUS हाँ, स्वयं बताया<br>2 = YES, AFTER READING हाँ, पढ़ने के बाद<br>0 = NO नहीं	Has your husband/partner ever had an operation to avoid having any more children?<br>और बच्चे को होने से रोकने के लिये क्या आपके पति/साथी ने आपरेशन कराया है ?<br>1 = YES हाँ<br>0 = NO नहीं

c.	**ORAL CONTRACEPTIVE PILL :** Women can take a pill every day or every week to avoid becoming pregnant.<br>xHk fujk/kd xkfy;k ½ जो एक महिला गर्भवती होने से बचने के लिये प्रतिदिन या सप्ताह में एक बार खा सकती है।	1 = YES, SPONTANEOUS हाँ, स्वयं बताया<br>2 = YES, AFTER READING हाँ, पढ़ने के बाद<br>0 = NO नहीं	1 = YES हाँ<br>0 = NO नहीं
d.	**IUD OR LOOP or Cu-T :** Women can have a loop or coil placed inside them by a doctor or a nurse.<br>vkb- ;- Mh- ;k Ykwi ;k dkij Vh ½ एक महिला गर्भवती होने से बचने के लिय डाक्टर या नर्स द्वारा अपने गर्भाशय के अन्दर लगवा सकती है।	1 = YES, SPONTANEOUS हाँ, स्वयं बताया<br>2 = YES, AFTER READING हाँ, पढ़ने के बाद<br>0 = NO नहीं	1 = YES हाँ<br>0 = NO नहीं
e.	**INJECTABLES :** Women can have an injection by a health provider that stops them from becoming pregnant for one or more months.<br>bUtD'ku ½ एक महिला किसी स्वास्थ्य कार्यकर्ता से इन्जेक्शन लगवा सकती है जिससे वह एक महिने या ज्यादा महीनों तक गर्भवती होने से बच सकती है।	1 = YES, SPONTANEOUS हाँ, स्वयं बताया<br>2 = YES, AFTER READING हाँ, पढ़ने के बाद<br>0 = NO नहीं	1 = YES हाँ<br>0 = NO नहीं
f.	CONDOM OR NIRODH : Men can put a rubber sheath on their penis before sexual intercourse.<br>dUMke ;k fujk/k ½ एक पुरूश यौन सम्बन्ध करने से पहले महिला को गर्भवती होने से बचाने के लिय अपने लिंग पर एक रबर का आवरण चढ़ा सकता है।	1 = YES, SPONTANEOUS हाँ, स्वयं बताया<br>2 = YES, AFTER READING हाँ, पढ़ने के बाद<br>0 = NO नहीं	1 = YES हाँ<br>0 = NO नहीं
g.	**FEMALE CONDOM :** Women can place a sheath in their vagina before sexual intercourse.<br>efgyk dUMke ½ एक महिला यौन सम्बन्ध करने से पहले अपनी योनि में एक रबर का आवरण लगा सकती है।	1= YES, SPONTANEOUS हाँ, स्वयं बताया<br>2= YES, AFTER READING हाँ, पढ़ने के बाद<br>0= NO नहीं	1 = YES हाँ<br>0 = NO नहीं
h.	**RHYTHM METHOD :** Every month that a woman is sexually active she can avoid pregnancy by not having sexual intercourse on the days of the month she is most likely to get pregnant.<br>Ljf{kr dkyvof/k ½ प्रत्येक महीने एक महिला उन दिनों में यौन सम्बन्ध नहीं बना कर गर्भधारण से बच सकती है जब उसके गर्भधारण की संभावनाएं सबसे अधिक होती है।	1 = YES, SPONTANEOUS हाँ, स्वयं बताया<br>2 = YES, AFTER READING हाँ, पढ़ने के बाद<br>0 = NO नहीं	1 = YES हाँ<br>0 = NO नहीं
i.	**WITHDRAWAL :** Men can be careful and pull out before climax.<br>ckgj fudkyuk ½ पुरूश सावधानी कर चरमावस्था पर पहुँचने से पहले अपना लिंग बाहर निकाल लेता है।	1 = YES, SPONTANEOUS हाँ, स्वयं बताया<br>2 = YES, AFTER READING हाँ, पढ़ने के बाद<br>0 = NO नहीं	1 = YES हाँ<br>0 = NO नहीं
j.	**EMERGENCY CONTRACEPTION :** Women can take pills up to three days after sexual intercourse to avoid becoming pregnant.<br>vikrdkyhu xHkfujk/kd ½ एक महिला यौन सम्बन्ध बनाने के तीन दिनों के अन्दर गोली खाकर गर्भधारण से बच सकती है।	1 = YES, SPONTANEOUS हाँ, स्वयं बताया<br>2 = YES, AFTER READING हाँ, पढ़ने के बाद<br>0 = NO नहीं	1 = YES हाँ<br>0 = NO नहीं
J1	**IMPLANTS:** The implant is a form of long-acting reversible contraception for women.Contraceptive implants are small rods about the size of a matchstick which are put under the skin in the inside of your arm. Implants last either three or five years depending whether there are one or two rods.<br><br>xHkfujk/kd çR;kjki.k : **इम्प्लांट एक तरीके का लचीली छर या कैप्सूल होता है जिन्हें उपरी बांह की त्वचा के अन्दर लगा दिया जाता है**	1 = YES, SPONTANEOUS हाँ, स्वयं बताया<br>2 = YES, AFTER READING हाँ, पढ़ने के बाद<br>0 = NO नहीं	1 = YES हाँ<br>= NO नहीं

J2	**DIAPHRAGM**: A shallow silicone cup inserted into the vagina to prevent pregnancy. A diaphragm can be used for over two years.<br><br>xHkZfujks/kd Vksih:**दयाफ्रम को सम्भोग से पहले योनी में गहरे रख दिया जाता है. यह ग्रीवा को धक् लेता है**	1 = YES, SPONTANEOUS हाँ, स्वयं बताया<br>2 = YES, AFTER READING हाँ, पढ़ने के बाद<br>0 = NO नहीं	1 = YES हाँ<br>= NO नहीं
J3	**FOAM/JELLY:** Spermicides are barrier birth control methods that are bought over the counter. Once inserted, these contraceptives kill sperm. Vaginal spermicides are available in several forms: spermicidal jelly, cream, foam, tablets, suppositories, sponge, and film. Spermicide is inserted into the vagina just before sex to prevent pregnancy.<br><br>Qke@ tSyh: **यह जेली जैसी सम्भोग के पहले योनी पे लगायी जाती है**	1 = YES, SPONTANEOUS हाँ, स्वयं बताया<br>2 = YES, AFTER READING हाँ, पढ़ने के बाद<br>0 = NO नहीं	1 = YES हाँ<br>= NO नहीं
J4	**LAM:**<br><br>A mother is breastfeeding her baby on demand, both day and night and not feeding other foods or liquids regularly(Occasional tastes of foods or other liquids are permitted, but they should never replace a feeding at the breast.) AND her baby is less than 6 months old<br>**LAM:** 6 माह तक मा द्वारा स्तनपान जो परिवार नियोजन का एक साधन भी हैं	**1 = YES, SPONTANEOUS** gk¡] Lo;a crk;k<br>**2 = YES, AFTER READING** gk¡] i<+us ds ckn<br>**0 = NO** ugha	1 = YES हाँ<br>= NO नहीं
J5	**STANDARD DAYS METHOD:** To use the method, couples abstain from sexual intercourse aon days 8 through 19 of the woman's menstrual cycle.<br><br>मानक तिथि बिधि	**1 = YES, SPONTANEOUS** gk¡] Lo;a crk;k<br>**2 = YES, AFTER READING** gk¡] i<+us ds ckn<br>**0 = NO** ugha	1 = YES हाँ<br>= NO नहीं
k.	Have you heard of any other ways or methods that women or men can use to avoid pregnancy?<br>क्या आपने किसी अन्य उपाय या तरीके के बारे में सुना है जो एक महिला या पुरूश गर्भधारण होने से बचने के लिये प्रयोग कर सकते है ?	1= YES (SPECIFY) हाँ (स्पष्ट करें)<br>0 = NO नहीं	1 = YES हाँ<br>0 = NO नहीं
	**SKIP H3- H16 FOR RC 6**		
H3.	After the delivery of [CHILD NAME], did you or your husband receive any counseling/advice on family planning by a health provider?<br>(बच्चे का नाम) के जन्म के बाद, क्या आप/आपके पति को किसी स्वास्थ्य सेवा प्रदाता द्वारा परिवार नियोजन के तरीकों के बारे में बताया गया था/सलाह दी गयी थी?	1 = YES हाँ<br>0 = NO नहीं → **GO TO H11**	

			YES	NO
H4.	After delivery, did you receive any counselling / advice on family planning from ASHA/AWW/ANM?<br><br>प्रसव के बाद क्या आपको परिवार नियोजन की सलाह मिली थी?	ASHA	1	0
		ANM	1	0
		AWW	1	0

H6.	What family planning methods were you/your husband informed about?<br>आप या आपके पति को परिवार नियोजन के किन तरिकों के बारे में बताया गया था?<br><br>**PROBE : ANY OTHERS?**<br>çkc % dN vkj\<br><br>**RECORD ALL MENTIONED**<br>lHkh mÙkj fy[kA	1 = FEMALE STERILIZATION महिला नसबन्दी<br>2 = MALE STERILIZATION पुरूश नसबन्दी<br>3 = PILL गर्भ निरोधक गोलियाँ<br>4 = IUCD/COPPER-T आई.यू.सी.डी./लूप<br>5 = INJECTABLES इन्जेक्शन<br>6 = CONDOM/NIRODH कान्डोम या निरोध<br>7 = IMPLANTS गर्भनिरोधक प्रत्यारोपण<br>8 = FEMALE CONDOM महिला कान्डोम<br>9 = DIAPHRAGM गर्भनिरोधक टोपी<br>10 = FOAM/JELLY फोम/जैली<br>11 = RHYTHM METHOD सुरक्षित कालअवधि<br>12 = WITHDRAWAL बाहर निकालना<br>13 = LAM 6 माह तक मा द्वारा स्तनपान जो परिवार नियोजन का एक साधन भी हैं<br>14 = EMERGENCY CONTRACEPTION आपातकाल गर्भनिरोधक<br>88 = OTHER (SPECIFY) अन्य (स्पष्ट करें)
H10.	Would you say that the health service provider was able to address all your/your husband's queries?<br>क्या आपको लगता है कि स्वास्थ्य सेवा प्रदाता आप/आपके पति के प्रश्नों का समाधान करने में सक्षम थे?	1 = YES हाँ<br>0 = NO नहीं
H11	During your pregnancy with [CHILD NAME], did you or your husband receive any counseling/advice on family planning by a health provider?<br>जब (बच्चे का नाम) आपके गर्भ मे था, तब क्या आप/आपके पति को किसी स्वास्थ्य सेवा प्रदाता द्वारा परिवार नियोजन के तरिकों के बारे में बताया गया था/सलाह दी गयी थी?	1 = YES हाँ<br>0 = NO नहीं → **GO TO H17**
H11 A.	(RC 8)<br>ASK IF B14=1 ELSE SKIP TO H12<br>During this pregnancy, did you receive any counselling / advice on family planning from ASHA/AWW/ANM?<br><br>इस गर्भवस्था के दौरान क्या आपको परिवार नियोजन की सलाह मिली थी?	(blank) | YES | NO<br>ASHA | 1 | 0<br>ANM | 1 | 0<br>AWW | 1 | 0
H12.	What family planning methods were you/your husband informed/advised about?<br>आप/आपके पति को परिवार नियोजन के किन तरिकों के बारे में बताया गया था/सलाह दी गयी थी?<br><br>**PROBE : ANY OTHERS?**<br>çkc % dN vkj\<br><br>**RECORD ALL MENTIONED**<br>lHkh mÙkj fy[kA	1 = FEMALE STERILIZATION महिला नसबन्दी<br>2 = MALE STERILIZATION पुरूश नसबन्दी<br>3 = PILL गर्भ निरोधक गोलियाँ<br>4 = IUCD/COPPER-T आई.यू.सी.डी./लूप<br>5 = INJECTABLES इन्जेक्शन<br>6 = CONDOM/NIRODH कान्डोम या निरोध<br>7 = IMPLANTS गर्भनिरोधक प्रत्यारोपण<br>8 = FEMALE CONDOM महिला कान्डोम<br>9 = DIAPHRAGM गर्भनिरोधक टोपी<br>10 = FOAM/JELLY फोम/जैली<br>11 = RHYTHM METHOD सुरक्षित कालअवधि<br>12 = WITHDRAWAL बाहर निकालना<br>13 = LAM 6 माह तक मा द्वारा स्तनपान जो परिवार नियोजन का एक साधन भी हैं<br>14 = EMERGENCY CONTRACEPTION आपातकाल गर्भनिरोधक<br>88 = OTHER (SPECIFY) अन्य (स्पष्ट करें)
	Would you say that the health service provider was able to address all your/your husband's queries?<br>क्या आपको लगता है कि स्वास्थ्य सेवा प्रदाता आप/आपके पति के प्रश्नों का समाधान करने में सक्षम थे?	1 = YES हाँ<br>0 = NO नहीं
H17	Are you/your husband currently doing something or using any method to delay or avoid getting pregnant?<br>क्या आप/आपके पति वर्तमान मे गर्भधारण को रोकने या उसमें देरी करने के लिए किसी तरीके का प्रयोग कर रहे है?	1 = YES हाँ<br>0 = NO नहीं → **GO TO H31**

H18	Which method are you using?<br>आप किस तरीके का प्रयोग कर रहे है?<br><br>**CODE ONLY ONE**<br>doy ,d mÙkj	1 = FEMALE STERILIZATION महिला नसबन्दी<br>2 = MALE STERILIZATION पुरूश नसबन्दी<br>3 = PILL गर्भ निरोधक गोलियाँ<br>4 = IUCD/COPPER-T आई.यू.सी.डी./लूप<br>5 = INJECTABLES इन्जेक्शन<br>6 = CONDOM/NIRODH कान्डोम या निरोध<br>7 = IMPLANTS गर्भनिरोधक प्रत्यारोपण<br>8 = FEMALE CONDOM महिला कान्डोम<br>9 = DIAPHRAGM गर्भनिरोधक टोपी<br>10 = FOAM/JELLY फोम/जैली<br>11 = RHYTHM METHOD सुरक्षित कालअवधि<br>12 = WITHDRAWAL बाहर निकालना<br>13 = LAM 6 माह तक मा द्वारा स्तनपान जो परिवार नियोजन का एक साधन भी हैं<br>14 = EMERGENCY CONTRACEPTION आपातकाल गर्भनिरोधक<br>88 = OTHER (SPECIFY) अन्य (स्पष्ट करें)
H19	When after the birth of [CHILD NAME], did you accept this method of family planning?<br>(बच्चे का नाम) के जन्म के बाद आपने इस परिवार नियोजन के तरिका को कब अपनाया था?	☐ ☐☐<br>1 = HOURS घंटे<br>2 = DAYS दिन<br>3 = WEEKS सप्ताह<br>4 = MONTHS महीना<br>5 = YEARS साल<br>99 = DO NOT KNOW/REMEMBER पता नही/याद नहीं
H19 B	**ASK ONLY IF IUCD**<br>When did you remove IUCD, after its insertion?<br>लगाने के बाद हटाया कब गया?	☐☐ / ☐☐ / ☐☐<br>DD MM YY<br><br>नहीं हटाया है तोह 00 डालें
H20	Did you and your husband discuss which method to adopt prior to accepting the method?<br>परिवार नियोजन का कौन सा तरीका अपनाना है इस बारे मे क्या आप और आपके पति ने परिवार नियोजन अपनाने से पहले चर्चा की थी?	1 = YES हाँ<br>0 = NO नहीं
H21	Was this the family planning method of your choice or you were forced to accept this method?<br>क्या परिवार नियोजन के इस तरीका को आपनाना अपकी मरजी थी या आपको मजबूर किया गया था?	1 = MY CHOICE मेरी मरजी → **GO TO H24**<br>2 = FORCED मजबूर किया गया
H22	Would you have chosen a different family planning method?<br>क्या आप परिवार नियोजन का कोई दूसरा तरीका अपनाती?	1 = YES हाँ<br>0 = NO नहीं → **GO TO H24**
H23	Which family planning method would you have chosen?<br>आप परिवार नियोजन का कौनसा तरीका अपनाती?<br><br>**CODE ONLY ONE**<br>doy ,d mÙkj	1 = FEMALE STERILIZATION महिला नसबन्दी<br>2 = MALE STERILIZATION पुरूश नसबन्दी<br>3 = PILL गर्भ निरोधक गोलियाँ<br>4 = IUCD/COPPER-T आई.यू.सी.डी./लूप<br>5 = INJECTABLES इन्जेक्शन<br>6 = CONDOM/NIRODH कान्डोम या निरोध<br>7 = IMPLANTS गर्भनिरोधक प्रत्यारोपण<br>8 = FEMALE CONDOM महिला कान्डोम<br>9 = DIAPHRAGM गर्भनिरोधक टोपी<br>10 = FOAM/JELLY फोम/जैली<br>11 = RHYTHM METHOD सुरक्षित कालअवधि<br>12 = WITHDRAWAL बाहर निकालना<br>13 = LAM 6 माह तक मा द्वारा स्तनपान जो परिवार नियोजन का एक साधन भी हैं<br>14 = EMERGENCY CONTRACEPTION आपातकाल गर्भनिरोधक<br>88 = OTHER (SPECIFY) अन्य (स्पष्ट करें)

H24	**ASK ONLY IF CODED 1 OR 2 IN H18**<br>;fn ,N18 e 1 ;k 2 dkM fd;k g rc iN<br>Before your sterilization operation, were you or your husband told that you would not be able to have any (more) children because of the operation?<br>क्या नसबन्दी करने से पहले आप/आपके पति को बताया गया था कि आप और बच्चे नहीं पैदा कर पायेंगी?	1 = YES हाँ<br>0 = NO नहीं
H25	Were you told what to do if you experienced side effects or problems with the method?<br>क्या आपको बताया गया था कि यदि आपको परिवार नियोजन के इस तरिके को अपनाने से कोई असुविधा या परेशानी हुई तब आपको क्या करना चाहिये?	1 = YES हाँ<br>0 = NO नहीं
H26	**ASK ONLY IF CODED 1 - 10 IN H18, ELSE GO TO H29**<br>;fn ,N18 e 1 l 10 dkM fd;k g rc iN] vU;Fkk ,N29 ij tk;<br>Did you receive any follow-up care after accepting this method?<br>क्या परिवार नियोजन के इस तरिके को अपनाने के बाद दोबारा आपको सेवा देने कोई आया था?	1 = YES हाँ<br>0 = NO नहीं → **GO TO H29**
H26 A.	Did you receive any **follow-up care** within a month after you started using this method?<br>इस बिधि के इस्तेमाल के बाद क्या कोई इलाज/सलाह देने एक महीने के अन्दर आपसे मिलने आया?	1=YES<br>0=NO
H27	Who provided that care?<br>आपको दोबारा सेवा किसने दी थी?<br><br>**PROBE : ANY OTHERS?**<br>çkc % dN vkj\<br><br>**RECORD ALL MENTIONED**<br>lHkh mÙkj fy[kA	1 = GOVERNMENT DOCTOR सरकारी डाक्टर<br>2 = PRIVATE DOCTOR प्राइवेट डाटर<br>3 = STAFF NURSE स्टाफ नर्स<br>4 = LHV एल.एच.वी.<br>5 = MALE HEALTH WORKER पुरूष स्वास्थ्यकर्मी<br>6 = ANM ए.एन.एम.<br>7 = OTHER HEALTH PERSONNEL अन्य स्वास्थ्यकर्मी<br>8 = ASHA आशा<br>9 = AWW आँगनवाड़ी कार्यकर्ता<br>10 = SBA/TRAINED DAI एसबीए/प्रशिक्षित दाई<br>11 = DAI दाई<br>12 = RMP आर.एम.पी. (झोला छाप डाक्टर)<br>88 = OTHERS (SPECIFY) अन्य (स्पष्ट करें)
H28	How long after accepting this method was the follow-up done?<br>परिवार नियोजन के इस तरिके को अपनाने के कितने समय बाद आपको दोबारा सेवा देने कोई आया था?	☐ ☐☐<br>1 = HOURS घंटे<br>2 = DAYS दिन<br>3 = WEEKS सप्ताह<br>4 = MONTHS महीना<br>5 = YEARS साल<br>99 = DO NOT KNOW/REMEMBER पता नही/याद नहीं
H29	Did you experience any difficulties due to using this method?<br>क्या आपको परिवार नियोजन के इस तरिके को अपनाने के बाद किसी तरह की परेशानी हुई थी?	1 = YES हाँ<br>0 = NO नहीं → **GO TO H34**
H30	What were the difficulties that you experienced?<br>परिवार नियोजन के इस तरिके को अपनाने के बाद आपको किस तरह की परेशानी हुई थी?<br><br>**PROBE : ANY OTHERS?**<br>çkc % dN vkj\<br><br>**RECORD ALL MENTIONED**<br>lHkh mÙkj fy[kA	1 = METHOD FAILED/GOT PREGNANT विधि विफल हुई/गर्भवती हो गई<br>2 = LACK OF SEXUAL SATISFACTION यौन संतुष्टि का अभाव<br>3 = CREATED MENSTRUAL PROBLEM माहवारी समस्या हो गयी<br>4 = CREATED HEALTH PROBLEM स्वास्थ्य समस्या हो गयी<br>5 = HARD TO USE प्रयोग करने मे कठिनाई<br>6 = HARD TO GET मिलने मे कठिनाई<br>7 = PUT ON WEIGHT वजन बढ़ना<br>88 = OTHERS (SPECIFY) अन्य (स्पष्ट करें)
H30 A.	Did you receive any advice or treatment for this complication?<br>इस गतिलता के लिए क्या आपको कोई सलाह मिली थी या इलाज करवाया था?	1=YES<br>0=NO

H31	**ASK ONLY IF THE WOMAN WANTS TO SPACE/LIMIT CHILDREN AND IS NOT USING ANY METHOD CURRENTLY**<br>dsoy rHkh iNs ;fn efgyk cPps ugh pkgrh@cPpks es vUrj djuk pkgrh gS vkj orZeku es ifjokj fu;kstu dk dksbZ rfjdk ugh ç;ksx dj jgh gSA<br><br>You said that you do not want to have more children/want to have a space between children, why are you not using any family planning methods currently?<br>आपने बताया कि आप और बच्चे नहीं चाहती/बच्चों में अन्तर करना चाहती हैं, वर्तमान मे परिवार नियोजन का कोई तरिका क्यों नहीं प्रयोग कर रही हैं?<br><br>**PROBE : ANY OTHERS?**<br>çkc % dN vkj\<br><br>**RECORD ALL MENTIONED**<br>lHkh mÙkj fy[kA	1 = INFREQUENT SEX/NO SEX यौन सम्बन्ध कभी कभी/बिल्कुल नहीं<br>2 = HUSBAND AWAY पति बाहर रहते हैं<br>3 = MENOPAUSAL/HYSTERECTOMY रजोनिवृत<br>4 = SUBFECUND/INFECUND बाँझ<br>5 = FATALISTIC भाग्य पर छोड़ दिया<br>6 = WANTS CHILDREN/AS MANY CHILDREN AS POSSIBLE बच्चे /जितने हो सके उतने बच्चे चाहते हैं<br>7 = UP TO GOD भगवान की मरजी<br>8 = BREASTFEEDING/POSTPARTUM AMENORRHOEA बच्चा दूध पी रहा है/प्रसवोत्तर एमनोरिया<br>9 = RESPONDENT OPPOSED उत्तरदाता द्वारा विरोध<br>10 = HUSBAND OPPOSED पति द्वारा विरोध<br>11 = OTHERS OPPOSED लोगों द्वारा विरोध<br>12 = RELIGIOUS PROHIBITION धार्मिक प्रतिबन्ध<br>13 = KNOWS NO METHOD कोई तरीका नहीं जानते<br>14 = KNOWS NO SOURCE कोई स्रोत नहीं जानते<br>15 = HEALTH CONCERNS स्वास्थ्य की चिन्ता<br>16 = FEAR OF SIDE EFFECTS उल्टे नतिजे का डर<br>17 = LACK OF ACCESS/TOO FAR पहुँच से दूर/काफी दूर है<br>18 = COSTS TOO MUCH बहुत महंगा है<br>19 = DIFFICULT/INCONVENIENT TO GET METHOD विधि मिलने मे मुश्किल/असुविधाजनक<br>20 = INCONVENIENT TO USE प्रयोग में असुविधाजनक<br>21 = INTERFERES WITH BODY'S NORMAL PROCESSES शरीर की सामान्य प्रक्रियाओं में बाधा डालता है<br>22 = DO NOT LIKE EXISTING METHOD मौजूदा तरिके पसन्द नहीं<br>23 = AFRAID OF STERILISATION नसबन्दी से डर लगता है<br>88 = OTHER (SPECIFY) अन्य (स्पष्ट करें)<br>99 = DO NOT KNOW पता नहीं
H32	**ASK ONLY IF H17= 0** ;fn ,N18 es 0 gS iNs<br>Do you think you will use a contraceptive method to delay or avoid pregnancy sometime during the next 6 months?<br>क्या आप सोचते है कि अगले 6 महिनों में आप बच्चों में देरी या अन्तर करने के लिए परिवार नियोजन का कोई तरिका प्रयोग करेंगे?	1 = YES हाँ GOTO H32B<br>0 = NO नहीं<br>99 = DO NOT KNOW पता नहीं
H32 A.	Do you think you will use a contraceptive method to delay or avoid pregnancy in the next 12 months?<br>क्या आपको लगता है के आप आने वाले 12 महीने में कोई परिवार नियोजन की पद्धति का इस्तेमाल करेंगे?	1 = YES हाँ<br>0 = NO नहीं<br>99 = DO NOT KNOW पता नहीं
H32 B.	Which contraceptive method would you prefer to use?<br>कौनसा?	1 = FEMALE STERILIZATION महिला नसबन्दी<br>2 = MALE STERILIZATION पुरूश नसबन्दी<br>3 = PILL गर्भ निरोधक गोलियाँ<br>4 = IUCD/COPPER-T आई.यू.सी.डी./लूप<br>5 = INJECTABLES इन्जेक्शन<br>6 = CONDOM/NIRODH कान्डोम या निरोध<br>7 = IMPLANTS गर्भनिरोधक प्रत्यारोपण<br>8 = FEMALE CONDOM महिला कान्डोम<br>9 = DIAPHRAGM गर्भनिरोधक टोपी<br>10 = FOAM/JELLY फोम/जैली<br>11 = RHYTHM METHOD सुरक्षित कालअवधि<br>12 = WITHDRAWAL बाहर निकालना<br>13 = LAM 6 माह तक मा द्वारा स्तनपान जो परिवार नियोजन का एक साधन भी हैं<br>14 = EMERGENCY CONTRACEPTION आपातकाल गर्भनिरोधक<br>88 = OTHER (SPECIFY) अन्य (स्पष्ट करें)

H33	**ASK ONLY IF NO IN H32 AND H32A**<br>What is the <u>main reason</u> that you think you will not use a contraceptive method at any time to delay or avoid in the future?<br>आपका भविश्य मे बच्चों में देरी या अन्तर करने के लिए परिवार नियोजन का कोई तरिका प्रयोग नहीं करने का <u>मुख्यत कारण</u> क्या है?<br><br>**CODE ONLY ONE**<br>dsoy ,d mÙkj	1 = INFREQUENT SEX/NO SEX यौन सम्बन्ध कभी कभी/बिल्कुल नहीं<br>2 = HUSBAND AWAY पति बाहर रहते हैं<br>3 = MENOPAUSAL/HYSTERECTOMY रजोनिवृत<br>4 = SUBFECUND/INFECUND बाँझ<br>5 = FATALISTIC भाग्य पर छोड़ दिया<br>6 = WANTS AS MANY CHILDREN AS POSSIBLE जितने हो सके उतने बच्चे चाहते हैं<br>7 = UP TO GOD भगवान की मरजी<br>8 = BREASTFEEDING/POSTPARTUM AMENORRHOEA बच्चा दूध पी रहा है /प्रसवोत्तर एमनोरिया<br>9 = RESPONDENT OPPOSED उत्तरदाता द्वारा विरोध<br>10 = HUSBAND OPPOSED पति द्वारा विरोध<br>11 = OTHERS OPPOSED लोगों द्वारा विरोध<br>12 = RELIGIOUS PROHIBITION धार्मिक प्रतिबन्ध<br>13 = KNOWS NO METHOD कोई तरीका नहीं जानते<br>14 = KNOWS NO SOURCE कोई स्रोत नहीं जानते<br>15 = HEALTH CONCERNS स्वास्थ्य की चिन्ता<br>16 = FEAR OF SIDE EFFECTS उल्टे नतिजे का डर<br>17 = LACK OF ACCESS/TOO FAR पहुँच से दूर/काफी दूर है<br>18 = COSTS TOO MUCH बहुत महंगा है<br>19 = DIFFICULT/INCONVENIENT TO GET METHOD विधि मिलने मे मुश्किल/असुविधाजनक<br>20 = INCONVENIENT TO USE प्रयोग में असुविधाजनक<br>21 = INTERFERES WITH BODY'S NORMAL PROCESSES शरीर की सामान्य प्रक्रियाओं में बाधा डालता है<br>22 = DO NOT LIKE EXISTING METHOD मौजूदा तरिके पसन्द नहीं<br>23 = AFRAID OF STERILISATION नसबन्दी से डर लगता है<br>88 = OTHER (SPECIFY) अन्य (स्पष्ट करें)<br>99 = DO NOT KNOW पता नहीं
H34	(RC 8)Would you say that using contraception is mainly your decision, mainly your husband's decision, you both decide together or someone else's?<br>परिवार नियोजन को प्रयोग करना मुख्यत किसका निर्णय है, आपका, आपके पति का, आप दोनों का या किसी और का?	1 = MAINLY RESPONDENT मुख्यत उत्तरदाता<br>2 = MAINLY HUSBAND मुख्यत पति<br>3 = JOINT DECISION दोनों का निर्णय<br>4 = MOTHER-IN-LAW'S DECISION सास का निर्णय<br>5 = APART FROM MIL OTHER'S DECISION सास के अलसवा दूसरों का निर्णय<br>88 = OTHER (SPECIFY) अन्य (स्पष्ट करें)

H34A.	**ASK IF CODED 10 OR 11 IN H31 OR H33, ELSE GO TO H35**<br><br>Which of the following things has your husband or mother-in-law ever done to oppose your using family planning?<br><br>निम्नलिखित में से क्या क्या आपके पति या सास ने कभी भी किया है, आपके परिवार नियोजन के फैसले के खिलाफ?	Yes	No
a.	Stopped you from going or refused to give you permission to go to a clinic or community 'health day' (VHND) to get family planning<br><br>आपको कोई भी स्वस्स्थ्य केंद्र या VHND में जाके परिवार नियोजन की सुविधा लेने से रोका	1	0
b.	Taken family planning (such as pills) away from you<br><br>परिवार नियोजन की बिधि जैसे पिल्स , कंडोम्स वगेरा आपसे ले लिया हो	1	0

c.	Told you that you would be abandoned if you tried to prevent or delay getting pregnant<br><br>आपको बताया के बच्चे होने को टालने की कोशिश की तोह आपको छोर देंगे	1	0
d.	Told you that you would be beaten if you tried to prevent or delay getting pregnant<br><br>आपको बताया के बच्चे होने को टालने की कोशिश की तोह आपको मारेंगेपीटेंगे/	1	0
e.	Told you that it was against your religion or culture to use family planning<br><br>आपको बताया के बच्चे पैदा करने को टालना धर्म और संस्कृति के खिलाफ है	1	0
f.	Told you that women who use FP do this so that they can have sex with other men<br><br>आपको बताया के महिला परिवार नियोजन के बिधि का प्रयोग इसीलिए करती है ताकि वोह दूसरे पुरुष के साथ सम्भोग कर सके	1	0
g.	Told you that you could not use FP because you did not have any or enough sons<br><br>आपको बताया के आप परिवार नियोजन का प्रयोग नहीं कर सकते क्यूंकि आपके पास जितनी चाहिए उतने बेटे नहीं है	1	0
H34B.	**ASK IF CODED 1 IN H34A a-g**<br><br>Who did or said this?<br><br>ऐसा किसने कहाँ?	1= HUSBAND<br><br>2=MOTHER-IN-LAW<br><br>3=BOTH	
H34C.	If you could use family planning without your husband or mother-in-law knowing, would you use family planning?<br><br>अगर आप अपने पति और सास को बिना बताये कोई परिवार नियोजन के बिधि का प्रयोग कर सके तोह वोह क्या होगा?	1=YES<br><br>0=NO<br><br>98= REFUSED TO ANSWER<br><br>99= DON'T KNOW	

H35	(RC 8)In your opinion, what is the ideal gap between two births?<br>आपके अनुसार दो बच्चों में कितने साल का अन्तर होना चाहिये?	☐☐. ☐☐YEARS साल<br>66 = AS CLOSE AS POSSIBLE जितना पास संबभ<br>99 = DO NOT KNOW पता नहीं
H36	(RC 8)Ideally, how many children would you like to have in total?<br>आदर्श रूप में, आप कुल कितने बच्चे चाहती हैं?	☐☐ NO. OF CHILDREN बच्चों की संख्या<br>99 = DO NOT KNOW पता नहीं

V. PREGNANCY (RC8)

This section will be asked to women of 15-49 years of age who are currently pregnant

Read out to the respondent: Now, I would like to ask you some questions about when you were pregnant with [CHILD NAME].

mÙkjnkrk dk i<dj lquk;s vc eS vkils ml le; ds ckjs eS dN ç'u iNwxh tc ¼cPps dk uke½ vkids iV es FkkA

V1.	How many months pregnant are you?<br>आप कितने महीने के गर्भ से हैं ?	☐ COMPLETED MONTHS पूर्ण महीने<br>99 = DO NOT KNOW पता नहीं
V2.	Have you registered this pregnancy?<br>क्या आपने गर्भावस्था का पंजीकरण कराया था?	1 = YES हाँ

		0 = NO नहीं → **GO TO V6**
V3.	Whom did you register your pregnancy with for the first time?<br>आपने गर्भावस्था का पंजीकरण पहली बार किसके पास कराया था?	1 = AWW आँगनवाड़ी कार्यकर्ता<br>2 = ASHA आशा<br>3 = ANM ए.एन.एम.<br>88 = OTHERS (SPECIFY) अन्य (स्पष्ट करें)
V4.	Did you get any card (Mother and Child Protection Card) after registering the pregnancy?<br>क्या गर्भावस्था का पंजीकरण कराने के बाद आपको कोई कोई कार्ड (माँ–बच्चा संरक्षण कार्ड) मिला था?	1 = YES हाँ<br>0 = NO नहीं
V5.	In which month of pregnancy was your pregnancy registered?<br>गर्भावस्था के किस महीने में आपने गर्भावस्था का पंजीकरण कराया था?	☐ COMPLETED MONTHS पूर्ण महीने<br>99 = DO NOT KNOW पता नहीं
V6.	During your pregnancy, did you go to see anyone for antenatal checkup?<br>क्या प्रसव–पूर्व जाँच के लिए आप किसी के पास गयी है?	1 = YES हाँ<br>0 = NO नहीं
V7.	During your pregnancy, did anyone come to you for antenatal checkup?<br>क्या प्रसव–पूर्व जाँच के लिए कोई आपके पास आया था?	1 = YES हाँ<br>0 = NO नहीं
V8.	**IF V6= 0 & V7= 0 SKIP TO V13**<br><br>Who all did your antenatal checkups during your pregnancy?<br>आपकी प्रसव–पूर्व जाँच किस–किस ने की थी?<br><br>**PROBE : ANY OTHERS?**<br>çkc % dN vkj\<br><br>**RECORD ALL MENTIONED**<br>lHkh mÙkj fy[kA	1 = GOVERNMENT DOCTOR सरकारी डाक्टर<br>2 = PRIVATE DOCTOR प्राइवेट डाक्टर<br>3 = STAFF NURSE स्टाफ नर्स<br>4 = LHV लेडी हैल्थ विजिटर (एल.एच.वी.)<br>5 = MALE HEALTH WORKER पुरुष स्वास्थ्य कर्मी<br>6 = ANM ए.एन.एम.<br>7 = OTHER HEALTH PERSONNEL अन्य स्वास्थ्य कर्मी<br>8 = ASHA आशा<br>9 = AWW आँगनवाड़ी कार्यकर्ता<br>10 = SBA/TRAINED DAI एसबीए/प्रशिक्षित दाई<br>11 = DAI दाई<br>12 = RMP आर.एम.पी. (झोला छाप डाक्टर)<br>88 = OTHER (SPECIFY) अन्य (स्पष्ट करें)
V9.	Where all did you receive antenatal checkup during your pregnancy?<br>आपकी प्रसव–पूर्व जाँच कहाँ हुई थी?<br><br>**PROBE : ANY OTHERS?**<br>çkc % dN vkj\<br><br>**RECORD ALL MENTIONED**<br>lHkh mÙkj fy[kA	1 = YOUR HOME अपना घर<br>2 = PARENT'S HOME माता–पिता का घर<br>3 = GOVERNMENT/MUNICIPAL HOSPITAL सरकारी/नगर निगम हस्पताल<br>4 = GOVERNMENT DISPENSARY सरकारी डिस्पेंसरी<br>5 = UHC/UHP/UFWC यूएचसी/यूएचपी/यूएफडब्ल्यूसी<br>6 = CHC सीएचसी<br>7 = APHC/NPHC/BPHC एपीएचसी/एनपीएचसी/बीपीएचसी<br>8 = SUB-CENTER उपकेन्द्र<br>9 = ANGANWADI CENTER आँगनवाड़ी केन्द्र<br>10 = VHND ग्राम स्वास्थ्य एवं पोष्ण दिवस<br>11 = NGO HOSPITAL/CLINIC गैर सरकारी संस्था का हस्पताल/क्लीनिक<br>12 = PRIVATE HOSPITAL/CLINIC प्राइवेट हस्पताल/क्लीनिक<br>88 = OTHERS (SPECIFY) अन्य (स्पष्ट करें)
V10.	During this pregnancy, how many months pregnant were you when you received antenatal checkup for the first time?<br>आपकी पहली बार प्रसव–पूर्व जाँच हुई तब आप कितने महीने गर्भवती थी?<br><br>**CODE IN COMPLETED MONTHS**<br>i.k eghuk e fy[kA	☐ COMPLETED MONTHS पूर्ण महीने<br>99 = DO NOT KNOW पता नहीं
V11.	During this pregnancy, how many times did you receive antenatal checkup?<br>इस गर्भवस्था के दौरान आपकी कितनी बार प्रसव–पूर्व जाँच हुई है?	☐ NO. OF TIMES इतनी बार<br>99 = DO NOT KNOW पता नहीं
V12.	During this pregnancy, as part of your antenatal check-ups were any of the following done at least once?<br>क्या आपकी प्रसव–पूर्व जाँच के रूप में निम्नलिखित जाँच कम से कम एक बार की गयी थी?	

	READ EACH ITEM BELOW (a-h) AND CODE YES OR NO FOR EACH. IF 'NO' GO TO NEXT LETTER<br>¼,&,p½ rd i<s vkj ÁR;sd dks fy, gka ;k ugha dk dksM djsA ;fn ugha gS vxys v{kj ij tk;sA				
	Tests<br>जाँचें	YES<br>हाँ	NO<br>नहीं	DO NOT KNOW पता नहीं	
a.	Were you weighed? क्या आपका वजन लिया गया था?	1	0	99	
b.	Was your blood pressure measured? क्या आपके रक्तचाप (ब्लड प्रेशर) की जाँच की गई थी?	1	0	99	
c.	Did you give a urine sample? क्या आपने पिशाप का नमूना दिया था?	1	0	99	
d.	Was your blood checked for hemoglobin level? हीमोग्लोबिन का स्तर जानने के लिये क्या आपके खून कि जाँच की गयी थी?	1	0	99	
e.	Was your abdomen checked? क्या आपके पेट की जाँच की गई थी?	1	0	99	
f.	Was your ultrasound done? क्या आपका अल्ट्रासाउंड किया गया था?	1	0	99	

V13.	During this pregnancy, were you given a TT injection?<br>इस गर्भवस्था के दौरान क्या आपको टिटनस की सुई लगी थी?	1 = YES हाँ<br>0 = NO नहीं → **GO TO V15**<br>99 = DO NOT KNOW पता नहीं → **GO TO V15**
V14.	During this pregnancy, how many times did you receive a tetanus injection?<br>इस गर्भवस्था के दौरान आपको कितने टिटनस की सुई लगी थी?	☐☐ NO. OF TIMES कितनी बार<br>99 = DO NOT KNOW/REMEMBER पता नहीं/याद नहीं
**V14A**	During this pregnancy, was your blood checked for Hemoglobin during the first three months?<br>गर्भवस्था के दौरान क्या पहले तीन महीने में आपके खून की जांच हुई थी, हीमोग्लोबिन के लिए?	1 = YES हाँ<br>0 = NO नहीं → **GO TO V14C**<br>99 = DO NOT KNOW पता नहीं → **GO TO V14C**
**V14B**	ASK IF YES FOR V12D OR V14A<br>After your blood was checked, were you informed regarding your haemoglobin status/ if you are anaemic or not?<br><br>खून की जांच के बाद क्या आपको हीमोग्लोबिन की स्थिति बताई गयी थी?	1 = YES हाँ<br>0 = NO नहीं
**V14C**	Have you been identified as anaemic?<br>क्या आपको खून की कमी है, ऐसा बताया गया था जांच के बाद?	1 = YES हाँ<br>0 = NO नहीं
V15.	During this pregnancy, did you receive any Iron Folic Acid (IFA) tablets?<br>इस गर्भवस्था के दौरान क्या आपको आई.एफ.ए. (ताकत की लाल गोली) की गोलियाँ मिली है?<br><br>**SHOW SAMPLES OF IFA TABLETS**<br>vkbZ-,Q-,- dh xkfy;ksa ds uewus fn[kk;sA	1 = YES हाँ<br>0 = NO नहीं → **GO TO V19**<br>99 = DO NOT KNOW पता नहीं → **GO TO V19**
V16.	In which month of pregnancy did you receive IFA tablets for the first time?<br>गर्भावस्था के किस महीने में आपको पहली बार आई.एफ.ए. (ताकत की लाल गोली) की गोलियाँ मिली थी?	☐ COMPLETED MONTHS पूर्ण महीने<br>99 = DO NOT KNOW पता नहीं
V17.	How many tablets did you receive in total during this pregnancy?आपको आई.एफ.ए. (ताकत की लाल गोली) की कितनी गोलियाँ मिली थी?<br><br>**PROBE FOR WHETHER TABLETS ARE ADULT (LARGE) OR PEDIATRIC (SMALL) DOSE.**<br>iNs fd ;g xkfy;ka cM+h Fkh ;k NksVh FkhA	☐☐☐ NUMBER OF LARGE TABLETS बड़ी गोलियों की संख्या<br>☐☐☐ NUMBER OF SMALL TABLETSछोटी गोलियों की संख्या<br>999 = DO NOT REMEMBER याद नहीं
V18.	During this pregnancy, how many tablets did you consume?<br>आपने आई.एफ.ए. (ताकत की लाल गोली) की कितनी गोलियाँ खायी थी?	☐☐☐ NUMBER OF LARGE TABLETS बड़ी गोलियों की संख्या<br>☐☐☐ NUMBER OF SMALL TABLETSछोटी गोलियों की संख्या<br>999 = DO NOT REMEMBER याद नहीं
V19.	During this pregnancy, were you given or did you buy any iron folic syrup?<br><br>इस गर्भवस्था के दौरान क्या आपको आईरन फोलिक एसिड (ताकत) की सिरप मिली थी या आपने खरीदी है?	1 = YES हाँ<br>0 = NO नहीं
**V19A**	In which month of pregnancy did you receive IFA syrup bottles for the first time?	☐ COMPLETED MONTHS पूर्ण महीने<br>DO NOT KNOW पता नहीं

	गर्भावस्था के कौनसे महीने में आपको पहली बार IFA सिरप की बोतले मिली थी?	
**V19B**	How many IFA syrup bottles did you receive in total during your whole pregnancy?<br><br>आई.एफ.ए. की सिरप की कितनी बोतलें मिली थी?	☐☐.☐ NUMBER OF BOTTLES<br>DO NOT REMEMBER
**V19C**	During the whole pregnancy, how many bottles did you consume?<br><br>आपने आई.एफ.ए. की सिरप की कितनी बोतलें खायी थी?	☐☐. ☐ NUMBER OF BOTTLES<br>= DO NOT REMEMBER
**V19D**	**ASK IF V18>0 OR V19C>0, ELSE GO TO V21**<br><br>Do you take the tablets and syrup daily?<br>**क्या आप रोजाना गोलिया या सिरप लेती है?**	IFA tablets(small or large): 1 = YES हाँ, 0 = NO नहीं<br>IFA syrup: 1 = YES हाँ, 0 = NO नहीं
V20.	**ASK IF V18>0 OR V19C>0, ELSE GO TO V2**<br>During this pregnancy, for how many days did you take the tablets or syrup?<br>गर्भावस्थ के दौरान आपने कितने दिनों तक आईरन फोलिक एसिड (ताकत) की गोली या सिरप खायी थी?<br><br>**IF ANSWER IS NOT NUMERIC, PROBE FOR APPROXIMATE NUMBER OF DAYS.**<br>;fn mÙkj l[;k esa ugha g] iNsa fd fnuksa dh l[;k yxHkx D;k FkhA	☐☐☐ NO OF DAYS FOR LARGE TABLETS बड़ी गोलियाँ खाने के दिनों की संख्या<br>☐☐☐ NO OF DAYS FOR SMALL TABLETS छोटी गोलियों दिनों की संख्या<br>☐☐☐ NO OF DAYS FOR SYRUP सिरप खाने के दिनों की संख्या<br>999 = DO NOT REMEMBER याद नहीं
V22.	During this pregnancy, were you given or did you buy any calcium tablets?<br>गर्भावस्थ के दौरान , क्या आपको केलसियम की गोली मिली थी या आपने खरीदी थी?	1 = YES हाँ<br>0 = NO नहीं → **GO TO V25**
V23.	During this pregnancy, how many calcium tablets were you given or did you buy?<br>गर्भावस्थ के दौरान , आपको केलसियम की कितनी गोली मिली थी या आपने खरीदी थी?	☐☐☐ NUMBER OF TABLETS गोलियों की संख्या<br>999 = DO NOT REMEMBER याद नहीं
V24.	During this pregnancy, how many tablets did you consume?<br>गर्भावस्थ के दौरान आपने केलसियम की कितनी गोलियाँ खायी थी?	☐☐☐ NUMBER OF TABLETS गोलियों की संख्या<br>999 DO NOT REMEMBER याद नहीं
**V24a**	Do you take the tablets daily?<br>**क्या आप रोजाना गोलिया लेती है?**	1 = YES हाँ<br>0 = NO नहीं
**V24b**	**ASK IF V24>0**<br>During the whole pregnancy, for how many days did you take the tablets? गर्भावस्थ के दौरान आपने कितने दिनों तक गोली खायी थी?<br><br>**IF ANSWER IS NOT NUMERIC, PROBE FOR APPROXIMATE NUMBER OF DAYS.**<br>;fn mÙkj l[;k esa ugha g] iNsa fd fnuksa dh l[;k yxHkx D;k FkhA	☐☐☐ NO OF DAYS FOR TABLETS गोलियाँ खाने के दिनों की संख्या<br>999=DO NOT REMEMBER याद नहीं
**V24C**	During this pregnancy, are you taking any multi micronutrient tablets/syrup?<br>**इस गर्भवस्था के दौरान क्या आप मुलती माइक्रो नुत्रिएन्त की गोलिया या सिरप ले रही है?**<br><br>SHOW SAMPLES OF MMN	1 = YES हाँ<br>0 = NO नहीं→ **GO TO V25**
**V24D**	Do you take the tablet daily?<br>**क्या आप रोजाना यह गोलियां लेती है?**	1 = YES हाँ<br>0 = NO नहीं
V25.	During this pregnancy, did you ever receive any Take Home Ration from the Anganwadi Center?<br>इस गर्भवस्था के दौरान क्या आपको कभी भी आँगनवाड़ी केन्द्र से पंजीरी मिली थी?	1 = YES हाँ<br>0 = NO नहीं
V26.	During this pregnancy, did you take rest more frequently than when you were not pregnant?<br>इस गर्भवस्था के दौरान क्या आप गर्भवती न होने के समय की तुलना में अधिक बार आराम करती थी?	1 = YES हाँ<br>0 = NO नहीं
V27.	During this pregnancy, did you consume more food as compared to when you were not pregnant?	1 = YES हाँ<br>0 = NO नहीं

	इस गर्भवस्था के दौरान क्या आप गर्भवती न होने के समय की तुलना में अधिक भोजन करती थी?	
**V27a.**	Which of the following foods did you consume almost daily during pregnancy?<br><br>कौनसे पढके सुनाई जाने वाली चीज़ें आपने रोजाना गर्भवस्था के दौरान खाया?<br><br>MULTIPLE CHOICE	1= Dark green leafy vegetables हरि सब्जियां<br>2=Yellow or orange fruits or vegetables (e.g. Pumpkin, carrots, ripe mango, ripe papaya) पीली या नारंगी फल और सब्जी<br>3=Lentils, dal दल<br>4=Roti, bread or rice रोटी, चावल, ब्रेड<br>5=Eggs अन्दा<br>6=Meat, chicken or fish मॉस<br>7=Milk, paneer or yogurt दूध, पनीर, दही<br>88=Othersअन्य
**V27b.**	How many kgs. did you gain during pregnancy?<br>गर्भवस्था के दौरान कितना वजन बढ़ा?	__ KGS<br>99 = DO NOT KNOW पता नहीं
V28.	During this pregnancy, did you avoid picking up heavy objects?<br>इस गर्भवस्था के दौरान क्या आप भारी सामान उठाने से बचती थी?	1 = YES हाँ<br>0 = NO नही
Read out to the respondent: *Now, I would ask you about awareness of different danger signs during pregnancy and complications that a woman may experience during her pregnancy.*<br>mÙkjnkrk dk i<dj lquk;s vc eS vkils xHkkZoLFkk ds nkjku [krjs ds fofHkUu y{k.kks vkj tfVyrkvks dh tkx:drk ds ckjs esa iNuk pkgwxh] tks ,d efgyk xHkkZoLFkk ds nkSjku vuHko dj ldrh g\		
V29.	Are you aware about the different danger signs during pregnancy which require visit to a health facility or contacting a healthcare provider?<br>क्या आप गर्भावस्था के दौरान होने वाले खतरे के विभिन्न लक्षणों के बारे में जानती हैं जिनके लिए स्वास्थ्य केंद्र जाने की या स्वास्थ्य प्रदाता से संपर्क करने की जरूरत होती है?	1 = YES हाँ<br>0 = NO नहीं → **GO TO V32**
V30.	What are the different danger signs during pregnancy which require visit to a health facility or contacting a healthcare provider?<br>गर्भावस्था के दौरान खतरे के वह कौन से लक्षण हैं जिनके लिए स्वास्थ्य केंद्र जाने की या स्वास्थ्य प्रदाता से संपर्क करने की जरूरत होती है?<br><br>**PROBE : ANY OTHERS?**<br>çkc % dN vkj\<br><br>**RECORD ALL MENTIONED**<br>lHkh mÙkj fy[kA	1 = EXCESSIVE VAGINAL BLEEDING योनि से अत्यधिक खून निकलना<br>2 = DIFFICULTY IN BREATHING सांस लेन में कठिनाई<br>3 = SEVERE HEADACHE तेज सिर दर्द<br>4 = BLURRED VISION धुंधला दिखना<br>5 = SWELLING OF THE HANDS, FEET, BODY OR FACE हाथों, पैरों, शरीर और चेहरे का सूजन<br>6 = HIGH FEVER तेज बुखार<br>7 = LOSS OF CONSCIOUSNESS होश खोना<br>8 = SEVERE ABDOMINAL PAIN पेट में तेज दर्द<br>9 = CONVULSIONS दौरे पड़ना<br>10 = HIGH BLOOD PRESSURE उच्च रक्तचाप<br>88 = OTHERS (SPECIFY) अन्य (स्पष्ट करें)
V31.	From where did you get the information about these danger signs during pregnancy?<br>गर्भावस्था के दौरान खतरे के लक्षणों के बारे में आपको कहाँ से जानकारी मिली थी?<br><br>**PROBE : ANY OTHERS?**<br>çkc % dN vkj\<br><br>**RECORD ALL MENTIONED**<br>lHkh mÙkj fy[kA	1 = GOVERNMENT DOCTOR सरकारी डाक्टर<br>2 = PRIVATE DOCTOR प्राइवेट डाक्टर<br>3 = STAFF NURSE स्टाफ नर्स<br>4 = LHV लेडी हैल्थ विजिटर (एल.एच.वी.)<br>5 = MALE HEALTH WORKER पुरुष स्वास्थ्य कर्मी<br>6 = ANM ए.एन.एम.<br>7 = OTHER HEALTH PERSONNEL अन्य स्वास्थ्य कर्मी<br>8 = ASHA आशा<br>9 = AWW आँगनवाड़ी कार्यकर्ता<br>10 = SBA/TRAINED DAI एसबीए/प्रशिक्षित दाई<br>11 = DAI दाई<br>12 = RMP आर.एम.पी. (झोला छाप डाक्टर)<br>13 = FAMILY MEMBER परिवार के सदस्य<br>14 = FRIENDS/NEIGHBOUR दोस्त/पड़ौसी<br>15 = RADIO/TV/NEWS PAPER रेडियो/टेलिविजन/अखबार<br>16 = MOBILE SMS मोबाइल एसएमएस<br>88 = OTHERS (SPECIFY) अन्य (स्पष्ट करें)

		98 = NO ONE कोई नहीं<br>99 = DO NOT KNOW पता नहीं		
V32.	During this pregnancy, were you told to visit a doctor/facility in case of any pregnancy complications?<br>गर्भावस्था के दौरान क्या आपको यह बताया गया था कि गर्भावस्था से संबंधित किसी भी परेशानी के मामले में डाक्टर के पास/स्वास्थ्य केंद्र पर जायें?	1 = YES हाँ<br>0 = NO नहीं		
V33.	During this pregnancy, what kind of problems did you experience?<br>इस गर्भवस्था के दौरान क्या आपको किसी प्रकार की परेशानी हुई है?<br><br>**DO NOT READ LIST. CODE ALL MENTIONS IN FIRST COLUMN. THEN READ EACH ITEM BELOW (a-j) THAT WASN'T MENTIONED AND CODE RESPONSE IN SECOND COLUMN. IF NOT MENTIONED, SKIP TO NEXT LETTER.**<br>lph dk u i<A igy dkye e lHkh mYy[k fd; x; mÙkjk dk dkM djA bld ckn mu fcUnvk ¼,&t½ dk i< ftudk mYy[k ugh fd;k x;k Fkk vkj nlj dkye e mÙkj dk dkM djA ;fn ugh crk;k x;k vxy fcn ij tk;A	1 = YES, SPONTANEOUS<br>हाँ, स्वयं बताया	2 = YES, AFTER READING<br>हाँ, पढ़ने के बाद	0 = NO<br>नहीं
a.	Excessive vaginal bleeding योनी से अत्यधिक रक्तस्राव	1	2	0
b.	Smelly vaginal discharge योनि से बदबूदार पानी निकलना	1	2	0
c.	Swelling of the hands, feet, body or face हाथों, पैरों, शरीर और चेहरे पर सूजन	1	2	0
d.	Headache सिर दर्द	1	2	0
e.	Blurred vision धुंधला दिखना	1	2	0
f.	Convulsions दौरे पड़ना	1	2	0
g.	Febrile illness बुखार जिसका कारण पता नहीं	1	2	0
h.	Severe abdominal pain that is not labor pain पेट में तेज दर्द जो प्रसव पीड़ा नहीं थी	1	2	0
i.	Pallor चेहरे का फीकापन	1	2	0
j.	Difficulty in breathing साँस लेन में कठिनाई	1	2	0
k.	Swelling of the hands, feet, body or face हाथों, पैरों, शरीर और चेहरे पर सूजन	1	2	0
l.	High fever तेज बुखार	1	2	0
m.	Loss of consciousness चेतना (होश) खोना	1	2	0
n.	High Blood Pressure उच्च रक्तचाप	1	2	0
o.	Heart disease दिल की बीमारी	1	2	0
p.	Diabetes मधुमेय	1	2	0
q.	Other medically diagnosed disease कोई बीमारी जो चिकित्सक द्वारा बतायी गयी है	1	2	0
V34.	**ASK ONLY IF ANY OF THE RESPONSES IN V33(a-hh) or V33A1 IS CODED 1 OR 2**<br><br>Did you seek treatment for this problem?<br>क्या आपने इस समस्या का इलाज कराया था?	1 = YES हाँ<br>0 = NO नहीं → **GO TO D36**		
V35.	From where did you seek treatment?<br>आपने इलाज कहाँ से कराया था?<br><br>**PROBE : ANY OTHERS?**<br>çkc % dN vkj\<br><br>**RECORD ALL MENTIONED**<br>lHkh mÙkj fy[kA	1 = GOVERNMENT/MUNICIPAL HOSPITAL सरकारी/नगरपालिका का अस्पताल<br>2 = GOVERNMENT DISPENSARY सरकारी दवाखाना<br>3 = UHC/UHP/UFWC यूएचसी/यूएचपी/यूएफडब्ल्यूसी<br>4 = CHC सीएचसी<br>5 = APHC/NPHC/BPHC एपीएचसी/एनपीएचसी/बीपीएचसी<br>6 = SUB-CENTER उपकेन्द्र<br>7 = ANGANWADI CENTER आँगनवाड़ी केन्द्र<br>8 = VHND ग्राम स्वास्थ्य एवं पोष्ण दिवस<br>9 = NGO HOSPITAL/CLINIC गैर सरकारी संस्था का अस्पताल/क्लीनिक<br>10 = PRIVATE HOSPITAL/CLINIC प्राइवेट अस्पताल/क्लीनिक<br>11 = MEDICINE SHOP दवा की दुकान<br>12 = FOLK HEALER ओझा/वैद्य/झाड़फूँक		

		13 = HOME REMEDIES घरेलू उपचार<br>14 = RMP आर.एम.पी. (झोला छाप डाक्टर)<br>88 = OTHERS (SPECIFY) अन्य (स्पष्ट करें)
**V35a**	During this pregnancy, did you receive any dose of Sp/Fansidar, which is used for **preventing** malaria?<br><br>इस गर्भवस्था के दौरान क्या आपको फंसिदर का कोई दोसे मिला था, जो मलेरिया से बचने के लिए लगाया जाता है?<br><br>**Show Sp/Fansidar sample**	1 = YES हाँ<br>0 = NO नहीं → **GO TO V35c**<br>9 = DO NOT KNOW पता नहीं → **GO TO V35c**
**V35b**	How many doses did you get?<br>**कितने दोसे मिले थे?**	☐NUMBER
**V35c**	During this pregnancy, did you have malaria?<br>**इस गर्भवस्था में क्या आपको मलेरिया हुआ है?**	1 = YES हाँ<br>0 = NO नहीं → **GO TO V35g**
**V35d**	Did you seek treatment for malaria?<br>**क्या आपने मलेरिया के लिए इलाज करवाया है?**	1 = YES हाँ<br>0 = NO नहीं→ **GO TO V35g**
**V35e**	From where did you seek treatment for malaria?<br>**इलाज कहाँ से करवाया है?**	1 = GOVERNMENT/MUNICIPAL HOSPITAL सरकारी/नगरपालिका का अस्पताल<br>2 = GOVERNMENT DISPENSARY सरकारी दवाखाना<br>3 = UHC/UHP/UFWC यूएचसी/यूएचपी/यूएफडब्ल्यूसी<br>4 = CHC सीएचसी<br>5 = APHC/NPHC/BPHC एपीएचसी/एनपीएचसी/बीपीएचसी<br>6 = SUB-CENTER उपकेन्द्र<br>7 = ANGANWADI CENTER आँगनवाड़ी केन्द्र<br>8 = VHND ग्राम स्वास्थ्य एवं पोष्ण दिवस<br>9 = NGO HOSPITAL/CLINIC गैर सरकारी संस्था का अस्पताल/क्लीनिक<br>10 = PRIVATE HOSPITAL/CLINIC प्राइवेट अस्पताल/क्लीनिक<br>11 = MEDICINE SHOP दवा की दुकान<br>12 = FOLK HEALER ओझा/वैद्य/झाड़फूँक<br>13 = HOME REMEDIES घरेलू उपचार<br>14 = RMP आर.एम.पी. (झोला छाप डाक्टर)<br>88 = OTHERS (SPECIFY) अन्य (स्पष्ट करें)
**V35f**	Were you given quinine or ACT, a medicine for treating malaria?<br>**क्या आपको मलेरिया की दवाई मिली थी?**<br><br>**SHOW SAMPLES OF QUININE or ACT**	1 = YES हाँ<br>0 = NO नहीं<br>9 = DO NOT KNOW पता नहीं
**V35g**	**During this pregnancy, were you tested for syphillis?**<br><br>इस गर्भवस्था में क्या आपको उपदंश के लिए टेस्ट करवाया है?(कोई ऐसी बिमारी जिसमें योनी के आस पास छाले पड़े हो)	1 = YES tested positive<br>जांच की गयी और पाया गया के उपदंश है<br>2 =YES tested negative → **GO TO V35k**<br>जांच की गयी और पाया गया की उपदंश नहीं है<br>0 = NO नहीं → **GO TO V35k**<br>99= DO NOT KNOW → **GO TO V35k**
**V35h**	**Did you seek treatment for syphilis?**<br>उपदंश का इलाज़ करवाया?	1 = YES हाँ<br>0 = NO नहीं→ **GO TO V35j**
**V35i**	From where did you seek treatment?<br>आपने इलाज कहाँ से कराया था?<br><br>**PROBE : ANY OTHERS?**<br>çkc % dqN vkj\<br><br>**RECORD ALL MENTIONED**<br>lHkh mÙkj fy[kA	1 = GOVERNMENT/MUNICIPAL HOSPITAL सरकारी/नगरपालिका का अस्पताल<br>2 = GOVERNMENT DISPENSARY सरकारी दवाखाना<br>3 = UHC/UHP/UFWC यूएचसी/यूएचपी/यूएफडब्ल्यूसी<br>4 = CHC सीएचसी<br>5 = APHC/NPHC/BPHC एपीएचसी/एनपीएचसी/बीपीएचसी<br>6 = SUB-CENTER उपकेन्द्र

		7 = ANGANWADI CENTER आँगनवाड़ी केन्द्र<br>8 = VHND ग्राम स्वास्थ्य एवं पोषण दिवस<br>9 = NGO HOSPITAL/CLINIC गैर सरकारी संस्था का अस्पताल/क्लीनिक<br>10 = PRIVATE HOSPITAL/CLINIC प्राइवेट अस्पताल/क्लीनिक<br>11 = MEDICINE SHOP दवा की दुकान<br>12 = FOLK HEALER ओझा/वैद्य/झाड़फूँक<br>13 = HOME REMEDIES घरेलू उपचार<br>14 = RMP आर.एम.पी. (झोला छाप डाक्टर)<br>88 = OTHERS (SPECIFY) अन्य (स्पष्ट करें)
**V35j**	Were you given a Penicillin injection/ tablet as treatment for syphilis?<br><br>**क्या आपको उपदंश के इलाज़ के लिए पेनिसिलिन जी का सुई लगाया गया था?**	0 = YES penicillin injection हाँ सुई लगी थी<br>1 =YES tablet हाँ टेबलेट लिया था<br>0 = NO नह<br>9 = DO NOT KNOW पता नहीं ीं
**V35k**	Were you screened for diabetes during this pregnancy?<br>**क्या इस गर्भवस्था के दौरान आपका डायबिटीज के लिए जांच की गयी थी ?**	1 = YES हाँ<br>0 = NO नहीं→ **GO TO V39**<br>9 = DO NOT KNOW पता नही → **GO TO V39**
**V35l**	Were you tested positive for diabetes during this pregnancy?<br>**क्या इस गर्भवस्था के दौरान जाँच के बाद पाया गया था के आपको डायबिटीज है ?**	1 = YES हाँ<br>0 = NO नहीं → **GO TO V39**
**V35m**	**Did you seek treatment for diabetes?**<br>इलाज़ करवाया?	1 = YES हाँ<br>0 = NO नहीं GOTO V35o
**V35n**	From where did you seek treatment?<br>आपने इलाज कहाँ से कराया था?<br><br>**PROBE : ANY OTHERS?**<br>çkc % dN vkj\<br><br>**RECORD ALL MENTIONED**<br>lHkh mÙkj fy[kA	1 = GOVERNMENT/MUNICIPAL HOSPITAL सरकारी/नगरपालिका का अस्पताल<br>2 = GOVERNMENT DISPENSARY सरकारी दवाखाना<br>3 = UHC/UHP/UFWC यूएचसी/यूएचपी/यूएफडब्ल्यूसी<br>4 = CHC सीएचसी<br>5 = APHC/NPHC/BPHC एपीएचसी/एनपीएचसी/बीपीएचसी<br>6 = SUB-CENTER उपकेन्द्र<br>7 = ANGANWADI CENTER आँगनवाड़ी केन्द्र<br>8 = VHND ग्राम स्वास्थ्य एवं पोषण दिवस<br>9 = NGO HOSPITAL/CLINIC गैर सरकारी संस्था का अस्पताल/क्लीनिक<br>10 = PRIVATE HOSPITAL/CLINIC प्राइवेट अस्पताल/क्लीनिक<br>11 = MEDICINE SHOP दवा की दुकान<br>12 = FOLK HEALER ओझा/वैद्य/झाड़फूँक<br>13 = HOME REMEDIES घरेलू उपचार<br>14 = RMP आर.एम.पी. (झोला छाप डाक्टर)<br>88 = OTHERS (SPECIFY) अन्य (स्पष्ट करें)
**V35o**	Were you given any anti diabetic pills for diabetes control?<br><br>**क्या आपको डायबिटीज के इलाज़ के लिए गोलिया दी गयी थी?**<br><br>SHOW ANTI DIABETIC PILLS SAMPLES	1 = YES हाँ<br>0 = NO नहीं
**V35p**	Were you given insulin injections for diabetes control?<br>**क्या आपको डायबिटीज के इलाज़ के लिए कोई सुई लगायी गयी थी**	1 = YES हाँ<br>0 = NO नहीं
**V36**	Prior to the delivery, did you plan or intend to deliver [CHILD NAME] at home or in a health facility?<br>प्रसव से पूर्व आपने (बच्चे का नाम) को कहाँ जन्म देने का सोचा था – घर पर या स्वास्थ्य केन्द्र मे?	1 = AT HOME घर पर<br>2 = IN A HEALTH FACILITY स्वास्थ्य केंद्र में<br>3 = DID NOT PLAN योजना नहीं बनाई
**V37**	Did you discuss plans for your delivery with [CHILD NAME's] father?	1 = YES हाँ<br>0 = NO नहीं

	क्या आपने प्रसव के संबंध में अपनी योजनाओं के बारे में (बच्चे का नाम) के पिता से विचार–विमर्श किया था?	9 = NOT APPLICABLE लागू नहीं
**V38**	Did you discuss your plans for your delivery with your mother-in-law?<br>क्या आपने प्रसव के संबंध में अपनी योजनाओं के बारे में अपनी सास से विचार–विमर्श किया था?	1 = YES हाँ<br>0 = NO नहीं<br>= NOT APPLICABLE लागू नहीं
Read out to the respondent: Now, I would like to ask you about preparations or decisions you made for the delivery of [CHILD NAME].<br>mÙkjnkrk dks i<dj lquk;s vc eSa vkils ¼cPps dk uke½ ds tUe ds ckjs esa dh xbZ rS;kfj;ksa vkSj fy;s x;s QSlyksa ds ckjs esa iwNuk pkgwaxhA		
Read out to the respondent Now I'd like to talk to you in more detail about contacts you had with health service providers such as ASHAs, AWWs, ANMs, LHVs and Government doctors either at home<br>mÙkjnkrk dks i<dj lquk;s vc eSa vkils vkids }kjk ?kj ij ;k vU; dgha vk'kk] vkxuokMh dk;ZdrkZ] ,-,u-,e-] ,y,poh ;k ljdkjh MkDVj tSls LokLF; lsok çnkrkvksa ds lkFk dh xbZ HksVksa ds fooj.k tkuuk pkgwaxhA		
V39.	Did the ASHA come to meet you at your home during your pregnancy?<br>इस गर्भवस्था के दौरान क्या आशा आपके घर आपसे मिलने आई है?	1 = YES हाँ<br>0 = NO नहीं → **GO TO V43**
V40.	How many times did the ASHA come to meet you at your home during the pregnancy?<br>इस गर्भवस्था के दौरान आशा आपके घर आपसे मिलने कितनी बार आई है ?	☐☐ NO. OF TIMES इतनी बार<br>99 = DO NOT REMEMBER याद नहीं
V41.	Did the ASHA come to meet you at your home during the last three months of your pregnancy?<br>इस गर्भवस्था के दौरान क्या गर्भावस्था के आखरी तीन महिनों में आशा आपके घर आपसे मिलने आई है?	1 = YES हाँ<br>0 = NO नहीं → **GO TO V43**<br>99= NOT APPLICABLE, MONTH OF PREGNANCY LESS THAN 7 MONTHS लागु नहीं, 7 महीने से कम से गर्भवती है → **GO TO V43**
V42.	How many times did the ASHA come to meet you at your home during the last three months of your pregnancy?<br>गर्भावस्था के आखरी तीन महिनों में आशा आपके घर आपसे मिलने कितनी बार आई थी?	☐☐ NO. OF TIMES इतनी बार<br>99 = DO NOT REMEMBER याद नहीं
V43.	Did the AWW come to meet you at your home during this pregnancy?<br>इस गर्भवस्था के दौरान क्या आँगनवाड़ी कार्यकर्ता आपके घर आपसे मिलने आई है?	1 = YES हाँ<br>0 = NO नहीं → **GO TO** V47
V44.	How many times did the AWW come to meet you at your home during this pregnancy?<br>इस गर्भवस्था के दौरान आँगनवाड़ी कार्यकर्ता आपके घर आपसे मिलने कितनी बार आई है?	☐☐ NO. OF TIMES इतनी बार<br>99 = DO NOT REMEMBER याद नहीं
V45.	Did the AWW come to meet you at your home during the last three months of this pregnancy?<br>गर्भावस्था के आखरी तीन महिनों में आँगनवाड़ी कार्यकर्ता आपके घर आपसे मिलने आई थी?	1 = YES हाँ<br>0 = NO नहीं → **GO TO** V47<br>99= NOT APPLICABLE, MONTH OF PREGNANCY LESS THAN 7 MONTHS लागु नहीं, 7 महीने से कम से गर्भवती है → **GO TO** V47
V46.	How many times did the AWW come to meet you at your home during the last three months of this pregnancy]?<br>आखरी तीन महिनों में आँगनवाड़ी कार्यकर्ता आपके घर आपसे मिलने कितनी बार आई थी?	☐☐ NO. OF TIMES इतनी बार<br>99 = DO NOT REMEMBER याद नहीं
V47.	Did the ANM come to meet you at your home during this pregnancy?<br>इस गर्भवस्था के दौरान क्या ए.एन.एम. आपके घर आपसे मिलने आई थी?	1 = YES हाँ<br>0 = NO नहीं → **GO TO V50a**
V48.	How many times did the ANM come to meet you at your home during this pregnancy?<br>ए.एन.एम. आपके घर आपसे मिलने कितनी बार आई थी?	☐☐ NO. OF TIMES इतनी बार<br>99 = DO NOT REMEMBER याद नहीं
V49.	Did the ANM come to meet you at your home during the last three months of this pregnancy?<br>क्या गर्भावस्था के आखरी तीन महिनों में ए.एन.एम. आपके घर आपसे मिलने आई थी?	1 = YES हाँ<br>0 = NO नहीं → **GO TO V50a**<br>99= NOT APPLICABLE, MONTH OF PREGNANCY LESS THAN 7 MONTHS लागु नहीं, 7 महीने से कम से गर्भवती है → **GO TO V50a**
V50.	How many times did the ANM come to meet you at your home during the last three months of this pregnancy?	☐☐ NO. OF TIMES इतनी बार<br>99 = DO NOT REMEMBER याद नहीं

	जब (बच्चे का नाम) आपगर्भावस्था के आखरी तीन महिनों में ए.एन.एम. आपके घर आपसे मिलने कितनी बार आई थी?	

V50a.	**THINKING ABOUT THE LAST VISIT**<br>Which frontline worker had visited you the last time at home?<br><br>पिछली बार आपको आशा/अन्गंवादी कार्यकर्ता/ऐ एन एम् में से कौन आपको मिलने आपके घर आयें?	1=ASHA<br>2=AWW<br>3=ANM<br>9=NONE
V50b.	How much time the frontline worker who visited you last spend during the session (in minutes)?<br>पिछली बार आपके घर जो कार्यकर्ता आई थी उसने कितना समय बिताया था ? (मिनट में)	|__|__| MINUTES मिनट
V50c.	On what topics did she advise/counsel you?<br><br>आपको उसने चीज़ों के बारें में सलाह दी ?<br><br>**PROBE : ANY OTHERS?**<br>çkc % dN vkj\<br><br>**RECORD ALL MENTIONED**<br>lHkh mÙkj fy[kA	1= MATERNAL NUTRITION मात्री पोषण<br>2= IFA CONSUMPTION IFA की सिरप या गोलियां लेना<br>3= DIETARY DIVERSITY FOR NUTRITION आहार बिबीधता ताकि महिला को ज़रुरत का पोषण मिले<br>4= TAKING CARE OF YOUR HEALTH DURING PREGNANCY गर्भावस्था के दौरान अपने स्वास्थ्य की देखभाल करना<br>5= GOING TO THE HOSPITAL FOR A CHECK-UP DURING PREGNANCY गर्भावस्था के दौरान जांच के लिए अस्पताल जाना<br>6= GOING TO A HEALTH FACILITY FOR DELIVERY प्रसव कराने के लिए स्वास्थ्य केंद्र या अस्पताल जाना<br>7= PLANNING IN ADVANCEFOR DELIVERY प्रसव के लिए पहले से तैयारी किस प्रकार करनी है<br>8= HOW TO TAKE CARE OF THE NEWBORN नवजाति शिशु की देखरेख कैसे करनी है<br>9=EXCLUSIVE BREASTFEEDING केवल स्तनपान<br>10= COMPLEMENTARY FEEDING पूरक आहार / उपरी आहार<br>11= IMMUNIZATION टीकाकरण<br>12= FAMILY PLANNING परिवार नियोजन<br>13= HANDWASHING AND SANITATION हाथ धोना और साफसफाई<br>88= OTHERS (SPECIFY) अन्य (स्पष्ट करें)
V50d.	Was she clear in explaining the topics?<br>क्या वह विषयों को स्पष्ट रूप से समझा पायी थी?	1 = YES हाँ<br>0 = NO नहीं
V50e.	Were you able to understand everything?<br>क्या आप सब कुछ समझ पायी थी?	1 = YES हाँ<br>0 = NO नहीं
V50f.	Was she able to answer all your queries clearly?<br>क्या वह आपके सभी सवालों का स्पष्ट रूप से उत्तर दे पायी थी?	1 = YES हाँ<br>0 = NO नहीं
V50g.	What job aid tools did she use during this most recent visit?<br>आशा/आंगनवाडी कार्यकर्ता ने सबसे हाल के इस दौरे के समय किस साधन या उपकरण का उपयोग किया?<br>**CODE ALL THAT APPLY**<br>lHkh ykxw gksus oky s dkM dj s	1= PLASTIC PICTURE CARDS (describe mobile kunji cards) प्लास्टिक के पिक्चर कार्ड (मोबाइल कुंजी)<br>2= PAMPHLET पम्फलेट<br>3=KATORA/SPOON सी कटोरा/चम्मच<br>4= COPPER-T ई कॉपर–टी<br>5= MALA-D एफ माला–डी<br>88=OTHERS (SPECIFY) अन्य (बताएं)

V50h.	**If D48G=1, ASK D48H. ELSE GO TO D48I**<br><br>Did you find the usage of these tools useful?<br>क्या आपने इन उपकरणों को उपयोगी पाया?	YES हाँ...1<br>NO नहीं..............0
V50i.	Which of these tools helped you to understand the issues explained by FLW?<br>इनमें से कौन कौन से उपकरणों से आपको अग्रणी कतार की कार्यकर्ता (एफएलडब्ल्यू) द्वारा स्पष्ट किये गये मुद्दों को समझने में मदद मिली?<br><br>**CODE ALL THAT APPLY**<br>lHkh ykxw gksus okys dksM djs	1= PLASTIC PICTURE CARDS (describe mobile kunji cards) प्लास्टिक के पिक्चर कार्ड (मोबाइल कुंजी)<br>2= PAMPHLET पम्फलेट<br>3=KATORA/SPOON सी कटोरा/चम्मच<br>4= COPPER-T ई कॉपर–टी<br>5= MALA-D एफ माला–डी<br>88=OTHERS (SPECIFY) अन्य (बताएं)

**V52**	Did the LHV/Government Doctor come to meet you at your home during your pregnancy?<br>क्या एल.एच.वी./सरकारी डाक्टर आपके घर आपसे मिलने आये थे?	1 = YES हाँ<br>0 = NO नहीं → **GO TO V**54		
**V53**	Did the LHV/Government Doctor come to meet you at your home during the last three months of your pregnancy?<br>क्या गर्भावस्था के आखरी तीन महिनों में एल.एच.वी./सरकारी डाक्टर आपके घर आपसे मिलने आये थे?	1 = YES हाँ<br>0 = NO नहीं → **GO TO V**54<br>99= NOT APPLICABLE, MONTH OF PREGNANCY LESS THAN 7 MONTHS लागु नहीं, 7 महीने से कम महीने से गर्भवती है→ **GO TO V**54		
**V54**	How many times did the LHV/Government Doctor come to meet you at your home during the last three months of your pregnancy?<br>गर्भावस्था के आखरी तीन महिनों में एल.एच.वी./सरकारी डाक्टर आपके घर आपसे मिलने कितनी बार आये है?	1 = YES हाँ<br>0 = NO नहीं		
**V55**	During this pregnancy, did anyone tell you about your expected delivery date?<br>इस गर्भवस्था के दौरान क्या किसी ने आपको आपकी प्रसव की सुभावित तिथि बतायी है?	1 = YES हाँ<br>0 = NO नहीं		
**V56**	During your pregnancy , what advice did you receive from ASHA/AWW/ANM related to your pregnancy/delivery during home visits?<br>क्या आपको आपकी गर्भावस्था/प्रसव के बारे में आशा/आँगनवाड़ी कार्यकर्ता/ए.एन.एम. ने से क्या सलाह मिली थी?<br><br>**DO NOT READ LIST. CODE ALL MENTIONS IN FIRST COLUMN. THEN READ EACH ITEM BELOW (a-p) THAT WASN'T MENTIONED AND CODE RESPONSE IN SECOND COLUMN. IF NOT MENTIONED, SKIP TO NEXT LETTER.**<br>lph dk u i<A igys dkye e lHkh mYys[k fd;s x;s mÙkjk dk dkM djA bl d ckn mu fcUnvk ¼,&ih dk i<s ftudk mYys[k ugh fd;k x;k Fkk vkj nlj dkye e mÙkj dk dkM djA ;fn ugh crk;k x;k rc vxy fcn ij tk;A<br><br>**Note to investigator: Please note that these are focused on advice on what the woman needs to do to take care of her health during pregnancy and prepare for delivery. Tell the woman you will ask about advice on caring for her child next.**<br>bofLVxsVj d fy, fVIi.kh d i;k /;ku n fd budk e[; lc/k xHk/kkj.k d nkjku vkj Álo dh r;kjh d le; viu LokLF; dh n[kj[k gr efgyk d fy, tk vko';d g mll lcf/kr lykg l gA mÙkjnkrk dk crk;s fd bld ckn vki mll cPp dh n[kj[k lc/kh lykg d ckj e iNxA	1 = YES, SPONTANEOUS<br>हाँ, स्वयं बताया	2 = YES, AFTER READING<br>हाँ, पढ़ने के बाद	0 = DID NOT RECEIVE ANY ADVICE<br>कोई सलाह नहीं मिली
a.	Taking rest आराम करना	1	2	0
b.	Eating more/healthy food अधिक/स्वास्थ्यकारी भोजन करना	1	2	0
b1	Dietary diversity आहार बिबीधता	1	2	0

B1a	Quantity of food to consume खाने की मात्रअ			
B1b	Weight to gain कितना वजन बढ़ना	1	2	0
c.	Must undergo at least 4 ANC check-ups कम से कम 4 प्रसव–पूर्व जांच कराना	1	2	0
d.	Must take TT injections टिटनस के टीके अवश्य लगाना	1	2	0
e.				
E1	Must consume 180 IFA tablets during pregnancy १८० ifa की गोली लेना	1	2	0
f.	Need of IFA (Iron) tablets during pregnancy गर्भावस्था के दौरान आई.एफ.ए. की गोलियों की जरूरत	1	2	0
F1.	Need of IFA (Iron) tablets after delivery प्रसव के बाद आई.एफ.ए. की गोलियों की जरूरत	1	2	0
F2.	Problems that you may face due to consuming the IFA tablets and syrup आई.एफ.ए. (ताकत की लाल गोली) की गोलियाँ खाने से होने वाली समस्याओं के बारे में	1	2	0
g.	Must consume calcium tablets केलसियम की गोलियाँ अवश्य लेना	1	2	0
h.	Need of calcium tablets during pregnancy गर्भावस्था के दौरान केलसियम की गोलियों की जरूरत	1	2	0
i.	Must consume 180 calcium tablets during pregnancy१८० कैल्शियम की गोलिया लेना	1	2	0
i1.	Need of calcium tablets after delivery प्रसव के बाद केलसियम की गोलियों की जरूरत	1	2	0
j.	Planning for a skilled birth attendant to be present if planning a home delivery यदि घर पर प्रसव कराने की योजना है तब कुशल जन्म सहायक की उपस्थिति की योजना बनाना	1	2	0
k.	Obtaining a new blade for delivery प्रसव के लिए नया ब्लेड लाना	1	2	0
l.	Obtaining a new thread for delivery प्रसव के लिए नया धागा लाना	1	2	0
m.	Saving money for potential complications संभावित जटिलताओं या समस्याओं के लिए पैसे की बचत	1	2	0
n.	Delivering in a health facility स्वास्थ्य सुविधा में प्रसव कराना	1	2	0
o.	Identifying transportation to go to health facility स्वास्थ्य सुविधा जाने के लिए वाहन की पहचान करना	1	2	0
p.	Kept important phone numbers handy, like the phone numbers of the ASHA, hospital, and ambulance आशा, अस्पताल या एंबुलेंस जैसे महत्वपूर्ण फोन नम्बर अपने पास रखना	1	2	0
q.	Staying 48 hours in the health facility after delivery प्रसव के बाद 48 घंटे तक स्वास्थ्य सुविधा में रहना	1	2	0
r.	Information on the danger of excessive vaginal bleeding, foul vaginal discharge, abdominal pain, or fever after delivery योनि से अत्यधिक खून निकलने, योनि से बदबूदार पानी निकलने, पेट में दर्द या प्रसव के बाद बुखार आदि के बारे में जानकारी	1	2	0
s.	Identifying a facility to go to in case of an emergency आपात्कालीन स्थिति होने पर जिस स्वास्थ्य सुविधा में जाना है उसकी पहचान करना	1	2	0
t.	Family planning परिवार नियोजन	1	2	0
**V57**	**ASK IF WOMAN IS PREGNANT FOR 0-6 MONTHS**<br>During this pregnancy, what advice did you receive on caring for your child from ASHA/AWW/ANM ?<br>आशा/आँगनवाड़ी कार्यकर्ता/ए.एन.एम. से आपको बच्चे की देखरेख के बारे में क्या सलाह मिली थी?<br><br>**DO NOT READ LIST. CODE ALL MENTIONS IN FIRST COLUMN. THEN READ EACH ITEM BELOW (a-i) THAT WASN'T MENTIONED AND CODE RESPONSE IN SECOND COLUMN. IF NOT MENTIONED, SKIP TO NEXT LETTER.**<br>lph dk u i<A igys dkye es lHkh mYys[k fd;s x;s mÙkjk dk dkM djA blds ckn mu fcUnqvk ¼,&vkb½ dk i<s ftudk mYys[k ugh fd;k x;k Fkk vkj nljs dkye es mÙkj dk dkM djA ;fn ugh crk;k x;k rc vxys fcUnq ij tk;A	1 = YES, SPONTANEOUS<br>हाँ, स्वयं बताया	2 = YES, AFTER READING<br>हाँ, पढ़ने के बाद	0 = DID NOT RECEIVE ANY ADVICE<br>कोई सलाह नहीं मिली
a.	Not applying anything on the cord नाल पर कुछ नहीं लगाना	1	2	0
b.	How to keep the baby warm बच्चे को गर्म रखना	1	2	0

c.	Skin to skin contact शरीर से सटा कर रखना	1	2	0
d.	Not bathing the baby within 3 days after birth in case of normal baby सामान्य प्रसव के बाद तीन दिनों तक बच्चे को न नहलाना	1	2	0
e.	Not bathing the baby within 7 days after birth in case of low birth weight baby यदि बच्चा अल्प वजन हुआ है सात दिनों तक बच्चे को न नहलाना	1	2	0
f.	Putting baby to breast within one hour after delivery प्रसव के बाद 1 घंटे के अन्दर बच्चे को स्तनपान कराना	1	2	0
G	Not feeding the baby honey, janam gutti, etc., before breastfeeding for the first time पहली बार स्तनपान कराने से पहले बच्चे को शहद, जन्म घुट्टी आदि न देना			
G1	Not feeding the baby honey, janam gutti, etc.,for 3 days after deliveryजन्म के 3 दिन के अन्दर बच्चे को शहद, जन्म घुट्टी आदि न देना	1	2	0
h.	Exclusive breastfeeding till 6 months 6 महीनों तक केवल स्तनपान कराना	1	2	0
i.	Information on the danger signs of the baby like - having trouble breathing, being difficult to wake up, losing interest in breastfeeding, or being cold to the touch<br>बच्चे के खतरे के संकेतों के बारे में जानकारी, जैसे कि सांस लेने में दिक्कत, बच्चे को उठाने मे कठिनाई, स्तनपान में बच्चे की दिलचस्पी न हो, छूने पर उसका शरीर ठंडा लगना	1	2	0
j.	Immunisation of the child बच्चे का टीकाकरण	1	2	0
V64	During this entire duration of your pregnancy, are there any food items you want to/have been wanting to eat but could not eat because it is not available with you?<br><br>इस पूरे गर्भावस्था के दौरान क्या ऐसी कोई चीज़ थी/है जो आप खाना चाहते थे/है पर खा नहीं सके क्यूंकि वोह उपलब्ध नहीं आपके पास?<br><br>**PROBE : ANY OTHERS?**<br>çkc ‘ dN vkj\<br><br>**RECORD ALL MENTIONED**<br>lHkh mÙkj fy[kA	1 = STARCH STAPLE FOOD स्टार्च युक्त भोजन (Rice, Roti, Bread, Pressed rice, Muri, Potato, sweet potato, arbi, Cassava, Banana, any other roots and tubers, noodles, pasta) (चावल, रोटी, ब्रेड, चिवड़ा, कसावा, आलू, शकरकंदी, सूरन, कसावा, केला, अन्य तववजे ंदक जनइमते, नूडल, पास्ता)<br>2 = BEANS AND PEAS फलियाँ और दाल (Beans, peas, Mung dal, Chana dal, Arahar dal, Urad dal, kidney beans, Matter dal, other pulse and Soybeans, Seem, mustard) (फलियाँ, मटर, मूंग दाल, चना दाल, अरहर दाल, उरद दाल, राजमा, मटर दाल, अन्य दाल और सोयाबीन, सेम, सरसों)<br>3 = NUTS AND SEEDS मेवा और बीज (Groundnut, Apricort, Almond, Pista, Coconut, Seesam seed, lotus seed, Jackfruit seed, cashew nut, walnut, mustard seed, sunflower seed, pumpkin seeds) (मुन्गफली, खुबानी, बदाम, पिस्ता, नारियल, तिल का बीज, कमल का बीज, कटहल का बीज, काजू, अखरोड, सरसों का बीज, सूरजमुखी का बीज, कद्दु का बीज)<br>4 = DAIRY दूध या दूध उत्पाद (Milk – cow/goat/buffalo, Powder milk, Liquid packet milk, Curd, Paneer, sewai, kheer, sweets, Butter, Ghee) (दूध – गाय/बकरी/भैस, डिब्बे का दूध, पैकेट का दूध, दही, पनीर, सेवाई, खीर, मिठाई, मक्खन, घी)<br>5 = FLESH FOOD मॉंसाहार भोजन (Meat – Lamb/Beef/pork, Organ meat, chicken, duck, other birds, fish – big/small/shell fish/prawn) (मॉंस – भेंड़/बकरी/गाय/बकरी/भैंस/सूअर, मछली – बड़ी/छोटी/कवचधारी/झींगा)<br>6 = EGGS अन्डा (Eggs – Chicken/duck/other bird) (अन्डा – मुर्गी/बतख/अन्य पक्शी)<br>7 = VITAMIN A DARK GREEN विटामिन ए युक्त हरी सबजियाँ (Leafy vegetable, palak saag, lal saag, green saag, pumpkin saag, mustard saag, matter saag, methi saag, muli saag, any		

		other saag) (पत्तेदार सबजियाँ, पालक का साग, लाल साग, हरा साग, कद्दु का साग, सरसों का साग, मटर का साग, मेथी का साग, मुली का साग, कोई और साग)<br>8 = OTHER VITAMIN A RICH VEGETABLES AND FRUITS अन्य विटामिन ए युक्त सबजियाँ और फल<br>(Ripe mangoes, ripe papaya, jack fruit, ripe tomato, carrot, pumpkin) (पका आम, पका पपीता, कटहल, पका टमाटर, गाजर, कद्दु)<br>9 = OTHER VEGETABLES अन्य सबजियाँ<br>(Green papaya, cabbage, cauliflower, eggplant, onion, radish, bitter gourd, bottle gourd, coriander leaves, tomato, pointed gourd, ladies finger, green banana, garlic, ginger) (हरा पपीता, बन्दगोभी, गोभी, बैगन, पेयाज, मूली, करेला, लौकी, धनिया पत्ती, टमाटर, परवल, भिंडी, हरा केला, लेहसुन, अदरक)<br>10 = OTHER FRUITS अन्य फल<br>(Citrus fruits – orange/lemon/malta, apple, guava, pear, watermelon)<br>(खट्टे फल – संतरा/नीबू/मान्लटा, सेब, नाशपाती, तरबूज़)<br>88=OTHERS (SPECIFY) अन्य (बताएं)<br>99=NONE

R. VERBAL AUTOPSY (FOR STILL BIRTHS/DEATH OF A CHILD) (RC7)				
Read out to the respondent: Now, I would like to ask you some questions about when you were pregnant.<br>mÙkjnkrk dk i<dj lquk;s vc eSa vkils mu le; ds ckjs esa dqN ç'u iNwxh tc vki xHkZorh FkhA				
R1.	During your pregnancy, did you go to see anyone for antenatal checkup?<br>जब आप गर्भवती थी तब क्या प्रसव–पूर्व जाँच के लिए आप किसी के पास गयी थी?	1 = YES हाँ<br>0 = NO नहीं		
R2.	During your pregnancy, did anyone come to you for antenatal checkup?<br>जब आप गर्भवती थी तब क्या प्रसव–पूर्व जाँच के लिए कोई आपके पास आया था?	1 = YES हाँ<br>0 = NO नहीं		
R3.	**IF R1= 0 & R2= 0 SKIP TO R6**<br>Who all did your antenatal checkups during your pregnancy?<br>जब आप गर्भवती थी तब आपकी प्रसव–पूर्व जाँच किस–किस ने की थी?<br><br>**PROBE : ANY OTHERS?**<br>çkc % dqN vkj\<br><br>**RECORD ALL MENTIONED**<br>lHkh mÙkj fy[ksA	1 = GOVERNMENT DOCTOR सरकारी डाक्टर<br>2 = PRIVATE DOCTOR प्राइवेट डाक्टर<br>3 = STAFF NURSE स्टाफ नर्स<br>4 = LHV लेडी हैल्थ विजिटर (एल.एच.वी.)<br>5 = MALE HEALTH WORKER पुरुष स्वास्थ्य कर्मी<br>6 = ANM ए.एन.एम.<br>7 = OTHER HEALTH PERSONNEL अन्य स्वास्थ्य कर्मी<br>8 = ASHA आशा<br>9 = AWW आँगनवाड़ी कार्यकर्ता<br>10 = SBA/TRAINED DAI एसबीए/प्रशिक्षित दाई<br>11 = DAI दाई<br>12 = RMP आर.एम.पी. (झोला छाप डाक्टर)<br>88 = OTHER (SPECIFY) अन्य (स्पष्ट करें)		
R4.	When you were pregnant, how many times did you receive antenatal checkup?<br>जब आप गर्भवती थी तब आपकी कितनी बार प्रसव–पूर्व जाँच हुई थी?	☐ NO. OF TIMES इतनी बार<br>99 = DO NOT KNOW पता नहीं		
R5.	During your pregnancy, as part of your antenatal check-ups were any of the following done at least once?<br>जब आप गर्भवती थी तब क्या आपकी प्रसव–पूर्व जाँच के रूप में निम्नलिखित जाँच कम से कम एक बार की गयी थी?<br><br>**READ EACH ITEM BELOW (a-f) AND CODE YES OR NO FOR EACH. IF 'NO' GO TO NEXT LETTER**<br>¼,&,Q½ rd i<sa vkSj ÁR;sd ds fy, gka ;k ugha dk dksM djsaA ;fn ugha gS vxys v{kj ij tk;saA			
	Tests<br>जाँचें	YES हाँ	NO नहीं	DO NOT KNOW<br>पता नहीं
a.	Were you weighed? क्या आपका वजन लिया गया था?	1	0	99
b.	Was your blood pressure measured? क्या आपके रक्तचाप (ब्लड प्रैशर) की जाँच की गई थी?	1	0	99
c.	Did you give a urine sample? क्या आपने पिशाप का नमूना दिया था?	1	0	99

d.	Was your blood checked for hemoglobin level? हीमोग्लोबिन का स्तर जानने के लिये क्या आपकी खून कि जाँच की गयी थी?	1	0	99
e.	Was your abdomen checked? क्या आपके पेट की जाँच की गई थी?	1	0	99
f.	Was your ultrasound done? क्या आपका अल्ट्रासाउंड किया गया था?	1	0	99
**R6a.**	During your pregnancy, were you identified as anaemic?<br>**जब आप गर्भवती थी तब क्या आपको बताया गया था के आपको खून की कमी है?**	1 = YES हाँ<br>0 = NO नहीं<br>99 = DO NOT KNOW पता नहीं		
R6.	During your pregnancy, were you given a TT injection?<br>जब आप गर्भवती थी तो क्या आपको ट्टिनस की सुई लगी थी?	1 = YES हाँ<br>0 = NO नहीं → **GO TO R8**<br>99 = DO NOT KNOW पता नहीं → **GO TO R8**		
R7.	During your pregnancy, how many times did you receive a tetanus injection?<br>जब आप गर्भवती थी तब आपको कितने ट्टिनस की सुई लगी थी?	☐☐ NO. OF TIMES कितनी बार<br>99 = DO NOT KNOW/REMEMBER पता नहीं / याद नहीं		
R8.	During your pregnancy did you receive any Iron Folic Acid (IFA) tablets?<br>जब आप गर्भवती थी तब क्या आपको आई.एफ.ए. (ताकत की लाल गोली) की गोलियाँ मिली थी?<br><br>**SHOW SAMPLES OF IFA TABLETS**<br>vkb-,Q-,- dh xkfy;k d ueu fn[kk;A	1 = YES हाँ<br>0 = NO नहीं → **GO TO R12**<br>99 = DO NOT KNOW पता नहीं → **GO TO R12**		
R9.	In which month of pregnancy did you receive IFA tablets for the first time?<br>गर्भावस्था के किस महीने में आपको पहली बार आई.एफ.ए. (ताकत की लाल गोली) की गोलियाँ मिली थी?	☐ COMPLETED MONTHS पूर्ण महीने<br>99 = DO NOT KNOW पता नहीं		
R10.	How many tablets did you receive in total during your whole pregnancy?<br>जब आप गर्भवती थी तब आपको आई.एफ.ए. (ताकत की लाल गोली) की कितनी गोलियाँ मिली थी?<br><br>**PROBE FOR WHETHER TABLETS ARE ADULT (LARGE) OR PEDIATRIC (SMALL) DOSE.**<br>iN fd ;g xkfy;k cMh Fkh ;k NkVh FkhA	☐☐☐ NUMBER OF LARGE TABLETS बड़ी गोलियों की संख्या<br>☐☐☐ NUMBER OF SMALL TABLETSछोटी गोलियों की संख्या<br>999 = DO NOT REMEMBER याद नहीं		
R11.	During the whole pregnancy with [CHILD NAME], how many tablets did you consume?<br>जब (बच्चे का नाम) आपके गर्भ में था तब आपने आई.एफ.ए. (ताकत की लाल गोली) की कितनी गोलियाँ खायी थी?	☐☐☐ NUMBER OF LARGE TABLETS बड़ी गोलियों की संख्या<br>☐☐☐ NUMBER OF SMALL TABLETSछोटी गोलियों की संख्या<br>999 = DO NOT REMEMBER याद नहीं		
R12.	During your pregnancy with [CHILD NAME], were you given or did you buy any iron folic syrup?<br>जब (बच्चे का नाम) आपके गर्भ में था, क्या आपको आईरन फोलिक एसिड (ताकत) की सिरप मिली थी या आपने खरीदी थी?	1 = YES हाँ<br>= NO नहीं → **GO TO R13**<br>0		
**R12a**	In which month of pregnancy did you receive IFA syrup bottles for the first time?<br>गर्भावस्था के कौनसे महीने में आपको पहली बार IFA सिरप की बोतले मिली थी?	☐ COMPLETED MONTHS पूर्ण महीने<br>DO NOT KNOW पता नहीं		
**R12b**	How many IFA syrup bottles did you receive in total during your whole pregnancy?<br><br>जब बच्चे आपके गर्भ में था तब आपने आई.एफ.ए. की सिरप की कितनी बोतलें मिली थी?	☐☐.☐ NUMBER OF BOTTLES<br>DO NOT REMEMBER		
**R12c**	During the whole pregnancy with [CHILD NAME], how many bottles did you consume?<br><br>जब बच्चे आपके गर्भ में था तब आपने आई.एफ.ए. की सिरप की कितनी बोतलें खायी थी?	☐☐. . ☐ NUMBER OF BOTTLES<br>= DO NOT REMEMBER		
R13.	**ASK IF r11>0 or r12c>0**<br>During the whole pregnancy, for how many days did you take the tablets or syrup?<br>जब आप गर्भवती थी पूरी गर्भावस्थ के दौरान आपने कितने दिनों तक आईरन फोलिक एसिड (ताकत) की गोली या सिरप खायी थी?<br><br>**IF ANSWER IS NOT NUMERIC, PROBE FOR APPROXIMATE NUMBER OF DAYS.**	☐☐☐ NO OF DAYS FOR LARGE TABLETS बड़ी गोलियाँ खाने के दिनों की संख्या<br>☐☐☐ NO OF DAYS FOR SMALL TABLETS छोटी गोलियाँ दिनों की संख्या<br>☐☐☐ NO OF DAYS FOR SYRUP सिरप खाने के दिनों की संख्या<br>999 DO NOT REMEMBER याद नहीं		

	;fn mÙkj l[;k e ugh g] iN fd fnuk dh l[;k yxHkx D;k FkhA	
**R13a 1**	During your pregnancy, were you given or did you buy any calcium tablets?<br><br>जब आप गर्भवती थी क्या आपको केलसियम की गोली मिली थी या आपने खरीदी थी?	1 = YES हाँ<br>0 = NO नहीं → **GO TO R14**
**R13a 2**	During your pregnancy, how many calcium tablets were you given or did you buy?<br><br>जब आप गर्भवती थी आपको केलसियम की कितनी गोली मिली थी या आपने खरीदी थी?	□□□ NUMBER OF TABLETS गोलियों की संख्या<br>999 DO NOT REMEMBER याद नहीं
**R13a 3**	During the whole pregnancy, how many tablets did you consume?<br><br>जब आप गर्भवती थी तब आपने केलसियम की कितनी गोलियाँ खायी थी?	□□□ NUMBER OF TABLETS गोलियों की संख्या<br>999 = DO NOT REMEMBER याद नहीं
**R13a 4**	During the whole pregnancy, for how many days did you take the tablet?<br>जब आप गर्भवती थी पूरी गर्भावस्थ के दौरान आपने कितने दिनों तक गोली खायी थी?<br><br>**IF ANSWER IS NOT NUMERIC, PROBE FOR APPROXIMATE NUMBER OF DAYS.**<br>;fn mÙkj l[;k e ugh g] iN fd fnuk dh l[;k yxHkx D;k FkhA	□□□ NO OF DAYS<br>999=DO NOT REMEMBER याद नहीं
**R13a 5**	During your pregnancy with [CHILD NAME], did you take rest more frequently than when you were not pregnant?<br>जब (बच्चे का नाम) आपके गर्भ में था तब क्या आप गर्भवती न होने के समय की तुलना में अधिक बार आराम करती थी?	1 = YES हाँ<br>0 = NO नहीं
**R13a 6**	During your pregnancy with [CHILD NAME] did you consume more food as compared to when you were not pregnant?<br>जब (बच्चे का नाम) आपके गर्भ में था तब क्या आप गर्भवती न होने के समय की तुलना में अधिक भोजन करती थी?	1 = YES हाँ<br>= NO नहीं
**R13a 7**	Which of the following foods did you consume almost daily during pregnancy?<br><br>कौनसे पढके सुनाई जाने वाली चीज़ें आपने रोजाना गर्भवस्था के दौरान खाया?<br><br>MULTIPLE CHOICE	1= Dark green leafy vegetables हरि सब्जियां<br>2=Yellow or orange fruits or vegetables (e.g. Pumpkin, carrots, ripe mango, ripe papaya) पीली या नारंगी फल और सब्जी<br>3=Lentils, dal दल<br>4=Roti, bread or rice रोटी, चावल, ब्रेड<br>5=Eggs अन्दा<br>6=Meat, chicken or fish मॉस<br>7=Milk, paneer or yogurt दूध, पनीर, दही<br>88=Othersअन्य
**R13a 8**	How many kgs. did you gain during pregnancy?<br>गर्भवस्था के दौरान कितना वजन बढ़ा?	__ KGS<br>99 = DO NOT KNOW पता नहीं
R14.	Are you aware about the different danger signs during pregnancy which require visit to a health facility or contacting a healthcare provider?<br><br>क्या आप गर्भावस्था के दौरान होने वाले खतरे के विभिन्न लक्षणों के बारे में जानती हैं जिनके लिए स्वास्थ्य केंद्र जाने की या स्वास्थ्य प्रदाता से संपर्क करने की जरूरत होती है?	1 = YES हाँ<br>0 = NO नहीं → **GO TO R17**
R15.	What are the different danger signs during pregnancy which require visit to a health facility or contacting a healthcare provider?<br>गर्भावस्था के दौरान खतरे के वह कौन से लक्षण हैं जिनके लिए स्वास्थ्य केंद्र जाने की या स्वास्थ्य प्रदाता से संपर्क करने की जरूरत होती है?<br><br>**PROBE : ANY OTHERS?**<br>çkc % dN vkj\<br><br>**RECORD ALL MENTIONED**<br>lHkh mÙkj fy[kA	1 = EXCESSIVE VAGINAL BLEEDING योनि से अत्यधिक खून निकलना<br>2 = DIFFICULTY IN BREATHING सांस लेन में कठिनाई<br>3 = SEVERE HEADACHE तेज सिर दर्द<br>4 = BLURRED VISION धुंधला दिखना<br>5 = SWELLING OF THE HANDS, FEET, BODY OR FACE हाथों, पैरों, शरीर और चेहरे का सूजन<br>6 = HIGH FEVER तेज बुखार<br>7 = LOSS OF CONSCIOUSNESS होश खोना<br>8 = SEVERE ABDOMINAL PAIN पेट में तेज दर्द<br>9 = CONVULSIONS दौरे पड़ना<br>10 = HIGH BLOOD PRESSURE उच्च रक्तचाप

		88 = OTHERS (SPECIFY) अन्य (स्पष्ट करें)		
R16.	From where did you get the information about these danger signs during pregnancy?<br>गर्भावस्था के दौरान खतरे के लक्षणों के बारे में आपको कहाँ से जानकारी मिली थी?<br><br>**PROBE : ANY OTHERS?**<br>çkc % dN vkj\<br><br>**RECORD ALL MENTIONED**<br>lHkh mÙkj fy[kA	1 = GOVERNMENT DOCTOR सरकारी डाक्टर<br>2 = PRIVATE DOCTOR प्राइवेट डाक्टर<br>3 = STAFF NURSE स्टाफ नर्स<br>4 = LHV लेडी हैल्थ विजिटर (एल.एच.वी.)<br>5 = MALE HEALTH WORKER पुरुष स्वास्थ्य कर्मी<br>6 = ANM ए.एन.एम.<br>7 = OTHER HEALTH PERSONNEL अन्य स्वास्थ्य कर्मी<br>8 = ASHA आशा<br>9 = AWW आँगनवाड़ी कार्यकर्ता<br>10 = SBA/TRAINED DAI एसबीए/प्रशिक्षित दाई<br>11 = DAI दाई<br>12 = RMP आर.एम.पी. (झोला छाप डाक्टर)<br>13 = FAMILY MEMBER परिवार के सदस्य<br>14 = FRIENDS/NEIGHBOUR दोस्त/पड़ौसी<br>15 = RADIO/TV/NEWS PAPER रेडियो/टेलिविजन/अखबार<br>16 = MOBILE SMS मोबाइल एसएमएस<br>88 = OTHERS (SPECIFY) अन्य (स्पष्ट करें)<br>98 = NO ONE कोई नहीं<br>99 = DO NOT KNOW मालूम नहीं		
R17.	During your pregnancy with [CHILD NAME] were you told to visit a doctor/facility in case of any pregnancy complications?<br>जब (बच्चे का नाम) आपके गर्भ मे था तब क्या आपको यह बताया गया था कि गर्भावस्था से संबंधित किसी भी परेशानी के मामले में डाक्टर के पास/स्वास्थ्य केंद्र पर जायें?	1 = YES हाँ<br>0 = NO नहीं		
R18.	During your pregnancy what kind of problems did you experience?<br>जब आप गर्भवती थी तब आपको किस प्रकार की परेशानी हुई थी?<br><br>**DO NOT READ LIST. CODE ALL MENTIONS IN FIRST COLUMN. THEN READ EACH ITEM BELOW (a-m) THAT WASN'T MENTIONED AND CODE RESPONSE IN SECOND COLUMN. IF NOT MENTIONED, SKIP TO NEXT LETTER.**<br>lph dk u i<A igy dkye e lHkh mYy[k fd; x; mÙkjk dk dkM djA bld ckn mu fcUnvk ¼,&t½ dk i< ftudk mYy[k ugh fd;k x;k Fkk vkj nlj dkye e mÙkj dk dkM djA ;fn ugh crk;k x;k vxy fcn ij tk;A	1 = YES, SPONTANEOUS<br>हाँ, स्वयं बताया	2 = YES, AFTER READING<br>हाँ, पढ़ने के बाद	0 = NO<br>नहीं
a.	Excessive vaginal bleeding योनी से अत्यधिक रक्तस्राव	1	2	0
b.	Difficulty in breathing साँस लेन में कठिनाई	1	2	0
c.	Severe headache तेज सिर दर्द	1	2	0
d.	Blurred vision धुंधला दिखना	1	2	0
e.	Swelling of the hands, feet, body or face हाथों, पैरों, शरीर और चेहरे पर सूजन	1	2	0
f.	High fever तेज बुखार	1	2	0
g.	Loss of consciousness चेतना (होश) खोना	1	2	0
h.	Severe abdominal pain पेट में तेज दर्द	1	2	0
i.	Convulsions दौरे पड़ना	1	2	0
j.	High Blood Pressure उच्च रक्तचाप	1	2	0
k.	Heart disease दिल की बीमारी	1	2	0
l.	Diabetes मधुमेय	1	2	0
m.	Other medically diagnosed disease कोई बीमारी जो चिकित्सक द्वारा बतायी गयी है	1	2	0
R19.	During the last 3 months of pregnancy, did you suffer from any of the following illnesses?<br>गर्भावस्ता के अखरी 3 महीनों में क्या आपको निम्न में से कोई बीमारी हुई थी?<br>**DO NOT READ LIST. CODE ALL MENTIONS IN FIRST COLUMN. THEN READ EACH ITEM BELOW (a-j) THAT WASN'T MENTIONED AND CODE RESPONSE IN SECOND COLUMN. IF NOT MENTIONED, SKIP TO NEXT LETTER.**	1 = YES, SPONTANEOUS<br>हाँ, स्वयं बताया	2 = YES, AFTER READING<br>हाँ, पढ़ने के बाद	0 = NO<br>नहीं

	सूची को न पढ़ें। पहले कालम में सभी उल्लेख किये गये उत्तरों को कोड करें। इसके बाद उन बिन्दुओं (ए–जे) को पढ़ें जिनका उल्लेख नहीं किया गया था और दूसरे कालम में उत्तर को कोड करें। यदि नहीं बताया गया अगले बिंदु पर जायें।			
a.	Vaginal bleeding योनी से रक्तस्राव	1	2	0
b.	Smelly vaginal discharge योनि से बदबूदार पानी निकलना	1	2	0
c.	Puffy face चेहरे का सुजन	1	2	0
d.	Headache सिर दर्द	1	2	0
e.	Blurred vision धुंधला दिखना	1	2	0
f.	Convulsions दौरे पड़ना	1	2	0
g.	Febrile illness बुखार जिसका कारण पता नहीं	1	2	0
h.	Severe abdominal pain that is not labor pain पेट में तेज दर्द जो प्रसव पीड़ा नहीं थी	1	2	0
i.	Pallor and difficulty in breathing साँस लेन में कठिनाई के साथ चेहरे का फीकापन	1	2	0
j.	Any other illness कोई और बीमारी	1	2	0
R20.	**ASK ONLY IF ANY OF THE RESPONSES IN r19(a-j) IS CODED 1 OR 2, ELSE GO TO r22**<br><br>Did you seek treatment for this problem?<br>क्या आपने इस समस्या का इलाज कराया था?	1 = YES हाँ<br>0 = NO नहीं → **GO TO R22**		
R21.	From where did you seek treatment?<br>आपने इलाज कहाँ से कराया था?<br><br>**PROBE : ANY OTHERS?**<br>çkc s dN vkj\<br><br>**RECORD ALL MENTIONED**<br>lHkh mÙkj fy[kA	1 = GOVERNMENT/MUNICIPAL HOSPITAL सरकारी/नगरपालिका का अस्पताल<br>2 = GOVERNMENT DISPENSARY सरकारी दवाखाना<br>3 = UHC/UHP/UFWC यूएचसी/यूएचपी/यूएफडब्ल्यूसी<br>4 = CHC सीएचसी<br>5 = APHC/NPHC/BPHC एपीएचसी/एनपीएचसी/बीपीएचसी<br>6 = SUB-CENTER उपकेन्द्र<br>7 = ANGANWADI CENTER आँगनवाड़ी केन्द्र<br>8 = VHND ग्राम स्वास्थ्य एवं पोषण दिवस<br>9 = NGO HOSPITAL/CLINIC गैर सरकारी संस्था का अस्पताल/क्लीनिक<br>10 = PRIVATE HOSPITAL/CLINIC प्राइवेट अस्पताल/क्लीनिक<br>11 = MEDICINE SHOP दवा की दुकान<br>12 = FOLK HEALER ओझा/वैद्य/झाड़फूँक<br>13 = HOME REMEDIES घरेलू उपचार<br>14 = RMP आर.एम.पी. (झोला छाप डाक्टर)<br>88 = OTHERS (SPECIFY) अन्य (स्पष्ट करें)		
R22.	Did the ASHA come to meet you at your home during your pregnancy?<br>जब आप गर्भवती थी तब क्या आशा आपके घर आपसे मिलने आई थी?	1 = YES हाँ<br>0 = NO नहीं → **GO TO R24**		
R23.	How many times did the ASHA come to meet you at your home?<br>जब आप गर्भवती थी तब आशा आपके घर आपसे मिलने कितनी बार आई थी?	☐☐ NO. OF TIMES इतनी बार<br>99 DO NOT REMEMBER याद नहीं		
**R23A**	Did the ASHA come to meet you at your home during the last three months of your pregnancy with [CHILD NAME]?<br>जब (बच्चे का नाम) आपके गर्भ में था, तब क्या गर्भावस्था के आखरी तीन महिनों में आशा आपके घर आपसे मिलने आई थी?	1 = YES हाँ<br>0 = NO नहीं → **GO TO R23C**		
**R23B**	How many times did the ASHA come to meet you at your home during the last three months of your pregnancy with [CHILD NAME]?<br>जब (बच्चे का नाम) आपके गर्भ में था, तब गर्भावस्था के आखरी तीन महिनों में आशा आपके घर आपसे मिलने कितनी बार आई थी?	☐☐ NO. OF TIMES इतनी बार<br>99 DO NOT REMEMBER याद नहीं		
R24.	Did the AWW come to meet you at your home during your pregnancy?<br>जब आप गर्भवती थी तब क्या आँगनवाड़ी कार्यकर्ता आपके घर आपसे मिलने आई थी?	1 = YES हाँ<br>0 = NO नहीं → **GO TO** R26		
R25.	How many times did the AWW come to meet you at your home?<br>जब आप गर्भवती थी तब आँगनवाड़ी कार्यकर्ता आपके घर आपसे मिलने कितनी बार आई थी?	☐☐ NO. OF TIMES इतनी बार<br>99 DO NOT REMEMBER याद नहीं		

**R25b**	Did the AWW come to meet you at your home during the last three months of your pregnancy?<br>जब आपके गर्भ में था, तब क्या गर्भावस्था के आखरी तीन महिनों में आँगनवाड़ी कार्यकर्ता आपके घर आपसे मिलने आई थी?	1 = YES हाँ<br>0 = NO नहीं → **GO TO** R26
**R25c**	How many times did the AWW come to meet you at your home during the last three months of your pregnancy?<br>जब बच्च आपके गर्भ में था, तब गर्भावस्था के आखरी तीन महिनों में आँगनवाड़ी कार्यकर्ता आपके घर आपसे मिलने कितनी बार आई थी?	☐☐ NO. OF TIMES इतनी बार<br>99 DO NOT REMEMBER याद नहीं
R26.	Did the ANM come to meet you at your home during your pregnancy?<br>जब आप गर्भवती थी तब क्या ए.एन.एम. आपके घर आपसे मिलने आई थी?	1 = YES हाँ<br>0 = NO नहीं → **GO TO R2**7c
R27.	How many times did the ANM come to meet you at your home?<br>जब आप गर्भवती थी तब ए.एन.एम. आपके घर आपसे मिलने कितनी बार आई थी?	☐☐ NO. OF TIMES इतनी बार<br>99 DO NOT REMEMBER याद नहीं
**R27a**	Did the ANM come to meet you at your home during the last three months of your pregnancy?<br>जब आपके गर्भ में था, तब क्या गर्भावस्था के आखरी तीन महिनों में ए.एन.एम. आपके घर आपसे मिलने आई थी?	1 = YES हाँ<br>0 = NO नहीं → **GO TO R27C**
**R27b**	How many times did the ANM come to meet you at your home during the last three months of your pregnancy?<br>जब (बच्चे का नाम) आपके गर्भ में था, तब गर्भावस्था के आखरी तीन महिनों में ए.एन. एम. आपके घर आपसे मिलने कितनी बार आई थी?	☐☐ NO. OF TIMES इतनी बार<br>99 = DO NOT REMEMBER याद नहीं

R27c	**THINKING ABOUT THE LAST VISIT**<br>Which frontline worker had visited you the last time at home?<br>पिछली बार जब आशा/अन्गंवादी कार्यकर्ता/ऐ एन एम् में से आपसे मिलने कौन आई थी?	1=ASHA<br>आशा<br>2=AWW<br>अन्गंवादी कार्यकर्ता<br>3=ANM<br>ऐ एन एम्<br>9=NONE
R27d	How much time the frontline worker who visited you last spend during the session (in minutes)?<br>पिछली बार आपके घर जो कार्यकर्ता आई थी उसने कितना समय बिताया था ? (मिनट में)	☐☐<br>MINUTES मिनट
R27e	Did she advise/counsel you on the following topics?<br>क्या उसने पिछली बार आपको निम्न लिखित चीज़ों के बारें में सलाह दी थी?	1= MATERNAL NUTRITION मात्री पोषण<br>2= IFA CONSUMPTION IFA की सिरप या गोलियां लेना<br>3= DIETARY DIVERSITY FOR NUTRITION आहार बिबीधता ताकि महिला को ज़रुरत का पोषण मिले<br>4= TAKING CARE OF YOUR HEALTH DURING PREGNANCY गर्भावस्था के दौरान अपने स्वास्थ्य की देखभाल करना<br>5= GOING TO THE HOSPITAL FOR A CHECK-UP DURING PREGNANCY गर्भावस्था के दौरान जांच के लिए अस्पताल जाना<br>6= GOING TO A HEALTH FACILITY FOR DELIVERY प्रसव कराने के लिए स्वास्थ्य केंद्र या अस्पताल जाना<br>7= PLANNING IN ADVANCEFOR DELIVERY प्रसव के लिए पहले से तैयारी किस प्रकार करनी है<br>8= HOW TO TAKE CARE OF THE NEWBORN नवजाति शिशु की देखरेख कैसे करनी है<br>9=EXCLUSIVE BREASTFEEDING केवल स्तनपान<br>10= COMPLEMENTARY FEEDING पूरक आहार/ उपरी आहार<br>11= IMMUNIZATION टीकाकरण<br>12= FAMILY PLANNING परिवार नियोजन<br>13= HANDWASHING AND SANITATION हाथ धोना और साफसफाई<br>88= OTHERS (SPECIFY) अन्य (स्पष्ट करें)

R27f	Was she clear in explaining the topics?<br>क्या वह विषयों को स्पष्ट रूप से समझा पायी थी?	1 = YES हाँ<br>0 = NO नहीं
R27g	Were you able to understand everything?<br>क्या आप सब कुछ समझ पायी थी?	1 = YES हाँ<br>0 = NO नहीं
R27h	Was she able to answer all your queries clearly?<br>क्या वह आपके सभी सवालों का स्पष्ट रूप से उत्तर दे पायी थी?	1 = YES हाँ<br>0 = NO नहीं
R27i	What job aid tools did she use during this most recent visit?<br>आशा/आंगनवाडी कार्यकर्ता ने सबसे हाल के इस दौरे के समय किस साधन या उपकरण का उपयोग किया?<br>**CODE ALL THAT APPLY**<br>lHkh ykxw gksus okys dksM djs	1= PLASTIC PICTURE CARDS (describe mobile kunji cards) प्लास्टिक के पिक्चर कार्ड (मोबाइल कुंजी)<br>2= PAMPHLET पम्फलेट<br>3=KATORA/SPOON सी कटोरा/चम्मच<br>4= COPPER-T ई कॉपर–टी<br>5= MALA-D एफ माला–डी<br>88=OTHERS (SPECIFY) अन्य (बताएं)
**R27k**	**<u>If R27i=1 OR R27j=1, ASK ,ELSE GO TO R28.</u>**<br><br>Did you find the usage of these tools useful?<br>क्या आपने इन उपकरणों को उपयोगी पाया?	YES हॉ..1<br>NO नहीं...............0 **GO TO R28→**
**R27l**	Which of these tools helped you to understand the issues explained by FLW?<br>इनमें से कौन कौन से उपकरणों से आपको अग्रणी कतार की कार्यकर्ता (एफएलडब्ल्यू) द्वारा स्पष्ट किये गये मुद्दों को समझने में मदद मिली?<br><br>**CODE ALL THAT APPLY**<br>lHkh ykxw gksus okys dksM djs	PLASTIC PICTURE CARDS प्लास्टिक के पिक्चर कार्ड......1<br>PAMPHLET पम्फलेट ....2<br>KATORA कटोरा ....3<br>COPPER-T कॉपर–टी...4<br>MALA-D माला डी...5<br>OTHER अन्य...........................88

R28.	Did the LHV/Government Doctor come to meet you at your home during your pregnancy?<br>जब आप गर्भवती थी तब क्या एल.एच.वी./सरकारी डाक्टर आपके घर आपसे मिलने आये थे?	1 = YES हाँ<br>0 = NO नहीं → **GO TO** Error! Reference source not found.
R29.	How many times did the LHV/Government Doctor come to meet you at your home?<br>जब आप गर्भवती थी तब एल.एच.वी./सरकारी डाक्टर आपके घर आपसे मिलने कितनी बार आये थे?	☐☐ NO. OF TIMES इतनी बार<br>99 = DO NOT REMEMBER याद नहीं
**R29A.**	After the delivery, how much iron folic acid tablets and syrups bottles did you receive or purchase?<br><br>प्रसव के बाद आपको कितने IFA की गोली और सिरप की बोतलें मिली थी?	Large Tablets ☐☐<br>बड़ी गोली<br>Small Tablets ☐☐<br>छोटी गोली<br>Bottles ☐☐ . ☐☐<br>बोतल<br>**IF 00, THEN SKIP TO R28C.**
**R28B.**	How many IFA tablets/syrup bottles did you consume after the delivery?<br><br>प्रसव के बाद आपको कितने IFA की गोली और सिरप की बोतलें खायी थी?	Large Tablets ☐☐<br>बड़ी गोली<br>Small Tablets ☐☐<br>छोटी गोली<br><br>Bottles ☐☐ . ☐☐<br>बोतल
**R28C.**	After the delivery, how many calcium tablets did you receive or purchase?<br><br>प्रसव के बाद आपको कितने कैल्शियम की गोली मिली थी?	टेबलेट'<br><br>Tablets ☐☐

**R28D.**	How many calcium tablet did you consume after the delivery?<br><br>सव के बाद आपको कितने कैल्शियम की गोली खायी थी?	टेबलेट'<br><br>Tablets ☐☐
**R28E.**	**ASK IF R28B >0 OR R28D>0**<br>After the delivery, for how many days did you take the tablets or syrup?<br>प्रसव के बाद आपने कितने दिनों तक आईरन फोलिक एसिड (ताकत) की गोली या सिरप खायी थी?<br><br>**IF ANSWER IS NOT NUMERIC, PROBE FOR APPROXIMATE NUMBER OF DAYS.**<br>;fn mÙkj l[;k e ugh g] iN fd fnuk dh l[;k yxHkx D;k FkhA	☐☐☐ NO OF DAYS FOR LARGE TABLETS बड़ी गोलियाँ खाने के दिनों की संख्या<br>☐☐☐ NO OF DAYS FOR SMALL TABLETS छोटी गोलियों दिनों की संख्या<br>☐☐☐ NO OF DAYS FOR SYRUP सिरप खाने के दिनों की संख्या<br>☐☐☐ NO OF DAYS FOR CALCIUM TABLETS कैल्शियम की गोली खाने के दिनों की संख्या<br><br>DO NOT REMEMBER याद नहीं

R30.	Where did the delivery take place?<br>प्रसव कहाँ हुआ था?<br><br>**IF AT A FACILITY, PROBE: WHAT TYPE OF FACILITY WAS THIS? IF UNABLE TO DETERMINE IF A HOSPITAL, HEALTH CENTRE, OR CLINIC IS PUBLIC OR PRIVATE MEDICAL SECTOR, WRITE THE NAME OF THE PLACE.**<br>;fn LokLF; lfo/kk e rc çkc dj% LokLF; lfo/kk fdl çdkj dh Fkh\ ;fn ;g fu/kkfjr dju e leL;k gk jgh g fd LokLF; lfo/kk] vLirky Fkk LokLF; dUæ lkoZtfud Fkk ;k futh {k= dk] LFkku dk uke fy[kA<br><br>____________________________<br>NAME OF PLACE स्थान का नाम<br><br>**CODE ONLY ONE**<br>dksy ,d mÙkj	1 = HOME घर पर<br>2 = GOVERNMENT/MUNICIPAL HOSPITAL सरकारी/नगरपालिका का अस्पताल<br>3 = GOVERNMENT DISPENSARY सरकारी दवाखाना<br>4 = UHC/UHP/UFWC यूएचसी/यूएचपी/यूएफडब्ल्यूसी<br>5 = CHC सीएचसी<br>6 = APHC/NPHC/BPHC एपीएचसी/एनपीएचसी/बीपीएचसी<br>7 = SUB-CENTER उपकेन्द्र<br>8 = NGO HOSPITAL/CLINIC गैर सरकारी संस्था का अस्पताल/क्लीनिक<br>9 = PRIVATE HOSPITAL/CLINIC प्राइवेट अस्पताल/क्लीनिक<br>88 = OTHERS (SPECIFY) अन्य (स्पष्ट करें)
R31.	Who conducted the delivery?<br>प्रसव किसने किया था?<br><br>**CODE ONLY ONE**<br>dksy ,d mÙkj	1 = GOVERNMENT DOCTOR सरकारी डाक्टर<br>2 = PRIVATE DOCTOR प्राइवेट डाक्टर<br>3 = STAFF NURSE स्टाफ नर्स<br>4 = LHV लेडी हैल्थ विजिटर (एल.एच.वी.)<br>5 = MALE HEALTH WORKER पुरुष स्वास्थ्य कर्मी<br>6 = ANM ए.एन.एम.<br>7 = OTHER HEALTH PERSONNEL अन्य स्वास्थ्य कर्मी<br>8 = ASHA आशा<br>9 = AWW आँगनवाड़ी कार्यकर्ता<br>10 = SBA/TRAINED DAI एसबीए/प्रशिक्षित दाई<br>11 = DAI दाई<br>12 = RMP आर.एम.पी. (झोला छाप डाक्टर)<br>13 = FAMILY MEMBER परिवार के सदस्य<br>14 = FRIENDS/NEIGHBOUR मित्र/पड़ौसी<br>88 = OTHERS (SPECIFY) अन्य (स्पष्ट करें)<br>98 = NO ONE कोई नहीं<br>99 = DO NOT KNOW पता नहीं
R32.	Who assisted the delivery?<br>प्रसव करने मे किस किस ने सहायता की थी?<br><br>**PROBE FOR THE TYPE OF PERSON AND RECORD ALL PERSONS ASSISTING. ANY OTHERS? RECORD ALL MENTIONED**<br>çkc % ml 0;fDr dk Ádkj D;k Fkk ftlu vkidh lgk;rk dh Fkh vkj lHkh 0;fDr;k dk nt djA D;k dkbl vkj Fkk\ lHkh mÙkj fy[kA<br><br>**IF RESPONDENT SAYS NO ONE ASSISTED, PROBE TO DETERMINE WHETHER ANY ADULTS WERE PRESENT AT THE DELIVERY.**	1 = GOVERNMENT DOCTOR सरकारी डाक्टर<br>2 = PRIVATE DOCTOR प्राइवेट डाक्टर<br>3 = STAFF NURSE स्टाफ नर्स<br>4 = LHV लेडी हैल्थ विजिटर (एल.एच.वी.)<br>5 = MALE HEALTH WORKER पुरुष स्वास्थ्य कर्मी<br>6 = ANM ए.एन.एम.<br>7 = OTHER HEALTH PERSONNEL अन्य स्वास्थ्य कर्मी<br>8 = ASHA आशा<br>9 = AWW आँगनवाड़ी कार्यकर्ता<br>10 = SBA/TRAINED DAI एसबीए/प्रशिक्षित दाई<br>11 = DAI दाई<br>12 = RMP आर.एम.पी. (झोला छाप डाक्टर)

	यदि उत्तरदाता यह कहती है कि किसी ने सहायता नहीं की, यह निर्धारित करने के लिए पूछें कि क्या कोई वयस्क व्यक्ति प्रसव के समय उपस्थित था।	13 = FAMILY MEMBER परिवार के सदस्य<br>14 = FRIENDS/NEIGHBOUR मित्र/पड़ौसी<br>88 = OTHERS (SPECIFY) अन्य (स्पष्ट करें)<br>98 = NO ONE कोई नहीं<br>99 DO NOT KNOW पता नहीं
**R32a**	**ASK IF R30>1**<br><br>How long did you stay at the healthcare facility?<br>आप स्वास्थ्य सुविधा में कब तक रूकी थी?<br>**CODE HOURS, DAYS, OR WEEKS.**<br>घंटे, दिन और सप्ताह में कोड करें।	☐ ☐☐<br>1 = HOURS घंटे<br>2 = DAYS दिन<br>3 = WEEKS सप्ताह
R32b	Was this earlier than the nurse/doctor recommended?<br>क्या यह नर्स/डॉक्टर की सलाह की गए समय से जल्दी थी?	1 = YES<br>0 = NO<br>99 = DID NOT RECOMMEND
R32c	What mode of transportation did you take to get to travel from healthcare facility to home?<br>आप स्वस्थ्य केंद्र से घर कैसे गए?<br>**CODE ALL THAT APPLY**	1= AMBULANCE<br>2 TAXI/JEEP/TRACTOR<br>3=MOTOR CYCLE<br>4=CAR/TRUCK<br>5= BUS<br>6= BULLOCK/ANIMAL-DRAWN CART<br>7= ON FOOT<br>8=BICYCLE<br>9= AUTORICKSHAW/TEMPO<br>88= OTHER (SPECIFY)
R32d	How long did it take you to reach the home from healthcare facility?<br>आपको स्वस्थ्य केंद्र से घर पहुचने में कितना समय लगा?	☐ ☐☐<br>1 = HOURS घंटे<br>2 = DAYS दिन
R33.	Do you know of any danger signs or symptoms that a woman might have during delivery or within the first month after delivery that would require seeking medical care?<br>क्या आप खतरे के ऐसे लक्षणों या संकेतों के बारे मे जानती हैं जिनका अनुभव महिला प्रसव के दौरान या प्रसव के बाद एक महीने के अन्दर कर सकती है और जिसके लिए चिकित्सा देखरेख की जरूरत होती है?	1 = YES हॉं<br>R0 = NO नहीं → **GO TO R37**
R34.	Could you name some of the danger signs or symptoms that a woman might have during delivery or within the first month after delivery that would require seeking medical care?<br>क्या आप खतरे के ऐसे लक्षणों या संकेतों के बारे मे बता सकती हैं जिनका अनुभव महिला प्रसव के दौरान या प्रसव के बाद एक महीने के अन्दर कर सकती है और जिसके लिए चिकित्सा देखरेख की जरूरत होती है?<br><br>**PROBE : ANY OTHERS?**<br>çkc ¼ dN vkj\<br><br>**RECORD ALL MENTIONED**<br>lHkh mÙkj fy[kA	1 = PROLONGED LABOUR FOR MORE THAN 12 HOURS लंबे समय तक, 12 घंटे से अधिक समय तक प्रसव पीड़ा<br>2 = RETAINED PLACENTA गर्भनाल का अंदर रहना<br>3 = OBSTRUCTED LABOUR (MALPOSITION/PRESENTATION) रुकावट के साथ प्रसव (शिशु का गलत स्थिति में होना)<br>4 = SEVERE BLEEDING अत्यधिक खून निकलना<br>5 = DIFFICULTY BREATHING सांस लेने में कठिनाई<br>6 = SEVERE PAIN IN ABDOMEN पेट में तेज दर्द<br>7 = SEVERE WEAKNESS अत्यधिक कमजोरी<br>8 = CONVULSIONS दौरे पड़ना<br>9 = FOUL SMELLING VAGINAL DISCHARGE योनि से बदबूदार पानी निकलना<br>10 = FEVER बुखार<br>11 = SEVERE HEADACHE तेज सिर दर्द<br>12 = LOSS OF CONSCIOUSNESS होश खोना<br>13 = DOUBLE VISION एक के दो दिखना<br>88 = OTHERS (SPECIFY) अन्य (स्पष्ट करें)
R35.	From where did you get the information about these danger signs that a woman can have during delivery or post-delivery?<br>प्रसव के दौरान या प्रसव के बाद महिला को खतरे के इन संकेतों के बारे में आपको कहाँ से जानकारी मिली थी?	1 = GOVERNMENT DOCTOR सरकारी डाक्टर<br>2 = PRIVATE DOCTOR प्राइवेट डाक्टर<br>3 = STAFF NURSE स्टाफ नर्स<br>4 = LHV लेडी हैल्थ विजिटर (एल.एच.वी.)<br>5 = MALE HEALTH WORKER पुरुष स्वास्थ्य कर्मी

	**PROBE : ANY OTHERS?**<br>çkc % dN vkj\<br><br>**RECORD ALL MENTIONED**<br>lHkh mÙkj fy[kA	6 = ANM ए.एन.एम.<br>7 = OTHER HEALTH PERSONNEL अन्य स्वास्थ्य कर्मी<br>8 = ASHA आशा<br>9 = AWW आँगनवाड़ी कार्यकर्ता<br>10 = SBA/TRAINED DAI एसबीए/प्रशिक्षित दाई<br>11 = DAI दाई<br>12 = RMP आर.एम.पी. (झोला छाप डाक्टर)<br>13 = FAMILY MEMBER परिवार के सदस्य<br>14 = FRIENDS/NEIGHBOUR दोस्त/पड़ोसी<br>15 = RADIO/TV/NEWS PAPER रेडियो/टेलिविजन/समाचार पत्र<br>16 = MOBILE SMS मोबाइल एसएमएस<br>88 = OTHERS (SPECIFY) अन्य (स्पष्ट करें)<br>98 = NO ONE कोई नहीं<br>99 = DO NOT KNOW पता नहीं		
R36.	Were you told to visit a doctor/facility in case of any complications during delivery or in the first month after delivery?<br>क्या आपको यह बताया गया था कि प्रसव के समय या प्रसव के बाद एक महीने के अन्दर किसी भी जटिलता या समस्या होने पर डाक्टर/स्वास्थ्य केंद्र जायें?	1 = YES हाँ<br>0 = NO नहीं		
R37.	Did you experience any danger signs during the pregnancy termination/delivery or during the first month after the delivery?<br>प्रसव के समय या उसके बाद एक महीने के अन्दर क्या आपने किसी समस्या या परेशानी का अनुभव किया था?<br><br>**DO NOT READ LIST. CODE ALL MENTIONS IN FIRST COLUMN. THEN READ EACH ITEM BELOW (a-r) THAT WASN'T MENTIONED AND CODE RESPONSE IN SECOND COLUMN. IF NOT MENTIONED, SKIP TO NEXT LETTER.**<br>lph dk u i<A igy dkye e lHkh mYy[k fd; x; mÙkjk dk dkM djA blds ckn mu fcUnvk ¼,&vkj½ dk i< ftudk mYy[k ugh fd;k x;k Fkk vkj nlj dkye e mÙkj dk dkM djA ;fn ugh crk;k x;k vxy fcn ij tk;A	1 = YES, SPONTANEOUS<br>हाँ, स्वयं बताया	2 = YES, AFTER READING<br>हाँ, पढ़ने के बाद	0 = NO<br>नहीं
a.	Premature labor (less than 37 weeks) समय से पहले (37 सप्ताह से पहले) प्रसव पीड़ा	1	2	0
b.	Preterm/premature rupture of membrane समय से पहले झिल्ली का फटना	1	2	0
c.	Prolonged labor for more than 12 hours प्रसव पीड़ा लंबे समय तक (12 घंटे से अधिक) रहना	1	2	0
d.	Retained placenta खेड़ी का बाहर नहीं निकलना	1	2	0
e.	Obstructed labor रुकावट के साथ प्रसव पीड़ा होना	1	2	0
f.	Breech/mal presentation भ्रूण का गलत स्थिति में होना	1	2	0
g.	Excessive bleeding before delivery प्रसव के पहले अत्यधिक खून निकलना	1	2	0
h.	Excessive bleeding immediately after delivery प्रसव के तुरन्त बाद अत्यधिक खून निकलना	1	2	0
i.	High Blood Pressure उच्च रक्तचाप	1	2	0
j.	Difficulty breathing साँस लेने में कठीनाई	1	2	0
k.	Severe pain in abdomen पेट में तेज दर्द	1	2	0
l.	Severe weakness अत्यधिक कमजोरी	1	2	0
m.	Convulsions/fits दौरे पड़ना	1	2	0
n.	Foul smelling vaginal discharge योनी से बदबूदार स्राव	1	2	0
o.	Fever बुखार	1	2	0
p.	Severe headache तेज सिर दर्द	1	2	0
q.	Loss of consciousness बेहोश होना	1	2	0
r.	Blurred vision धुंधला दिखना	1	2	0
**R38a.**	Did you have sepsis after the delivery (HIGH FEVER WITH CHILLS, FOUL SMELLING VAGINAL DISCHARGE, PAIN IN THE LOWER ABDOMEN)?	1 = YES हाँ<br>0= NO नहीं		

	क्या आपको प्रसव के बाद संक्रमण हुआ था (तेज़ बुखार, कपकपी, योनी से बदबूदार पानी निकलना, पेट के निच्ले हिस्से में तेज़ दर्द) ?	
R38.	**ASK ONLY IF IN ANY OF THE RESPONSES R37(a-r)IS CODED 1 OR 2**<br>;g Á'u rHkh iNs tc **R37(a-r)** es fdlh mÙkj dk dkM 1 g<br><br>Did you seek treatment for this problem?<br>क्या आपने इस समस्या का इलाज कराया था?	1 = YES हाँ<br>0 = NO नहीं → **GO TO R40**
R39.	From where did you seek treatment?<br>आपने इलाज कहाँ कराया था?<br><br>**PROBE : ANY OTHERS?**<br>çkc s dN vkj\<br><br>**RECORD ALL MENTIONED**<br>lHkh mÙkj fy[kA	1 = GOVERNMENT/MUNICIPAL HOSPITAL सरकारी / नगरपालिका का अस्पताल<br>2 = GOVERNMENT DISPENSARY सरकारी दवाखाना<br>3 = UHC/UHP/UFWC यूएचसी / यूएचपी / यूएफडब्ल्यूसी<br>4 = CHC सीएचसी<br>5 = APHC/NPHC/BPHC एपीएचसी / एनपीएचसी / बीपीएचसी<br>6 = SUB-CENTER उपकेन्द्र<br>7 = ANGANWADI CENTER आँगनवाड़ी केन्द्र<br>8 = VHND ग्राम स्वास्थ्य एवं पोषण दिवस<br>9 = NGO HOSPITAL/CLINIC गैर सरकारी संस्था का अस्पताल / क्लीनिक<br>10 = PRIVATE HOSPITAL/CLINIC प्राइवेट अस्पताल / क्लीनिक<br>11 = MEDICINE SHOP दवा की दुकान<br>12 = FOLK HEALER ओझा / वैद्य / झाड़फूँक<br>13 = HOME REMEDIES घरेलू उपचार<br>14 = RMP आर.एम.पी. (झोला छाप डाक्टर)<br>88 = OTHERS (SPECIFY) अन्य (स्पष्ट करें)
**R30a.**	Did any health service provider give you this tablet **[SHOW MISOPROSTOL TABLETS]** to consume immediately after abortion?<br>क्या किसी स्वास्थ्य दाता ने आपके गर्भपात के तुरन्त बाद खाने के लिए यह गोली ¼feblkçkLVky dh xkfy;k fn[kk;½ दी थी?	1 = YES हाँ<br>= NO नहीं → **GO TO C**26A
**R30b.**	How many tablets did the health service provider give you?<br>स्वास्थ्य सेवा प्रदाता ने आपको कितनी गोलियाँ दी थी?	☐ NUMBER OF TABLETS गोलियों की संख्या
**R30c.**	How many tablets did you consume?<br>आपने कितनी गोलियाँ खाई थी?	☐ NUMBER OF TABLETS गोलियों की संख्या<br>0 = NO TABLETS CONSUMED कोई गोली नहीं खायी → **GO TO** C26A
R40.	Did the ASHA come to your home to check on you within 24 hours after the delivery?<br>गर्भसमापन के बाद 24 घंटे के अन्दर क्या आशा आपकी जाँच करने आपके घर आई थी?	1 = YES हाँ<br>0 = NO नहीं<br>99 = DO NOT KNOW पता नहीं
R41.	Did the AWW come to your home to check on you within 24 hours after the delivery?<br>गर्भसमापन के बाद 24 घंटे के अन्दर क्या आँगनवाड़ी कार्यकर्ता आपकी जाँच करने आपके घर आई थी?	1 = YES हाँ<br>0 = NO नहीं<br>99 = DO NOT KNOW पता नहीं
Now I would like to ask some questions about the child.		
**R41a**	Was the delivery caesarean?<br>क्या प्रसव आपरेशन द्वारा किया गया था?	1 = YES हाँ<br>0 = NO नहीं
R42.	Did the child/expulsed featus cry at birth, even if only a little bit?<br>क्या जन्म के बाद थोड़ी देर के लिए भी, बच्चा / निष्कासित भ्रूण रोया था?	1 = YES हाँ<br>0 = NO नहीं
R43.	Did the child/expulsed featus make any movement at birth, even if only a little bit?<br>क्या जन्म के बाद थोड़ी देर के लिए भी, बच्चा / निष्कासित भ्रूण हिला डुला था?	1 = YES हाँ<br>0 = NO नहीं
R44.	Did the child/expulsed featus breathe after birth, even if only a little bit?<br>क्या जन्म के बाद थोड़ी देर के लिए भी, बच्चा / निष्कासित भ्रूण ने साँस ली थी?	1 = YES हाँ<br>0 = NO नहीं
R45.	Was the child given assistance to breathe after birth?<br>क्या जन्म के बाद बच्चे को साँस लेने में सहायता दी गया थी?	1 = YES हाँ<br>0 = NO नहीं
R46.	Did the doctor/nurse/other health worker tell you that the child was alive or dead?	1 = ALIVE हाँ<br>2 = DEAD नहीं

	क्या किसी डाक्टर/नर्स/अन्य स्वास्थ्य प्रदाता ने आपको बताया था कि बच्चा जिन्दा पैदा हुआ था या मरा हुआ था?	9 = NOT APPLICABLE लागु नहीं<br>99 = DO NOT KNOW पता नहीं
R47.	**<u>ASK IF THE CHILD DID NOT MOVE, BREATHE OR CRY</u>**<br>iNsa ;fn cPpk fgyk Mqyk ugha Fkk] lkal ugha yh Fkh ;k jks;k ugha Fkk\<br>Was the child born alive or dead?<br>क्या बच्चा जिन्दा जन्मा था या मरा हुआ था?	1 = ALIVE हाँ<br>2= DEAD नहीं → **GO TO R48**
**R123**	How old was child when he/she died?<br>जब बच्चे की मृत्यु हुई उसकी आयु क्या थी?	☐ ☐☐<br>1 = HOURS घंटे<br>2 = DAYS दिन<br>3 = WEEKS सप्ताह<br>4 = MONTHS महीना<br>5 = YEARS साल<br>99 = DO NOT KNOW/REMEMBER पता नही/याद नहीं
**R48**	If the child was born dead, then did the child showed signs of decay?<br>;fn बच्चा मरा जन्मा था, क्या बच्चे को सड़न के लक्षण थे?	1 = YES हाँ<br>0 = NO नहीं
**R48a**	**<u>ASK IF CHILD WAS BORN DEAD (CODE 2 in R47)</u>**<br>What was the reason due to which the child was born dead?<br>बच्चे के मृत पैदा होने का कारण क्या था?<br><br>**RECORD ALL MENTIONED**<br>lHkh mÙkj fy[ksa	1 = PROLONGED LABOUR FOR MORE THAN 12 HOURS लंबे समय तक, 12 घंटे से अधिक समय तक प्रसव पीड़ा<br>2 = RETAINED PLACENTA गर्भनाल का अंदर रहना<br>3 = OBSTRUCTED LABOUR (MALPOSITION/PRESENTATION) रुकावट के साथ प्रसव (शिशु का गलत स्थिति में होना)<br>4 = SEVERE BLEEDING अत्यधिक खून निकलना<br>5 = SEVERE PAIN IN ABDOMEN पेट में तेज दर्द<br>6 = CONVULSIONS दौरे पड़ना<br>7 = FOUL SMELLING VAGINAL DISCHARGE योनि से बदबूदार पानी निकलना<br>88 = OTHERS (SPECIFY) अन्य (स्पष्ट करें)<br>99 DO NOT KNOW पता नहीं
**R48b**	**<u>ASK IF CHILD WAS BORN ALIVE (CODE 1 in R47)</u>**<br>What was the reason due to which the child died?<br>बच्चे की मृत्यु का कारण क्या था?<br>**RECORD ALL MENTIONED**<br>lHkh mÙkj fy[ksa	1 = DIARRHOEA दस्त<br>2 = FEVER बुखार<br>3 = ARI एआरआई<br>4 = CHILD WAS BORN PREMATURE समय से पूर्व जन्म<br>5 = CHILD WAS WEAK AT BIRTH बच्चा जन्म के समय कमजोर था<br>6 = ASPHYXIA दम घुटगया था<br>8 = OTHERS (SPECIFY) अन्य (स्पष्ट करें)<br>9 = DO NOT KNOW पता नहीं
**R49**	What was the gender of the child?<br>बच्चे का लिंग क्या था?	1 = MALE हाँ<br>2 = FEMALE नहीं<br>99 = DO NOT KNOW पता नहीं
**R51**	Did the pregnancy end earlier than expected?<br>क्या गर्भावस्था समय से पहले समाप्त हुआ था?	1 = YES हाँ<br>0 = NO नहीं
**R52**	How many weeks before the expected date of delivery did the pregnancy end?<br>प्रसव की सम्भावित तिथि से कितने सप्ताह पहले गर्भावस्था का समापन हुआ था?	☐☐ IN WEEKS सप्ताह में
**R53**	GOTO R119 IF CHILD BORN DEAD<br><br>Did the child stop moving in the womb before labour started?<br>क्या प्रसव शुरू होने से पहले बच्चा ने गर्भ मे हरकत करना बन्द कर दिया था?	1 = YES हाँ<br>0 = NO नहीं<br>99 = DO NOT KNOW पता नहीं
**R54**	Which part of the baby came first?<br>बच्चे का कौन सा हिस्सा पहले बाहर निकला था?	1 = HEAD सर<br>2 = BOTTOM बच्चा का पिछला हिस्सा<br>3 = FEET पैर<br>4 = ARM/HAND हाथ/बच्चा बाँह<br>88 = OTHERS (SPECIFY) अन्य (स्पष्ट करें)<br>99 = DO NOT KNOW पता नहीं
**R55**	Did the umblical cord come out before the baby was born?	1 = YES हाँ

	क्या प्रसव के बाद बच्चे के बाहर निकलने से पहले नाल बाहर निकला था?	0 = NO नहीं
**R56**	Was the umbilical cord wrapped several times (more than once) around the neck of the child at birth?<br>क्या बच्चे के जन्म के समय नाल बच्चे की गर्दन से लिपटा (कई बार) हुआ था?	1 = YES हाँ<br>0 = NO नहीं<br>99 DO NOT KNOW पता नहीं
**R56B**	What instrument was used to cut the umbilical cord?<br>नाल को काटने के लिए क्या प्रयोग किया गया था?<br><br>**PROBE : Was it new or used and from where did you get it?**<br>çkc% D;k ;g u;k Fkk ;k iqjkuk vkSj vkius mls dgk¡ ls çkIr fd;k\<br><br>**CODE ONLY ONE**<br>dsoy ,d mÙkj	1 = NEW BLADE FROM HOME/ DDK KIT<br>घर/डीडीके किट से नया ब्लेड<br>2 = USED BLADE पहले इस्तेमाल किया गया ब्लेड<br>3 = BLADE FROM SERVICE PROVIDER<br>सेवा प्रदाता का ब्लेड<br>4 = SCISSOR कैंची<br>88 = OTHERS (SPECIFY) अन्य (स्पष्ट करें)<br>99 DO NOT KNOW irk ugha
**R56C**	What was used to tie the cord?<br>नाल को बाँधने के लिए क्या प्रयोग किया गया था?<br><br>**PROBE : Was it new or used and from where did you get it?**<br>çkc% D;k ;g u;k Fkk ;k iqjkuk vkSj vkius mls dgk¡ ls çkIr fd;k\<br><br>**CODE ONLY ONE**<br>dsoy ,d mÙkj	1 = NEW THREAD FROM HOME/DDK KIT<br>घर/डीडीके किट से नया धागा<br>2 = USED THREAD पहले इस्तेमाल किया गया धागा<br>3 = THREAD FROM SERVICE PROVIDER<br>सेवा प्रदाता का धागा<br>4 = CORD CLIP गर्भनाल क्लिप<br>88 = OTHERS (SPECIFY) अन्य (स्पष्ट करें)<br>99 = DO NOT KNOW पता नहीं
**R57**	Was anything applied to the umbilical cord stump after birth?<br>क्या जन्म के बाद बच्चे की नाल पर कुछ लगाया गया था?	1 = YES हाँ<br>0 = NO नहीं<br>99 DO NOT KNOW पता नहीं
**R57A**	Was anything applied to the umbilicus after the cord dropped off?<br>क्या नाल गिरने के बाद नाभि पर कुछ लगाया गया था?	1 = YES हाँ<br>0 = NO नहीं<br>99 DO NOT KNOW पता नहीं
**R57B**	**ASK IF R57=1 OR R57A=1**<br><br>What was applied to the cord after cutting and tying?<br>नाल को बाँधने और काटने के बाद क्या लगया गया था?<br><br>**PROBE : ANY OTHERS?**<br>çkc % dqN vkSj\<br><br>**RECORD ALL MENTIONED**<br>lHkh mÙkj fy[ksA	1 = DETTOL/SAVLON डेटाल/सैव्लोन<br>2 = ALCOHOL/SPIRIT शराब/स्प्रिट<br>3 = MUSTARD OIL सरसों का तेल<br>4 = SINDOOR सिंदूर<br>5 = GHEE घी<br>6 = BORIC POWDER बोरिक पाउडर<br>7 = GENTIAN VIOLET (NEELI DAWAI)<br>जेनसिन व्वाइलेट (नीली दवा)<br>8 = TALCUM POWDER टेलकम पाउडर<br>9 = COW DUNG गाय का गोबर<br>10 = ASH राख<br>11 = CHLOROHEXIDINE क्लोरोहेक्सिडाइन<br>88 = OTHERS (SPECIFY) अन्य (स्पष्ट करें)<br>99 DO NOT KNOW पता नहीं
**R57B1**	How long after the delivery was anything applied to the cord?<br>बच्चे के जन्म के कितने समय बाद नाल पे कुछ लगाया गया था?	☐☐☐<br>0= IMMEDIATELY /MINUTES(less than 1 hours) तुरंत/मिनट में (1 घण्टे के अन्दर)<br>1 = HOURS घंटे<br>2 = DAYS दिन<br>99 = DO NOT KNOW पता नहीं
**R57C1**	After delivery, was the baby wiped dry, wiped with a wet cloth, or bathed?<br>प्रसव के बाद क्या बच्चे को सुखे कपडे से पोछा गया, गीले कपड़े से पोंछा गया या नहलाया गया?	1 = WIPED WITH A DRY CLOTH सुखे कपड़े से पोंछा गया<br>2 = WIPED WITH A WET CLOTH गीले कपड़े से पोंछा गया<br>3 = BATHED नहलाया गया<br>99 = DO NOT KNOW पता नहीं
**R57c1 a**	**ASK IF R57C1=1**<br><br>How soon after the delivery was the baby wiped dry?<br>प्रसव के कितने समय बाद क्या बच्चे को सुखे कपडे से पोछा गया?	☐☐☐<br>0=MINUTES<br>1 = HOURS घंटे<br>2 = DAYS दिन<br>98 = NOT WIPED DRY पोचा नहीं गया<br>99 = DO NOT KNOW पता नहीं
**R57C2**	Was the baby wrapped in a cloth after delivery?	1 = YES हाँ

	प्रसव के बाद क्या बच्चे को कपड़े में लपेटा गया था?	0 = NO नहीं<br>99 = DO NOT KNOW पता नहीं
**R57C3**	**ASK IF R57C2=1, ELSE SKIP TO R57C**<br><br>Was the child wrapped in the same cloth used for wiping?<br>क्या बच्चे को जिस कपडे से पोचा था, उसी कपडे से लपेटा था?	1 = YES हाँ<br>0 = NO नहीं<br>99=DO NOT KNOW पता नहीं
**R57C**	How soon after the delivery was your child given (his/her) first bath?<br>प्रसव के कितने समय बाद आपने आपके बच्चे को पहली बार नहलाया गया था?<br><br>**CODE ONLY ONE**<br>doy ,d mÙkj	☐ ☐☐<br>0 = IMMEDIATELY (less than 1 hours) तुरंत (1 घंटे के अन्दर)<br>1 = HOURS घंटे<br>2 = DAYS दिन<br>98 = NOT BATHED अभी तक नहलाया नहीं गया<br>DO NOT KNOW पता नहीं
**R57C3**	At any time when you were in the labour room or during your stay in the hospital, were you advised by the nurse or anyone else to keep BABY naked on your chest, next to your skin?<br>जब आप प्रसव कक्ष में थीं या अस्पताल मे थीं तब क्या नर्स या किसी और ने आपको यह बताया था कि बच्च को त्वचा से त्वचा से संपर्क बना कर बिना कपड़ों के अपनी छाती पर रखना है।<br><br>**SHOW PICTURES OF SKIN TO SKIN CONTACT FROM WHO GUIDELINES.**<br>fo'o LokLF; laxBu ds fn'kkfunsZ'kksa ls Ropk ls Ropk laidZ ds fp= fn[kk;sA	1 = YES हाँ<br>0 = NO नहीं
**R57D**	Did you practice keeping your child in that position after the child was born?<br>प्रसव के कितनी देर बाद आपने अपने बच्चे को इस स्थिति में रखा था?	1= YES हाँ<br>0= NO नहीं
**R57G 1**	Did the ASHA/AWW/ANM advice you to keep [CHILD NAME] naked on your chest, next to your skin?<br>क्या आपको ए.एन.एम./आशा/आँगनवाड़ी कार्यकर्ता ने (बच्चे का नाम) को त्वचा से त्वचा के बीच संपर्क बना कर बिना कपड़ों के अपनी छाती पर रखने का सुझाव दिया था?<br>**SHOW PICTURES OF SKIN TO SKIN CONTACT FROM WHO GUIDELINES.**<br>fo'o LokLF; laxBu ds fn'kkfunsZ'kksa ls Ropk ls Ropk laidZ ds fp= fn[kk;sA	1 = YES हाँ<br>0 = NO नहीं
**R57h1**	Did you practice keeping [CHILD NAME] in this position while at home?<br>क्या घर पर आपने (बच्चे का नाम) को इस स्थिति में रखा था?	1 = YES हाँ<br>0 = NO नहीं
**R57H2**	**ASK IF R57D=1 OR R57H1=1, ELSE SKIP TO R57G**<br>How soon after the delivery did you first keep your child in that position?<br>प्रसव के कितनी देर बाद आपने अपने बच्चे को पहली बार इस अवस्था मे रक्खा था?	☐ ☐☐<br>1 = HOURS घंटे<br>2 = DAYS दिन<br>99 = DO NOT KNOW/REMEMBER पता नही/याद नहीं
**R57H3**	How much time did you keep your child in that position, the first time?<br>पहली बार आपने अपने बच्चे को इस अवस्था मे कितनी देर तक रक्खा था?	☐ ☐☐<br>1 = HOURS घंटे<br>2 = DAYS दिन<br>99 = DO NOT KNOW/REMEMBER पता नही/याद नहीं
**R57I1**	For how many days after birth did you practice this?<br>जन्म के बाद कितने दिनों तक आपने यह किया था?	☐☐ NUMBER OF DAYS दिनों की संख्या<br>99 = DO NOT KNOW/REMEMBER पता नहीं/याद है
**R57J1**	For how many hours each day?<br>हर दिन कितने घंटे?	☐☐.☐☐ HOURS घंटे
**R57G**	Did you ever breastfeed the child?<br>क्या बच्चे को आपने कभी स्तनपान कराया?	1 = YES हाँ → **GO TO R57j**<br>0 = NO नहीं
**R57H**	How long after birth did you first put [CHILD NAME] to the breast?<br>जन्म के कितने समय बाद आपने (बच्चे का नाम) को स्तनों से लगाया?<br><br>**IF LESS THAN 1 HOUR, CODE '00.' IF LESS THAN 24 HOURS, RECORD HOURS. OTHERWISE, RECORD DAYS.**	☐ ☐☐<br>00 = IMMEDIATELY/WITHIN 1 HOUR तुरन्त/1 घंटे के अन्दर<br>1 = HOURS घंटे<br>2 = DAYS दिन<br>99 DO NOT KNOW पता नहीं

	यदि एक घंटे से कम तो '00' का कोड करें। यदि 24 घंटे से कम तो घंटे मे लिखें। नहीं तो दिनों मे लिखें।	
**R57I**	Did you feed [CHILD NAME] your first yellow thick milk (local term)?<br>क्या आपने (बच्चे का नाम) को अपना पहला गाढ़ा पीला दूध पिलाया था (स्थानीय शब्दों में)?	1 = YES हाँ<br>0 = O नहीं
**R57J**	Did you or anyone else give [CHILD NAME] anything such as honey, water, tea, jaggary, ghutti before giving breast milk for the first time?<br>क्या (बच्चे का नाम) को पहली बार स्तनपान कराने से पहले आपने या किसी और ने शहद, पानी, चाय, गुड़, घुट्टी जैसा कुछ दिया था?	1 = YES हाँ<br>0 = NO नहीं<br>99 DO NOT KNOW पता नहीं
**R57K**	Did you or anyone else give [CHILD NAME] anything such as honey, water, tea, jaggary, ghutti other than breast milk within the first three days after birth?<br>क्या (बच्चे का नाम) को जन्म के पहले तीन दिन में आपने या किसी और ने शहद, पानी, चाय, गुड़, घुट्टी जैसा कुछ दिया था?	1 = YES हाँ<br>0 = NO नहीं<br>99 DO NOT KNOW पता नहीं
**R57L**	**ASK IF '1' IN r57j or r57k**<br>;fn ,Q9 ;k ,Q10 e mÙkj 1 gk rk ;g ç'u iNA<br><br>What was [CHILD NAME] given?<br>(बच्चे का नाम) को और क्या दिया गया था?<br><br>**PROBE : ANY OTHERS?**<br>çkc % dN vkj\<br><br>**RECORD ALL MENTIONED**<br>lHkh mÙkj fy[kA	1 = MILK OTHER THAN BREAST MILK (ANIMAL MILK) स्तनपान के अलावा (पशुओं का दूध)<br>2 = PLAIN WATER सादा पानी<br>3 = SUGAR OR GLUCOSE WATER चीनी या ग्लूकोज का पानी<br>4 = GRIPE WATER ग्राइप वाटर<br>5 = SUGAR-SALT-WATER SOLUTION चीनी–नमक का घोल<br>6 = FRUIT JUICE फलों का रस<br>7 = INFANT FORMULA/LACTOGEN शिशु फार्मूला/लैक्टोजिन<br>8 = TEA/COFEE चाय/काफी<br>9 = HONEY शहद<br>10 = JANAM GHUTTI जन्म घुट्टी<br>88 OTHER (SPECIFY) अन्य (स्पष्ट करें)
**R58a**	Was the child weighed at the birth?<br>क्या बच्चे का वजन किया गया था जन्म के समय?	1 = YES हाँ<br>0 = NO नहीं<br>99 = DO NOT KNOW पता नहीं
**R59**	What was the birth weight of the baby?<br>बच्चे का जन्म के समय वजन क्या था?<br><br>**RECORD IN KILOGRAMS**<br>fdykxke e nt dj	☐☐. ☐☐ KILOGRAMS किलोग्राम<br>9998 = NEVER WEIGHED कभी वजन नहीं लिया गया<br>9999 O NOT KNOW पता नहीं
**R60**	Did the child have any noticeable malformation?<br>क्या बच्चे को कोई विकृति थी जो दिख रही थी?	1 = YES हाँ<br>0 = NO नहीं<br>99 = DO NOT KNOW पता नहीं
**R61**	Did the child have a swelling/defect on the back?<br>क्या बच्चे की पीठ पर कोई सूजन/दोश था?	1 = YES हाँ<br>0 = NO नहीं<br>99 = DO NOT KNOW पता नहीं
**R62**	Did the child have a very large head?<br>क्या बच्चे का सिर बहुत बड़ा था?	1 = YES हाँ<br>0 = NO नहीं<br>99 = DO NOT KNOW पता नहीं
**R63**	Did the child have a very small head?<br>क्या बच्चे का सिर बहुत छोटा था?	1 = YES हाँ<br>0 = NO नहीं<br>99 = DO NOT KNOW पता नहीं
**R64**	What was the colour of child at birth?<br>जन्म के समय बच्चे का रंग क्या था?	1= NORMAL सामान्य GOTO R66<br>2= ABNORMAL असामान्य<br>99 = DO NOT KNOW पता नहीं GOTO R66
**R64a**	IF OPTION 2 IS CODED FOR R64, ASK ELSE SKIP TO R66<br><br>What was the colour of the child at birth?<br>क्या रंग था?	1= YELLOWपीला<br>2=BLUE नीला<br>99 = DO NOT KNOW पता नहीं
**R66**	Did the child become unresponsive or unconscious soon after birth (less than 24 hours)?<br>क्या जन्म के बाद (24 घंटे से अन्दर) बच्चा बेहोश या अनुत्तरदायी हुआ था?	1 = YES हाँ<br>0 = NO नहीं<br>99 = DO NOT KNOW पता नहीं

**R67**	Did the child become unresponsive or unconscious more than 1 day after birth?<br>क्या जन्म के 1 दिन के बाद बच्चा बेहोश या अनुत्तरदायी हुआ था?	1 = YES हाँ<br>0 = NO नहीं<br>99 = DO NOT KNOW पता नहीं
**R68**	Did the child suffer from any injury or accident that led to his/her death?<br>क्या बच्चे को कोई चोट लगी थी/दुर्घटना हुई थी जिस्से उसकी मृत्यु हुई थी?	1 = YES हाँ<br>0 = NO नहीं<br>99 = DO NOT KNOW पता नहीं
**R69**	Did the child suffer from any illness before his/her death?<br>क्या मृत्यु से पहले बच्चे को कोई बिमारी हुई थी?	1 = YES हाँ<br>0 = NO नहीं<br>99 = DO NOT KNOW पता नहीं
**R70**	For how long was the child ill before his/her death?<br>बिमारी के कितनी समय बाद बच्चे का मृत्यु हुई थी?	☐ ☐☐<br>1 = HOURS घंटे<br>2 = DAYS दिन<br>3 = WEEKS सप्ताह<br>4 = MONTHS महीना<br>99 = DO NOT KNOW/REMEMBER पता नही/याद नहीं
**R71**	Was the child able to suckle or bottle-feed within the first 24 hours after birth?<br>क्या जन्म के बाद पहले 24 घंटों में बच्चा स्तनपान या बोतल से दूध पी पा रहा था/रही थी?	1 = YES हाँ<br>0 = NO नहीं → **GO TO R73**<br>99 = DO NOT KNOW पता नहीं → **GO TO** Error! Reference source not found.
**R72**	Did the child stop suckling of bottle feeding 3 days after birth?<br>क्या जन्म के 3 दिनों के बाद 24 बच्चे ने स्तनपान या बोतल से दूध पीना बन्द कर दिया था?	1 = YES हाँ<br>0 = NO नहीं<br>99 = DO NOT KNOW पता नहीं
**R73**	Did the child ever have convulsions?<br>क्या बच्चे को कभी भी दौरे पड़े थे?	1 = YES हाँ<br>0 = NO नहीं<br>99 = DO NOT KNOW पता नहीं
**R74**	Did the child have convulsions within the first 24 hours after birth?<br>क्या जन्म के बाद पहले 24 घंटों में बच्चे को दौरे पड़े थे?	1 = YES हाँ<br>0 = NO नहीं<br>99 = DO NOT KNOW पता नहीं
**R75**	Did the child have convulsions starting on the second day or later day after birth?<br>क्या जन्म के बाद पहले दूसरे दिन या उसके बाद बच्चे को दौरे पड़ना शुरू हुआ था?	1 = YES हाँ<br>0 = NO नहीं<br>99 = DO NOT KNOW पता नहीं
**R76**	Did the child's body become stiff, with the back arched backwards?<br>क्या बच्चे का शरीर जकड़ा हुआ था और उसकी पीठ पीछे की तरफ मुडी हुई थी?	1 = YES हाँ<br>0 = NO नहीं<br>99 = DO NOT KNOW पता नहीं
**R79**	Did the child have fever?<br>क्या बच्चे को बुखार था?	1 = YES हाँ<br>0 = NO नहीं<br>99 = DO NOT KNOW पता नहीं
**R80**	Did the child become cold to touch before death?<br>क्या बच्चा मृत्यु से पहले छुने पर ठंडा लगता था?	1 = YES हाँ<br>0 = NO नहीं<br>99 = DO NOT KNOW पता नहीं
**R81**	Did the child have cough?<br>क्या बच्चे को खाँसी हुई थी?	1 = YES हाँ<br>0 = NO नहीं<br>99 = DO NOT KNOW पता नहीं
**R82**	Did the child make a whooping sound when coughing?<br>क्या बच्चे खाँसते समय घरघराने जैसी आवाज कर रहा था?	1 = YES हाँ<br>0 = NO नहीं<br>99 = DO NOT KNOW पता नहीं
**R83**	Did the child have difficulty breathing?<br>क्या बच्चे को साँस लेने में परेशानी थी?	1 = YES हाँ<br>0 = NO नहीं
**R84**	Did the child have fast breathing?<br>क्या बच्चा तेजी के साथ साँस लेता था या जोर जोर से साँस लेता था?	1 = YES हाँ<br>0 = NO नहीं<br>99 = DO NOT KNOW पता नहीं
**R85**	Did the child have breathlessness?<br>क्या बच्चे की साँस फूल रही थी?	1 = YES हाँ<br>0 = NO नहीं<br>99 = DO NOT KNOW पता नहीं
**R86**	Did you see the lower chest wall/ribs being pulled in as the child breathed?<br>क्या साँस लेते समय बच्चे की छाती की निचला हिस्सा/पसलियाँ धस रही थी?	1 = YES हाँ<br>0 = NO नहीं<br>99 = DO NOT KNOW पता नहीं
**R87**	Did the child have noisy breathing (grunting or wheezing)?<br>क्या बच्चा साँस लेते समय आवाज (घुरघुराहट/खरखराहट) करता था?	1 = YES हाँ<br>0 = NO नहीं

		99 = DO NOT KNOW पता नहीं
**R88**	Did the child have diarrhea?<br>क्या बच्चे को दस्त हुआ था?	1 = YES हाँ<br>0 = NO नहीं<br>99 = DO NOT KNOW पता नहीं
**R89**	Was there blood in the stools?<br>क्या बच्चे को शौच के साथ खुन आया था?	1 = YES हाँ<br>0 = NO नहीं<br>99 = DO NOT KNOW पता नहीं
**R90**	Did the child vomit?<br>क्या बच्चे को ऊल्टी हुई थी?	1 = YES हाँ<br>0 = NO नहीं<br>99 = DO NOT KNOW पता नहीं
**R91**	Did the child vomit bright red blood?<br>क्या बच्चे की ऊल्टी में लाल खून था?	1 = YES हाँ<br>0 = NO नहीं<br>99 = DO NOT KNOW पता नहीं
**R93**	Did the child have a more than usual protruding abdomen?<br>क्या बच्चे का पेट सामान्य से अधिक बाहर निकला हुआ था?	1 = YES हाँ<br>0 = NO नहीं<br>99 = DO NOT KNOW पता नहीं
**R94**	Did the child have redness or discharge from the umblical cord stump?<br>क्या बच्चे की नाभी में लाली या उससे कोई रिसाव था?	1 = YES हाँ<br>0 = NO नहीं<br>99 = DO NOT KNOW पता नहीं
**R95**	Did the child have yellow palms or soles?<br>क्या बच्चे के हथेलियाँ या पैरों के तलवे पीले थे?	1 = YES हाँ<br>0 = NO नहीं<br>99 = DO NOT KNOW पता नहीं
**R96**	Did the child have any skin problems?<br>क्या बच्चे को त्वचा की परेशानी थी?<br>**EXPLAIN ULCERS, SORES, ABCESS.**<br>QkM] ukl j] ?kko dk ckjs es le>k;A	1 = YES हाँ<br>0 = NO नहीं<br>99 = DO NOT KNOW पता नहीं
**R98**	Did s/he have abdominal pain?<br>क्या बच्चे के पेट में दर्द था?	1 = YES हाँ<br>0 = NO नहीं<br>99 = DO NOT KNOW पता नहीं
**R99**	Did s/he have any mass in the abdomen?<br>क्या बच्चे के पेट में कोई मास था?	1 = YES हाँ<br>0 = NO नहीं<br>99 = DO NOT KNOW पता नहीं
**R102**	Did s/he become unconscious?<br>क्या बच्चे बेहोश हुआ था?	1 = YES हाँ<br>0 = NO नहीं<br>99 = DO NOT KNOW पता नहीं
**R103**	Did s/he have paralysis of the lower limbs?<br>क्या बच्चे के शरीर के निचले भाग मे लकवा हुआ था?	1 = YES हाँ<br>0 = NO नहीं<br>99 = DO NOT KNOW पता नहीं
**R104**	Was there any change in the amount of urine s/he passed daily?<br>क्या बच्चे के रोज की पिशाप की मात्रा में कोई अन्तर आया था?	1 = YES हाँ<br>0 = NO नहीं<br>99 = DO NOT KNOW पता नहीं
**R105**	Did s/he have red eyes?<br>क्या बच्चे के आँखें लाल थी?	1 = YES हाँ<br>0 = NO नहीं<br>99 = DO NOT KNOW पता नहीं
**R106**	Did s/he have bleeding from the nose, mouth, or anus?<br>क्या बच्चे नाक, मुँह या गुदा से खुन आता था?	1 = YES हाँ<br>0 = NO नहीं<br>99 = DO NOT KNOW पता नहीं
**R107**	Did s/he have weight loss?<br>क्या बच्चे का वजन घटा था?	1 = YES हाँ<br>0 = NO नहीं<br>99 = DO NOT KNOW पता नहीं
**R108**	Did s/he have mouth sores or white patches in the mouth or on the tongue?<br>क्या बच्चे के मुँह या जीभ पर घाव या सफेद धब्बा था?	1 = YES हाँ<br>0 = NO नहीं<br>99 = DO NOT KNOW पता नहीं
**R109**	Did s/he have any swelling? For example, face, joints, ankle, whole body etc.<br>क्या बच्चे के मुँह, टखना, जोंड़ो या पूरे शरीर आदि पर सूजन था?	1 = YES हाँ<br>0 = NO नहीं<br>99 = DO NOT KNOW पता नहीं
**R110**	Did s/he have any lumps? For example, in neck, armpit, groin etc.<br>क्या बच्चे के गरदन, बगल, ऊसन्धि आदि पर गांठे थी?	1 = YES हाँ<br>0 = NO नहीं<br>99 = DO NOT KNOW पता नहीं
**R111**	Did s/he have yellow discoloration of the eyes?<br>क्या बच्चे के आँखें पीली थी?	1 = YES हाँ<br>0 = NO नहीं

		99 = DO NOT KNOW पता नहीं
**R112**	Did her/his hair color change to reddish or yellowish?<br>क्या बच्चे के बालों का रंग बदल कर कुछ लाल सा या पीला सा हो गया था?	1 = YES हाँ<br>0 = NO नहीं<br>99 = DO NOT KNOW पता नहीं
**R113**	Did s/he look pale (thinning/lack of blood) or have pale palms, eyes or nail beds?<br>क्या बच्चा पीला लगता था या उसके हाथेली, आंखें या नाखूनो का रंग पीला सा हो गया था? (खून की कमी के कारण)	1 = YES हाँ<br>0 = NO नहीं<br>99 = DO NOT KNOW पता नहीं
**R114**	Did s/he have sunken eyes?<br>क्या बच्चे की आँखें धसी हुई थी?	1 = YES हाँ<br>0 = NO नहीं<br>99 = DO NOT KNOW पता नहीं
Read out to the respondent: Now, I would like to ask some questions about the treatment that the child received before death.<br>mÙkjnkrk dk i<dj lquk;s vc esa vkils vkids cPps ds eR;q ls igys djk;k byktds ckjs esa dqN ç'u iNwxhA		
**R115**	Did child ever receive any vaccinations to prevent (him/her) from getting diseases?<br>क्या बच्चे को कभी बिमारियों से बचाव के लिये कोई टीका लगा था?<br>**INCLUDE VACCINATIONS RECEIVED IN A PULSE POLIO PROGRAM**<br>iYl i®yh;® dk;Øe es **दिए गए टीकों को भी भाामिल करें।**	1 = YES हाँ<br>0 = NO नहीं<br>99 = DO NOT KNOW पता नहीं
**R116**	Did the child receive any treatment for the illness that led to death?<br>जिस बिमारी से बच्चे की मृत्यु हुई थी, क्या उस बिमारी का इलाज कराया गया था?	1 = YES हाँ<br>0 = NO नहीं<br>99 = DO NOT KNOW पता नहीं
**R117**	Where did the child receive treatment?<br>बच्चे का इलाज कहाँ कराया गया था?<br><br>**PROBE : ANY OTHERS?**<br>çkc % dqN vkj\<br><br>**RECORD ALL MENTIONED**<br>lHkh mÙkj fy[kA	1 = GOVERNMENT/MUNICIPAL HOSPITAL सरकारी/नगरपालिका का अस्पताल<br>2 = GOVERNMENT DISPENSARY सरकारी दवाखाना<br>3 = UHC/UHP/UFWC यूएचसी/यूएचपी/यूएफडब्ल्यूसी<br>4 = CHC सीएचसी<br>5 = APHC/NPHC/BPHC एपीएचसी/एनपीएचसी/बीपीएचसी<br>6 = SUB-CENTER उपकेन्द्र<br>7 = ANGANWADI CENTER आँगनवाड़ी केन्द्र<br>8 = VHND ग्राम स्वास्थ्य एवं पोष्ण दिवस<br>9 = NGO HOSPITAL/CLINIC गैर सरकारी संस्था का अस्पताल/क्लीनिक<br>10 = PRIVATE HOSPITAL/CLINIC प्राइवेट अस्पताल/क्लीनिक<br>11 = MEDICINE SHOP दवा की दुकान<br>12 = FOLK HEALER ओझा/वैद्य/झाड़फूँक<br>13 = HOME REMEDIES घरेलू उपचार<br>14 = RMP आर.एम.पी. (झोला छाप डाक्टर)<br>88 = OTHERS (SPECIFY) अन्य (स्पष्ट करें)
**R118**	How many contacts did you have with health providers for treatment of the child?<br>बच्चे की बिमारी के इलाज के लिए स्वास्थ्य प्रदाता से आपकी कितनी बार मुलाकात हुई थी?	☐☐ NO. OF TIMES इतनी बार<br>99 = DO NOT REMEMBER याद नहीं
**R119**	Did the health provider tell you the cause of death of child?<br>क्या स्वास्थ्य कार्यकर्ता ने आपको बच्चे की मृत्यु का कारण बताया था?	1 = YES हाँ<br>0 = NO नहीं → **GO TO R121**<br>99 = DO NOT KNOW पता नहीं → **GO TO R121**
**R120**	What was the cause of death according to the health provider?<br>स्वास्थ्य कार्यकर्ता के अनुसार बच्चे की मृत्यु का क्या कारण था?	1 = DIARRHOEA दस्त<br>2 = FEVER बुखार<br>3 = ARI एआरआई<br>4 = CHILD WAS BORN PREMATURE समय से पूर्व जन्म<br>5 = CHILD WAS WEAK AT BIRTH बच्चा जन्म के समय कमजोर था<br>6 = ASPHYXIA दम घुटगया था<br>8 = OTHERS (SPECIFY) अन्य (स्पष्ट करें)<br>9 = DO NOT KNOW पता नहीं
**R121**	Where did the death occur?	1 = HOME घर पर

	बच्चे की मृत्यु कहाँ हुई थी?	2 = HEALTH FACILITY स्वास्थ्य सुविधा<br>88 = OTHERS (SPECIFY) अन्य (स्पष्ट करें)
**R122**	Do you have a death registration number for the child?<br>क्या आपके पास बच्चे का मृत्यु पंजीकरण है?	1 = YES हाँ<br>0 = NO नहीं<br>99 DO NOT KNOW पता नहीं

J.ADOLOSCENT HEALTH (RC5)

This section will be asked to adolescent girls of 10-19 years

***Read out to the respondent**: I would like to you some questions regarding some services for adolescents that you might have received from the FLWs recently.*
mÙkjnkrk dk i<dj lquk;s vc esa vkils fd'kksfj;ksa ds fy;s dqN ,slh lsokvksa ds ckjs esa Á'u iwNuk pkgwaxh tks gky esa vk'kk] vkxuokM+h dk;ZdrkZ vkSj ,,u,e }kjk Ánku dh x;h gSA

J3.	Have you ever been tested for hemoglobin (blood test)?<br>क्या कभी भी आपकी हीमोग्लोबिन की जाँच (रक्त जाँच) की गयी थी?	1 = YES हाँ<br>0 = NO नहीं → **GO TO J7**
J4.	Were you tested for hemoglobin (blood test) in the last three months?<br>क्या पिछले तीन महीने में आपकी हीमोग्लोबिन की जाँच (रक्त जाँच) की गयी थी?	1 = YES हाँ<br>0 = NO नहीं
J5.	Have you ever received IFA from an ASHA/AWW/ANM?<br>क्या आपने आशा/आँगनवाड़ी कार्यकर्ता/ए.एन.एम. से आई.एफ.ए. गोली (ताकत की लाल गोली) मिली है?	1 = YES हाँ<br>0 = NO नहीं
J6.	Have you ever received IFA at school?<br>क्या आपको कभी स्कूल में आई.एफ.ए. गोली मिली है?	1 = YES हाँ<br>0 = NO नहीं
J7.	**ASK ONLY IF CODED '1' IN EITHER J5 OR J6, ELSE GO TO J12**<br>तभी पूछें जब जे5 या जे6 में 1 का कोड दिया गया है। नहीं तो जे10 पर जायें।<br><br>Did you receive IFA tablets in the last one month?<br>क्या आपने पिछले एक महीने में आई.एफ.ए. की गोलियाँ प्राप्त की हैं?	1 = YES हाँ<br>0 = NO नहीं → **GO TO J14**
J8.	How many IFA tablets did you receive?<br>आपने आईएफए की कितनी गोलियाँ प्राप्त की हैं?	☐☐☐ NUMBER OF TABLETS RECEIVED प्राप्त की गयी गोलियाँ<br>999 = DO NOT KNOW पता नहीं
J9.	How many IFA tablets did you consume?<br>आपने आईएफए की कितनी गोलियाँ खाई?	☐☐☐ NUMBER OF TABLETS CONSUMED खायी गयी गोलियाँ<br>999 = DO NOT KNOW पता नहीं
J10.	Did the ASHA/AWW/ANM tell you about problems that you may face due to consuming the IFA tablets?<br>क्या आशा/आँगनवाड़ी कार्यकर्ता/ए.एन.एम. ने आपको उन समस्याओं के बारे में बताया जो आई.एफ.ए. की गोलियाँ लेने की वजह से हो सकती हैं?	1 = YES हाँ<br>0 = NO नहीं → **GO TO J14**
J11.	What problems did the ASHA/AWW/ANM tell you about?<br>आशा/आँगनवाड़ी कार्यकर्ता/ए.एन.एम. ने किन समस्याओं के बारे में बताया था?<br><br>**PROBE : ANY OTHERS?**<br>çkc % dqN vkSj\<br><br>**RECORD ALL MENTIONED**<br>lHkh mÙkj fy[kA	1 = BLACK STOOLS काला मल<br>2 = NAUSEA उबकाई<br>3 = FATIGUE थकान<br>4 = CONSTIPATION कब्ज<br>5 = DIARRHEA दस्त<br>6 = STOMACH CRAMPS/UPSET STOMACH पेट में ऐंठन/पेट खराब होना<br>7 = DÁZINESS चक्कर आना<br>8 = RASH/ITCHING चकत्ते पड़ना/खुजली होना<br>9 = TROUBLE BREATHING साँस लेने में दिक्कत<br>88 = OTHERS (SPECIFY) अन्य (स्पष्ट करें)
J12.	Did you receive any dose of Albendazole (400 mg) tablet for deworming in the last 6 months?<br>क्या पिछले 6 महीने में पेट के कीड़े मारने के लिये आपको अल्बेंडाजोल की गोली (400 मिग्राम) मिली है?	1 = YES हाँ<br>0 = NO नहीं → **GO TO J16**
J13.	Did you consume the Albendazole (400 mg) tablet for deworming in the last 6 months?<br>क्या पिछले 6 महीने में पेट के कीड़े मारने के लिये आपने अल्बेंडाजोल की गोली (400 मिग्राम) गोली खाई है?	1 = YES हाँ<br>0 = NO नहीं<br>99 = DO NOT KNOW पता नहीं
J14.	When did your last menstrual period start?	☐ ☐☐☐

	आपकी पिछली माहवारी कब शुरू हुई थी?	1 = DAYS AGO दिन पहले<br>2 = WEEKS AGO सप्ताह पहले<br>3 = MONTHS AGO महीना पहले<br>77 = NOT YET STARTED अभी शुरू नहीं हुई है → **GO TO J18**
J15.	What material do you use during menstruation?<br>महवारी के दौरान आप किस चीज का उपयोग करती हैं?<br><br>**PROBE : ANY OTHERS?**<br>çkc ¼ dN vkj\<br><br>**RECORD ALL MENTIONED**<br>lHkh mÙkj fy[kA	1 = SANITARY PAD सेनिटरी पैड<br>2 = OLD CLOTH (SAME CLOTH RE-USED) पुराना कपड़ा (उसी कपड़े का फिर से उपयोग)<br>3 = NEW CLOTH (DISPOSED AFTER EVERY USE) नया कपड़ा (हर इस्तेमाल के बाद फेंकने वाला) 88 = OTHERS (SPECIFY) अन्य (स्पष्ट करें)
J16.	**ASK IF USING SANITARY PAD**<br>From where do you get the sanitary pad for use during menstruation?<br>महवारी के दौरान उपयोग के लिये आपने सेनिटरी पैड कहाँ से खरीदा?<br><br>**PROBE : ANY OTHERS?**<br>çkc ¼ dN vkj\<br><br>**RECORD ALL MENTIONED**<br>lHkh mÙkj fy[kA	1 = GENERAL STORE जनरल स्टोर<br>2 = PHARMACY दवा की दुकान<br>3 = FLW आशा/आँगनवाड़ी कार्यकर्ता/ए.एन.एम.<br>88 = OTHERS (SPECIFY) अन्य (स्पष्ट करें
J17.	**ASK IF GOT SANITARY PADS FROM FLW**<br>Did you ever buy sanitary napkins from ASHA/AWW/ANM in the last six months?<br>क्या पिछले 6 महीने में आपने कभी आशा/आँगनवाड़ी कार्यकर्ता/ए.एन.एम. से सेनिटरी नेपकिन प्राप्त किये हैं?	1 = YES हाँ<br>0 = NO नहीं

J18.	Are you usually allowed to go to the following places alone, only with someone else, or not at all?<br>क्या आपको निम्नलिखित स्थानों पर अकेले जाने दिया जाता है, या किसी और के साथ या बिल्कुल भी नहीं जाने दिया जाता है?	ALONE<br>अकेले	WITH SOMEONE ELSE ONLY<br>केवल किसी और के साथ	NOT AT ALL<br>बिल्कुल भी नहीं जाने दिया जाता
a.	Health centre or clinic स्वास्थ्य केन्द्र या क्लीनिक	1	2	3
b.	Friend's or relative's house दोस्तों या सम्बन्धियों के घर	1	2	3
c.	To the market बाजार जाना	1	2	3

Now I would like to know from you if you are aware of anaemia

अब मैं आपसे जानना चाहूंगी के आपको अनेमिया के बारें में कितनी जानकारी है

**J18a**	Are you aware about what anaemia is?<br><br>क्या आपको अनेमिया के बारें में पता है, जो आयरन की कमी की वजह से होता है?	1 = YES हाँ<br><br>0 = NO नहीं **SKIP TO J18c**
**J18b**	**ASK IF YES FOR J18a**<br><br>What according to you causes anaemia?<br><br>आपके हिसाब से अनेमिया किस कारन से होता है?<br><br>**MULTIPLE CHOICE**	1=Not consuming Iron rich diet<br>आयरन युक्त खाना न खाने की वजह से<br>2=Not taking Iron supplementation during pregnancy<br>गर्भवस्था के दौरान आयरन की टेबलेट या आयरन की सिरप न लेने की वजह से<br>3=No interval between subsequent pregnancies<br>बच्चो के बीच में अंतर न होने की वजह से<br>4=Increased blood loss during periods<br>माहवारी में ज्यादा खून बहने से<br>5=Due to hook worm infestation<br>पेट में कीदें लग जाने पर<br>88=Others<br>अन्य<br>99= DO NOT KNOW<br>नहीं पता

**J18c**	What are the some of the food items which are rich in iron?<br><br>कुछ ऐसे खाने की चीज़ें बताईये जिनमें आयरन होता है?<br><br>**MULTIPLE CHOICE**	1=Dates/dry fruits<br>मेवे, खजूर, बादाम, मुमफली इत्यादि<br>2=Chicken/Mutton<br>मॉस- चिकन, मटन<br>3=Green leafy vegetables<br>हरी पट्टी वाली सब्जियां<br>4=Eggs<br>अन्दा<br>5=Milk<br>दूध<br>6=Cereals<br>अनाज<br>88=Others<br><br>99= DO NOT KNOW

I. MEDIA (RC1/2/3/4/5/6/7/8)

This section will be asked to all categories

Read out to the respondent: We've spent a lot of time talking about your experiences with your pregnancy of [CHILD NAME]. Now, I'm going to ask you specifically about where you hear and gather information related to your health and [CHILD NAME's] health from the media.
mÙkjnkrk dk i<dj lquk;s geu vHkh rd vkidh xHkkoLFkk d ckjs es ckr djus es dkQh le; fcrk;k gSA vc es vkils bl ckjs es ckr d:xh fd vki viu vkj viu cPp d LokLF; d ckjs es dgka ls tkudkjh Áklr djrs gs ;k lqurs gSA

I1.	Do you read newspaper(s) or magazine(s)?<br>क्या आप समाचार पत्र या पत्रिका पढ़ती हैं?	1 = YES हाँ<br>0 = NO नहीं → **GO TO I3**
I2.	Have you read any maternal or child or adolescent health related information/messages in newspapers/magazines in the past three months?<br>क्या पिछले तीन महीनों में आपने समाचार पत्र/पत्रिका मे माँ के स्वास्थ्य या बच्चे के स्वास्थ्य से सम्बन्धित कोई जानकारी/संदेश पढ़ा है?	1 = YES हाँ<br>0 = NO नहीं
I3.	Do you listen to the radio?<br>क्या आप रेडियो सुनती हैं?	1 = YES हाँ<br>0 = NO नहीं → **GO TO I5**
I4.	Have you heard any maternal or child or adolescent health related information/messages on radio in the past three months?<br>क्या पिछले तीन महीनों में आपने रेडियो मे माँ के स्वास्थ्य या बच्चे के स्वास्थ्य से सम्बन्धित कोई जानकारी/संदेश सुना है?	1 = YES हाँ<br>0 = NO नहीं
I5.	Do you watch television?<br>क्या आप टेलीविजन देखती हैं?	1 = YES हाँ<br>0 = NO नहीं → **GO TO I7**
I6.	Have you seen any maternal or child or adolescent health related information on television in the past three months?<br>क्या पिछले तीन महीनों में आपने टी.वी. पर माँ के स्वास्थ्य या बच्चे के स्वास्थ्य से सम्बन्धित कोई जानकारी/संदेश देखी है?	1 = YES हाँ<br>0 = NO नहीं
I7.	**ASK ONLY IF YES IN I2, I4, I6 AND RESPONDENT CATEGORY<>5**<br>;fn **I2, I4, I6** es gk¡ g] iNs<br>What messages related to maternal and child health have you read, hear or seen?<br>माँ के स्वास्थ्य या बच्चे के स्वास्थ्य से सम्बन्धित किन सन्देशों को आपने पढ़ा, सुना या देखा है?<br><br>**PROBE : ANY OTHERS?**<br>çksc % dqN vkSj\<br><br>**RECORD ALL MENTIONED**<br>lHkh mÙkj fy[ksA	1 = TAKING CARE OF YOUR HEALTH DURING PREGNANCY गर्भावस्था के दौरान अपने स्वास्थ्य की देखरेख करना<br>2 = GOING TO THE HOSPITAL FOR A CHECK-UP DURING PREGNANCY गर्भावस्था के दौरान जाँच के लिये अस्पताल जाना<br>3 = GOING TO A HEALTH FACILITY FOR DELIVERY प्रसव कराने के लिये स्वास्थ्य सुविधा में जाना<br>4 = PLANNING IN ADVANCE FOR DELIVERY प्रसव के लिये पहले से योजना बनाना<br>5 = HOW TO TAKE CARE OF THE NEWBORN नवजात शिशु की देखरेख कैसे करनी है<br>6 = BREASTFEEDING स्तनपान<br>7 = COMPLEMENTARY FEEDING उपर का खाना पूरक आहार<br>8 = IMMUNISATION टीकाकरण

		9 = FAMILY PLANNING परिवार नियोजन<br>10 = HANDWASHING AND SANITATION हाथ धोना और सफाई<br>12 = MENSTRUAL HYGINE मासिक धर्म स्वच्छता<br>13 = CONSUMPTION OF IFA TABLETS आई.एफ.ए. (ताकत की लाल गोली) की गोलियाँ खाना<br>14=Varieties and amounts of foods to eat during pregnancy<br>गर्भवस्था के दौरान भिन्न प्रकार का खाना और कितने मात्र में खाना<br>15=Amount of weight to gain during pregnancy<br>गर्भवस्था के दौरान कितना वजन भाधना<br>16=Placing baby on breast immediately after delivery<br>बच्चे को जन्म के तुरंत बाद स्तन पे लगाना<br>17=Exclusive breastfeeding, not even water in the first 6 months<br>जन्म के पहले ६ महीनो तक केवल माँ का दूध पिलाना<br>18=Not giving any fluids in the first 3 days after delivery<br>जन्म के पहले 3 दिन कोई भी तरल पदार्थ न देना<br>19=Amount of food and number of meals to feed children 6-23 months of age<br>६-23 महीने वाले बच्चे को कितने मात्र में और कितने बार खाना खिलाना व्हाहिये<br>20=Varieties of foods to feed children of 6-23 months of age<br>६-23 महीने के बच्चे को किस प्रकार का खाना देना चाहिए<br>21=Washing hands with soap before food preparation and feeding children<br>बच्चे को खाना खिलाने और खाना बनाने के पहले साबुन से हाथ धोना<br>88 = OTHER (SPECIFY) अन्य (स्पष्ट करें)
I8.	**ASK ONLY IF YES IN I2, I4, I6 AND RESPONDENT CATEGORY = 5**<br>;fn **I2, I4, I6** e gk g vkj mÙkjnkrk dk oxZ 5 g] iNs<br>What messages related to adolescent health have you read, hear or seen?<br>किशोरी स्वास्थ्य से सम्बन्धित किन सन्देशों को आपने पढ़ा, सुना या देखा है?<br><br>**PROBE: ANY OTHERS?**<br>çkc ¼ dN vkj\<br><br>**RECORD ALL MENTIONED**<br>lHkh mÙkj fy[kA	8 = IMMUNISATION टीकाकरण<br>9 = FAMILY PLANNING परिवार नियोजन<br>10 = HANDWASHING AND SANITATION हाथ धोना और सफाई<br>11 = DELAYED AGE AT MARRIAGE शादी मे देर करना<br>12 = MENSTRUAL HYGINE मासिक धर्म स्वच्छता<br>13 = CONSUMPTION OF IFA TABLETS आई.एफ.ए. (ताकत की लाल गोली) की गोलियाँ खाना<br>88 = OTHER (SPECIFY) अन्य (स्पष्ट करें)
I9.	Do you have a mobile phone?<br>क्या आपके पास मोबाईल फोन है?	1 = YES हाँ<br>0 = NO नहीं
I10.	Does anyone else in your household have a mobile phone?<br>क्या आपके घर के किसी अन्य सदस्य के पास मोबाईल फोन है?	1 = YES हाँ<br>0 = NO नहीं
I11.	**ASK IF YES IN I9,I10 ELSE GO TO I12**<br>;fn **I9,I10** e gk g] iNs] vU;Fkk **I12** ij tk;s<br><br>In the previous three months, have you heard about maternal and child health care on your mobile phone/through SMS messaging?	1 = YES हाँ<br>0 = NO नहीं

	क्या पिछले तीन महीनों में आपको मोबाईल फोन से/एस.एम.एस. के जरिये माँ के स्वास्थ्य या बच्चे के स्वास्थ्य से सम्बन्धित कोई जानकारी/संदेश मिली है?	
I12.	In the previous 3 months have you seen any drama, community event, puppet shows, school celebrations, nukkad natak, video shows, festival celebrations or street performance that included messages on maternal and child health?क्या पिछले तीन महीनों में आपने माँ के स्वास्थ्य या बच्चे के स्वास्थ्य से सम्बन्धित कोई नुकड़ नाटक, कठपुतली नाटक, विडियो शोज, या किसी प्रकार का सेलिब्रेशन देखा है?	1 = YES हाँ<br>0 = NO नहीं
I13.	Have you ever seen this logo?<br>क्या आपने यह प्रतीक चिन्ह देखा है?<br>**SHOW THE LOGO OF THE HAUSLA CAMPAIGN.**<br>gkl yk vfHk;ku dk çrhd fpUg n[kk;A	1 = YES हाँ<br>0 = NO नहीं
I14.	Have you heard of the Hausla campaign with the tagline '*mujhse bada hai mera hausla*'?<br>क्या आपने हौसला अभियान जिसका टेगलाइन है "मुझसे बड़ी है मेरा हौसला" के बारे सुना है?	1 = YES हाँ<br>0 = NO नहीं
I15.	Have you watched the serial '*Main kuch bhi kar sakti hoon'* on TV?<br>**क्या आपने 'मैं कुछ भी कर सकती हूँ' सीरियल देखा है?**	1 = YES हाँ<br>0= NO नहीं

**M. ACCESS TO GOVT SCHEME AND FACILITIES (RC1/2/3/4/5/6/7/8)**<br>This section will be asked to all categories<br>***Read out to the respondent: Now I would like to ask you about the access of this family to Government schemes and facilities.***<br>mÙkjnkrk dk i< dj lquk;s % vc eS ljdkj }kjk pyk;h tk jgh ;kstukvksa vkj lqfo/kkvksa rd vkidh ifjokj dh igqp d s ckjs esa iNwxhA		
M1.	Does any member of this household have a bank account or post-office account?<br>क्या इस परिवार के किसी भी सदस्य का बैंक या पोस्ट आफिस में खाता है?	1 = YES हाँ<br>0 = NO नहीं<br>99 = DO NOT KNOW पता नहीं
M1a	Do you have a bank account or post-office account?<br>क्या आपका बैंक या पोस्ट आफिस में खाता है?	1 = YES हाँ<br>0 = NO नहीं<br>99 = DO NOT KNOW पता नहीं
M1b	Do you have an AADHAR card?<br>क्या आपका आधार कार्ड है?	1 = YES हाँ<br>0 = NO नहीं<br>99 = DO NOT KNOW पता नहीं
M2.	Does this household have a BPL card/coupon?<br>क्या इस परिवार के पास गरीबी रेखा (बी. पी. एल.) कार्ड/कूपन है?<br>**DESCRIBE BPL FOR CLARITY**<br>ch-ih-,y- ds ckjs esa crk;sA	1 = YES, CARD SEEN हाँ, कार्ड देखा<br>2 = YES, CARD NOT SEEN हाँ, कार्ड नहीं देखा<br>0 = NO CARD कार्ड नहीं है<br>99 = DO NOT KNOW पता नहीं
M3.	Does this household have an Antodaya card/coupon?<br>क्या इस परिवार के पास अन्तोदाये कार्ड/कूपन है?<br>**DESCRIBE BPL FOR CLARITY**<br>ch-ih-,y- ds ckjs esa crk;sA	1 = YES, CARD SEEN हाँ, कार्ड देखा<br>2 = YES, CARD NOT SEEN हाँ, कार्ड नहीं देखा<br>0 = NO CARD कार्ड नहीं है<br>99 = DO NOT KNOW पता नहीं
M4.	Does this household have a RSBY card?<br>क्या इस परिवार के पास राष्ट्रीय स्वास्थ्य बीमा योजना कार्ड है?<br>**DESCRIBE RSBY FOR CLARITY**<br>jk"Vªh; LokLF; chek ;kstuk ds ckjs esa crk;sA	1 = YES, CARD SEEN हाँ, कार्ड देखा<br>2 = YES, CARD NOT SEEN हाँ, कार्ड नहीं देखा<br>0 = NO CARD कार्ड नहीं है<br>99 = DO NOT KNOW पता नहीं
M5.	Does this household have a MGNREGA card?<br>क्या इस परिवार के पास मगाँनरेगा का जाब कार्ड है?<br>**DESCRIBE NREGA FOR CLARITY**<br>egkRek xka/kh jk"Vªh; xzkeh.k jkstxkj xkjaVh vf/kfu;e ds ckjs esa crk;sA	1 = YES, CARD SEEN हाँ, कार्ड देखा<br>2 = YES, CARD NOT SEEN हाँ, कार्ड नहीं देखा<br>0 = NO CARD कार्ड नहीं है<br>99 DO NOT KNOW पता नहीं

**O. SOCIAL PARTICIPATION IN GROUPS (RC1/2/3/4/6/7/8)**<br>***Read out to the respondent: Now I would like to talk to you about groups in your community.***<br>mÙkjnkrk dk i<dj lquk;s% vc eS vkils vkids lenk; ds leqg ;k lxBuksa ds lEcU/k esa iNuk pkgwxhA			
O1.	Do you know of any social groups in your area?<br>क्या आप अपने क्षेत्र में मौजूद किसी सामाजिक समूह के बारे में जानती हैं?	1 = YES हाँ<br>0 = NO नहीं	
O2.	Are these groups there in your area?	YES हाँ	NO नहीं

	क्या यह समूह आपके क्षेत्र में हैं?		
a.	Village health and sanitation committee ग्राम स्वास्थ्य और स्वच्छता समिति	1	0
b.	Reproductive health group प्रजनन स्वास्थ्य समूह	1	0
c.	Rogi kalyan samiti रोगी कल्यान समिति	1	0
d.	Women's/mothers groups महिलाओं/माताओं के समूह	1	0
e.	Self-help group स्वयं सहायता समूह	1	0
f.	Mahila Mandal महिला मंडल	1	0
g.	Savings group बचत समूह	1	0
**O2a1**	**<u>ASK IF YES IN OPTION g OF O2</u>**<br>Are you a member of any savings group in your area?<br>क्या आप अपने क्षेत्र की किसी **बचत समूह** की सदस्य हैं?	1 = YES हाँ<br>0= NO नहीं. GOTO O3	
**O2a2**	Since when have you been a member of this group?<br>**आप कबसे इस समूह के सदस्य है?**	☐☐ NO. OF MONTHS<br>99 = DO NOT REMEMBER याद नह	
**O2a3**	How often do you save through this group?<br>**आप कब कब इस समूह में पैसे रखते है?**	1= MONTHLY<br>2=EVERY 3 MONTHS<br>3=EVERY 6 MONTHS<br>4=YEARLY<br>99 = DO NOT REMEMBER याद नह	
**O2a4**	How many times have you taken a loan from this group?<br>**अब तक कितनी बार इस समूह से आपने लोन लिया?**	☐☐ NO. OF MONTHS<br>99 = DO NOT REMEMBER याद नह	
O3.	**<u>ASK IF YES IN a-f OPTION OF O2</u>**<br>Are you a member of any mahila mandal in your area?<br>क्या आप अपने क्षेत्र की किसी महिला मंडल की सदस्य हैं?	1 = YES हाँ<br>0= NO नहीं	
**O3A**	Is any member of your household a member of any mahila mandal in your area?<br>क्या आपके परिवार में कोई इस क्षेत्र की किसी महिला मंडल की सदस्य ह ?<br>**IF O3=0 and O3A=0, SKIP TO O11**	1 = YES हाँ<br>0 = NO नहीं	
**O3B**	**ASK ONLY IF O3=0 and O3A=1, SKIP to O11 AFTER ANSWERING THIS QUESTION**<br>Did the household member mention about discussing any health messages in the Mahila Mandal?<br>क्या उस परिवार सदस्य ने आपको कभी बताया के महिला मंडल में स्वस्थ्य सम्बंधित चर्चा होती है?	1 = YES हाँ<br>0= NO नहीं→ **GO TO O11**	
**O3C**	Has that member ever communicated any health messages to you which were discussed in the Mahila Mandal?<br>क्या उस परिवार के सदस्य ने महिला मंडल में चर्चा की जाने वाली स्वस्थ्य सम्बंधित चीज़ों के बारें में बताया?	1 = YES हाँ<br>0= NO नहीं	
O4.	**ASK O4 ONWARDS ONLY IF O3=1**<br>Have you participated in or attended any mahila mandal meeting in the past three months?<br>क्या आपने पिछले तीन महीनों में इस तरह की महिला मंडल की बैठक में भाग लिया है?	1 = YES हाँ<br>0 = NO नहीं → **GO TO O11**	
O5.	Are maternal and infant health topics discussed in the meeting(s)?<br>क्या इन बैठकों में माताओं और शिशुओं के स्वास्थ्य के मुददों पर चर्चा होती है?	1 = YES हाँ<br>0 = NO नहीं → **GO TO O7**<br>9 = DO NOT KNOW पता नहीं → **GO TO O7**	
O6.	Which heath topics are discussed in the meeting(s)?<br>इन बैठकों में किन स्वास्थ्य सम्बन्धी विष्यों पर विचार विमर्श किया जाता है?<br><br>**PROBE : ANY OTHERS?**<br>çkc ¶ dN vkj\<br><br>**RECORD ALL MENTIONED**<br>lHkh mÙkj fy[kA	1 = WHAT TO DO IF A PREGNANT WOMEN HAS AN EMERGENCY अगर कोई गर्भवती महिला संकट की स्थिति में हो तो क्या किया जाये<br>2 = ANTENATAL CARE OF MOTHERS माताओं की प्रसव–पूर्व देखरेख<br>3 = PREPARATION FOR DELIVERY प्रसव की तैयारी<br>4 = HEALTHCARE FACILITY SERVICES स्वास्थ्य देखरेख, सुविधा सेवायें<br>5 = JSY, JSSK, OR OTHER SCHEME जेएसवाई, जेएसएसके, और अन्य योजनायें	

		6 = CLEAN AND SAFE DELIVERIES स्वाच्छ और सुरक्षित प्रसव<br>7 = POSTNATAL CARE OF MOTHERS माताओं की प्रसवोत्तर देखरेख<br>8 = WHAT TO DO WHEN NEWBORN BABIES HAVE EMERGENCIES नवजात शिशु के संकट की स्थिति में होने पर क्या करना है<br>9 = HOW TO KEEP BABIES HEALTHY शिशुओं को स्वस्थ्य कैसे रखना है<br>10 = BREASTFEEDING स्तनपान<br>11 = COMPLEMENTARY FEEDING पूरक आहार<br>12 = IMMUNÁATIONS टीकाकरण<br>13 = FAMILY PLANNING परिवार नियोजन<br>14=Varieties and amounts of foods to eat during pregnancy<br>गर्भवस्था के दौरान भिन्न प्रकार का खाना और कितने मात्र में खाना<br>15=Amount of weight to gain during pregnancy<br>गर्भवस्था के दौरान कितना वजन भाधना<br>16=Placing baby on breast immediately after delivery<br>बच्चे को जन्म के तुरंत बाद स्तन पे लगाना<br>17=Exclusive breastfeeding, not even water in the first 6 months<br>जन्म के पहले ६ महीनो तक केवल माँ का दूध पिलाना<br>18=Not giving any fluids in the first 3 days after delivery<br>जन्म के पहले 3 दिन कोई भी तरल पदार्थ न देना<br>19=Amount of food and number of meals to feed children 6-23 months of age<br>६-23 महीने वाले बच्चे को कितने मात्र में और कितने बार खाना खिलाना व्हाहिये<br>20=Varieties of foods to feed children of 6-23 months of age<br>६-23 महीने के बच्चे को किस प्रकार का खाना देना चाहिए<br>21=Washing hands with soap before food preparation and feeding children<br>बच्चे को खाना खिलाने और खाना बनाने के पहले साबुन से हाथ धोना<br>22 = SANITATION/HYGIENE स्वच्छता और साफ सफाई<br>88 = OTHER (SPECIFY) अन्य (स्पष्ट करें)
O7.	How often does the group meet for discussions or activities?<br>विचार–विमर्श या कार्यकलाप के लिए समूह की बैठक कब–कब होती है?	☐ ☐☐<br>1 = MONTH महीना<br>2 = YEAR साल<br>98 = NOT DECIDED तय नहीं है
O8.	How many times did you attend the meeting in the last three months?<br>पिछले तीन महीनों मे आपने कितनी बार इन बैठकों मे भाग लिया था?	☐ NO. OF TIMES कितनी बार
O9.	Do front line health workers including AWWs, ASHAs and ANMs attend the meetings?<br>क्या आँगनवाड़ी कार्यकर्ता, आशा और ए.एन.एम. इन बैठकों में भाग लेते हैं?<br><br>**PROBE : ANY OTHERS?**<br>çkc ¼ dN vkj\	1 = AWW आँगनवाड़ी कार्यकर्ता<br>2 = ASHA आशा<br>3 = ANM ए.एन.एम.<br>9 = NO नहीं

	**RECORD ALL MENTIONED**<br>lHkh mÙkj fy[kA	
O10.	Do community leaders like pradhans, panchayat members/opinion makers attend these meetings?<br>क्या समुदाय के नेता जैसे प्रधान, पंचायत के सदस्य/राय बनाने वाले इन बैठकों में भाग लेते हैं?	1 = YES हाँ<br>0 = NO नहीं
O11.	<u>**ASK IF YES IN ANY OPTION OF O2**</u><br>Are you a member of any Self-Help Group in your area?<br>क्या आप किसी स्वयं सहायता समूह की सदस्य हैं?	1 = YES हाँ<br>0 = NO नहीं
O11A	Is any member from your family a member of any SHG in your area?<br>क्या आपके परिवार में कोई इस क्षेत्र की किसी स्वयं सहायता समूह की सदस्य हैं?<br>**IF O11=0 and O11A=0, SKIP TO S1**	1 = YES हाँ<br>0= NO नहीं
**O11B**	**ASK ONLY IF O3=0 and O3A=1, SKIP to O11 AFTER ANSWERING THIS QUESTION**<br>Did the household member mentioned about discussing any health messages in the SHG?<br>क्या उस परिवार सदस्य ने आपको कभी बताया के समूह में स्वस्थ्य सम्बंधित चर्चा होती है?	1 = YES हाँ<br>0= NO नहीं→ **GO TO S1**
**O11C**	Has that member ever communicated any health messages to you which were discussed in the SHG?<br>क्या उस परिवार के सदस्य ने समूह में चर्चा की जाने वाली स्वस्थ्य सम्बंधित चीज़ों के बारें में बताया?	1 = YES हाँ<br>0= NO नहीं
O12.	**ASK O12 ONWARDS ONLY IF O11=1**<br>Have you participated in or attended any Self-Help Group meeting in the past three months?<br>क्या आपने पिछले तीन महीनों में स्वयं सहायता समूह की किसी बैठक में भाग लिया है?	1 = YES हाँ<br>0 = NO नहीं → **GO TO S1**
O13.	Does the group collect membership fees, not including self-help group contributions?<br>क्या यह समूह सदस्यों से स्वयं सहायता समूह के योगदान के अतिरिक्त सदस्यता शुल्क एकत्र करता है?	1 = YES हाँ<br>0 = NO नहीं
O14.	Are maternal and infant health topics discussed in the meeting(s)?<br>क्या इन बैठकों में माताओं और शिशुओं के स्वास्थ्य के मुददों पर चर्चा होती है?	1 = YES हाँ<br>0 = NO नहीं → **GO TO O16**<br>9 = DO NOT KNOW पता नहीं → **GO TO O16**
O15.	Which heath topics are discussed in the meeting(s)?<br>इन बैठकों में किन स्वास्थ्य सम्बन्धी विष्यों पर विचार विमर्श किया जाता है?<br><br>**PROBE : ANY OTHERS?**<br>çkc s dN vkj\<br><br>**RECORD ALL MENTIONED**<br>lHkh mÙkj fy[kA	1 = WHAT TO DO IF A PREGNANT WOMEN HAS AN EMERGENCY अगर कोई गर्भवती महिला संकट की स्थिति में हो तो क्या किया जाये<br>2 = ANTENATAL CARE OF MOTHERS माताओं की प्रसव–पूर्व देखरेख<br>3 = PREPARATION FOR DELIVERY प्रसव की तैयारी<br>4 = HEALTHCARE FACILITY SERVICES स्वास्थ्य देखरेख, सुविधा सेवायें<br>5 = JSY, JSSK, OR OTHER SCHEME जेएसवाई, जेएसएसके, और अन्य योजनायें<br>6 = CLEAN AND SAFE DELIVERIES स्वाच्छ और सुरक्षित प्रसव<br>7 = POSTNATAL CARE OF MOTHERS माताओं की प्रसवोत्तर देखरेख<br>8 = WHAT TO DO WHEN NEWBORN BABIES HAVE EMERGENCIES नवजात शिशु के संकट की स्थिति में होने पर क्या करना है<br>9 = HOW TO KEEP BABIES HEALTHY शिशुओं को स्वस्थ्य कैसे रखना है<br>10 = BREASTFEEDING स्तनपान<br>11 = COMPLEMENTARY FEEDING पूरक आहार<br>12 = IMMUNÁATIONS टीकाकरण

		13 = FAMILY PLANNING परिवार नियोजन<br>14 = SANITATION/HYGIENE स्वच्छता और साफ सफाई<br>88 = OTHER (SPECIFY) अन्य (स्पष्ट करें)
O16.	How often does the group meet for discussions or activities?<br>विचार–विमर्श या कार्यकलाप के लिए समूह की बैठक कब–कब होती है?	☐ ☐☐<br>1 = MONTH महीना<br>2 = YEAR साल<br>98 = NOT DECIDED तय नहीं है
O17.	How many times did you attend the meeting in the last three months?<br>पिछले तीन महीनों मे आपने कितनी बार इन बैठकों मे भाग लिया था?	☐ NO. OF TIMES कितनी बार
O18.	Do front line health workers including AWWs, ASHAs and ANMs attend the meetings?<br>क्या आँगनवाड़ी कार्यकर्ता, आशा और ए.एन.एम. इन बैठकों में भाग लेते हैं?<br><br>**PROBE : ANY OTHERS?**<br>çkc ½ dN vkj\<br><br>**RECORD ALL MENTIONED**<br>lHkh mÙkj fy[kA	1 = AWW आँगनवाड़ी कार्यकर्ता<br>2 = ASHA आशा<br>3 = ANM ए.एन.एम.<br>9 = NO नहीं
O19.	Do community leaders like pradhans, panchayat members/opinion makers attend these meetings?<br>क्या समुदाय के नेता जैसे प्रधान, पंचायत के सदस्य/राय बनाने वाले इन बैठकों में भाग लेते हैं?	1 = YES हाँ<br>0 = NO नहीं

**S.DECISION MAKING, SELF-EFFICACY AND WOMEN EMPOWERMENT**<br>फैसला लेना, स्व–क्षमता और महिला सशक्तीकरण<br>This section will be asked to all categories except adolescent girls of 10-19 years			
Now, I would like to ask few questions about key decisions that are taken in your household regarding health care, children or household purchases.<br>अब मैं आपसे स्वास्थ्य देखरेख, बच्चों और घरेलू खरीददारियों के संबंध में आपके परिवार में लिये जानेवाले मुख्य फैसलों के बारे में कुछ सवाल पूछना चाहूंगा।			
S19.	Who in your household usually makes decisions about healthcare for yourself? Would you say yourself, your partner or both equally or any other?<br>आपके परिवार में घर की महिलाओं के स्वास्थ्य के बारे में अंतिम फैसला किसका होता है? आप लेती हैं, आपके पति लेते है या दोनों समान रूप से लेते हैं या कोई अन्य लेता है।	1=SELF<br>स्वयं<br>2=HUSBAND<br>पति<br>3=BOTH<br>दोनों<br>4=OTHERS<br>अन्य	
S20.	Who in your household usually makes decisions about your children (their schooling, their activities)? Would you say yourself, your partner or both equally or any other?<br>आपके परिवार में आपके बच्चों के संबंध में (यानी उनके स्कूल में पढ़ाने, अन्य कार्यकलापों के बारे में) अंतिम फैसला कौन लेता है? आप लेती हैं, आपके पति लेते है या दोनों समान रूप से लेते हैं या कोई अन्य लेता है।	1=SELF<br>स्वयं<br>2=HUSBAND<br>पति<br>3=BOTH<br>दोनों<br>4=OTHERS<br>अन्य<br>77=NOT APPLICABLE<br>लागू नहीं	
S21.	Who in your household usually makes decisions about how your family spends money on food and clothing? Would you say yourself, your partner or both equally or any other?<br>आपके परिवार में भोजन और कपड़ों पर पैसा खर्च करने के संबंध में अंतिम फैसला कौन लेता है? आप लेती हैं, आपके पति लेते है या दोनों समान रूप से लेते हैं या कोई अन्य लेता है।	1=SELF<br>स्वयं<br>2=HUSBAND<br>पति<br>3=BOTH<br>दोनों<br>4=OTHERS<br>अन्य	
S22.	Who usually makes decisions about major household purchases? Would you say yourself, your partner or both equally or any other?	1=SELF<br>स्वयं<br>2=HUSBAND<br>पति<br>3=BOTH	

	कार या घर या फिर घर के सामान जैसी महंगी चीजों पर पैसा खर्च करने के बारे में अंतिम फैसले कौन लेता है? आप लेती हैं, आपके पति लेते है या दोनों समान रूप से लेते हैं या कोई अन्य लेता है।	दोनों<br>4=OTHERS<br>अन्य				
S22 A	Who usually makes decisions about how money that you earn is used? Would you say yourself, your partner or both equally or any other?<br>आपकी कमाई हुई आय को कैसे खर्च करना है,इसके बारे में अंतिम फैसले कौन लेता है? आप लेती हैं, आपके पति लेते है या दोनों समान रूप से लेते हैं या कोई अन्य लेता है।	1=SELF<br>स्वयं<br>2=HUSBAND<br>पति<br>3=BOTH<br>दोनों<br>4=OTHERS<br>अन्य<br>77=NOT APPLICABLE<br>लागू नहीं				
S22 B	Are you usually allowed to go to the following places alone, only with someone else, or not at all?<br>क्या आपको निम्नलिखित स्थानों पर अकेले जाने दिया जाता है, या किसी और के साथ या बिल्कुल भी नहीं जाने दिया जाता है?	ALONE<br>अकेले	WITH SOMEONE ELSE ONLY<br>केवल किसी और के साथ	NOT AT ALL<br>बिल्कुल भी नहीं जाने दिया जाता		
	A.Health centre or clinic स्वास्थ्य केन्द्र या क्लीनिक	1	2	3		
	B.Places outside of community अपने समुदाय के बाहर की जगह	1	2	3		
	C.To the market बाजार जाना	1	2	3		
S23 A	Sometimes a husband is annoyed or angered by things that his wife does. In your opinion, is a husband justified in hitting or beating his wife in these situations?<br>कभी कभी एक पति उसकी पत्नी के कीये गये व्यवहार से नाराज या गुस्सा हो सकता है। क्या आपके विचार मे एक पति को ऐसे स्थितियों में उसकी पत्नी को मारना उचित है?	YES हाँ		NO नहीं		
1	If she goes out without telling him? यदि वह बिना बताये बाहर चली जाती है?	1		0		
2	If she neglects the house or the children? यदि वह घर और बच्चों की ठीक से देखभाल नहीं करती है?	1		0		
3	If she argues with him? यदि वह पति से बहस करती है?	1		0		
4	If she refuses to have sex with him? यदि वह यौन संबंध के लिए मना कर दिया?	1		0		
5	If she doesn't cook the food properly? यदि वह खाना बनाने के लिए मना करती है?	1		0		
6	If he suspects her of being unfaithful? यदि उसके पति को शक होता है कि उसको वह बिना बताये बाहर चली जाती है?	1		0		
7	If she shows disrespect for her in-laws? यदि वह सास/ससुर का अनादर करती है?	1		0		
S24.	Not including any help you receive from others, how do you and your husband divide the following tasks:<br>दूसरों से मिलने वाली मदद को छोड़ दें तो आप और आपका पति निम्नलिखित कामों का बंटवारा कैसे करते हैं:	SELF<br>अपने आप	HUSBAND<br>पति	JOINTLY<br>एक साथ	Others do this work<br>ये काम कोई और करता है	S24A.<br>If s24=2 or s24=3 ask else skip to s25<br>If so, how often did your husband do the following tasks for your family in past 30

						days?<br>अगर हाँ तोह पिछले ३० दिन में कब कब आपके पति ने ऐसा किया होगा?
a.	Preparing food<br>खाना पकाना	1	2	3	77	1=DAILY<br>रोजाना<br>2=MOSTLY<br>ज्यादातर<br>3=SOMETIMES<br>कभी कभी
b.	Cleaning the house<br>घर की सफाई	1	2	3	77	1=DAILY<br>रोजाना<br>2=MOSTLY<br>ज्यादातर<br>3=SOMETIMES<br>कभी कभी
c.	Washing clothes<br>कपड़े धोना	1	2	3	77	1=DAILY<br>रोजाना<br>2=MOSTLY<br>ज्यादातर<br>3=SOMETIMES<br>कभी कभी
d.	Taking care of the children<br>बच्चों की देखभाल करना	1	2	3	77	1=DAILY<br>रोजाना<br>2=MOSTLY<br>ज्यादातर<br>3=SOMETIMES<br>कभी कभी
S25.	Has your husband ever talked to you about what to use or do to prevent or stop a pregnancy?<br>क्या आपके पति ने कभी आपसे इस बारे में बात की है कि गर्भधारण को रोकने के लिए किसका उपयोग करना है या क्या करना है?	1=YES<br>हां<br>0=NO<br>नहीं				
S26.	In the past one year were you afraid of your (last) husband: most of the time, sometimes or never?<br>पिछले एक साल में क्या आप अपने पति (पिछले पति) से अधिकतर समय, कभी–कभी या कभी नहीं डरती हैं (थीं)?	1= MOST OF THE TIMES<br>ज्यादातर समय<br>2= SOMETIMES<br>कभी–कभी<br>3= NEVER<br>कभी नहीं				
S27.	Does (did) your husband drink alcohol?<br>क्या आपका पति शराब पीता है (था)?	1=YES<br>हां<br>0=NO **GO TO Error! Reference source not found.**<br>नहीं एस23 पर जायें				
S28.	How often does (did) he get drunk: often, only sometimes, or never?<br>वह कब–कब शराब पीकर होश खो देता है: अक्सर, कभी–कभी, कभी नहीं?	1= ALWAYS<br>हमेशा<br>2=OFTEN<br>अक्सर<br>3=SOMETIMES<br>कभी–कभी<br>4=NEVER<br>कभी नहीं				

S29.	Has your husband ever prohibited you from getting a job, going to work, trading, earning money or participating in income generation projects?<br>क्या आपके पति ने कभी आपको नौकरी पाने, काम पर जाने, व्यापार करने, पैसा कमाने, आय अर्जित करने वाले काम में भाग लेने से रोका है?	1=YES<br>हां<br>0=NO<br>नहीं	**ASK IF YES IN S29**<br>तभी पूछें जब प्र. 29 का उत्तर "हां" हो<br>S30. How often did this happen during the last 12 months: often, only sometimes, or not at all?<br>पिछले बारह महीनों में ऐसा कब–कब हुआ है?<br>1=OFTEN<br>अक्सर<br>2=SOMETIMES<br>कभी–कभी<br>3=NEVER<br>कभी नहीं	
S31.	Has your husband ever taken your earnings from you?<br>क्या आपके पति ने कभी आपसे आपकी कमाई ली है?	1=YES<br>हां<br>0=NO<br>नहीं	**ASK IF YES IN S31**<br>तभी पूछें जब प्र. 31 का उत्तर "हां" हो<br>S32. How often did this happen during the last 12 months: often, only sometimes, or not at all?<br>पिछले बारह महीनों में ऐसा कब–कब हुआ है?<br>1=OFTEN<br>अक्सर<br>2=SOMETIMES<br>कभी–कभी<br>3=NEVER<br>कभी नहीं	
S33.	Has your husband ever thrown you or your children out of the house where you were living?<br>क्या आपके पति ने कभी आपको या आपके बच्चों को उस घर से बाहर निकाला है जहां आप रहती थीं?	1=YES<br>हां<br>0=NO<br>नहीं	**ASK IF YES IN S33**<br>तभी पूछें जब प्र. 33 का उत्तर "हां" हो<br>S34. How often did this happen during the last 12 months: often, only sometimes, or not at all?<br>पिछले बारह महीनों में ऐसा कब–कब हुआ है?<br>1=OFTEN<br>अक्सर<br>2=SOMETIMES<br>कभी–कभी<br>3=NEVER<br>कभी नहीं	
S35.	Has your husband ever refused to give you money you needed for household expenses even when he has money for other things?<br>क्या आपके पति ने कभी दूसरी चीजों के लिए पैसा होने पर भी घर की जरूरत के लिए आपको पैसा देने से मना किया है?	1=YES<br>हां<br>0=NO<br>नहीं	**ASK IF YES IN S35**<br>तभी पूछें जब प्र. 35 का उत्तर "हां" हो<br>S36. How often did this happen during the last 12 months: often, only sometimes, or not at all?<br>पिछले बारह महीनों में ऐसा कब–कब हुआ है?<br>1=OFTEN<br>अक्सर<br>2=SOMETIMES<br>कभी–कभी<br>3=NEVER<br>कभी नहीं	

VIOLENCE AGAINST WOMEN
महिलाओं के विरुद्ध हिंसा

Now I would like to ask you questions about violence that you might be experiencing in your household which might be perpetrated by your husband or other members of your household.

अब मैं आपसे कुछ प्रश्न पूछूंगी जिनका संबंध आपके पति या परिवार के अन्य सदस्यों द्वारा की जाने वाली उस हिंसा से है जिसका हो सकता है आप सामना कर रही हों।

I know that some of these questions are very personal. However, your answers are crucial for helping to understand the condition of women in India. Let me assure you that your answers are completely confidential and will not be told to anyone and no one else will know that you were asked these questions.

मैं जानती हूं कि इनमें से कुछ सवाल व्यक्तिगत हैं। पर भारत में महिलाओं की स्थिति को समझने के लिए आपके उत्तर महत्वपूर्ण है। मैं आपको यह आश्वासन देती हूं कि आपके उत्तर गोपनीय रखे जायेंगे और किसी को भी यह पता नहीलं चलेगा कि आपसे ये प्रश्न पूछे गये थे।

S37.	First, I am going to ask you about some situations which happen to some women. Please tell me if these apply to your relationship with your (last) husband.<br>सबसे पहले मैं आपसे ऐसी घटनाओं के बारे में पूछूंगी जो किसी भी महिला के साथ घट सकती हैं। कृपया मुझे यह बतायें कि क्या ये आपके पति (पिछले पति) के साथ आपके संबंध पर लागू होती हैं।	YES<br>हां	NO<br>न्हीं	DK<br>मालूम नहीं	
a.	He (does/did) not permit you to meet your female friends.<br>वह आपको अपनी सहेलियों से मिलने की अनुमति नहीं देता है/देता था।	1	0	8	
b.	He (tries/tried) to limit your contact with your family<br>वह आपके परिवार के साथ आपके संपर्क को कम करता है।	1	0	8	
c.	He (does/did) not trust you with any money.<br>वह आपको कोई पैसे देने में विश्वास नहीं करता है.	1	0	8	
d.	He (insists/insisted) on knowing where you (are/were) at all times.<br>वह यह जानने पर जोर देता है/देता था कि आप पूरे समय कहां रहीं/थीं।	1	0	8	
e.	He frequently (accuses/accused) you of being unfaithful.<br>वह बार–बार आप पर बेवफा होने का आरोप लगाता है।	1	0	8	
f.	He (is/was) jealous or angry if you (talk/talked) to other men.<br>अगर आप किसी दूसरे पुरुष से बात करती हैं/करती थीं तो उसे जलन होती है/होती थी	1	0	8	
g.	He (ignores/ignored) you or (treats/treated) you indifferently<br>वह आपकी उपेक्षा करता है/करता था या आपके साथ उदासीनता का व्यवहार करता है/करता था।	1	0	8	
h.	He (expects/expected) you to seek permission before seeking health care for herself<br>वह आपसे यह उम्मीद करता है/करता था कि आप अपनी स्वास्थ्य देखरेख प्राप्त करने के लिए उससे इजाजत लेंगी।	1	0	8	
i.	He (does/did) not permit you to work outside your home.<br>वह आपको आपके घर के बहार किसी भी प्रकार का काम, जिससे आमदनी हो, वोह करने नहीं देते	1	0	8	
j.	He (does/did) not permit you to use contraceptives.<br>वह आपको 117कोई परिवार नियोजन की पद्धति का प्रयोग करने नहीं देते	1	0	8	
S38.	Now if you will permit me, I need to ask some more questions about your relationship with your (last) husband.<br>(Does/did) your (last) husband ever:<br>अगर आप मुझे इजाजत दें तो मैं आपके (पिछले) पति के साथ आपके संबंध के बारे में कुछ और सवाल पूछूंगी।<br>(क्या आपके (पिछले) पति ने कभी:	YES<br>हां	NO<br>नहीं	**ASK IF YES IN S38**<br>तभी पूछें जब प्र. 38 का उत्तर हां तो<br>S39.<br>Did this happen during the last 12 months?<br>एस41.क्या पिछले 12 महीनों में यह हुआ: ?<br><br>1= YES	

				हाँ<br>0= NO<br>नहीं	
a.	Say or do something to humiliate/insult you in front of others?<br>दूसरों के सामने आपकी बेइज्जती करने के लिए कुछ कहा या किया है?	1	0	|___|	
b.	Threaten to hurt or harm you or someone close to you?<br>आपको या आपके किसी नजदीकी व्यक्ति को चोट या नुकसान पहुंचाने की धमकी दी?	1	0	|___|	
c.	Insult you or make you feel bad about yourself?<br>आपकी बेइज्जती की या बुरा महसूस करवाया?	1	0	|___|	
d.	Scare or intimidate you on purpose for example, by the way he looks at you, by yelling or smashing things?<br>जानबूझ कर आपको डराया या धमकाया; उदाहरण के लिए आपको देखने के अपने तरीके से, आप पर चिल्ला कर या चीजों की तोड़फोड़ करके?	1	0	|___|	
e.	Hurt people you care about as a way of hurting you or damaging things of importance to you?<br>आपको चोट पहुंचाने के लिए उन लोगों को चोट पहुंचाई जिनकी आप परवाह करती या फिर आपके लिउ महत्वपूर्ण चीजों को तोड़ा–फोड़ा।	1	0	|___|	
S40.	(Does/did) your (last) husband ever do any of the following things to you:<br>क्या आपके (पिछले) पति ने निम्नलिखित में से कुछ किया था?	YES<br>हां	NO<br>नहीं	**ASK IF YES IN S40**<br>तभी पूछें जब प्र. एस40 का उत्तर "हां" हो<br>S41. Did this happen during the last 12 months?<br>एस41.क्या पिछले 12 महीनों में यह हुआ: ?<br>1= YES<br>ळां<br>0= NO<br>नहीं	
a.	Slap you?<br>आपको थप्पड़ मारा	1	0	|___|	
b.	Twist your arm or pull your hair?<br>आपके बांह मरोड़ी, बाल खींचे?	1	0	|___|	
c.	Push you, shake you or throw something at you?<br>धक्का दिया, झकझोरा, आप पर कुछ उठा कर फेंका?	1	0	|___|	
d.	Kick you, drag you or beat you up?<br>लात मारी, घसीटा और पिटाई की?	1	0	|___|	
e.	Try to choke you or burn you?<br>गला घोंटने, जलाने की कोशिश की?	1	0	|___|	
f.	Punch you with his fist or with something that could hurt you?<br>मुक्का मारा या किसी और ऐसी चीज से जो आपको चोट पहुंचा सकती थी?	1	0	|___|	
g.	Threaten or attack you with a knife, gun or any other weapon?<br>चाकू, बंदूक या किसी और हथियार से आप पर हमरा करने की धमकी दी।	1	0	|___|	
h.	Physically force you to have sexual intercourse with him even when you did not want to?<br>आपको शरीरिक रूप से यौन संपर्क करने के लिए मजबूर किया जबकि आप नहीं चाहती थी?	1	0	|___|	
i.	Physically force you to perform any other sexual acts you did not want to?	1	0	|___|	

	आपको शारीरिक रूप से ऐसे यौन कार्य करने के लिए मजबूर किया जो आप नहीं चाहती थीं।				
j.	Force you with threats or in any other way to perform sexual acts you did not want to?<br>आपको धमकी देकर या किसी और तरीके से ऐसे यौन कार्य करने के लिए मजबूर किया जो आप पहीं चाहती थी?	1	0	|___|	
k.	Had sexual intercourse when you did not want to because you were afraid of what your husband might do?<br>आपके न चाहने पर भी आपके साथ इसलिए यौन संपर्क किया क्योंकि आपको डर था कि आपका पति कुछ कर सकता है?	1	0	|___|	
l.	Force you to do something sexual that you found degrading or humiliating?<br>आपको ऐसा यौन कार्य करने के लिए मजबूर किया, जो आपको अपमानजनक लगा।	1	0	|___|	

S4	**ASK ONLY IF YES IN S37 OR S38 OR S40 ELSE GO TO S44**<br>यह प्रश्न तभी पूछें जब प्रश्न एस 37 या एस 38 या एस40 का उत्तर "हां" हो; नहीं तो प्रश्न एस44 पर जायें।<br>How long after you first got married to your (last) husband did (this/any of these things) first happen?<br>अपने पति (पिछले) से विवाह होने के कितने समय बाद ऐसा/इनमें से कोई भी घटना पहली बार कब हुई?	|___||___| NUMBER OF YEARS<br>वर्षों की संख्या<br>99= DON'T KNOW<br>मालूम नहीं<br>IF LESS THAN ONE YEAR, RECORD 00<br>यदि एक वर्ष से कम तो 00 दर्ज करें		
S4	Did the following ever happen as a result of what your (last) husband did to you?<br>आपके (पिछले) पति ने आपके साथ जो किया क्या उसकी वजह से कभी ऐसा हुआ था?	YES<br>हां	NO<br>नहीं	
a.	You had cuts, bruises or aches?<br>आपको कटने का घाव लगना, चोट पहुंचना, शरीर दर्द करना?	1	0	
b.	You had burns?<br>ज्लना	1	0	
c.	You had eye injuries, sprains, dislocations?<br>आंखों में चोट, मोच आना, हड्डी का खिसकना, हल्के जलने का निशान	1	0	
d.	You had wounds, broken bones, broken teeth or other injury?<br>घाव के निशान, हड्डियों का टूटना, दांत टूटना या अन्य चोटें	1	0	
	**Instruction for interviewer:**<br>साक्षात्कारकर्ता के लिए निर्देश:<br>Check with the woman if she was married before the current husband and then continue.<br>यह जांच करें कि क्या वर्तमान पति के साथ विवाह से पूर्व महिला विवाहित थी और फिर जारी रखें।<br>1= YES, HAD PREVIOUS HUSBAND→**CONTINUE**<br>हां, पिछला पति था.........जारी<br>2= ONLY MARRIED ONCE→**GO TO S46**<br>केवल एक बार विवाह हुआ .... एस46 पर जायें			
S4	So far we have been talking about the behaviour of your(current/last) husband. Now I want to ask you	YES<br>हां	NO<br>नहीं	**ASK ONLY IF YES IN S44**<br>तभी पूछें जब प्र.44 का उत्तर "हां" हो<br>S45. How long ago did this happen?<br>एस45. यह कितने समय पहले हुआ था?

	about thebehaviour of any previous husband.<br>अब तक हम आपके (वर्तमान/पिछले) पति के व्यवहार के बारे में बात करते रहे हैं। अब मैं आपसे किसी पिछले पति के व्यवहार के बारे में प्रश्न पूछना चाहूंगी।			0-11 months<br>0–11 महीने पहले	12 or more months ago<br>12 या उससे अधिक महीने पहले	Don't rememb er<br>याद नहीं	
a.	Did any previous husband ever hit, slap, kick, or do anything else to hurt you physically?<br>क्या आपके पिछले किसी पति ने आपके मारा, थप्पड़ मारा, ठोकर भारी या शारीरिक रूप से आपको नुकसान पहुंचाने के लिए और कुछ किया था?	1	0	1	2	3	
b.	Did any previous husband physically forceyou to have intercourse or perform any other sexual acts against your will?<br>क्या आपने किसी पिछले पति ने आपकी इच्छा के विरुद्ध आपको यौन संपर्क करने के लिए शारीरिक रूप से मजबूर किया या अन्य यौन कार्य किया था?	1	0	1	2	3	
S4(	From the time you were 15 years old has anyone (other than your husband) ever hit you, slapped you, kicked you, or done anything else to hurt you physically?<br>15 वर्ष की होने के बाद से क्या किसी ने (आपके पति के अलावा) आपको कभी मारा, थप्पड़ मारा, ठोकर मारी या शारीरिक रूप से आपको नुकसान पहुंचाने के लिए और कुछ किया था?	1=YES<br>हां<br>0=NO **GO TO S49**<br>नहीं एस49 पर जायें					
S4'	Who has hurt you in this way?<br>आपको इस तरह से किसने चोट पहुंचाई थी?<br><br>**MULTIPLERESPONSE POSSIBLE**<br>अनेक उत्तर संभव हैं	1=MOTHER/STEP-MOTHER<br>मां/सौतेली मां<br>2=FATHER/STEP-FATHER<br>पिता/सौतेला पिता<br>3=SISTER/BROTHER<br>बहन/भाई<br>4=DAUGHTER/SON<br>लड़की/लड़का<br>5=OTHER RELATIVE<br>अन्य संबंधी<br>6=CURRENT BOYFRIEND<br>वर्तमान ब्वाय फ्रैंड<br>7=FORMER BOYFRIEND<br>पूर्व ब्वाय फ्रैंड<br>8=MOTHER-IN-LAW<br>सास<br>9=FATHER-IN-LAW<br>ससुर<br>10=OTHER IN-LAW<br>ससुराल के अन्य लोग<br>11=TEACHER<br>शिक्षक/शिक्षिका<br>12=EMPLOYER/SOMEONE AT WORK<br>मालिक/कार्य स्थल पर कोई व्यक्ति<br>13=POLICE/SOLDIER<br>पुलिस/सिपाही<br>88=OTHERS (SPECIFY)<br>अन्य (उल्लेख करें)					
S4{	In the last 12 months, how often has (this person/have these persons) physically hurt you: often, only sometimes, or not at all?<br>पिछले 12 महीनों में उसने (इस व्यक्ति/व्यक्तियों ने) आपको कब–कब चोट पहुंचाई: अक्सर, कभी–कभी, कभी नहीं?	1=OFTEN<br>अक्सर<br>2=SOMETIMES<br>कभी–कभी<br>3=NOT AT ALL<br>बिल्कुल नहीं					

S4[illegible]	Has anyone ever hit, slapped, kicked, or done anything else to hurt you physically or sexually during your MOST RECENT pregnancy?<br>क्या गर्भावस्था के दौरान आपको कभी किसी ने मारा, थप्पड़ मारा, ठोकर मारी या शारीरिक रूप से चोट पहुंचाने के लिए और कुछ किया था?	1=YES<br>हां<br>0=NO **GO TO S51**<br>नहीं एस51 पर जायें<br>77=NOT APPLICABLE **GO TO S51**<br>लागू नहीं एस51 पर जायें	
S5[illegible]	Who has done any of these things to physically or sexually hurt you during your MOST RECENT pregnancy?<br>गर्भवती होने के दौरान आपको शारीरिक रूप से चोट पहुंचाने के लिए ऐसा किसने किया था?<br><br>**MULTIPLE RESPONSE POSSIBLE**<br>अनेक उत्तर संभव हैं	1=CURRENT HUSBAND/PARTNER<br>वर्तमान पति/साझेदार<br>2=FORMER HUSBAND/PARTNER<br>पूर्व प9ति/साझेदार<br>3=CURRENT/FORMER BOYFRIEND<br>वर्तमान/पूर्व ब्याय फ्रैंड<br>4=FATHER/STEP-FATHER<br>पिता/ससुर<br>5=BROTHER/STEP-BROTHER<br>भाई/सौतेला भाई<br>6=OTHER RELATIVE<br>अन्य रिश्तेदार<br>7=IN-LAW<br>सास–ससुर<br>8=OWN FRIEND/ACQUAINTANCE<br>अपना मित्र/परिचित<br>9=FAMILY FRIEND<br>पारिवारिक मित्र<br>10=TEACHER<br>शिक्षक/शिक्षिका<br>11=EMPLOYER/SOMEONE AT WORK<br>फैक्ट्री का मालिक/कार्य स्थल पर कोई व्यक्ति<br>12=POLICE/SOLDIER<br>पुलिस/सिपाई<br>13=PRIEST/RELIGIOUS LEADER<br>पुजारी/धार्मिक नेता<br>14=STRANGER<br>अजनबी<br>88=OTHERS (SPECIFY)<br>अन्य (उल्लेख करें)	
S5[illegible]	Now I want to ask you about things that may have been done to you by someone other than (your/any) husband.<br>अब मैं आपसे ऐसी बातों के बारे में पूछना चाहूंगी जो (आपके/किसी) पति के अलावा और किसी ने आपके साथ की होंगी।<br>At any time in your life, as a child or as an adult, has anyone ever forced you in any way to have sexual intercourse or perform any other sexual acts when you did not want to?<br>अपने जीवन में किसी भी समय, एक बच्चे या वयस्क के रूप में, क्या किसी ने आपके साथ कभी जबर्दस्ती यौन संपर्क या कोई अन्य यौन कार्य किया है जो आप नहीं चाहती थीं?	1=YES<br>हां<br>0=NO **GO TO S55**<br>नहीं एस57 पर जायें	
S5[illegible]	Who was the person who was forcing you the very first time thishappened?<br>पहली बार जब ऐसा हुआ तो आपके साथ किस व्यक्ति ने जबर्दस्ती की थी?<br><br>**MULTIPLE RESPONSE POSSIBLE**<br>अनेक उत्तर संभव हैं	1=CURRENT HUSBAND/PARTNER<br>वर्तमान पति/साझेदार<br>2=FORMER HUSBAND/PARTNER<br>पूर्व प9ति/साझेदार<br>3=CURRENT/FORMER BOYFRIEND<br>वर्तमान/पूर्व ब्याय फ्रैंड<br>4=FATHER/STEP-FATHER<br>पिता/ससुर<br>5=BROTHER/STEP-BROTHER<br>भाई/सौतेला भाई<br>6=OTHER RELATIVE	

		अन्य रिश्तेदार<br>7=IN-LAW<br>सास–ससुर<br>8=OWN FRIEND/ACQUAINTANCE<br>अपना मित्र/परिचित<br>9=FAMILY FRIEND<br>पारिवारिक मित्र<br>10=TEACHER<br>शिक्षक/शिक्षिका<br>11=EMPLOYER/SOMEONE AT WORK<br>फैक्ट्री का मालिक/कार्य स्थल पर कोई व्यक्ति<br>12=POLICE/SOLDIER<br>पुलिस/सिपाई<br>13=PRIEST/RELIGIOUS LEADER<br>पुजारी/धार्मिक नेता<br>14=STRANGER<br>अजनबी<br>88=OTHERS (SPECIFY)<br>अन्य (उल्लेख करें)	
S5[illegible]	In the last 12 months, has anyoneother than (your/any) husbandphysically forced you to havesexual intercourse when you didnot want to?<br>पिछले 12 महीनों में क्या (आपके किसी) पति के अलावा किसी और ने आपके न चाहने पर भी आपके साथ जबर्दस्ती यौन संपर्क किया था?	1=YES<br>हां<br>0=NO<br>न्हीं	
S5[illegible]	How old were you the firsttime you were forced to time you were forced tohave sexual intercourse or perform any other sexual acts by anyone, including (your/any)husband?<br>जब आप कितने वर्ष की थीं जब (आपके किसी) पति के अलावा किसी और ने आपके साथ जबर्दस्ती यौन संपर्क या कोई अन्य यौन कार्य किया था?	|___||___| COMPLETED YEARS<br>पूरे वर्षों में	

SEEKING HELP ON DOMESTIC VIOLENCE<br>घरेलू हिंसा के लिए मदद प्राप्त करना			
Now I would like to ask you if you ever sought help to stop violence that you might be experiencing in your household which might be perpetrated by your husband or other members of your household.<br>अब मैं आपसे यह पूछना चाहूंगा कि अपने पति या परिवार के अन्य सदस्य द्वारा अपने खिलाफ घर पर होने वाली हिंसा को रोकने के लिए क्या आपने कभी मदद मांगी थी?			
S57.	**ASK IF YES IN S37 OR S38 OR S40 OR S44 OR S46 OR S49 OR S51, ELSE GO TO S60**<br>यह प्रश्न तभी पूछें जब प्र. एस37 या एस38 या एस40 या एस44 या एस46 या एस49 या एस51 का उत्तर हां हो; नहीं तो एस60 पर जायें।<br>Have you ever tried to seek help to stop your husband/other household members from doing this to you?<br>क्या आपने कभी अपने पति/परिवार के अन्य सदस्यों को अपने साथ ऐसा करने से रोकने के लिए कभी मदद मांगी है?	1=YES<br>हां<br>0=NO **GO TO S60**<br>नहीं एस60 पर जायें	
S58.	From whom have you sought help to stop this?<br>Anyone else?<br>इसे रोकने के लिए आपने किससे मदद मांगी?<br><br>**MULTIPLE RESPONSE POSSIBLE**<br>अनेक उत्तर संभव हैं	1=FATHER/MOTHER<br>पिता/माता<br>2=FATHER-IN-LAW/MOTHER-IN-LAW<br>ससुर/सास<br>3=OTHER RELATIVES OR FRIENDS<br>अन्य संबंधी या दोस्त<br>4=PANCHAYAT MEMBERS<br>पंचायत सदस्य<br>5=SHG GROUPS	

		स्वयं सहायता समूह<br>6=POLICE<br>पुलिस<br>7=RELIGIOUS LEADERS<br>धार्मिक नेता<br>8=NON-GOVERNMENT ORGANIZATION<br>गैर–सरकारी संस्था<br>88=OTHER (SPECIFY)<br>अन्य (उल्लेख करें)	
S59.	Did this violence stop because of the intervention of the concerned person whom you had approached for help?<br>आपने जिससे मदद मांगी क्या उसके हस्तक्षेप के बाद यह हिंसा रुक गई थी?	1=YES<br>हां<br>0=NO<br>न्हीं<br>77=NOT APPLICABLE<br>लागू नहीं	
S60.	Were you discouraged to voice against violence by your family or other community members?<br>क्या आपको आपके परिवार और समुदाय के अन्य सदस्यों ने हिंसा के खिलाफ आवाज उठाने से रोका था?	1=YES<br>हां<br>0=NO<br>न्हीं	
S61.	Who do you think you can approach to seek help for addressing the situation of violence against you?<br>अपने खिलाफ हिंसा की समस्या को हल करने के लिए आप सहायता पाने किसके पास जा सकती हैं?<br><br>**MULTIPLE RESPONSE POSSIBLE**<br>अनेक उत्तर संभव हैं	1=MOTHER-IN-LAW<br>सास<br>2=FATHER/MOTHER<br>पिता/माता<br>3=PANCHAYAT MEMBERS<br>पंचायत सदस्य<br>4=SHG GROUPS<br>स्वंय सहायता समूह<br>5=NON-GOVERNMENT ORGANIZATION<br>गैर–सरकारी संगठन<br>6=POLICE<br>पुलिस<br>88=OTHERS (SPECIFY)<br>अन्य (उल्लेख करें)	
S62.	Are there any groups or activists in your village who support women suffering from violence?<br>क्या आपके गांव में ऐसी संस्थाएं हैं या कार्यकलाप चलाये जाते हैं जो हिंसा से पीड़ित महिलाओं की मदद करे?	1=YES<br>हां<br>0=NO **GO TO S64**<br>नहीं एस64 पर जायें	
S63.	Please tell me what these groups are.<br>कृपया बताएं कि ये संस्थाएं/व्यक्ति कौन है?	1=PANCHAYAT MEMBERS<br>पंचायत सदस्य<br>2=SHG GROUPS<br>स्वयं सहायता समूह<br>3=NON-GOVERNMENT ORGANIZATION<br>गैर–सरकारी संगठन<br>88=OTHER (SPECIFY)<br>अन्य (उल्लेख करें)	
S64.	What should a woman do if she is facing violence within the family?<br>परिवार के अंदर हिंसा का सामना करने वाली महिला को क्या करना चाहिए?<br><br>**MULTIPLE RESPONSE POSSIBLE**<br>अनेक उत्तर संभव हैं	1=TALK TO OTHER MEMBERS OF HER IMMEDIATE FAMILY<br>अपने परिवार के दूसरे सदस्यों के साथ बात करना<br>2=TALK ABOUT IT TO RELATIVES SHARE WITH A FRIEND<br>अपने रिश्तेदारों या किसी मित्र से इस संबंध में बात करना<br>3=APPROACH THE PANCHAYAT FOR HELP<br>सहायता के लिए पंचायत के पास जाना<br>4=APPROACH THE POLICE FOR HELP<br>सहायता के लिए पुलिस के पास जाना<br>5=COUNSEL HER USBAND<br>पति को परामर्श देना<br>6=BECOME MORE ADJUSTING<br>अपने को अधिक ढालना<br>7=FIND OUT WHAT ANGERS HER HUSBAND AND AVOID DOING THOSE THINGS/ BEHAVE HERSELF PROPERLY	

		यह पता लगाना कि पति को किस बात पर गुस्सा आता है और उन कामो को नहीं करना/अपना व्यव्हार ठीक रखन<br>8=KEEP QUIET/ NOT DO ANYTHING<br>शांत रहना/कुछ न करना<br>88=OTHERS (SPECIFY)<br>अन्य (उल्लेख करें)<br>99= DON'T KNOW<br>मालूम नहीं	

**P. HOUSEHOLD CHARACTERISTICS (RC1/2/3/4/5/6/7/8)**<br>**This section will be asked to all categories**		
Read out to the respondent: *Now I will ask you some questions about your religion and caste.*<br>mÙkjnkrk dk i<+ dj lquk;s¿ vc eS vkils vkids /keZ vkj tkfr dk ckjs es iNwxhA		
P1.	Name of the respondent उत्तरदाता का नाम	
P2.	Line Number of respondent<br>उत्तरदाता का लाइन नंबर	☐☐
P3.	What is your religion?<br>आपका धर्म क्या है?<br><br>**CODE ONE ONLY**<br>Dzoy ,d dksM	1 = HINDU हिन्दू<br>2 = MUSLIM मुसलिम<br>3 = CHRISTIAN ईसाइ<br>4 = SIKH सिख<br>5 = BUDDHIST/NEO-BUDDHIST बौद्ध<br>6 = JAIN जैन<br>7 = JEWISH यहूदी<br>8 = PARSI/ZOROASTRIAN पारसी<br>9 = NO RELIGION कोई घर्म नहीं<br>88 = OTHER (SPECIFY) अन्य (स्पष्ट करें)
P4.	Do you belong to a scheduled caste, scheduled tribe, other backward class or general class?<br>क्या आपकी जाति अनुसूचित जाति, अनुसूचित जनजाति, अन्य पिछड़ी जाति या सामान्य है?	1 = SCHEDULED CASTE अनुसूचित जाति<br>2 = SCHEDULED TRIBE अनुसूचित जनजाति<br>3 = OTHER BACKWARD CASTE अन्य पिछड़ी जाति<br>4 = GENERAL सामान्य<br>88 = OTHER (SPECIFY) अन्य (स्पष्ट करें)
Read out to the respondent: Now I am going to ask you about some of the characteristics of your home.<br>**उत्तरदाता को पढ़ कर सुनायें : अब मैं आपसे आपके घर की विशेशताओं के बारे मे पूछूँगी।**		
P5.	Does your household own this house or any other house?<br>क्या आपका परिवार इस घर का या अन्य किसी घर का मालिक है?	1 = YES हाँ<br>0 = NO नहीं<br>99 = DO NOT KNOW पता नहीं
P6.	**LOOK AT THE FLOOR AND CODE THE MAIN MATERIAL OF THE FLOOR**<br>फर्श को देखें और फर्श के मुख्य पदार्थ को कोड करें।<br><br>**CODE ONE ONLY**<br>dzoy ,d dksM	1 = MUD/CLAY/EARTH मिट्टी/जमीन<br>2 = SAND बालू<br>3 = DUNG गोबर<br>4 = RAW WOOD PLANKS कच्ची लकड़ी से<br>5 = PALM/BAMBOO ताड़ या बासँ<br>6 = BRICK ईट<br>7 = STONE पत्थर<br>8 = PARQUET OR POLISHED WOOD लकड़ी से या पोलिष लकड़ी से<br>9 = VINYL OR ASPHALT विनाइल या डामार<br>10 = CERAMIC TILES चीनी मिट्टी की टाइल<br>11 = CEMENT सिमेंट<br>12 = CARPET कारपेट<br>13 = POLISHED STONE/MARBLE/GRANITE पोलिश पत्थर/मारबल/ग्रेनाइट<br>88 = OTHER (SPECIFY) अन्य (स्पष्ट करें)
P7.	**LOOK AT THE ROOF AND CODE THE MAIN MATERIAL OF THE ROOF**<br>छत को देखें और छत के मुख्य पदार्थ को कोड करें।<br><br>**CODE ONE ONLY**<br>dzoy ,d dksM	1 = NO ROOF छत नहीं है<br>2 = THATCH/PALM LEAF/REED/GRASS छप्पर/ताड़ की पत्ती/सरकंडा/घास<br>3 = MUD मिट्टी<br>4 = SOD/MUD AND GRASS MIXTURE घास में मिली हुई मिट्टी<br>5 = PLASTIC/POLYTHENE SHEETING

		प्लास्टिक/पोलिथीन सीट<br>6 = RUSTIC MAT सादी चटाई<br>7 = PALM/BAMBOO ताड़ या बाँस<br>8 = RAW WOOD PLANKS/TIMBER कच्ची लकड़ी से/लकड़ी<br>9 = UNBURNT BRICK कच्ची ईंट<br>10 = LOOSELY PACKED STONEखुला पत्थर<br>11 = METAL/GI धातु या जी. आई.<br>12 = WOOD लकड़ी<br>13 = CALAMINE/CEMENT FIBER कैलामाइन या सिमेंट फाइबर<br>14 = ASBESTOS SHEETS अदह शीट<br>15 = RCC/RBC/CEMENT/CONCRETE आर. सी. सी./आर. बी. सी./सिमेंट/कंक्रीट<br>16 = ROOFING SHINGLES नालीदार छत<br>17 = TILES टाईल<br>18 = SLATE स्लेट<br>19 = BURNT BRICK पक्की ईंट<br>88 = OTHER (SPECIFY) अन्य (स्पष्ट करें)	
P8.	**LOOK AT THE EXTERIOR WALLS AND CODE THE MAIN MATERIAL OF THE WALLS**<br>Ckkgjh nhokj dk n[k vkj nhokj d e[; inkFk dk dkM djA<br><br>**CODE ONE ONLY**<br>doy ,d dkM	1 = NO WALLS कोई दिवार नहीं<br>2 = CANE/PALM/TRUNKS/BAMBOO बेंत/ताड़/तना/बाँस<br>3 = MUD मिट्टी<br>4 = GRASS/REEDS/THATCH घास/सरकंडा/भूसा<br>5 = BAMBOO WITH MUD बाँस के साथ मिट्टी<br>6 = STONE WITH MUD पत्थर के साथ मिट्टी<br>7 = PLYWOOD प्लाईवुड<br>8 = CARDBOARD कार्डबोर्ड<br>9 = UNBURNT BRICK कच्ची ईंट<br>10 = RAW WOOD/REUSED WOOD कच्ची लकड़ी<br>11 = CEMENT/CONCRETE सीमेंट/कंक्रीट<br>12 = STONE WITH LIME/CEMENT पत्थर के साथ सीमेंट<br>13 = BURNT BRICKS पक्की ईंट<br>14 = CEMENT BLOCKS सिमेंट ब्लोक<br>15 = WOOD PLANKS/SHINGLES लकड़ी के पट्टे<br>16 = GI/METAL/ASBESTOS SHEETS मेटेल शीट<br>88 = OTHER (SPECIFY) अन्य (स्पष्ट करें)	
P9.	**TYPE OF WINDOWS** f[kMfd;k dk Ádkj<br>**RECORD OBSERVATION** n[kdj fy[k	Yes हाँ	No नहीं
a.	ANY WINDOWS कोई खिड़की	1	0 → **GO TO P10**
b.	WINDOWS WITH GLASS कँाच की खिड़की	1	0
c.	WINDOWS WITH SCREENS जाली वाली खिड़की	1	0
d.	WINDOWS WITH CURTAINS OR SHUTTERS शटर/पर्दे वाली खिड़की	1	0
P10.	How many rooms in this house are used for sleeping?<br>इस घर मे सोने के लिये कितने कमरे प्रयोग किये जाते हैं?<br>**PROBE: CODE "0" IF SLEEPING OUTSIDE**<br>Ákc % ;fn ?kj d ckgj lkr g rk 0 dkM djA	☐☐ NUMBER OF ROOMS कमरों की संख्या	
P11.	Do you have a separate room which is used as a kitchen? क्या आपके घर में कोई अलग कमरा है जिसे आप रसोई की तरह प्रयोग करती हैं?	1 = YES हाँ<br>0 = NO नहीं	
P12.	What type of fuel does your household mainly use for cooking?<br>आपका परिवार खाना बनाने के लिए मुख्यतः कौन सा ईंधन प्रयोग करता है?<br><br>**CODE ONE ONLY**<br>doy ,d dkM<br><br>**PROBE : FOR MAIN SOURCE**	1 = ELECTRICITY बिजली<br>2 = LPG/NATURAL GAS एलपीजी/प्राकृतिक गैस<br>3 = BIOGAS बायोगैस<br>4 = KEROSENE केरोसीन/मिट्टी का तेल<br>5 = COAL/LIGNITE कोयला/लकड़ी का कोयला<br>6 = CHARCOAL चारकोयला<br>7 = WOOD लकड़ी<br>8 = STRAW/SHRUBS/GRASS खर/झाड़ी/घास	

	çkc % e[; l®r iNA	9 = AGRICULTURAL CROP WASTE खराब फसल<br>10 = DUNG CAKES गोबर के उपल<br>88 = OTHER (SPECIFY) अन्य (स्पष्ट करें)
P13.	What is the main source of lighting in this household?<br>इस घर की रोशनी का मुख्य स्रोत क्या हैं?<br><br>**CODE ONE ONLY**<br>doy ,d dkM	1 = LANTERN लालटेन<br>2 = KEROSENE LAMP केरोसिन कुप्पी<br>3 = CANDLE मोमबत्ती<br>4 = ELECTRIC बिजली<br>5 = LPG/BATTERYरसोइ गैस/बैटरी<br>6 = SOLAR ENERGY सौर्य ऊजा<br>7 = NONE कुछ नहीं<br>88 = OTHERS (SPECIFY) अन्य (स्पष्ट करें)
P14.	What is the main source of drinking water for members of your household?<br>आपके परिवार के सदस्यों के लिये पीने के पानी का मुख्य स्रोत क्या है ?<br><br>**PROBE : FOR MAIN SOURCE. PLEASE SHOW PICTURES.**<br>çkc % e[; l®r iNA Ñl;k fp= fn[kkb;A<br><br>**CODE ONE ONLY**<br>doy ,d dkM	1 = PIPED INTO DWELLING घर में पाइप<br>2 = PIPED TO YARD/PLOT आँगन/प्लाट का पाइप<br>3 = PUBLIC TAP/STANDPIPE पब्लिकटोटी/स्टैंडपाइप<br>4 = TUBE WELL/BOREHOLE टयूबबेल/बोरहोल<br>5 = PROTECTED WELLढका कुआँ<br>6 = UNPROTECTED WELLखुला कुआँ<br>7 = PROTECTED SPRING सुरक्षित मौसमी पानी<br>8 = UNPROTECTED SPRING असुरक्षित मौसमी पानी<br>9 = RAINWATER बरसात का पानी<br>10 = TANKER TRUCK टैंकर ट्रक<br>11 = CART WITH SMALL TANK छोटे टैंक वाली गाड़ी<br>12 = SURFACE WATER (RIVER/DAM/LAKE/POND/ STREAM/CANAL/IRRIGATION CHANNEL) सतही पानी (नदी/नाला/झील/तालाब/धारा/नहर/सिंचाई)<br>13 = BOTTLED WATER बोतल का पानी<br>14 = GOVERNMENT HAND PUMP सरकारी हैंडपम्प<br>15 = HAND PUMP AT HOME घर में हैंडपम्प<br>16 = NEIGHBOUR'S HAND PUMP पडोसी का हैंडपम्प<br>88 OTHER (SPECIFY) अन्य (स्पष्ट करें)
**P14A**	DO NOT ASK IF P14=1/2/9/13/15<br><br>How much time does it take to walk till the water source from your home?<br>आपके घर से पानी के श्रोत तक चलके पहुचने में कितना समय लगता है?	☐☐ NUMBER OF MINUTES
P15.	Do you treat your water in any way to make it safer to drink?<br>क्या आप पानी को पीने योग्य बनाने के लिये बनाने के लिए कुछ करती हैं?	1 = YES हाँ<br>0 = NO नहीं → **GO TO P16A**<br>99 = DO NOT KNOW पता नहीं → **GO TO P16A**
P16.	What do you usually do to the water to make it safer to drink?<br>सामान्यतया आप पानी को पीने योग्य बनाने के लिये क्या करती हैं?<br><br>**PROBE : ANY OTHERS?**<br>çkc % dN vkj\<br><br>**RECORD ALL MENTIONED**<br>lHkh mÙkj fy[kA	1 = BOIL उबालना<br>2 = USE ALUM फिटकरी का प्रयोग<br>3 = ADD BLEACH/CHLORINE TABLETS ब्लीच/क्लोरीन की टेबलेट को मिलाना<br>4 = STRAIN THROUGH A CLOTH कपड़े से छान्ना<br>5 = USE WATER FILTER (CERAMIC/SAND/COMPOSITE/ETC.) पानी के फिल्टर का इस्तेमाल करना(सेरेमिक/बालू/कम्पोजिट)<br>6 = USE ELECTRONIC PURIFIER बिजली के पयूरिफायर का इस्तेमाल करना<br>7 = LET IT STAND AND SETTLE पानी को ठहरे रहने देना और स्थिर होने देना<br>88 = OTHER (SPECIFY) अन्य (स्पष्ट करें)

		99 DO NOT KNOW पता नहीं
**P16A**	Does this household have any mosquito nets that can be used for sleeping?<br><br>क्या इस परिवार के पास मच्छर दानी हैजो सोने के समय इस्तेमाल की जा सके?	1 = YES हाँ<br>0 = NO नहीं
**P16B**	Does this household have insecticide treated nets that can be used for sleeping?<br><br>क्या इस परिवार के पास कीनाचक युक्त मच्छर दानी है जो सोने के समय इस्तेमाल की जा सके?	1 = YES हाँ<br>0 = NO नहीं
**P16C**	ASK IF P16A=1 OR P16B=1<br><br>Does this household use mosquito nets (treated/untreated) for sleeping children aged 0-5 years?<br><br>क्या आपके परिवार में 0-५ साल के उम्र के बच्चो को सुलाते वक़्त मछार दानी का इस्तेमाल किया जाता है?	1 = YES हाँ<br>0 = NO नहीं
**P16D**	Has your household ever been treated with indoor residual spraying?<br>क्या आपके घर में कभी कीड़े मारने वाली स्प्रे हुआ है?	1 = YES हाँ<br>0 = NO नहीं
**P16E**	If yes, when was the last time it was sprayed?<br>कितने समय पहले वोह स्प्रे हुआ था?	☐☐ NUMBER OF TIMES
P17.	What kind of toilet facility do members of your household usually use?<br>आपके परिवार के सदस्य किस प्रकार का शौचालय इस्तेमाल करते हैं?<br><br>**PLEASE SHOW PICTURES**<br>ÑI;k fp= fn[kkb;A<br><br>**CODE ONE ONLY**<br>doy ,d dkM	1 = FLUSH TO PIPED SEWER SYSTEM पाइप सीवर सिस्टम से फ्लश<br>2 = FLUSH TO SEPTIC TANK सेप्टिक टैंक से फ्लश<br>3 = FLUSH TO PIT LATRINE गडढ़े वाले शौचालय से फ्लश<br>4 = FLUSH TO SOMEWHERE ELSE कहीं और फ्लश<br>5 = FLUSH, DON'T KNOW WHERE फ्लश, पता नहीं कहाँ<br>6 = VENTILATED IMPROVED PIT (VIP)/BIOGAS LATRINE हवा वाला बेहतर गडढ़ा/बायोगैस शौचालय<br>7 = PIT LATRINE WITH SLAB पट्टीवाले गडढ़े वाले शौचालय<br>8 = PIT LATRINE WITHOUT SLAB/ OPEN PIT बिना पट्टीवाले गडढ़े वाले शौचालय/खुला गडढ़ा<br>9 = TWIN PIT/COMPOSTING TOILET जुड़े हुये गडढ़े/कम्पोजिट शौचालय<br>10 = DRY TOILET खुला शौचालय<br>11 = NO FACILITY/USES OPEN SPACE OR FIELD कोई सुविधा नहीं/खुले स्थान का इस्तेमाल या खेत में → **GO TO 0**<br>88 = OTHER (SPECIFY) अन्य (स्पष्ट करें)
P18.	In the past week has anyone in your household including children defecated in the open for example in the field or in the river?<br>पिछले सप्ताह में क्या आपके घर का कोई सदस्य या बच्चा शौच के लिए खुले में गया जैसे कि खुले मैदान में या नदी के पास ?	1 = YES हाँ<br>0 = NO नहीं
**P18a0.**	Where do you generally defecate: use toilets or go out in open?<br>आम तौर पे आप शौच कहाँ करते हैं : शौचालय में या खुले में ?	1= USE TOILET शौचालय में<br>2= DEFECATE IN OPEN खुले में शौच
**P18a**	**ASK ONLY FOR CATEGORY 1,2 AND 3**<br>**IF** [CHILD NAME] **IS ALIVE, ASK:**<br>;fn ¼cPp dk uke½ thfor g] iN% ¾<br><br>The last time [CHILD NAME] passed stools, what was done to dispose of the stools?<br>अन्तिम बार जब (बच्चे का नाम) ने मल/पखाना किया था, तब मल को कहाँ फेंका गया था?	1 = CHILD USED TOILET/ LATRINE बच्चे ने शौचालय प्रयोग किया<br>2 = CHILD DEFECATED IN THE DRAIN बच्चे ने नाली में शौच किया<br>3 = CHILD DEFECATED IN THE OPEN बच्चे ने खुले में शौच किया

		3 = PUT/RINSED INTO TOILET OR LATRINE शौचालय में धोया<br>3 = PUT/RINSED INTO DRAIN OR DITCH नाली या नल पर धोया था<br>4 = THROWN INTO GARBAGE कूड़े में डाल दिया<br>5 = BURIED गाढ़ दिया<br>6 = LEFT/WASHED IN THE OPEN खुले में डाला/धो दिया था<br><br>= THROWN IN OPEN AFTER WRAPPING लपेट कर खुले में फेंका<br><br>= THROWN IN OPEN WITHOUT WRAPPING बिना लपेटे खुले में फेंका<br>88 = OTHER (SPECIFY) अन्य (स्पष्ट करें)<br>99 = DO NOT KNOW पता नहीं
P20.	On what occasions did you wash your hands with soap since this time yesterday?<br>कल इस समय से आज इस समय तक, आपने कब– कब औसरों पर साबुन से हाथ धोये थे?<br><br>**PROBE : ANY OTHERS?**<br>çkc % dIN vkj\<br><br>**RECORD ALL MENTIONED**<br>lHkh mÙkj fy[kA	1 = NO SOAP AVAILABLE IN THE HOUSEHOLD घर में साबुन नहीं है<br>2 = SOAP AVAILABLE IN THE HOUSEHOLD BUT DID NOT USE IT TO WASH HANDS घर में साबुन है, पर हाथ धोते समय प्रयोग नहीं करते<br>3 = BEFORE COOKING खाना बनाने के पहले<br>4 = AFTER HANDLING FOOD खाना परोसने के बाद<br>5 = BEFORE EATING खाना खाने के पहले<br>6 = BEFORE FEEDING THE INFANT बच्चे को खाना खिलाने के पहले<br>7 = AFTER CLEANING INFANT FECES बच्चे का पखाने धोने के बाद<br>8 = WHEN I WASH CLOTHES कपड़े धोते समय<br>9 = WHEN I BATHE नहाते समय<br>10 = WHEN I WASH DISHES बर्तन धोते समय<br>11 = AFTER USING TOILET शौचालय जाने के बाद<br>88 = OTHER SPECIFY अन्य (स्पष्ट करें)
P21a	OBSERVE AND NOTE<br>Is there soap and water available near the place where food is prepared?<br>क्या खाना बनाने वाले जगह पे पानी और साबुन उपलब्ध है?	1= Yes हाँ<br>0त्रNo नहीं

Read out to the respondent: Now I am going to ask you about the assets that your household has.
mÙkjnkrk dk i< dj lquk;s % vc e vkils vkid ifjokj d ikl miyC/k lifRr d ckj e iNxhA

P21.	Does your household have क्या आपके घर में ....है	Yes हाँ	No नहीं
a.	Electricity बिजली	1	0
b.	A mattress गद्दा	1	0
c.	A pressure cooker प्रेशर कूकर	1	0
d.	A chair कुर्सी	1	0
e.	A cot or bed चारपाई या बिस्तर	1	0
f.	A table मेज	1	0
g.	An electric fan बिजली वाला पंखा	1	0
h.	A radio or transistor रेडियो या ट्राजिस्टर	1	0
i.	A black and white television ब्लैक एण्ड व्हाइट टेलीविजन	1	0
j.	A color television रंगीन टेलीविजन	1	0
k.	A sewing machine सिलाई मशीन	1	0
l.	A mobile telephone मोबाइल	1	0
m.	Any other type of telephone किसी अन्य प्रकार का टेलीफोन	1	0
n.	A computer/laptop कम्प्यूटर/लेपटाप	1	0
o.	A refrigerator रेफ्रिजरेटर	1	0
p.	A watch or clock घड़ी	1	0
q.	A bicycle साइकिल	1	0
r.	A motorcycle or scooter मोटर साइकिल या स्कूटर	1	0
s.	An animal-drawn cart जानवरों द्वारा खीची जाने वाली गाड़ी	1	0
t.	A car कार	1	0
u.	A water pump पानी का पम्प	1	0
v.	A thresher थ्रेशर	1	0

w.	A tractor ट्रैक्टर	1	0
x.	Internet ईन्टरनेट	1	0
y.	Air Conditioner or Cooler एयर कन्डीश्नर या कुलर	1	0
z.	Washing Machine कपड़े धोने की मशीन	1	0
P22A1	Do you have salt available in the house?<br>क्या आपके घर में नमक है?	1 = YES हाँ<br>0 = NO नहीं	
P22A3	Do you use packaged or loose salt?<br>क्या आप पैकेट वाला या खुला नमक इअतेमाल करते हैं?	PACKAGED……………….1<br>पैकेट वाला नमक<br>LOOSE……………………..2<br>खुला नमक	
P22A2	Do you use iodized salt?<br><br>क्या वो नमक आयोडाईजड नमक है?	1 = YES हाँ<br>0 = NO नहीं<br>9 = DO NOT KNOW पता नहीं	
P23	In the past four weeks, did your household ever run out of money to buy food?<br>पिछले चार सप्ताह में क्या भोजन खरीदने के लिये आपके पास पैसा नहीं था?	1 = YES हाँ<br>0 = NO नहीं	
P24	In the past four weeks, did you ever limit the types of food you fed your children because you did not have enough money to buy food for a meal?<br>पिछले चार सप्ताह में भोजन खरीदने के लिये पर्याप्त पैसा न होने के कारण क्या आपको बच्चे के भोजन के प्रकार सीमित करना पड़ा था?	1 = YES हाँ<br>0 = NO नहीं	
P25	In the past four weeks, did you cut the size of meals or skip meals because there was not enough money for food?<br>पिछले चार सप्ताह में भोजन खरीदने के लिये पर्याप्त पैसा न होने के कारण क्या आपको अपने भोजन में कटौती करने पड़ा था या कुछ कम बार भोजन खाया था?	1 = YES हाँ<br>0 = NO नहीं	
P26	In the last four weeks, did you or any household member go to sleep at night hungry because there was not enough food?<br>पिछले चार सप्ताह क्या पर्याप्त भोजन न होने के कारण आपको या आपके परिवार के किसी सदस्य को भूखा सोना पड़ा था?	1 = YES हाँ<br>0 = NO नहीं	
P27	In the last four weeks, did you have to mortgage any household assets to buy food?<br>पिछले चार सप्ताह में क्या भोजन खरीदने के लिये आपको अपने परिवार की कोई सम्पत्ति गिरवी रखनी पड़ी थी?	1 = YES हाँ<br>0 = NO नहीं	
P28	Have you ever faced any problems in accessing health care when you were sick?<br>**क्या आपने कभी स्वस्थ सुविधा का इस्तेमाल करने में कोई बाधा महसूस किया है?**	1 = YES हाँ<br>0 = NO नहीं	
P29	ASK IF YES FOR P28<br>What problems did you face?<br>**क्या बाधा आई?**	1=DID NOT HAVE ENOUGH MONEY FOR TRANSPORT<br>स्वस्थ्य केंद्र तक जाने के लिए गाडी के लिए पैसे नहीं थे<br>2=DID NOT HAVE ENOUGH MONEY FOR DOCTOR FEES<br>डॉक्टर की फीस के पैसे नहीं थे<br>3=DID NOT HAVE MONEY FOR MEDICINES<br>दवाईयों के लिए पैसे नहीं थे<br>88=OTHERS	
P30	What are the different food groups available in your household currently?<br>आपके घर में इस वक़्त कौन कौन से खाने की सामग्री अभी उपलब्ध है?<br><br>**PROBE : ANY OTHERS?**<br>çkc % dqN vkj\<br><br>**RECORD ALL MENTIONED**<br>lHkh mÙkj fy[kA	1 = STARCH STAPLE FOOD स्टार्च युक्त भोजन<br>(Rice, Roti, Bread, Pressed rice, Muri, Potato, sweet potato, arbi, Cassava, Banana, any other roots and tubers, noodles, pasta) (चावल, रोटी, ब्रेड, चिवड़ा, कसावा, आलू, शकरकंदी, सूरन, कसावा, केला, अन्य तववजे ंदक जनइमते, नूडल, पास्ता)<br>2 = BEANS AND PEAS फलियाँ और दाल	

		(Beans, peas, Mung dal, Chana dal, Arahar dal, Urad dal, kidney beans, Matter dal, other pulse and Soybeans, Seem, mustard) (फलियाँ, मटर, मूंग दाल, चना दाल, अरहर दाल, उरद दाल, राजमा, मटर दाल, अन्य दाल और सोयाबीन, सेम, सरसों)<br>3 = NUTS AND SEEDS मेवा और बीज<br>(Groundnut, Apricort, Almond, Pista, Coconut, Seesam seed, lotus seed, Jackfruit seed, cashew nut, walnut, mustard seed, sunflower seed, pumpkin seeds) (मुन्गफली, खुबानी, बदाम, पिस्ता, नारियल, तिल का बीज, कमल का बीज, कटहल का बीज,<br>काजू, अखरोड, सरसों का बीज, सूरजमुखी का बीज, कद्दु, का बीज)<br>4 = DAIRY दूध या दूध उत्पाद<br>(Milk – cow/goat/buffalo, Powder milk, Liquid packet milk, Curd, Paneer, sewai, kheer, sweets, Butter, Ghee) (दूध – गाय/बकरी/भैस, डिब्बे का दूध, पैकेट का दूध, दही, पनीर, सेवाई, खीर, मिठाई, मक्खन, घी)<br>5 = FLESH FOOD माँसाहार भोजन<br>(Meat – Lamb/Beef/pork, Organ meat, chicken, duck, other birds, fish – big/small/shell fish/prawn) (माँस – भेंड़/बकरी/गाय/बकरी/भैंस/सूअर, मछली – बड़ी/छोटी/कवचधारी/झींगा)<br>6 = EGGS अन्डा<br>(Eggs – Chicken/duck/other bird) (अन्डा – मुर्गी/बतख/अन्य पक्शी)<br>7 = VITAMIN A DARK GREEN विटामिन ए युक्त हरी सबजियाँ<br>(Leafy vegetable, palak saag, lal saag, green saag, pumpkin saag, mustard saag, matter saag, methi saag, muli saag, any other saag) (पत्तेदार सबजियाँ, पालक का साग, लाल साग, हरा साग, कद्दु का साग, सरसों का साग, मटर का साग, मेथी का साग, मुली का साग, कोई और साग)<br>8 = OTHER VITAMIN A RICH VEGETABLES AND FRUITS अन्य विटामिन ए युक्त सबजियाँ और फल<br>(Ripe mangoes, ripe papaya, jack fruit, ripe tomato, carrot, pumpkin) (पका आम, पका पपीता, कटहल, पका टमाटर, गाजर, कद्दु)<br>9 = OTHER VEGETABLES अन्य सबजियाँ<br>(Green papaya, cabbage, cauliflower, eggplant, onion, radish, bitter gourd, bottle gourd, coriander leaves, tomato, pointed gourd, ladies finger, green banana, garlic, ginger) (हरा पपीता, बन्दगोभी, गोभी, बैगन, पेयाज, मूली, करेला, लौकी, धनिया पत्ती, टमाटर, परवल, भिंडी, हरा केला, लेहसुन, अदरक)<br>10 = OTHER FRUITS अन्य फल<br>(Citrus fruits – orange/lemon/malta, apple, guava, pear, watermelon) (खट्टे फल – संतरा/नीबू/मान्लटा, सेब, नाशपाती, तरबूज़)	

		88=OTHERS	
P31	Yesterday (24 hours from morning till night), what types of food and liquids did you consume?<br><br>पिछले 24 घंटे में आपने किस किस प्रकार के तरल और ठोस पदार्थ खाए है?<br><br>**PROBE : ANY OTHERS?**<br>çkc ¶ dN vkj\<br><br>**RECORD ALL MENTIONED**<br>lHkh mÙkj fy[kA	1 = STARCH STAPLE FOOD स्टार्च युक्त भोजन<br>(Rice, Roti, Bread, Pressed rice, Muri, Potato, sweet potato, arbi, Cassava, Banana, any other roots and tubers, noodles, pasta) (चावल, रोटी, ब्रेड, चिवड़ा, कसावा, आलू, शकरकंदी, सूरन, कसावा, केला, अन्य तववजे ंदक जनइमते, नूडल, पास्ता)<br>2 = BEANS AND PEAS फलियाँ और दाल<br>(Beans, peas, Mung dal, Chana dal, Arahar dal, Urad dal, kidney beans, Matter dal, other pulse and Soybeans, Seem, mustard) (फलियाँ, मटर, मूंग दाल, चना दाल, अरहर दाल, उरद दाल, राजमा, मटर दाल, अन्य दाल और सोयाबीन, सेम, सरसों)<br>3 = NUTS AND SEEDS मेवा और बीज<br>(Groundnut, Apricort, Almond, Pista, Coconut, Seesam seed, lotus seed, Jackfruit seed, cashew nut, walnut, mustard seed, sunflower seed, pumpkin seeds) (मुन्गफली, खुबानी, बदाम, पिस्ता, नारियल, तिल का बीज, कमल का बीज, कटहल का बीज,<br>काजू, अखरोड, सरसों का बीज, सूरजमुखी का बीज, कद्दु, का बीज)<br>4 = DAIRY दूध या दूध उत्पाद<br>(Milk – cow/goat/buffalo, Powder milk, Liquid packet milk, Curd, Paneer, sewai, kheer, sweets, Butter, Ghee) (दूध – गाय/बकरी/भैस, डिब्बे का दूध, पैकेट का दूध, दही, पनीर, सेवाई, खीर, मिठाई, मक्खन, घी)<br>5 = FLESH FOOD माँसाहार भोजन<br>(Meat – Lamb/Beef/pork, Organ meat, chicken, duck, other birds, fish – big/small/shell fish/prawn) (माँस – भेंड़/बकरी/गाय/बकरी/भैंस/सूअर, मछली – बड़ी/छोटी/कवचधारी/झींगा)<br>6 = EGGS अन्डा<br>(Eggs – Chicken/duck/other bird) (अन्डा – मुर्गी/बतख/अन्य पक्शी)<br>7 = VITAMIN A DARK GREEN विटामिन ए युक्त हरी सबजियाँ<br>(Leafy vegetable, palak saag, lal saag, green saag, pumpkin saag, mustard saag, matter saag, methi saag, muli saag, any other saag) (पत्तेदार सबजियाँ, पालक का साग, लाल साग, हरा साग, कद्दु का साग, सरसों का साग, मटर का साग, मेथी का साग, मुली का साग, कोई और साग)<br>8 = OTHER VITAMIN A RICH VEGETABLES AND FRUITS अन्य विटामिन ए युक्त सबजियाँ और फल<br>(Ripe mangoes, ripe papaya, jack fruit, ripe tomato, carrot, pumpkin) (पका आम, पका पपीता, कटहल, पका टमाटर, गाजर, कद्दु)<br>9 = OTHER VEGETABLES अन्य सबजियाँ	

		(Green papaya, cabbage, cauliflower, eggplant, onion, radish, bitter gourd, bottle gourd, coriander leaves, tomato, pointed gourd, ladies finger, green banana, garlic, ginger) (हरा पपीता, बन्दगोभी, गोभी, बैगन, पेयाज, मूली, करेला, लौकी, धनिया पत्ती, टमाटर, परवल, भिंडी, हरा केला, लेहसुन, अदरक)<br>10 = OTHER FRUITS अन्य फल (Citrus fruits – orange/lemon/malta, apple, guava, pear, watermelon) (खट्टे फल – संतरा/नीबू/मान्लटा, सेब, नाशपाती, तरबूज़)<br>88=OTHERS<br>99= NONE	

U. ANTHROPOMETRY (RC1/2/3/4/5/7/8)		
Read out to the respondent: I'd like to measure [CHILD NAME's] weight and height. I'm going to take each measurement two times to make sure I get an accurate measurement.<br>mÙkjnkrk dk i< dj lquk;s ¼ ge ½cPps dk uke½ dk otu vkj yEckbZ ukiuk pkgrs gSA ge 2 ckj ÁR;sd uki yxk ftlls ge lgh lgh uki ys ldsA<br>**MEASURE ONLY [CHILD NAME] LYING DOWN.** ¼cPps dk uke½ dh yEckbZ doy fyVk dj ukisA		
Q1.	**RECORD DATE OF BIRTH**<br>बच्चे की जन्म की तारीख नोट करें<br>ASK FOR [CHILD NAME's] DATE OF BIRTH AND COMPARE WITH THE HOUSEHOLD ROSTER TO CONFIRM<br>(बच्चे का नाम) की जन्म की तारीख पुछें और उसे रोस्टर में दी गई तारीख से मिलायें।	☐☐ DD दिनांक<br>☐☐ MM महिना<br>☐☐☐☐ YYYY साल
Q2.	**RECORD AGE OF [CHILD NAME]**<br>¼cPps dk uke½ dh vk;q ntZ djs<br>CONFIRM AGE IS CONSISTENT WITH INSTRUCTIONS.<br>सुनिश्चित करें कि आयु बताये निंदेशों के अनुसार है।	☐☐ IN COMPLETED MONTHS पूर्ण महीनों में
Q3.	RECORD WHETHER WEIGHT WAS TAKEN OR NOT AND REASON WHY NOT.<br>नोट करें कि वजन लिया गया था या नहीं और यदि नहीं तो क्यों नहीं	1 = MEASURED तौला गया<br>2 = CHILD ABSENT बच्चा नहीं था → **GO TO Q6**<br>3 = CHILD REFUSED बच्चे ने मनाकर दिया → **GO TO Q6**<br>4 = MOTHER REFUSED माँ ने मनाकर दिया → **GO TO Q6**<br>5 = CHILD DEFORMED बच्चा विकलांग → **GO TO Q6**<br>6 = CHILD ILL बच्चा बीमार → **GO TO Q6**<br>7 = CHILD DEAD बच्चा मृत → **GO TO Q6**<br>88 = OTHER (SPECIFY) अन्य (स्पष्ट करें) → **GO TO Q6**
Q4.	RECORD WEIGHT (G) OF THE MOTHER AND CHILD माँ और बच्चे का वजन नोट करें	
a.	FIRST MEASUREMENT पहला माप	☐☐. ☐☐ KILOGRAMS किलोग्राम
b.	SECOND MEASUREMENT दूसरा माप	☐☐. ☐☐ KILOGRAMS किलोग्राम
Q5.	RECORD WEIGHT (G) OF THE MOTHER/ADOLESCENT माँ/किशोरी का वजन नोट करें	
C.	FIRST MEASUREMENT पहला माप	☐☐. ☐☐ KILOGRAMS किलोग्राम
D	SECOND MEASUREMENT दूसरा माप	☐☐. ☐☐ KILOGRAMS किलोग्राम
Q6.	RECORD WHETHER HEIGHT OF CHILD WAS TAKEN OR NOT AND REASON WHY NOT.<br>नोट करें कि लम्बाई लिया गया था या नहीं और यदि नहीं तो क्यों नहीं	1 = MEASURED नापा गया<br>2 = CHILD ABSENT बच्चा नहीं था →<br>3 = CHILD REFUSED बच्चे ने मनाकर दिया →<br>4 = MOTHER REFUSED माँ ने मनाकर दिया →<br>5 = CHILD DEFORMED बच्चा विकलांग →<br>6 = CHILD ILL बच्चा बीमार →<br>7 = CHILD DEAD बच्चा मृत →<br>88 = OTHER (SPECIFY) अन्य (स्पष्ट करें) →<br>**IF NOT EQUAL TO 1, GOTO 7A**

Q7.	RECORD HEIGHT (CM) OF THE CHILD की बच्चे लंबाई नोट करें	
C	FIRST MEASUREMENT पहला माप	□□.□□ CM से.मी.
D	SECOND MEASUREMENT दूसरा माप	□□.□□ CM से.मी.
7A	RECORD WHETHER HEIGHT OF MOTHER/ADOLESCENT WAS TAKEN OR NOT AND REASON WHY NOT.<br>नोट कर माँ/किशोरी ` कि लम्बाई लिया गया था या नहीं और यदि नहीं 'तो क्यों नहीं	1 = MEASURED नापा गया<br>2 = MOTHER REFUSED माँ ने मनाकर दिया →<br>88 = OTHER (SPECIFY) अन्य (स्पष्ट करें) →<br><br>**IF 2 OR 88 END INTERVIEW**
	RECORD HEIGHT (CM) OF THE MOTHER लंबाई नोट करें	
A.	FIRST MEASUREMENT पहला माप	□□.□□ CM से.मी.
B.	SECOND MEASUREMENT दूसरा माप	□□.□□ CM से.मी.
Q8.	RECORD WHETHER HEAMOGLOBIN OF THE CURRENTLY PREGNANT WOMAN WAS TAKEN OR NOT AND REASON WHY NOT.<br>नोट करें कि महिला का हिमोगलोबिन लिया गया था या नहीं और यदि नहीं 'तो क्यों नहीं	1 = MEASURED नापा गया<br>2 = MOTHER REFUSED माँ ने मनाकर दिया<br>88 = OTHER (SPECIFY) अन्य (स्पष्ट करें)<br><br>**IF 2 OR 88 END INTERVIEW**
Q9.	RECORD HEAMOGLOBIN (GM/DL) हिमोगलोबिन नोट करें	
a.	MEASUREMENT माप	□□.□□ GM/DL से.मी.
b.	MEASUREMENT माप	□□.□□ GM/DL से.मी.
Q10.	Those are all the questions I have. Thank you so much for taking the time to speak with me.<br>मैं आपसे यही सब प्रश्न पूछना चाहती थी । मुझसे बात करने के लिए आप ने अपना समय निकाला इसके लिये आपको धन्यवाद।<br>**RECORD TIME WHEN YOU END THE INTERVIEW.**<br>'kk{kkRdkjdrk 'kk{kkRdkj lekIr djus dk le; fy[kA	□□ HOUR घंटे<br>□□ MINUTES मिनट

END lekIr
